# Recent climate change has driven divergent hydrological shifts in high-latitude peatlands

**DOI:** 10.1038/s41467-022-32711-4

**Published:** 2022-08-24

**Authors:** Hui Zhang, Minna Väliranta, Graeme T. Swindles, Marco A. Aquino-López, Donal Mullan, Ning Tan, Matthew Amesbury, Kirill V. Babeshko, Kunshan Bao, Anatoly Bobrov, Viktor Chernyshov, Marissa A. Davies, Andrei-Cosmin Diaconu, Angelica Feurdean, Sarah A. Finkelstein, Michelle Garneau, Zhengtang Guo, Miriam C. Jones, Martin Kay, Eric S. Klein, Mariusz Lamentowicz, Gabriel Magnan, Katarzyna Marcisz, Natalia Mazei, Yuri Mazei, Richard Payne, Nicolas Pelletier, Sanna R. Piilo, Steve Pratte, Thomas Roland, Damir Saldaev, William Shotyk, Thomas G. Sim, Thomas J. Sloan, Michał Słowiński, Julie Talbot, Liam Taylor, Andrey N. Tsyganov, Sebastian Wetterich, Wei Xing, Yan Zhao

**Affiliations:** 1 Key Laboratory of Cenozoic Geology and Environment, Institute of Geology and Geophysics, Chinese Academy of Sciences, Beijing, China; 2 Environmental Change Research Unit (ECRU), Ecosystems and Environment Research Programme, University of Helsinki, Helsinki, Finland; 3 Helsinki Institute of Sustainability Science (HELSUS), Helsinki, Finland; 4 Geography, School of Natural and Built Environment, Queen’s University Belfast, Belfast, UK; 5 Ottawa-Carleton Geoscience Centre and Department of Earth Sciences, Carleton University, Ottawa, Canada; 6 Mathematics Research Centre CIMAT, Guanajuato, Mexico; 7 Geography, College of Life and Environmental Sciences, University of Exeter, Exeter, UK; 8 Lomonosov Moscow State University, Moscow, Russia; 9 Shenzhen MSU-BIT University, Shenzhen, China; 10 School of Geography, South China Normal University, Guangzhou, China; 11 Penza State University, Penza, Russia; 12 Department of Earth Sciences, University of Toronto, Toronto, Canada; 13 Department of Geology, Babes-Bolyai University, Cluj-Napoca, Romania; 14 Goethe University, Frankfurt, Germany; 15 STAR-UBB Institute, Babeş-Bolyai University, Cluj-Napoca, Romania; 16 Department of Geography, Geotop Research Center and Interuniversity Research Group in Limnology, Université du Québec à Montréal, Montréal, Canada; 17 Florence Bascom Geoscience Center, U.S. Geological Survey, Reston, USA; 18 School of Science and the Environment, Manchester Metropolitan University, Manchester, UK; 19 Department of Geological Sciences, University of Alaska, Anchorage, USA; 20 Climate Change Ecology Research Unit, Adam Mickiewicz University, Poznań, Poland; 21 A.N. Severtsov Institute of Ecology and Evolution, Russian Academy of Sciences, Moscow, Russia; 22 Environment, University of York, Heslington, York, UK; 23 Interuniversity Research Group in Limnology, Department of Geography, Université de Montréal, Montréal, Canada; 24 School of Earth Sciences, Zhejiang University, Hangzhou, China; 25 Department of Renewable Resources, University of Alberta, Edmonton, Canada; 26 School of Geography, University of Leeds, Leeds, UK; 27 Past Landscape Dynamic Laboratory, Institute of Geography and Spatial Organization, Polish Academy of Sciences, Warsaw, Poland; 28 Alfred Wegener Institute, Helmholtz Center for Polar and Marine Research, Potsdam, Germany; 29 Institute of Geography, Technical University Dresden, Dresden, Germany; 30 National Park Research Center, Sanming University, Sanming, China; 31 Institute of Geographic Sciences and Natural Resources Research, Chinese Academy of Sciences, Beijing, China

**Keywords:** Climate-change ecology, Wetlands ecology, Palaeoecology

## Abstract

High-latitude peatlands are changing rapidly in response to climate change, including permafrost thaw. Here, we reconstruct hydrological conditions since the seventeenth century using testate amoeba data from 103 high-latitude peat archives. We show that 54% of the peatlands have been drying and 32% have been wetting over this period, illustrating the complex ecohydrological dynamics of high latitude peatlands and their highly uncertain responses to a warming climate.

## Abstract (web summary)

A recent synthesis study found 54% of the high-latitude peatlands have been drying and 32% have been wetting over the past centuries, illustrating their complex ecohydrological dynamics and highly uncertain responses to a warming climate.

## Introduction

The majority of peatlands are located in high latitudes^1^ and store *ca*. one third of the global soil carbon (C)^2^. The balance between photosynthesis-driven carbon dioxide (CO_2_) sequestration and decomposition-driven CO_2_ and methane (CH_4_) emissions determines the peatland net C budget and subsequently the overall climate feedback. Peatland water-table position is a decisive factor in this balance. Peatland water-table drawdown results in a net increase of greenhouse gas emissions (mainly CO_2_) and consequently a positive (warming) net climate effect^3^. Conversely, water-saturated peatlands are large CH_4_ sources, as evidenced in permafrost peatlands under a warming climate^4^. Accordingly, understanding the current peatland moisture status, past successional pathways and their link to climate is important. These dynamics can be studied using testate amoebae, which are hydrology-sensitive biological proxies. Testate amoeba data were recently used to document a widespread drying of central-European peatlands^5^. However, there was no efforts made to compile data from boreal, subarctic and permafrost peatlands. It is therefore uncertain whether drying is a more geographically extensive phenomenon that extends to the subarctic-arctic ecosystems. Our data compilation aims to resolve how the amplified warming in high latitudes is reflected in peatland moisture conditions. More than 50% of pan-Arctic peatlands contain permafrost^6^, which is thawing in many places^7–9^—despite the insulative effects of the overlying peat stratum, which slows the rate of thawing^10^. The subsequent development of wetter or drier conditions depends on local topographic controls, drainage networks, regional precipitation patterns (geographical and seasonal distribution, frequency, and amount), evapotranspiration, and ice richness of the permafrost^7,11–13^.

Predicting peatland moisture balance using climatic parameters only may not be reasonable due to the complex interactions with precipitation-evapotranspiration, runoff, permafrost dynamics, and autogenic processes^11,14,15^. The hydrologically sensitive testate amoebae, archived in peats, provide an opportunity to study the contemporary moisture conditions over the period when peat they occupied was growing at the surface^5^. Transfer functions based on local/regional modern training sets of well-established testate amoeba data and supplemented by robust recent chronologies enabled us to carry out water-table depth (WTD) reconstructions for high latitudes, focusing on the past four centuries. This focused period encompasses the post-Little Ice Age (LIA) warming and the more recent global warming, for both the timing has varied regionally.

**Fig. 1:**
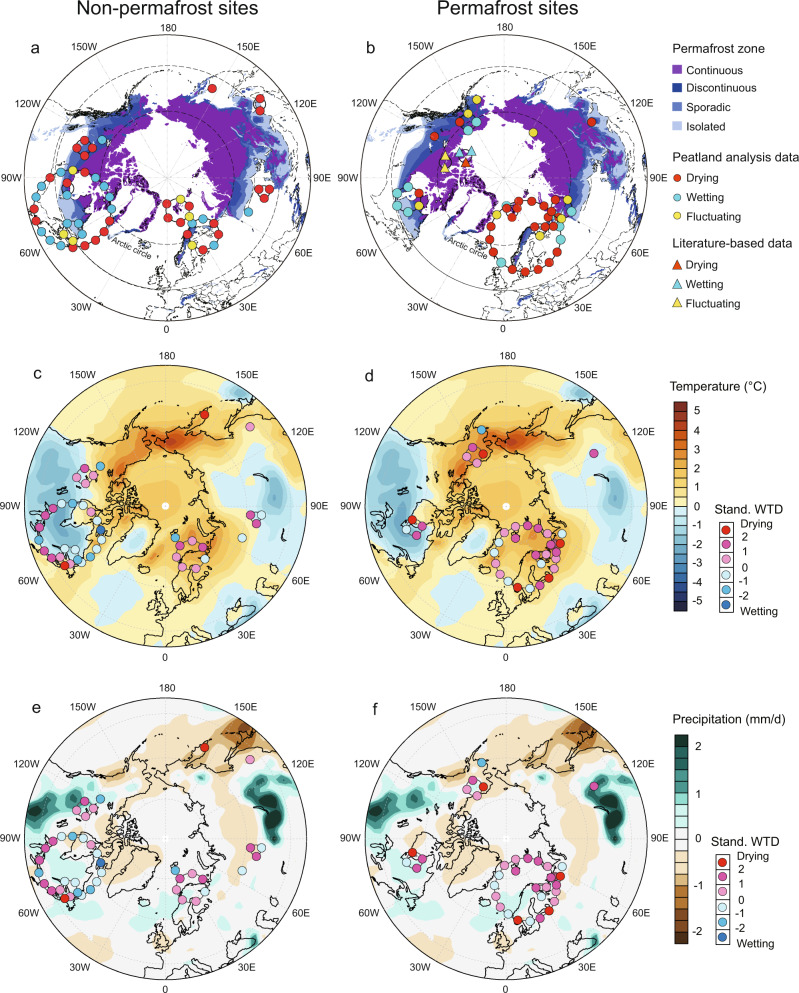
Study sites and their hydrological responses. Non-permafrost (**a**, **c**, **e**) and permafrost (**b**, **d**, **f**) sites are plotted separately. **a**, **b** Reconstructed hydrological response of 98 records since 1600 CE on the map of northern permafrost zones^35^. Literature-based five records are indicated by triangle symbol. **c**, **d** Reconstructed standardised water-table depths (WTDs) on the map of summer (June-July-August) temperature anomaly (°C). **e**, **f** Reconstructed WTDs on the map of summer precipitation anomaly (mm/day). WTDs, temperature and precipitation data presented in **c**–**f** are values calculated using (1963 to 2012 CE average) minus (1851 to 1900 CE average). 76 records that have data points for these two periods are shown. Temperature and precipitation data (*ca*. 2° latitude x 2° longitude grids) are from NOAA-ESRL and CIRES twentieth century Reanalysis (V2c)^33^. The coordinates of the study sites on the maps are adjusted using a ‘ring’ Points Displacement to avoid overlapping; the actual coordinates are in the center of each ring and can be found in the Supplementary Fig. 1 and Datasheet 1.

**Fig. 2:**
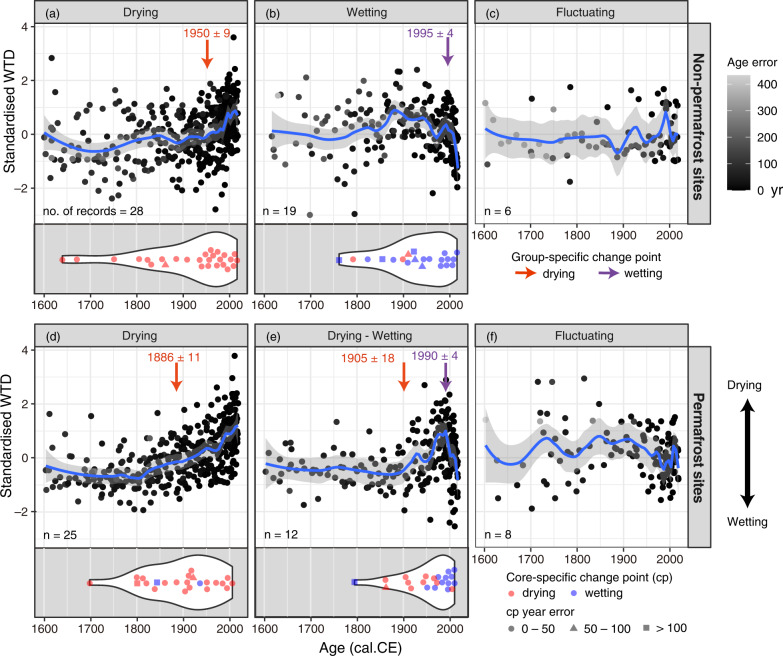
Standardised water-table depth (WTD) data over the past centuries compiled into different hydrological response groups. Non-permafrost (**a**–**c**) and permafrost (**d**–**f**) sites are plotted separately. A LOESS model is shown as a blue line, with the gray shading indicating the 95% confidence interval. The age error scale indicates the chronological precision of each data point (determined through Bayesian age modelling). Inside-plot arrows indicate the estimated hydrological change points (Est. ± SE) of the compiled data in each group. Violin plots show the estimated hydrological change points of individual records included in each corresponding group (with few exceptions addressed in Supplementary Datasheet 1). The age error of the estimated change points presented in the violin plots was indicated using different shapes.

In this study, we compiled 103 peatland testate amoeba records across the northern high latitudes, including sites inside and slightly outside the northern permafrost distribution region, to examine changes in peatland surface wetness during the last four centuries (Fig. 1 and Supplementary Fig. 1). We used a comprehensive spatial collection of available records from different northern peatland types and permafrost distribution zones (bog, fen, palsa, peat plateau; continuous, discontinuous, sporadic, isolated permafrost zones), with approximately half of the sites representing permafrost peatlands. Most of the site are nutrient-poor systems (Supplementary Datasheet 1). Even though different peatland types have distinctly different testate amoeba communities, hydrology remains the most important environmental control. When applying applicable transfer functions, they provide valid hydrological reconstructions^16^. WTD reconstruction was conducted for each record using the best available modern training sets and transfer functions for testate amoebae, and the results were standardized to enable spatial comparison^5^. Those records that showed a similar hydrological shift over the past four centuries were grouped together, regardless their geographical location. Statistically significant change points in hydrological conditions (from wet to dry or vice versa) were detected for individual records and for the compiled groups to capture the timing of major hydrological change (Fig. 2). In order to directly link the observed hydrological successions to climate variables, we compared the standardized WTD patterns to summer temperature and precipitation anomalies between modern and pre-industrial periods (1963 to 2012 CE average minus 1851 to 1900 CE average; Fig. 1c–f) followed the approach used in Swindles et al. (2019)^5^. This procedure allows us to include as many records as possible regardless the low temporal resolution of some of the records. By doing this also the data provide the best possible spatial perspective.

## Results and discussion

### Hydrological changes in high-latitude peatlands

We observed three hydrological pathways, i.e., drying, wetting, and fluctuating, for both peatland clusters, non-permafrost and permafrost peatlands (Fig. 2). Approximately 54% of the studied peatlands have shifted towards drier surface conditions since 1800 CE and more intensively since 1900 CE (Fig. 2a, d), which is in line with the post-LIA warming. The overall change point to drier conditions was dated to *ca*. 1950 CE for non-permafrost sites and *ca*. 1890 CE for the permafrost compiled group. Approximately 32% of the studied peatlands have shifted towards wetter conditions (Fig. 2b, e). The overall shifting point to wetter conditions was dated to *ca*. 1995 CE for non-permafrost peatlands and to *ca*. 1990 CE for permafrost peatlands. Wetting has been especially intensive since 1900 CE for non-permafrost peatlands and since 1950 CE for permafrost peatlands. Interestingly, the data showed that in permafrost peatlands a significant dry shift always preceded a wet shift (Fig. 2e). Approximately 14% of the studied peatlands indicated no clear trend, with fluctuating hydrological conditions (Fig. 2c, f).

Non-permafrost peatlands generally showed spatially extensive drying across the northern high latitudes, apart from northeastern Canada, where a wetting trend was more frequently observed. Permafrost peatlands, however, were more variable, with some drying, some wetting, and no overall coherent regional pattern was visible (Fig.1a, b). It should be noted that peatlands synthesized here have undergone little or no direct human impact, i.e., their surface hydrology was not significantly affected by human disturbances such as drainage, when compared to, for example, central European peatlands discussed in Swindles et al. (2019)^5^. This implies that climate and/or local topographical forcing are the predominant hydrological drivers in this study. The dataset is to some extent biased as there are more non-permafrost records from northeastern Canada but more permafrost records from northern Sweden and this might result in regional overestimation to either wetting or drying trends. Nevertheless, the pattern of diverse timing of the hydrological shifts between the individual coring points (Fig. 2) indicates the variability in sensitivity of different regions/peatlands to climate changes.

### Potential links to climate change and permafrost dynamics

The comparison of the reconstructed water table and climate data suggests that climate, especially summer temperature, has played an important role in shaping the peatland water table (Fig. 1c–f). The pattern detected here for non-permafrost peatlands, an extensive drying, is comparable to that observed for central European peatlands^5^. In addition to direct climate forcing, a recent acceleration of peat accumulation might partly explain the drying trend by disconnecting the peatland surface from the water table^17^. However, for northeastern Canada a wetting trend has been observed more often, possibly regulated by the regional climate that shows clearly less warming in the focused period compared to other regions (Fig. 1c, d).

Permafrost initiation in the past caused a peat surface uplift and is probably detected as a dry phase (Fig. 2d, e). Post-LIA warming-induced increase in evapotranspiration may have strengthened the surface drying which originally resulted from surface-uplift and probably mitigated the gradual wetting related to permafrost thawing^11^. The level of warming has varied among the regions. In some areas such as northeastern Canada temperature has increased less and, when combined with higher precipitation or higher effective moisture level, may have caused surface wetting in permafrost peatlands. This is a direct climate forcing rather than permafrost thawing, which is a consequence of climate warming, i.e., more indirect climate forcing. To date, it is yet challenging to estimate any one tipping point of warming that might trigger permafrost thawing, as the local conditions vary from bottom ground soil conditions to hydrology and vegetation. The consequent wetting or drying depends on evapotranspiration and ice richness etc., which further challenges the prediction of hydrological conditions of permafrost peatlands.

The divergent three moisture patterns may occur in the same region and even in the same peatland, especially if the permafrost is present. This complex response pattern is well supported by the records from the Abisko region, Sweden, where replicated sampling was carried out, and captured different successional stages of local permafrost peatlands^7,18^. In contrast to permafrost peatlands, non-permafrost peatlands are more likely to experience a more consistent ecosystem response pattern^19^ as supported by the replicated records suggesting the same pattern happening simultaneously in several regions (Fig. 1a). The fluctuating pattern of many records reported here suggests that the past and recent climate has not yet caused a state change in hydrological conditions.

### Insights into carbon dynamics and future perspective

Generally, our results suggest that the recent climate warming has caused hydrological shifts in most high-latitude peatlands, highlighting its pronounced effect on shaping peatland moisture balance, and further on driving peatland C balance. It has been reported that a 1-cm water-table drawdown would increase 3.3–5.0 mg CO_2_ m^−2^ h^−1^ and decrease 2.2–3.6 mg CO_2_-eq m^−2^ h^−1^ (CH_4_) to the atmosphere, and the average sensitivity of CO_2_ and CH_4_ combined was 0.8–2.3 mg CO_2_-eq m^−2^ h^−1^ cm^−1^ according to a global scale analysis, including sites from high-latitudes^3^. However, it should be noted that the sensitivity of greenhouse gas fluxes to the magnitude of hydrological changes might vary among different regions and peatland types. It appears that most pan-Arctic peatlands are undergoing a drying trend, that may lead to a decreased C sink capacity^3,19^, if not compensated by increased C uptake from the atmosphere^20^.

It is very likely that over the 21st century warming in high latitudes will continue to be more pronounced than the global average^21^. Precipitation is projected to increase, albeit with large regional variability. Also, extreme events with heavy rainfall and drought are becoming more frequent and intense^22^. It is estimated that about 20% of permafrost zone is experiencing accelerated and abrupt permafrost thaw that is likely causing wetting conditions^4^, while gradual permafrost thaw has been observed across the circumpolar regions^23^. Both an increase in precipitation and permafrost thaw might mitigate the drying pressure caused by warming and increased evapotranspiration. However, abrupt permafrost thaw in peatlands can result in a rapid (over years to decades) loss of C from the formerly frozen permafrost peat, causing these peatlands to be a net source of C to the atmosphere before post-thaw accumulation returns them to a net sink (centuries to millennia)^12,13^. The future C sink and source function of peatlands is a key element in contributing to climate change, but the observed divergent pathways of peatland hydrological successions further challenge the projections of high-latitude peatland C sink and source dynamics. Conversely, it clearly highlights the importance of climate forcing in peatland succession scenarios. Our study reveals that the response of high-latitude peatlands to changing climate conditions is complex. We detect variable ecohydrological trajectories, and in the future, these will determine the C sink capacity of northern peatlands. The observed patterns inevitably create challenges for the climate change modelling community. How to capture the highly heterogenic successional pathways of northern peatlands needs to be a key research focus.

## Methods

### Study sites

In total, 103 sites with suitable data were identified and compiled (Supplementary Datasheet 1). Of 103, 98 sites were included in the current data analyses. These analyses were supplemented by five previously published records (without applicable transfer functions) presented in the Fig. 1b. The presence of permafrost at the sampling point indicates the conditions at the time when the samples were collected.

### Chronology

Age-depth models were constructed for each record using chronological data including ^14^C, ^210^Pb, and other age-equivalent stratigraphic markers such as ^137^Cs and tephra dates. Bayesian age models were generated for each record to achieve good accuracy and quantification of age errors using the ‘rplum’ package^24^ in R version 3.6.1^25^ (Supplementary Fig. 2). The age of the midpoint depth of the analysed sample was derived. Hereafter, all references to ages or years refer to the maximum probability age at a given depth, as determined from the age model.

### Water-table depth reconstruction

Only records with the dominate testate amoeba taxa presented in the transfer functions and a minimum total count of *ca*. 50 reached were include in water-table depth (WTD) reconstructions. Taxonomic harmonisation was necessary in order to apply the transfer functions based on European, North American, Asian and Holarctic training sites^26–28^ (Supplementary Datasheet 1). The reconstructions were carried out in R version 3.6.1^25^ using location-specific transfer functions defined by geographic location of the study site, and different datasets with and without weak silicic idiosomic tests^5,29^. Based on the outputs (Supplementary Figs. 3 and 4), the Holarctic transfer function that contains the most abundant data was selected for European and North American sites, and Asian transfer function for Asian sites, unless otherwise specified in Supplementary Datasheet 1. The reconstructions run on the data without the weak silicic idiosomic tests were used for subsequent analyses.

### Hydrological response analysis

The 98 peat records were divided into six groups based on the presence of on-site permafrost and the recent hydrological response trend. A LOESS smoothing function^30^ with a span value (degree of smoothing) setting of 0.2 was applied for the compiled six groups. Change-point analysis was performed on the compiled groups and individual records to detect the overall and local breakpoints of the linear trend of hydrological conditions over time using the package ‘Segmented’^31^ in R version 3.6.1^25^. The temporal span used in this analysis was 1600 CE to present. In cases that no segmented linear breakpoints were estimated, detections of changes in mean and variance over time were carried out using the ‘At Most One Change’ and ‘Pruned Exact Linear Time’ methods using the package ‘Changepoint’^32^ in R version 3.6.1^25^ (Supplementary Datasheet 1).

### Climatic data

Temperature and precipitation data representing the period 1851–2012 were from the NOAA-ESRL and CIRES Twentieth Century Reanalysis (V2c) dataset^33^ and downloaded from the Earth System Grid Federation^34^. These data are with monthly temporal resolution and spatial resolution of *ca*. 2° latitude x 2° longitude. The temperature and precipitation data were split into two 50-year time periods of 1851–1900 and 1963–2012 respectively for the summer months of June, July and August. The difference between these two periods was then calculated and used for producing the maps.

## Supplementary information

Supplementary Information

Description of Additional Supplementary Files

Supplementary Data 1

## Data Availability

The peat record data that support the findings of this study can be accessed at the WDC for Geophysics, Beijing (10.12197/2022GA021).
