# Supplementary material for: Recent climate change has driven divergent hydrological shifts in high-latitude peatlands
Source: Nat Commun. 2022 Aug 24;13:4959. doi: 10.1038/s41467-022-32711-4 (PMC9402595; doi:10.1038/s41467-022-32711-4)
Supplement: Supplementary file 1 — Supplementary Information [file 41467_2022_32711_MOESM1_ESM.pdf]

Supplementary information

Recent climate change has driven divergent hydrological shifts in high-latitude peatlands

Supplementary Figure 1

Study site information. a) Locations of the study sites presented on the map of northern permafrost zones. The yellow points (n = 98) are sites included in the data analyses; the orange points (n = 5) are sites from the literature. b) Distribution of study sites in each permafrost zone with number of sites that have *in situ* permafrost present. c) Violin plots depicting magnitude and distribution density of collected samples over time period 1600 CE to present in each permafrost zone.

Northern permafrost distribution information can be found in Brown, J., Hinkel, K.M., Nelson, F.E. The circumpolar active layer monitoring (CALM) program: Research desighs and initial results. *Polar Geogr*. **24**, 165-258, (2000).

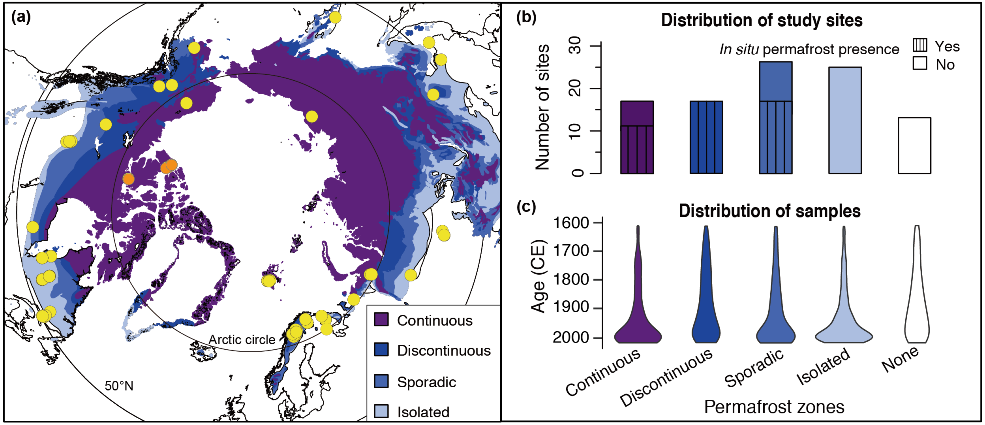

(a)
Arctic circle
50°N
Continuous
Discontinuous
Sporadic
Isolated
(b)
Distribution of study sites
In situ permafrost presence
Yes
No
Number of sites
0
10
20
30
(c)
Distribution of samples
Age (CE)
1600
1700
1800
1900
2000
Continuous
Discontinuous
Sporadic
Isolated
None
Permafrost zones

Supplementary Figure 2

Bayesian age-depth models of the analysed 98 peat records developed using ‘rplum’ package in R. Upper panels depict the MCMC iterations (first panel), the prior (green curves) and posterior (grey curve) distributions for the accumulation rate (second panel) and memory (third panel), the prior (green) and posterior (grey) distribution of the parameters related to the ^{210}Pb part of the model (fourth and fifth panels if available; influx and supported ^{210}Pb). Bottom panel shows the calibrated ^{14}C dates (transparent purple), measured unsupported ^{210}Pb activities (if available; blue), ^{210}Pb dates (when the ^{210}Pb concentration data not available; transparent green; records TFS1 and TFS2), ^{137}Cs and tephra dates (if available; transparent green) and the age-depth model (darker greys indicate more likely calendar ages; grey stippled lines show 95% confidence intervals; red curve shows single 'best' model based on the mean age for each depth).

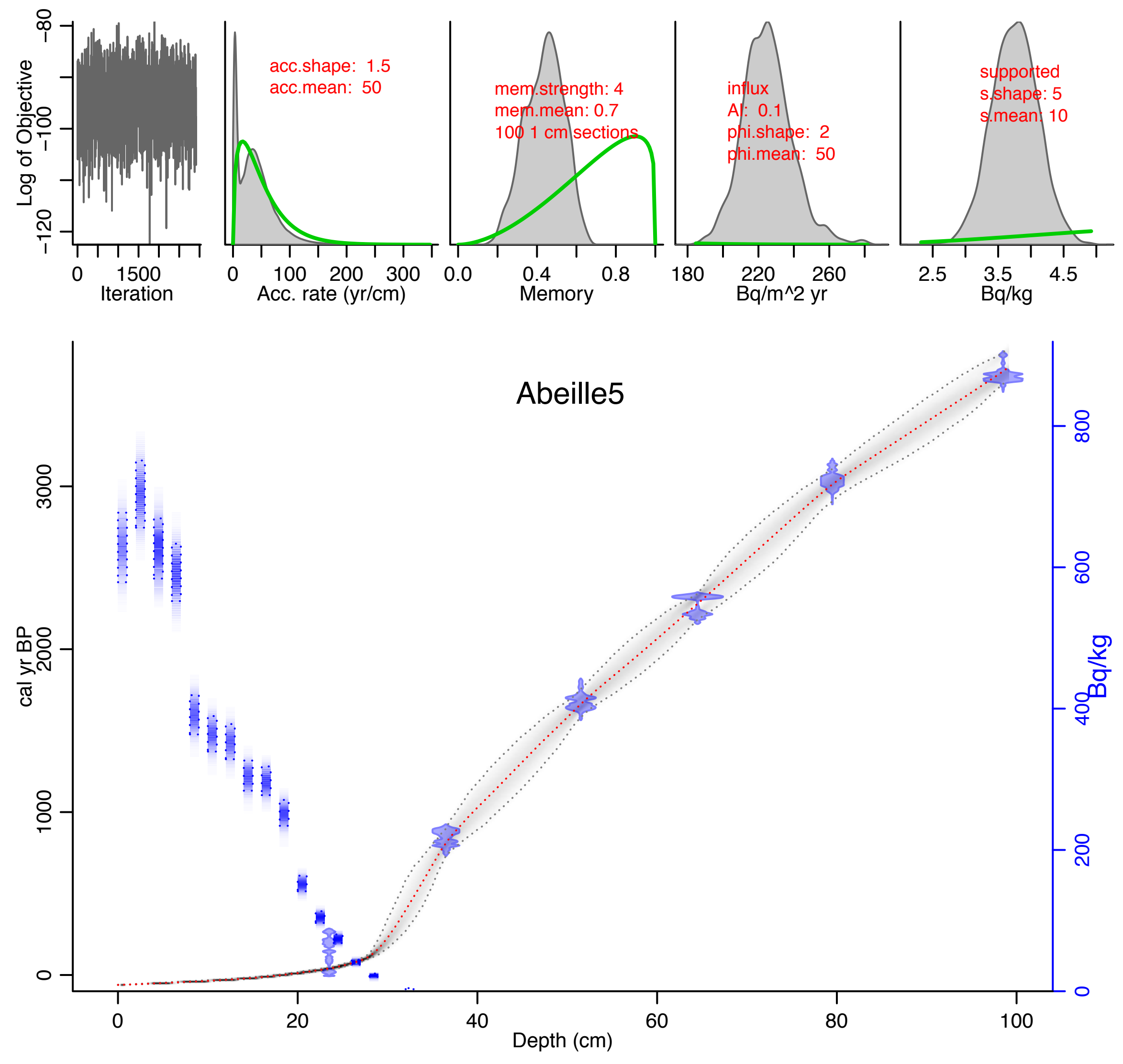

Log of Objective
-80
-100
-120
0
1500
Iteration
acc.shape: 1.5
acc.mean: 50
0
100
200
300
Acc. rate (yr/cm)
mem.strength: 4
mem.mean: 0.7
100 1 cm sections
0.0
0.4
0.8
Memory
influx
Al: 0.1
phi.shape: 2
phi.mean: 50
180
220
260
Bq/m^2 yr
supported
s.shape: 5
s.mean: 10
2.5
3.5
4.5
Bq/kg
Abeille5
cal yr BP
0
1000
2000
3000
Bq/kg
0
200
400
600
800
0
20
40
60
80
100
Depth (cm)

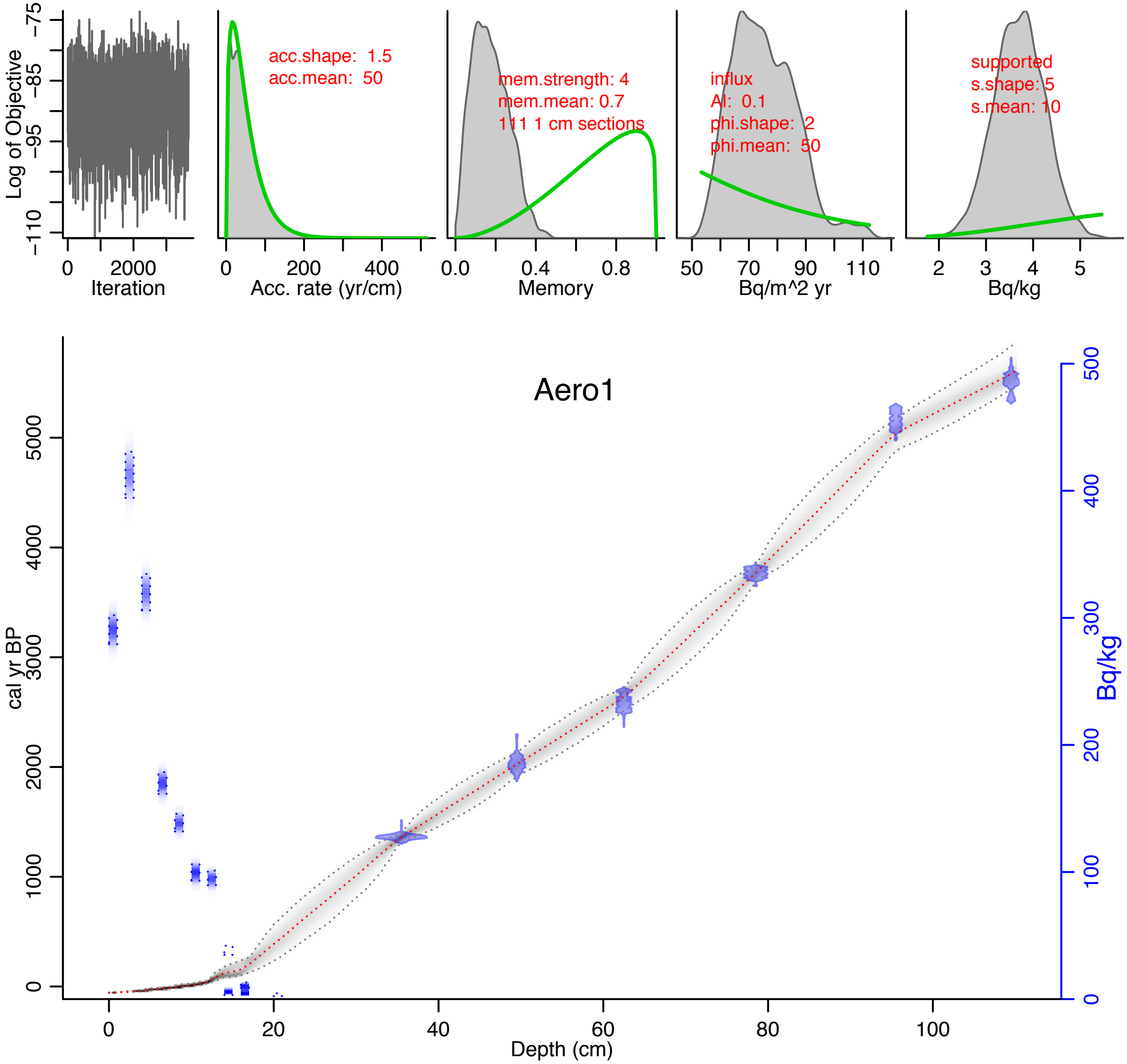

Log of Objective
−75
−85
−95
−110
0
2000
Iteration
acc.shape: 1.5
acc.mean: 50
0
200
400
Acc. rate (yr/cm)
mem.strength: 4
mem.mean: 0.7
111 1 cm sections
0.0
0.4
0.8
Memory
influx
Al: 0.1
phi.shape: 2
phi.mean: 50
50
70
90
110
Bq/m^2 yr
supported
s.shape: 5
s.mean: 10
2
3
4
5
Bq/kg
Aero1
cal yr BP
0
1000
2000
3000
4000
5000
Bq/kg
0
100
200
300
400
500
0
20
40
60
80
100
Depth (cm)

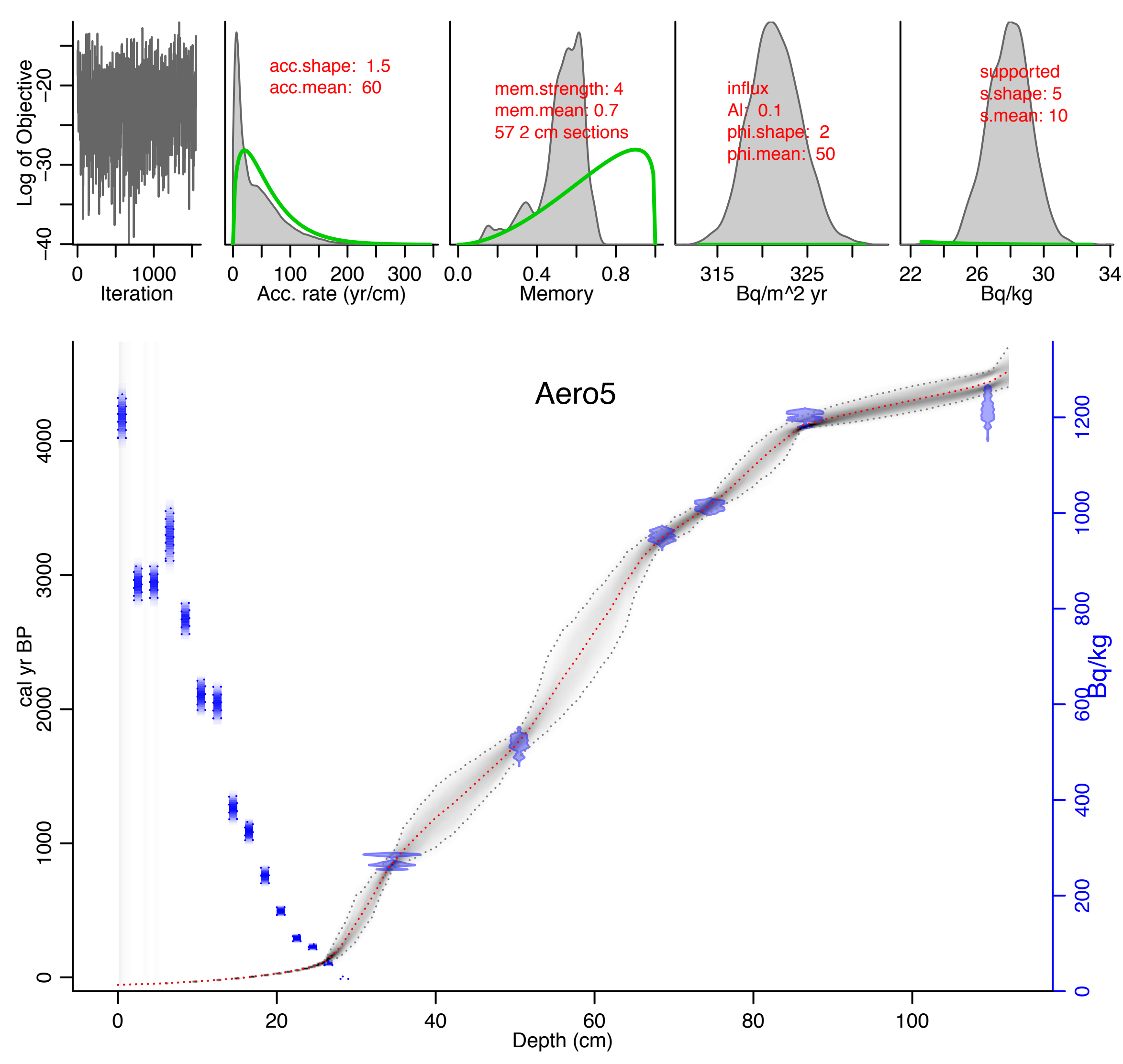

Log of Objective
Iteration
acc.shape: 1.5
acc.mean: 60
Acc. rate (yr/cm)
mem.strength: 4
mem.mean: 0.7
57 2 cm sections
Memory
influx
Al: 0.1
phi.shape: 2
phi.mean: 50
Bq/m^2 yr
supported
s.shape: 5
s.mean: 10
Bq/kg
Aero5
cal yr BP
Depth (cm)
Bq/kg

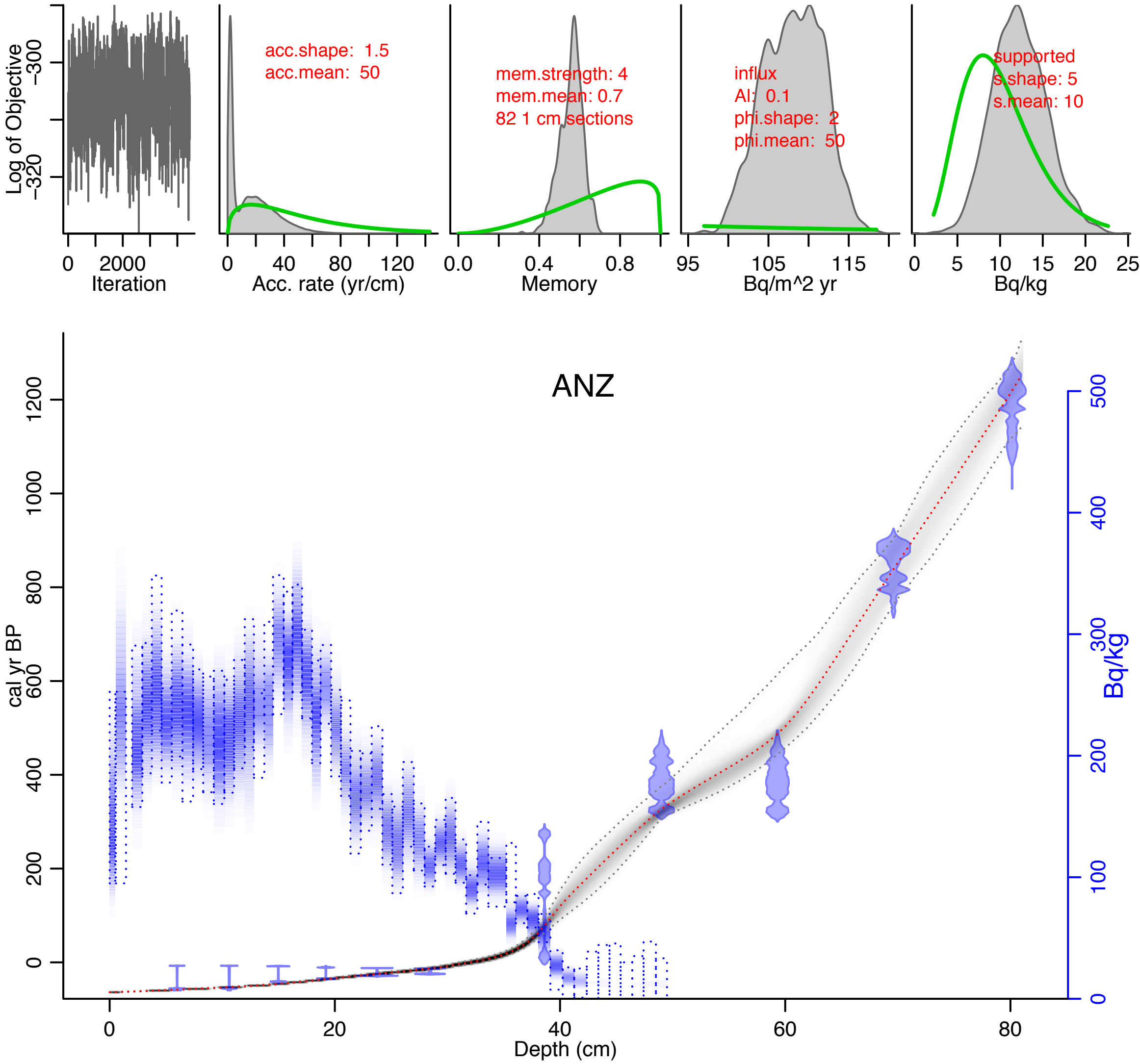

Log of Objective
−300
−320
0
2000
Iteration
acc.shape: 1.5
acc.mean: 50
0
40
80
120
Acc. rate (yr/cm)
mem.strength: 4
mem.mean: 0.7
82 1 cm sections
0.0
0.4
0.8
Memory
influx
Al: 0.1
phi.shape: 2
phi.mean: 50
95
105
115
Bq/m^2 yr
supported
s.shape: 5
s.mean: 10
0
5
10
15
20
25
Bq/kg
ANZ
cal yr BP
0
200
400
600
800
1000
1200
Bq/kg
0
100
200
300
400
500
0
20
40
60
80
Depth (cm)

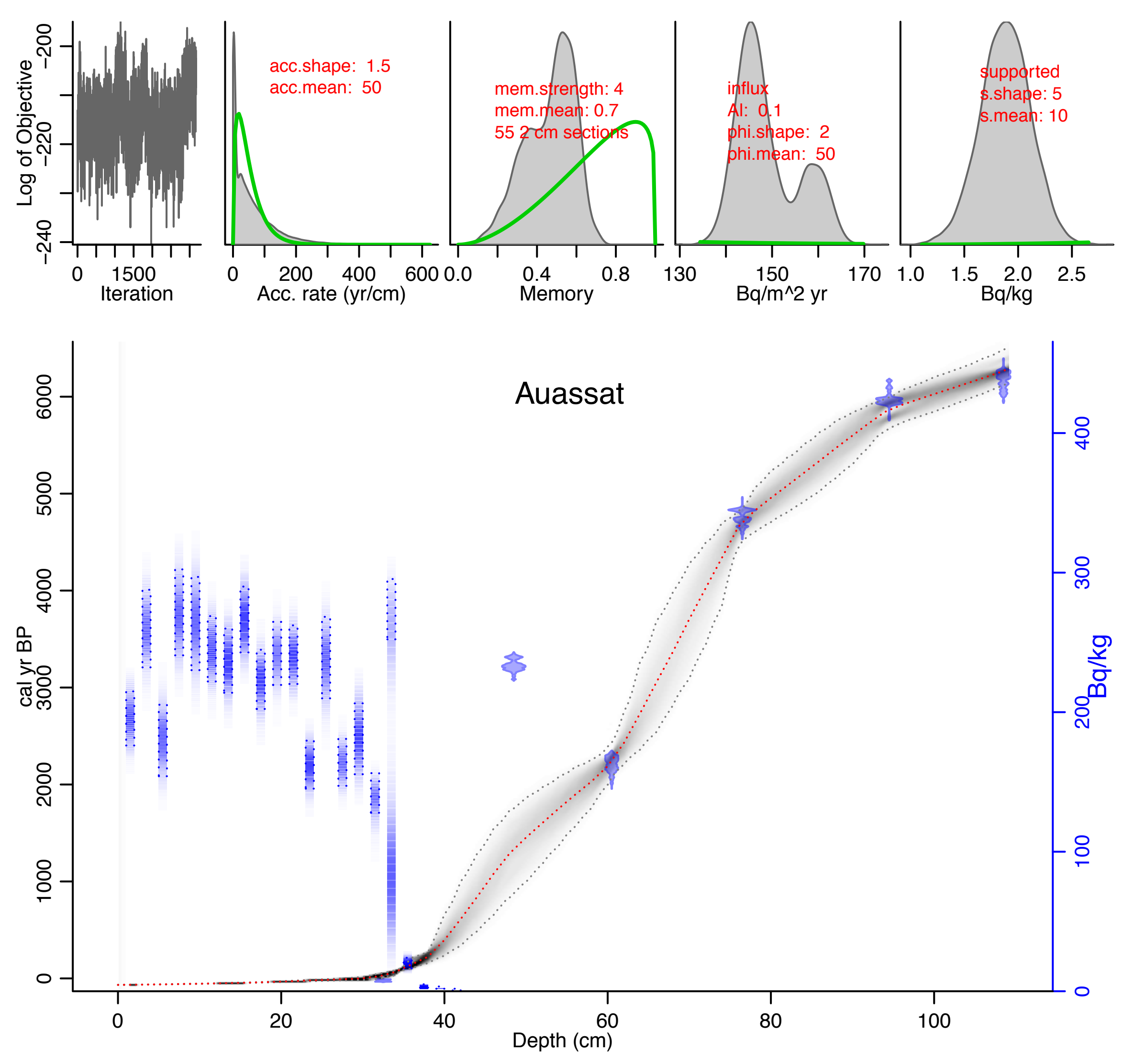
Log of Objective
Iteration
acc.shape: 1.5
acc.mean: 50
Acc. rate (yr/cm)
mem.strength: 4
mem.mean: 0.7
55 2 cm sections
Memory
influx
Al: 0.1
phi.shape: 2
phi.mean: 50
Bq/m^2 yr
supported
s.shape: 5
s.mean: 10
Bq/kg
Auassat
cal yr BP
Bq/kg
Depth (cm)

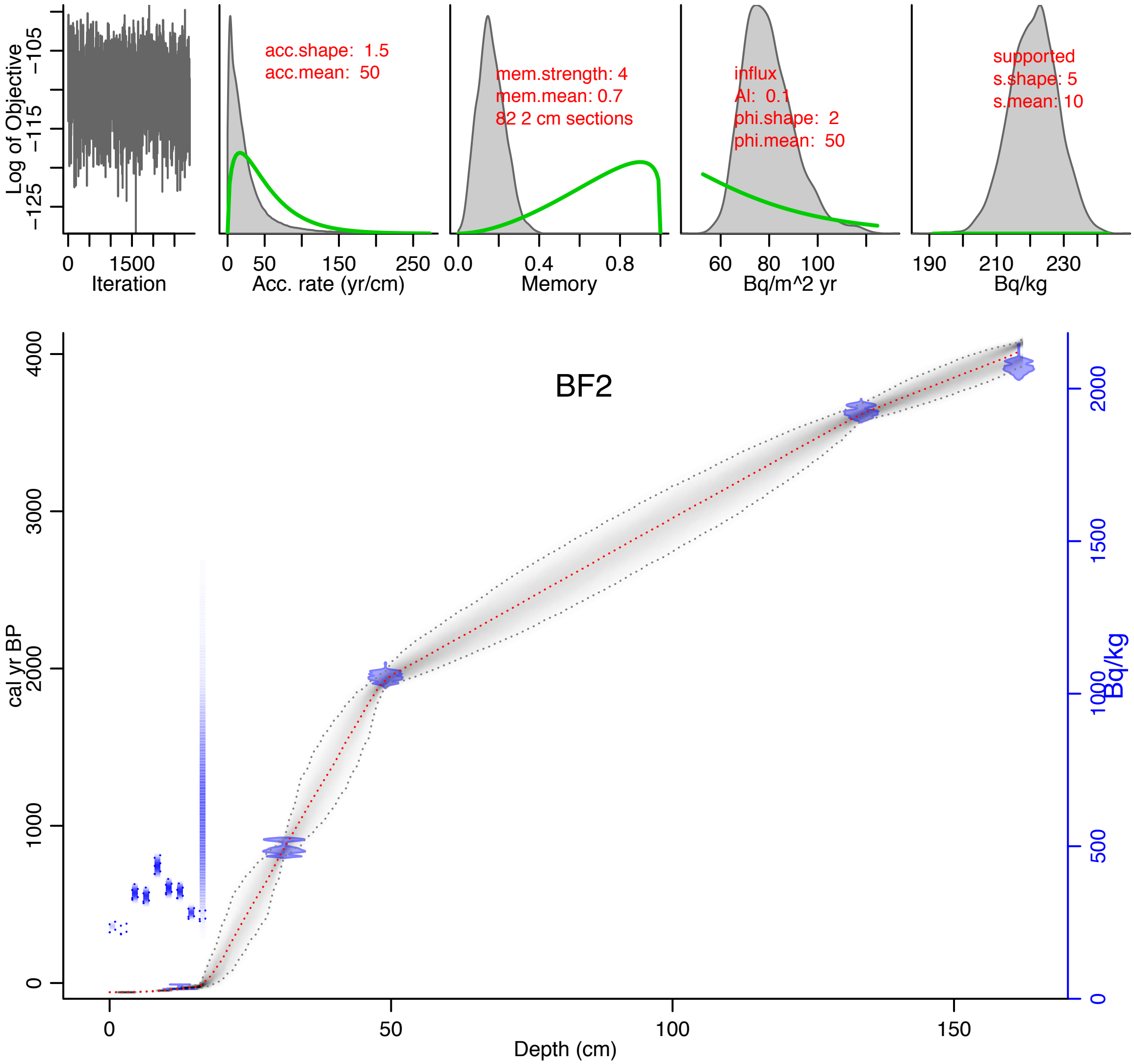

Log of Objective
−105
−115
−125
0
1500
Iteration
acc.shape: 1.5
acc.mean: 50
0
50
150
250
Acc. rate (yr/cm)
mem.strength: 4
mem.mean: 0.7
82 2 cm sections
0.0
0.4
0.8
Memory
influx
Al: 0.1
phi.shape: 2
phi.mean: 50
60
80
100
Bq/m^2 yr
supported
s.shape: 5
s.mean: 10
190
210
230
Bq/kg
BF2
cal yr BP
0
1000
2000
3000
4000
Depth (cm)
0
50
100
150
Bq/kg
0
500
1000
1500
2000

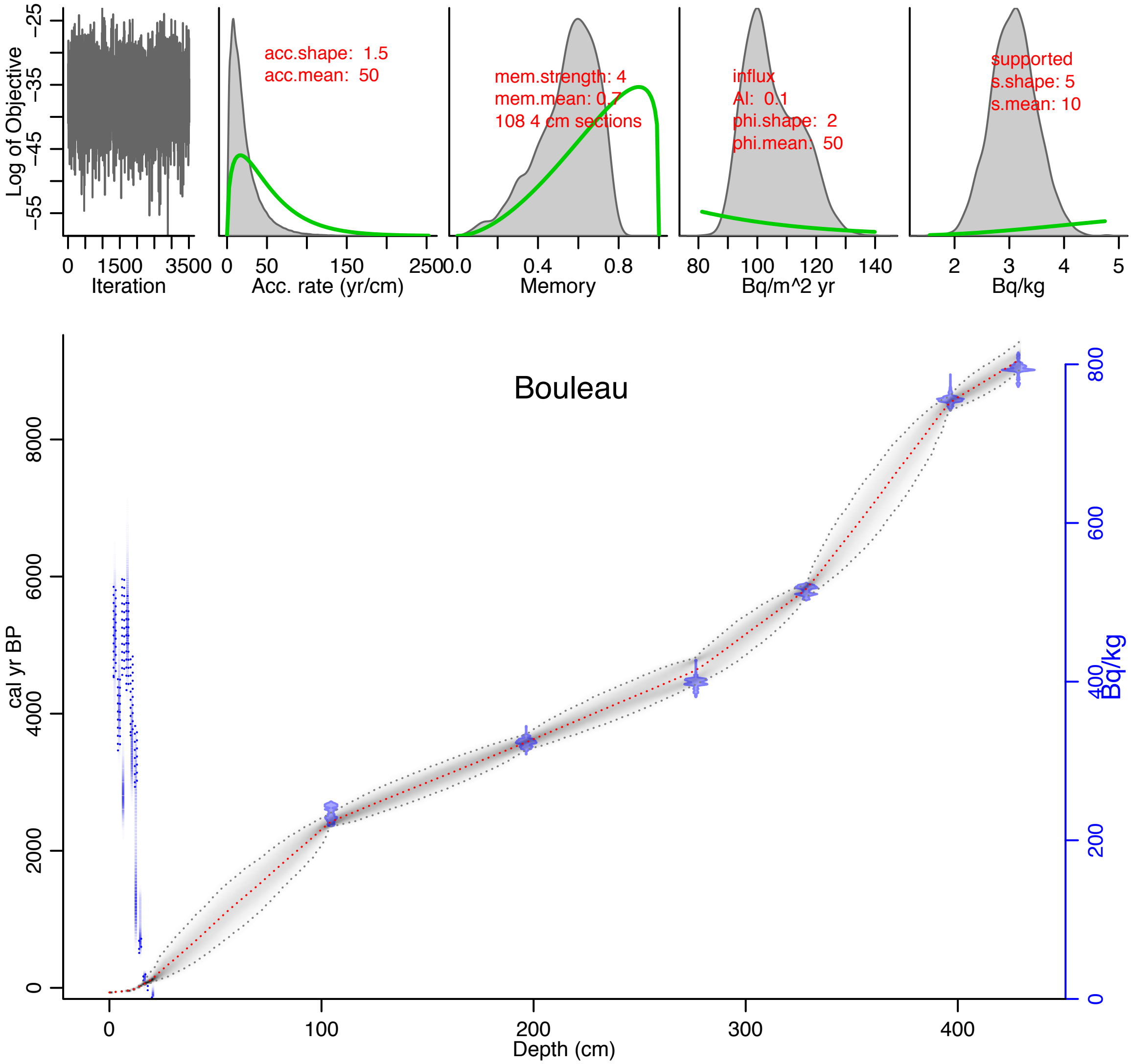

Log of Objective
Iteration
acc.shape: 1.5
acc.mean: 50
Acc. rate (yr/cm)
mem.strength: 4
mem.mean: 0.7
108 4 cm sections
Memory
influx
Al: 0.1
phi.shape: 2
phi.mean: 50
Bq/m^2 yr
supported
s.shape: 5
s.mean: 10
Bq/kg
Bouleau
cal yr BP
Depth (cm)
Bq/kg

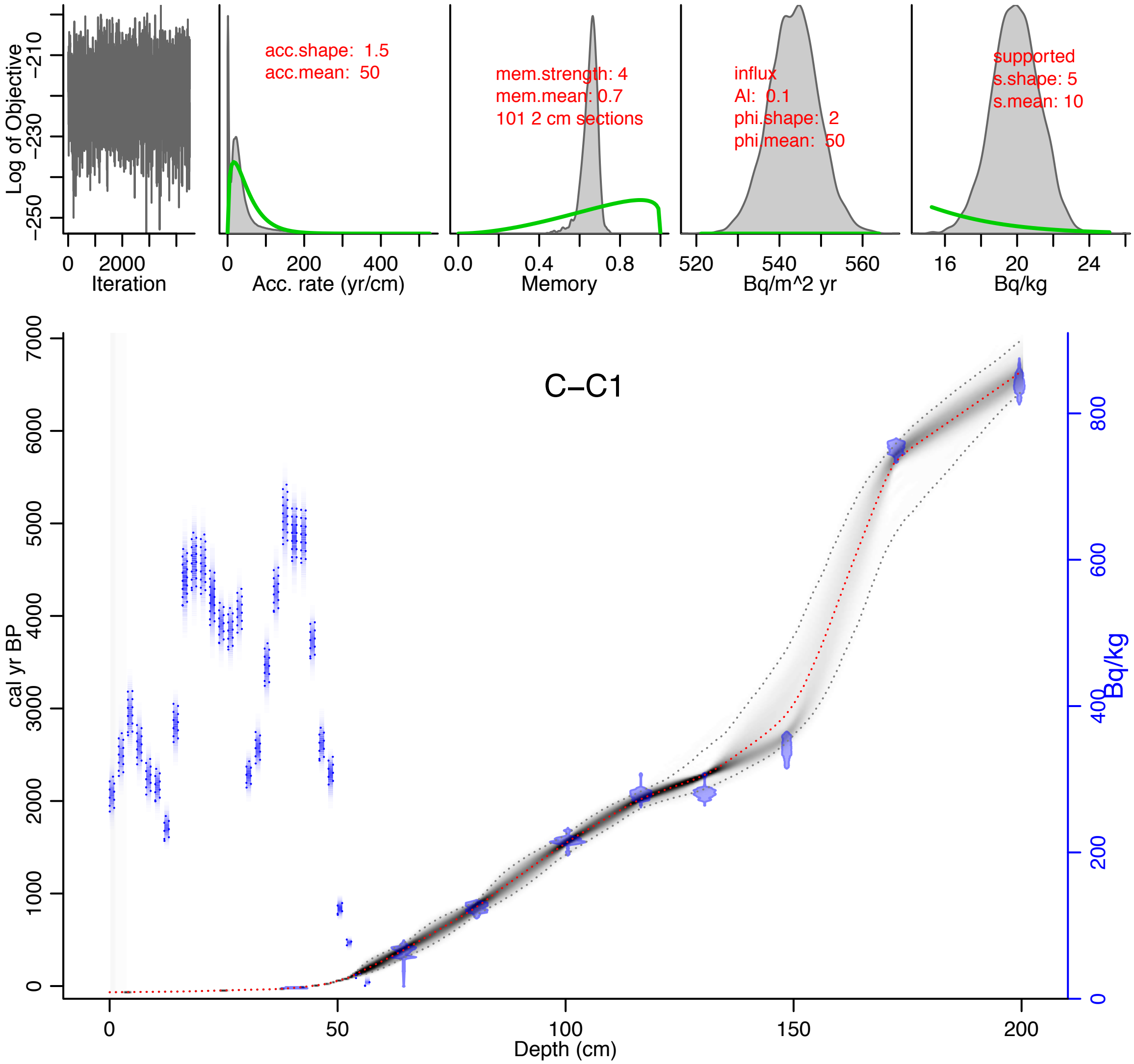
Log of Objective
−210
−230
−250
0
2000
Iteration
acc.shape: 1.5
acc.mean: 50
0
200
400
Acc. rate (yr/cm)
mem.strength: 4
mem.mean: 0.7
101 2 cm sections
0.0
0.4
0.8
Memory
influx
Al: 0.1
phi.shape: 2
phi.mean: 50
520
540
560
Bq/m^2 yr
supported
s.shape: 5
s.mean: 10
16
20
24
Bq/kg
C−C1
cal yr BP
0
1000
2000
3000
4000
5000
6000
7000
Depth (cm)
0
50
100
150
200
Bq/kg
0
200
400
600
800

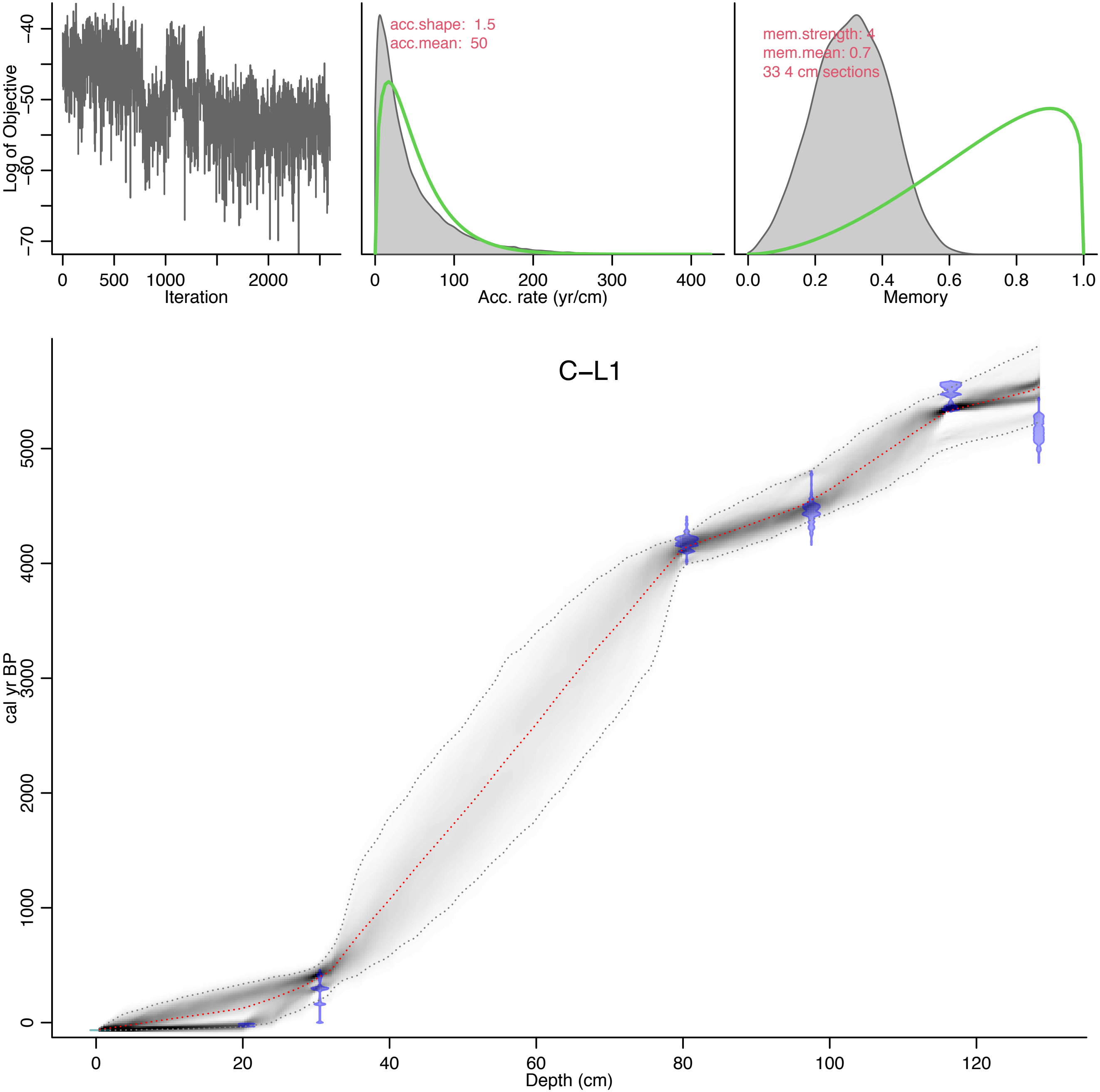

Log of Objective
Iteration
acc.shape: 1.5
acc.mean: 50
Acc. rate (yr/cm)
mem.strength: 4
mem.mean: 0.7
33 4 cm sections
Memory
C−L1
cal yr BP
Depth (cm)

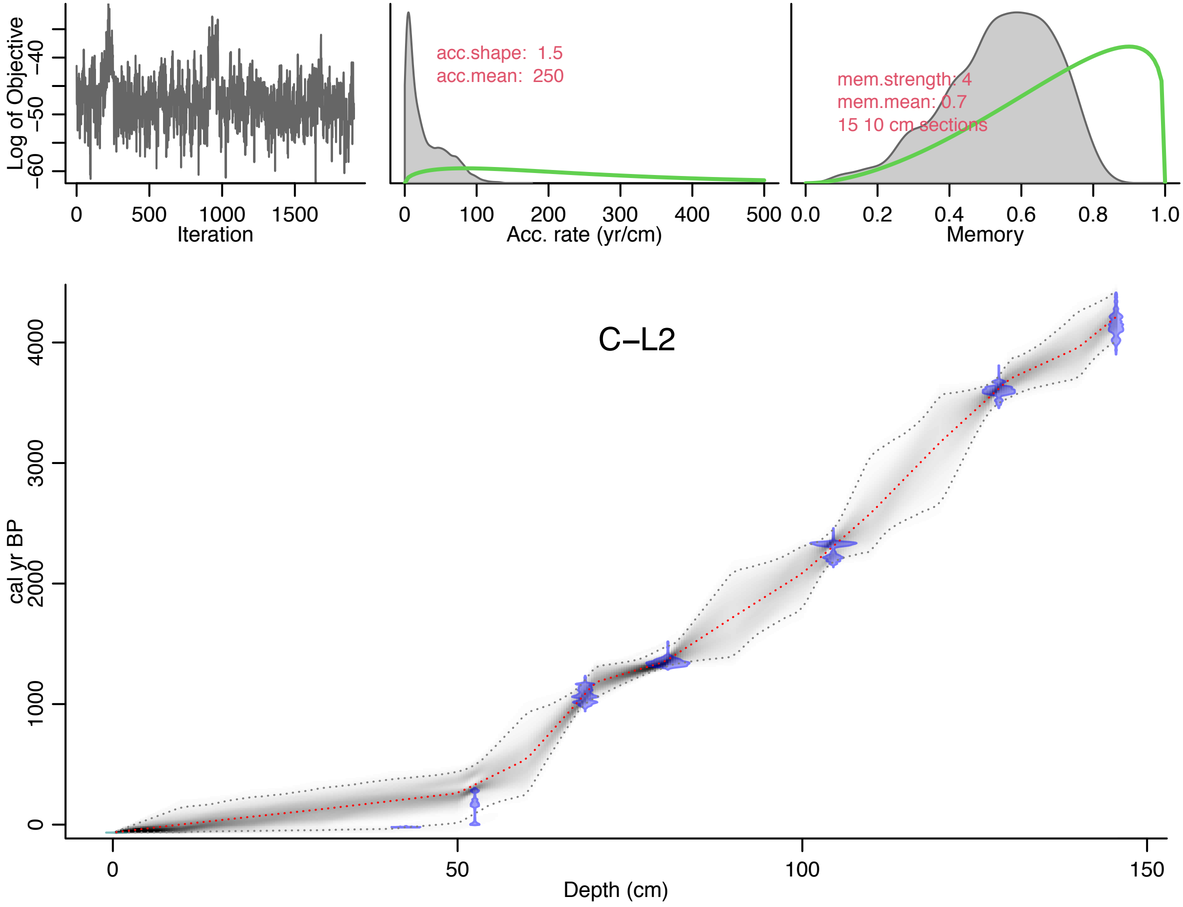
Log of Objective
Iteration
acc.shape: 1.5
acc.mean: 250
Acc. rate (yr/cm)
mem.strength: 4
mem.mean: 0.7
15 10 cm sections
Memory
C–L2
cal yr BP
Depth (cm)

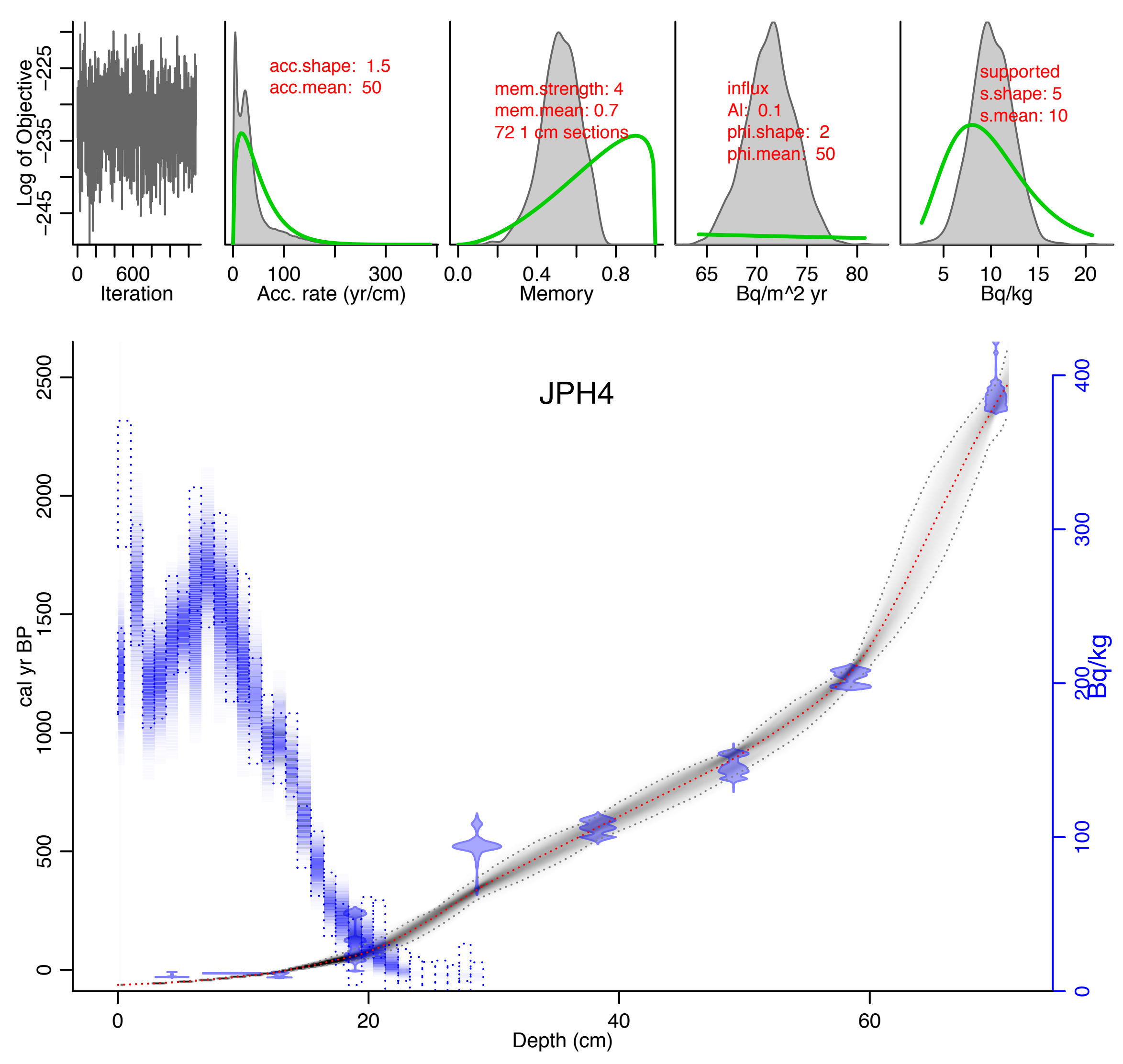
Log of Objective
−225
−235
−245
0
600
Iteration
acc.shape: 1.5
acc.mean: 50
0
100
300
Acc. rate (yr/cm)
mem.strength: 4
mem.mean: 0.7
72 1 cm sections
0.0
0.4
0.8
Memory
influx
Al: 0.1
phi.shape: 2
phi.mean: 50
65
70
75
80
Bq/m^2 yr
supported
s.shape: 5
s.mean: 10
5
10
15
20
Bq/kg
JPH4
cal yr BP
2500
2000
1500
1000
500
0
Depth (cm)
0
20
40
60
Bq/kg
400
300
200
100
0

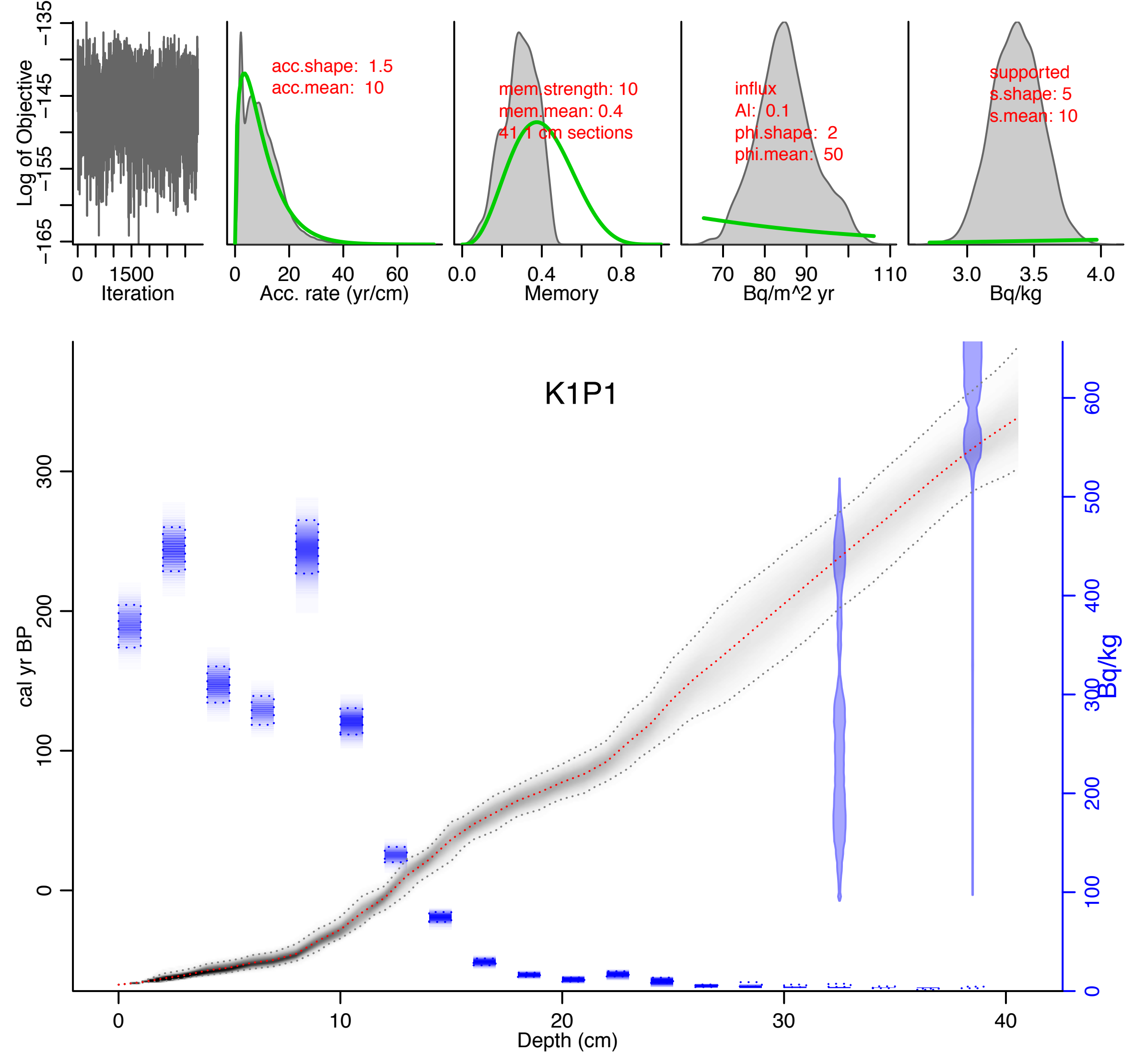

Log of Objective
Iteration
acc.shape: 1.5
acc.mean: 10
Acc. rate (yr/cm)
mem.strength: 10
mem.mean: 0.4
41 1 cm sections
Memory
influx
Al: 0.1
phi.shape: 2
phi.mean: 50
Bq/m^2 yr
supported
s.shape: 5
s.mean: 10
Bq/kg
K1P1
cal yr BP
Depth (cm)
Bq/kg

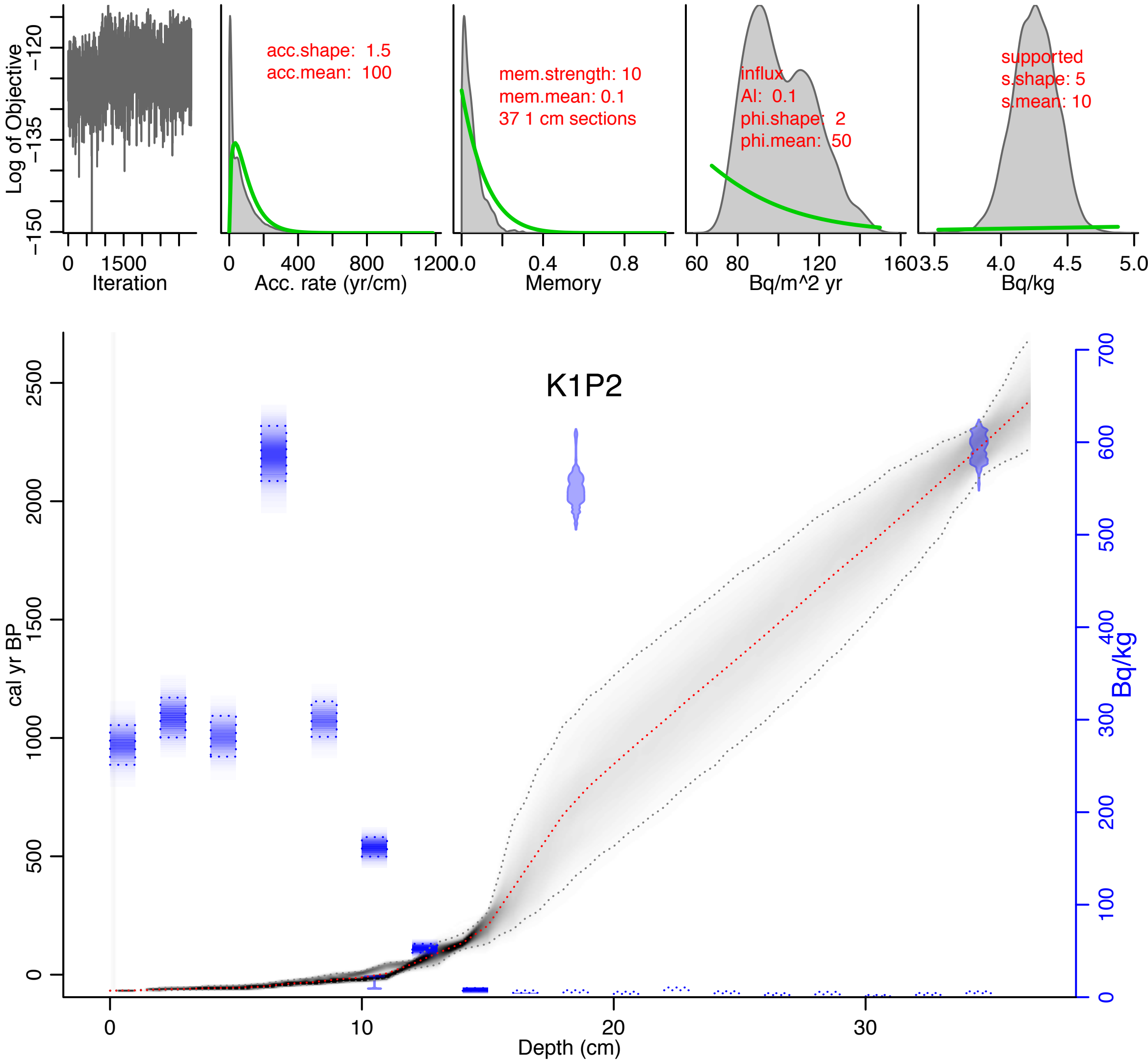
Log of Objective
−120
−135
−150
0
1500
Iteration
acc.shape: 1.5
acc.mean: 100
0
400
800
1200
Acc. rate (yr/cm)
mem.strength: 10
mem.mean: 0.1
37 1 cm sections
0.0
0.4
0.8
Memory
influx
Al: 0.1
phi.shape: 2
phi.mean: 50
60
80
120
160
Bq/m^2 yr
supported
s.shape: 5
s.mean: 10
3.5
4.0
4.5
5.0
Bq/kg
K1P2
cal yr BP
0
500
1000
1500
2000
2500
Depth (cm)
0
10
20
30
Bq/kg
0
100
200
300
400
500
600
700

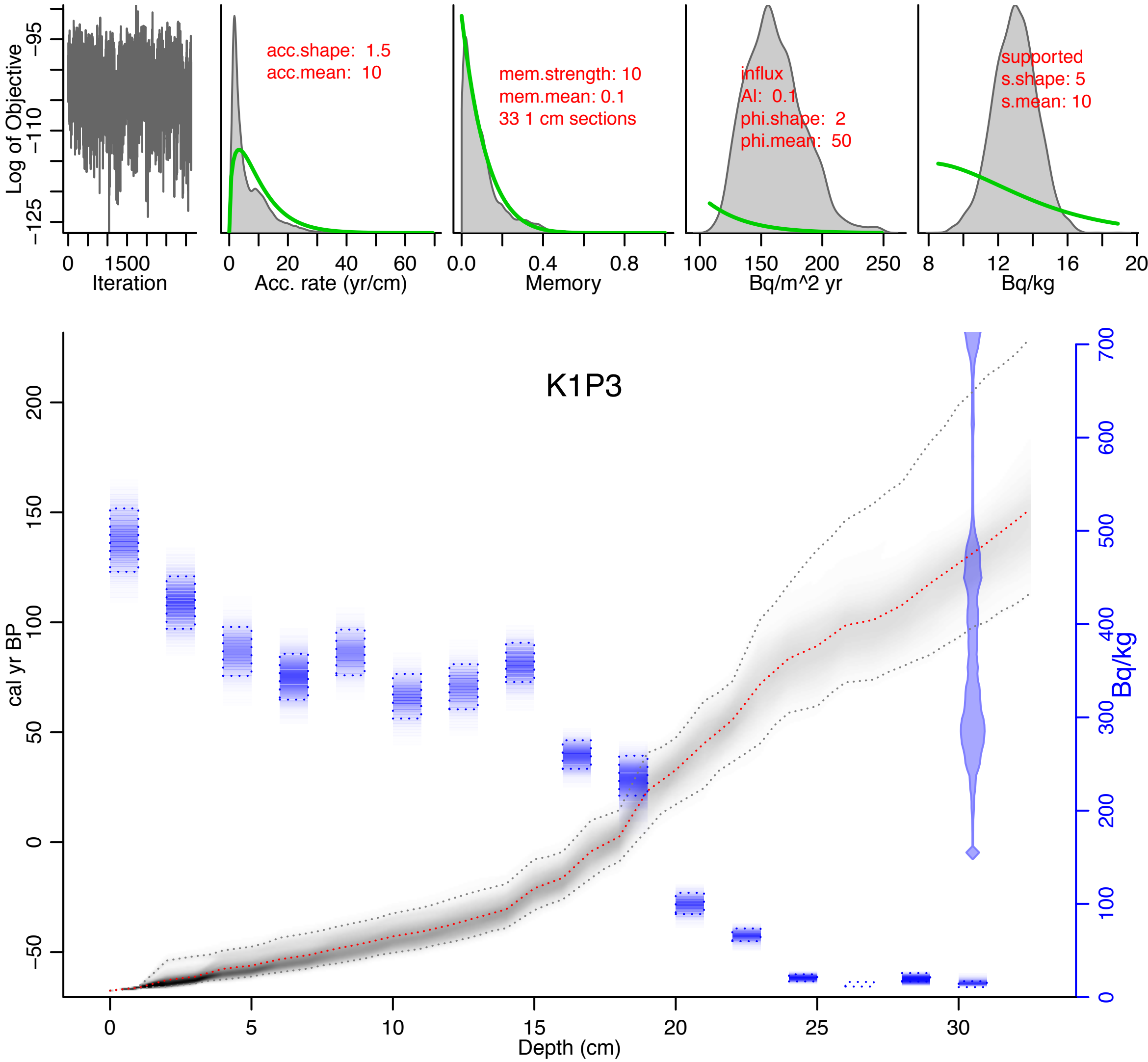

Log of Objective
Iteration
acc.shape: 1.5
acc.mean: 10
Acc. rate (yr/cm)
mem.strength: 10
mem.mean: 0.1
33 1 cm sections
Memory
influx
Al: 0.1
phi.shape: 2
phi.mean: 50
Bq/m^2 yr
supported
s.shape: 5
s.mean: 10
Bq/kg
K1P3
cal yr BP
Bq/kg
Depth (cm)

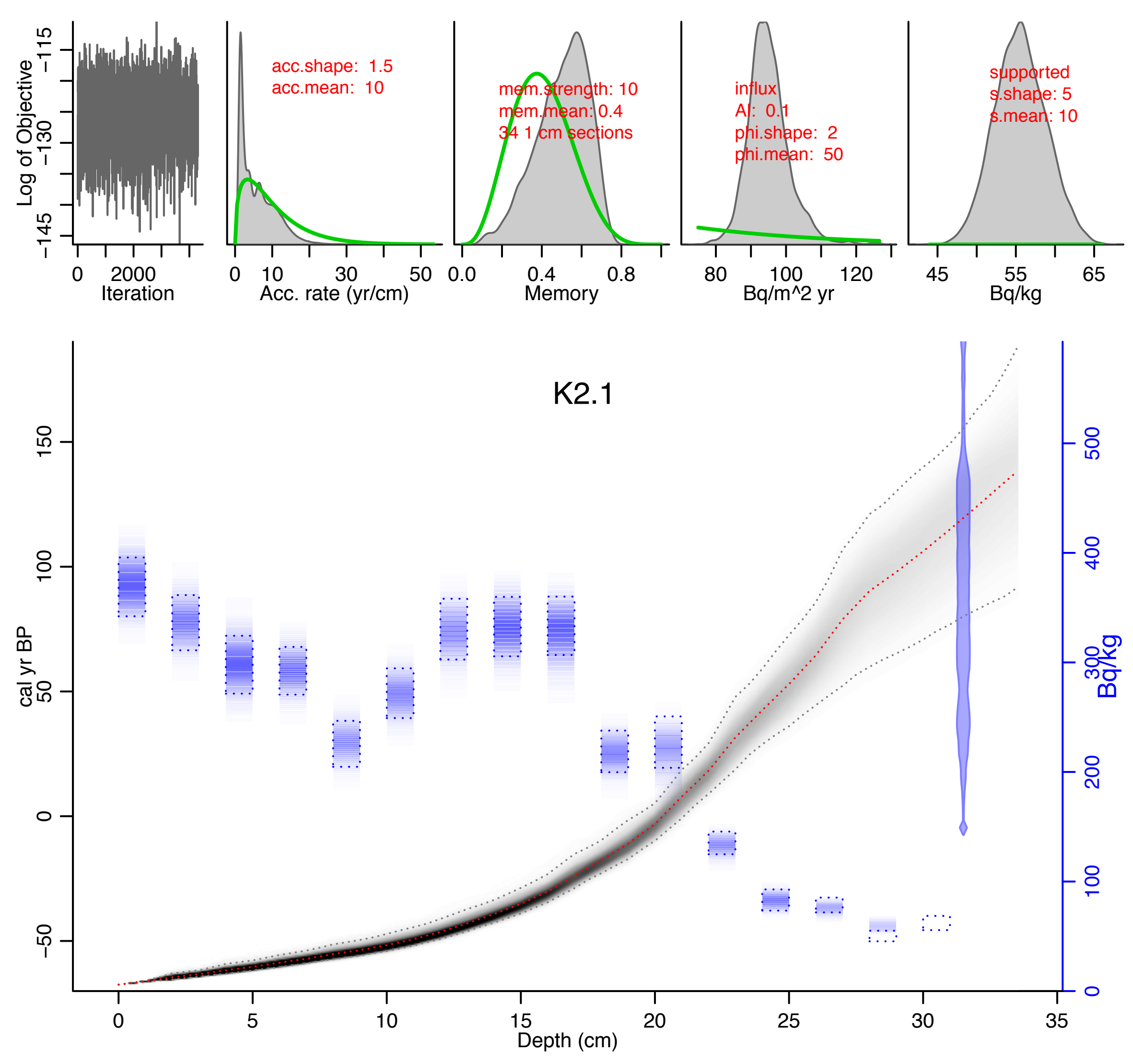

Log of Objective
−115
−130
−145
0
2000
Iteration
acc.shape: 1.5
acc.mean: 10
0
10
30
50
Acc. rate (yr/cm)
mem.strength: 10
mem.mean: 0.4
34 1 cm sections
0.0
0.4
0.8
Memory
influx
Al: 0.1
phi.shape: 2
phi.mean: 50
80
100
120
Bq/m^2 yr
supported
s.shape: 5
s.mean: 10
45
55
65
Bq/kg
K2.1
cal yr BP
150
100
50
0
−50
Bq/kg
500
400
300
200
100
0
0
5
10
15
20
25
30
35
Depth (cm)

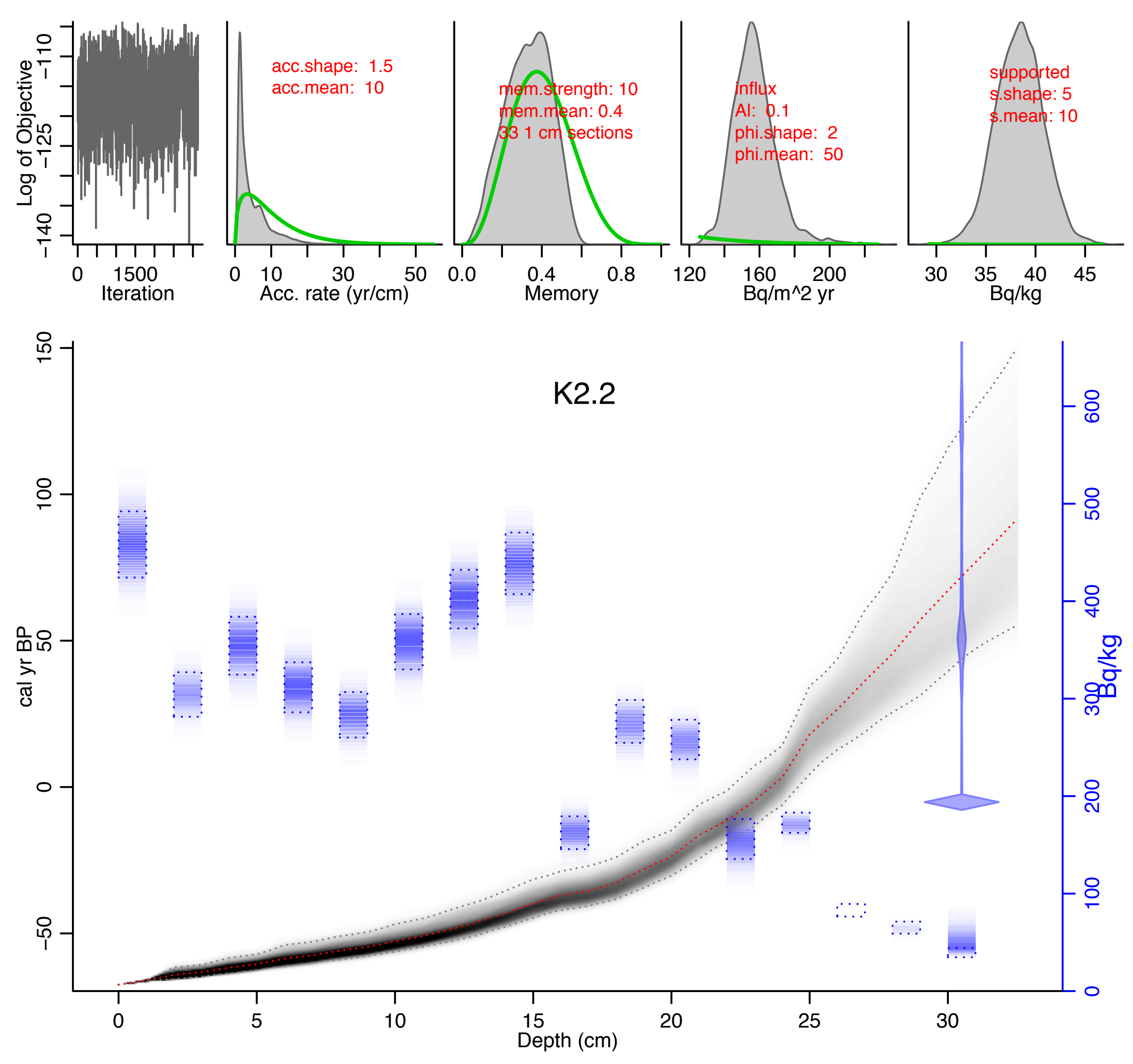

Log of Objective
Iteration
acc.shape: 1.5
acc.mean: 10
Acc. rate (yr/cm)
mem.strength: 10
mem.mean: 0.4
33 1 cm sections
Memory
influx
Al: 0.1
phi.shape: 2
phi.mean: 50
Bq/m^2 yr
supported
s.shape: 5
s.mean: 10
Bq/kg
K2.2
cal yr BP
Depth (cm)
Bq/kg

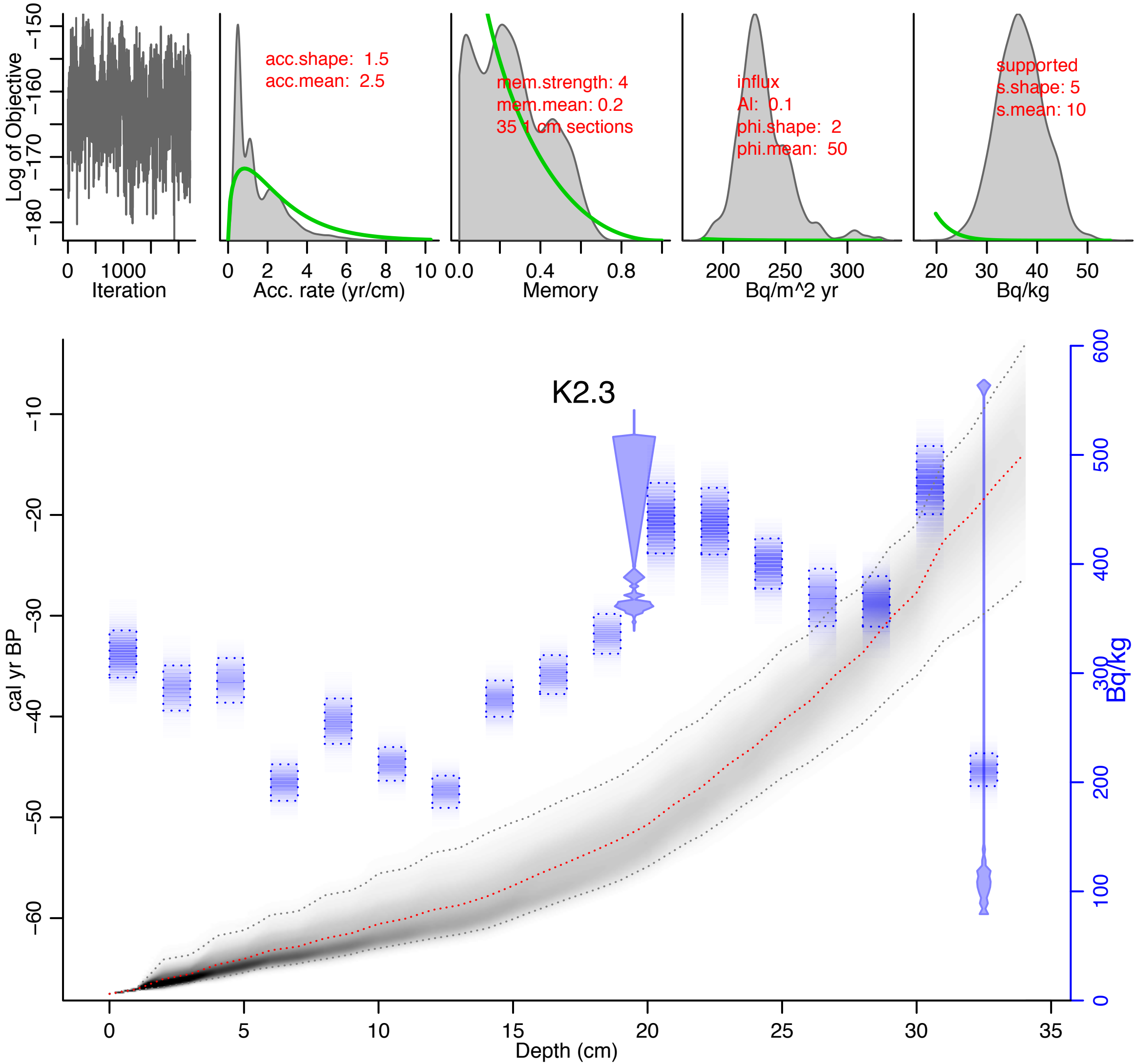

Log of Objective
Iteration
acc.shape: 1.5
acc.mean: 2.5
Acc. rate (yr/cm)
mem.strength: 4
mem.mean: 0.2
35 1 cm sections
Memory
influx
Al: 0.1
phi.shape: 2
phi.mean: 50
Bq/m^2 yr
supported
s.shape: 5
s.mean: 10
Bq/kg
K2.3
cal yr BP
Depth (cm)
Bq/kg

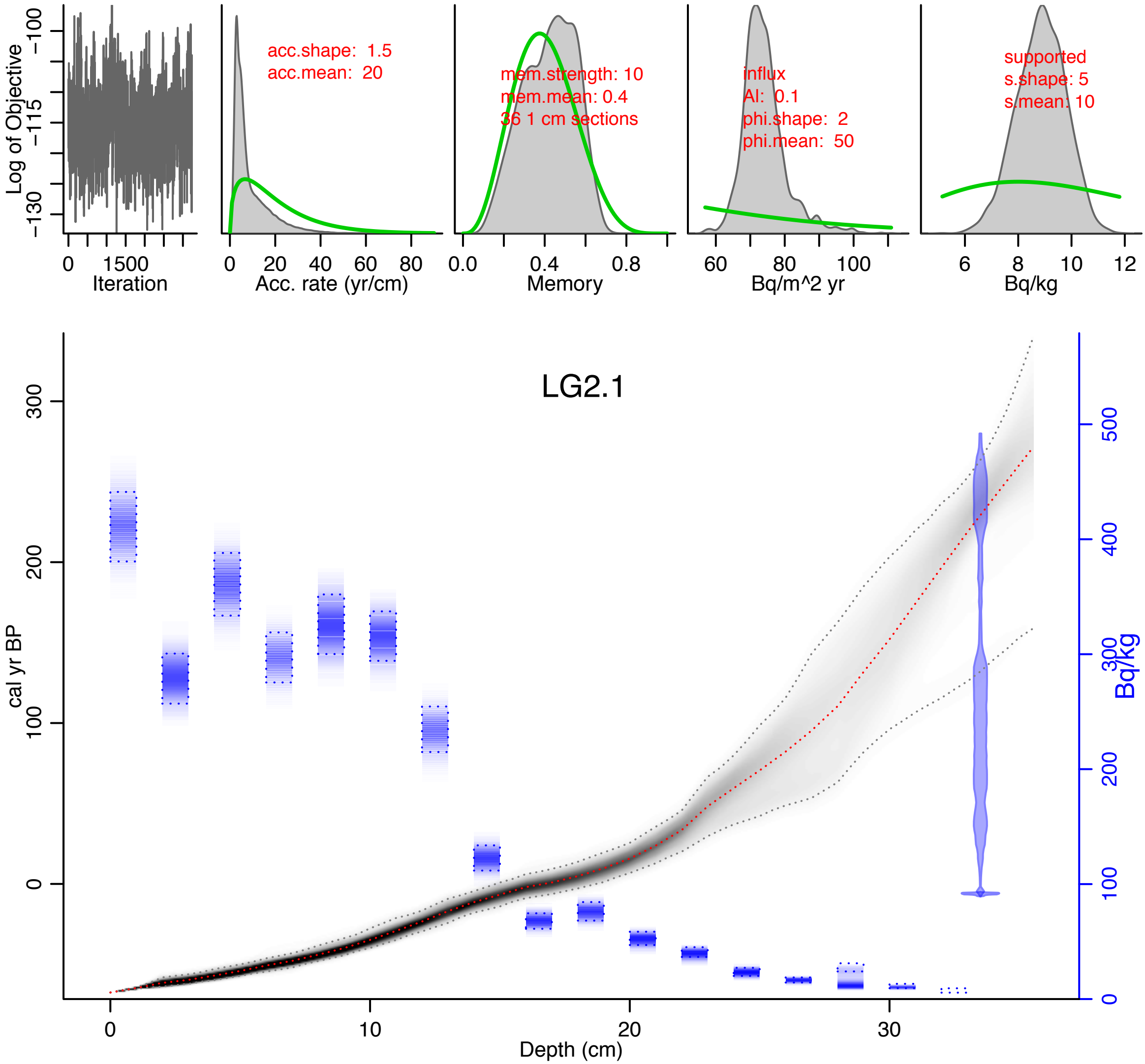

Log of Objective
−100
−115
−130
0
1500
Iteration
acc.shape: 1.5
acc.mean: 20
0
20
40
60
80
Acc. rate (yr/cm)
mem.strength: 10
mem.mean: 0.4
36 1 cm sections
0.0
0.4
0.8
Memory
influx
Al: 0.1
phi.shape: 2
phi.mean: 50
60
80
100
Bq/m^2 yr
supported
s.shape: 5
s.mean: 10
6
8
10
12
Bq/kg
LG2.1
cal yr BP
0
100
200
300
Depth (cm)
0
10
20
30
Bq/kg
0
100
200
300
400
500

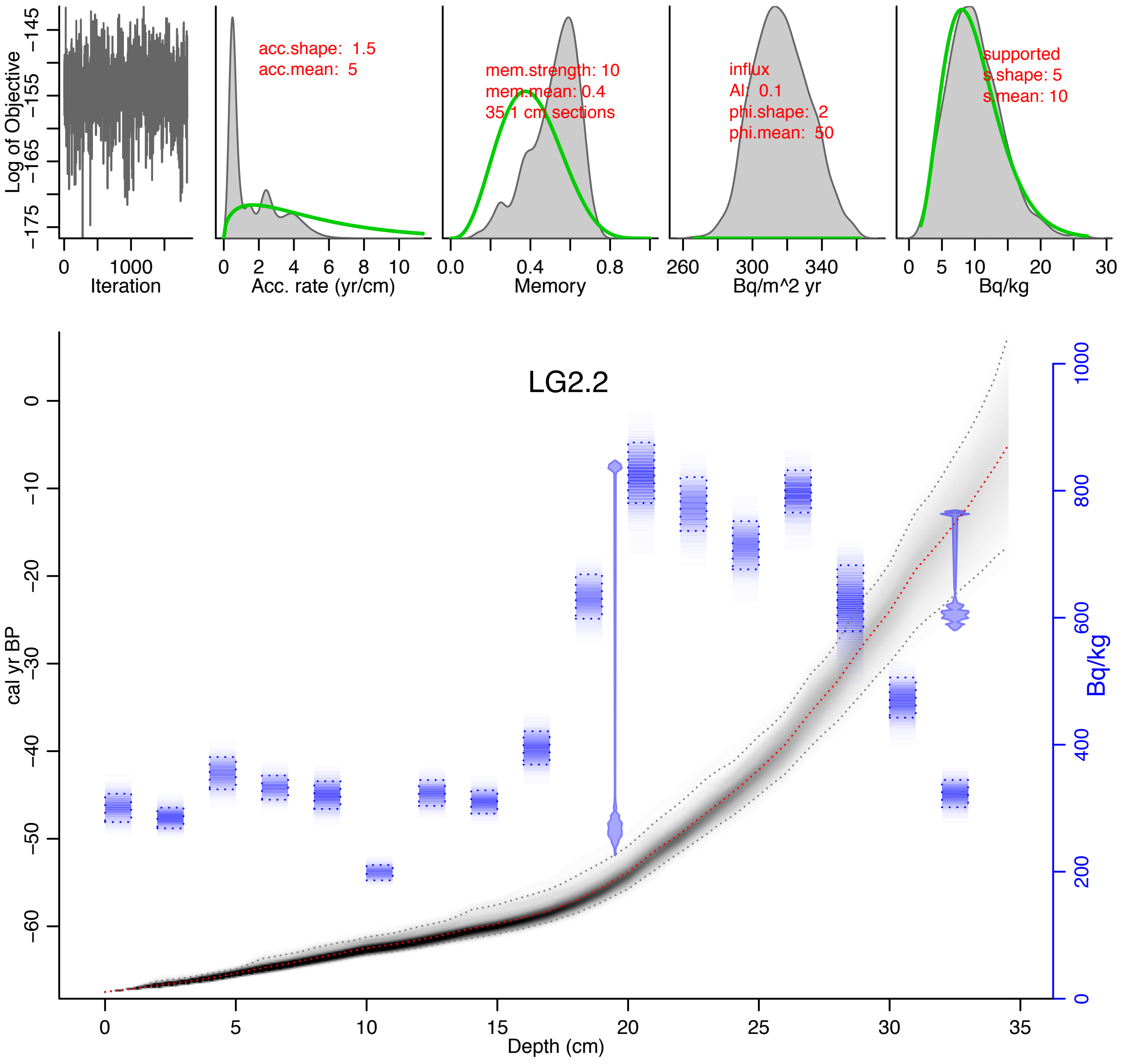

Log of Objective
Iteration
acc.shape: 1.5
acc.mean: 5
Acc. rate (yr/cm)
mem.strength: 10
mem.mean: 0.4
35 1 cm sections
Memory
influx
Al: 0.1
phi.shape: 2
phi.mean: 50
Bq/m^2 yr
supported
s.shape: 5
s.mean: 10
Bq/kg
LG2.2
cal yr BP
Depth (cm)
Bq/kg

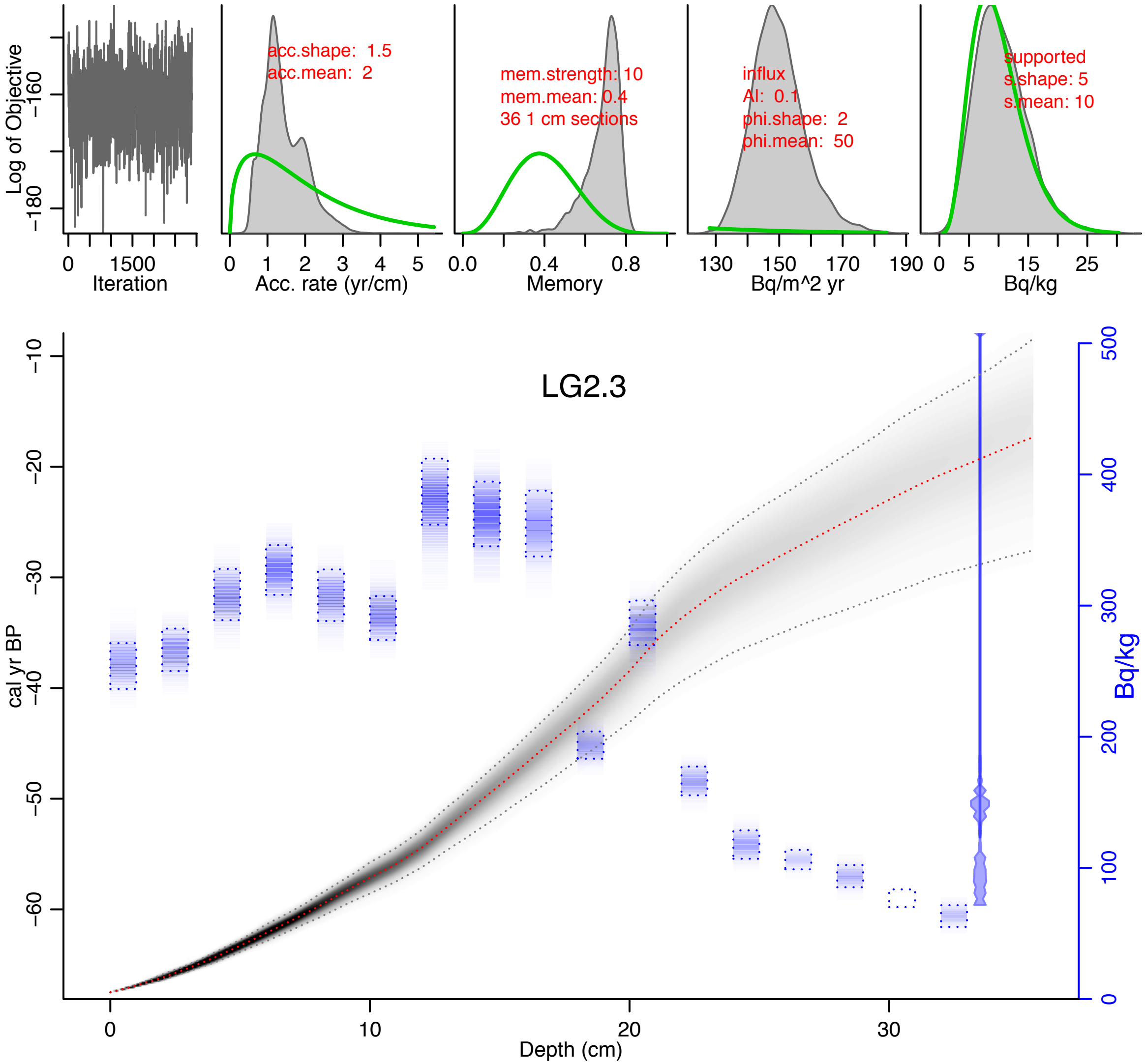

Log of Objective
−160
−180
0
1500
Iteration
acc.shape: 1.5
acc.mean: 2
0 1 2 3 4 5
Acc. rate (yr/cm)
mem.strength: 10
mem.mean: 0.4
36 1 cm sections
0.0 0.4 0.8
Memory
influx
Al: 0.1
phi.shape: 2
phi.mean: 50
130 150 170 190
Bq/m^2 yr
supported
s.shape: 5
s.mean: 10
0 5 15 25
Bq/kg
LG2.3
cal yr BP
−10 −20 −30 −40 −50 −60
0 10 20 30
Depth (cm)
Bq/kg
0 100 200 300 400 500

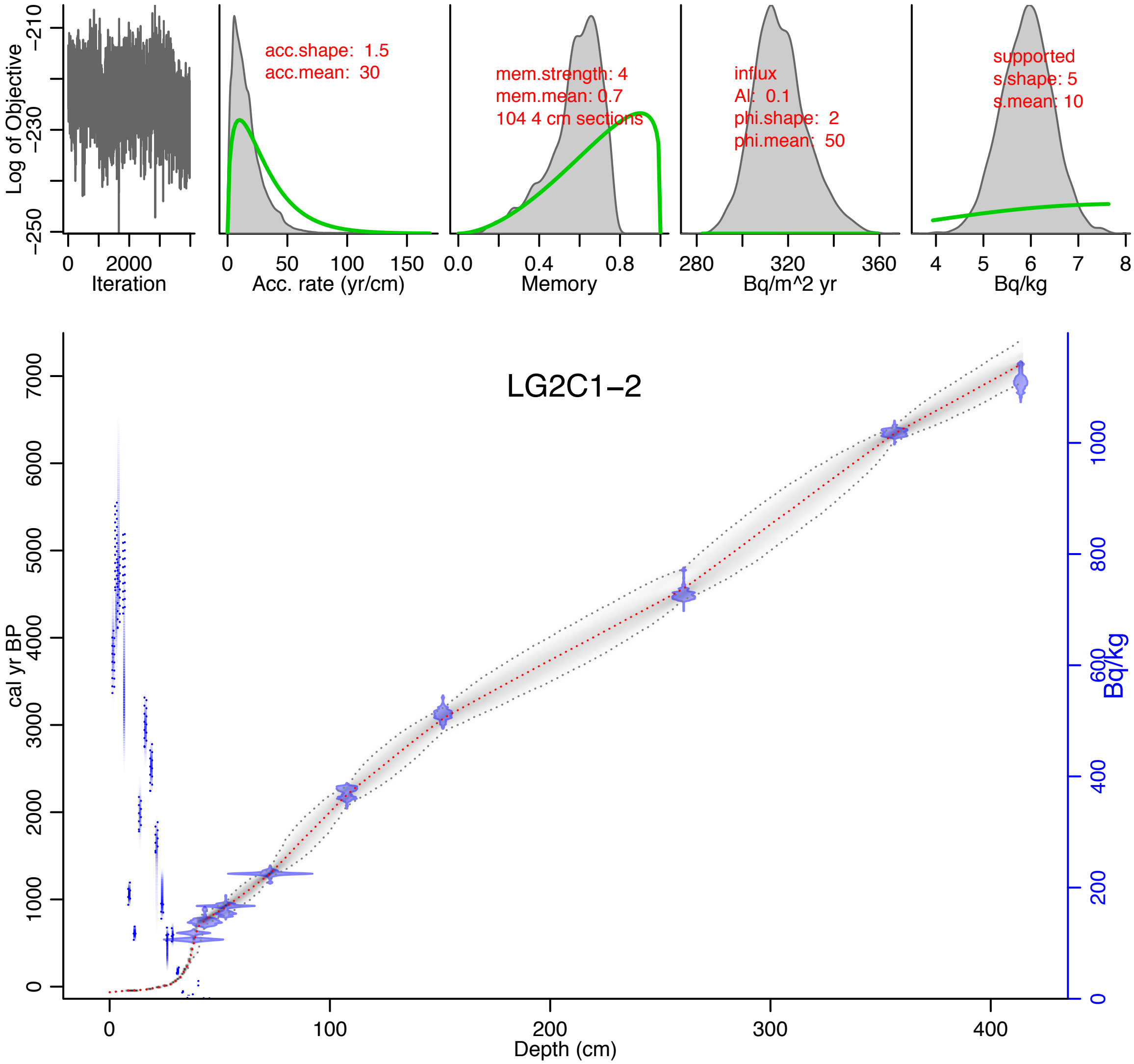

Log of Objective
−210
−230
−250
0
2000
Iteration
acc.shape: 1.5
acc.mean: 30
0
50
100
150
Acc. rate (yr/cm)
mem.strength: 4
mem.mean: 0.7
104 4 cm sections
0.0
0.4
0.8
Memory
influx
Al: 0.1
phi.shape: 2
phi.mean: 50
280
320
360
Bq/m^2 yr
supported
s.shape: 5
s.mean: 10
4
5
6
7
8
Bq/kg
LG2C1−2
cal yr BP
0
1000
2000
3000
4000
5000
6000
7000
Depth (cm)
0
100
200
300
400
Bq/kg
0
200
400
600
800
1000

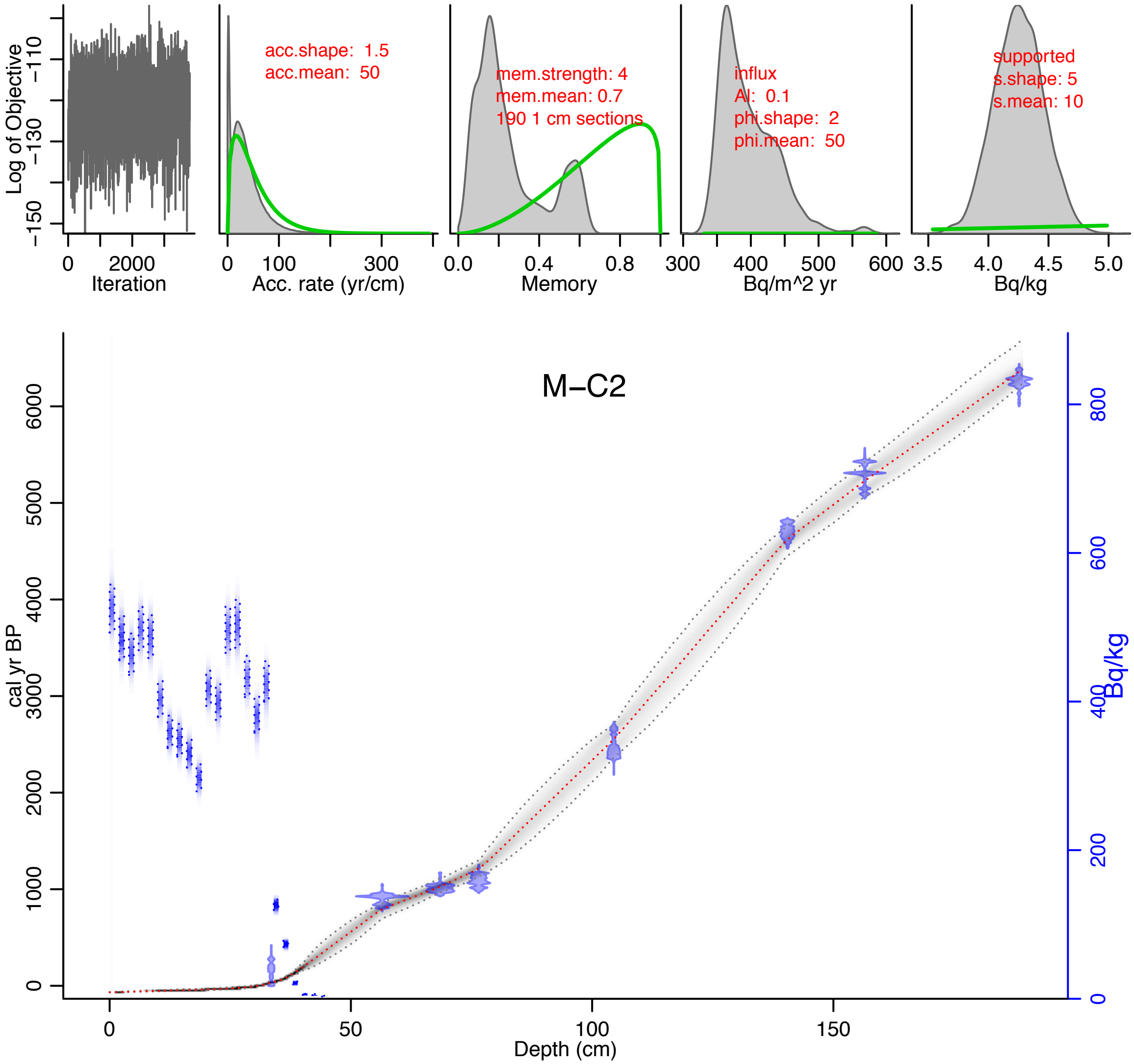

Log of Objective
−110
−130
−150
0
2000
Iteration
acc.shape: 1.5
acc.mean: 50
0
100
300
Acc. rate (yr/cm)
mem.strength: 4
mem.mean: 0.7
190 1 cm sections
0.0
0.4
0.8
Memory
influx
Al: 0.1
phi.shape: 2
phi.mean: 50
300
400
500
600
Bq/m^2 yr
supported
s.shape: 5
s.mean: 10
3.5
4.0
4.5
5.0
Bq/kg
M−C2
cal yr BP
0
1000
2000
3000
4000
5000
6000
Bq/kg
0
200
400
600
800
0
50
100
150
Depth (cm)

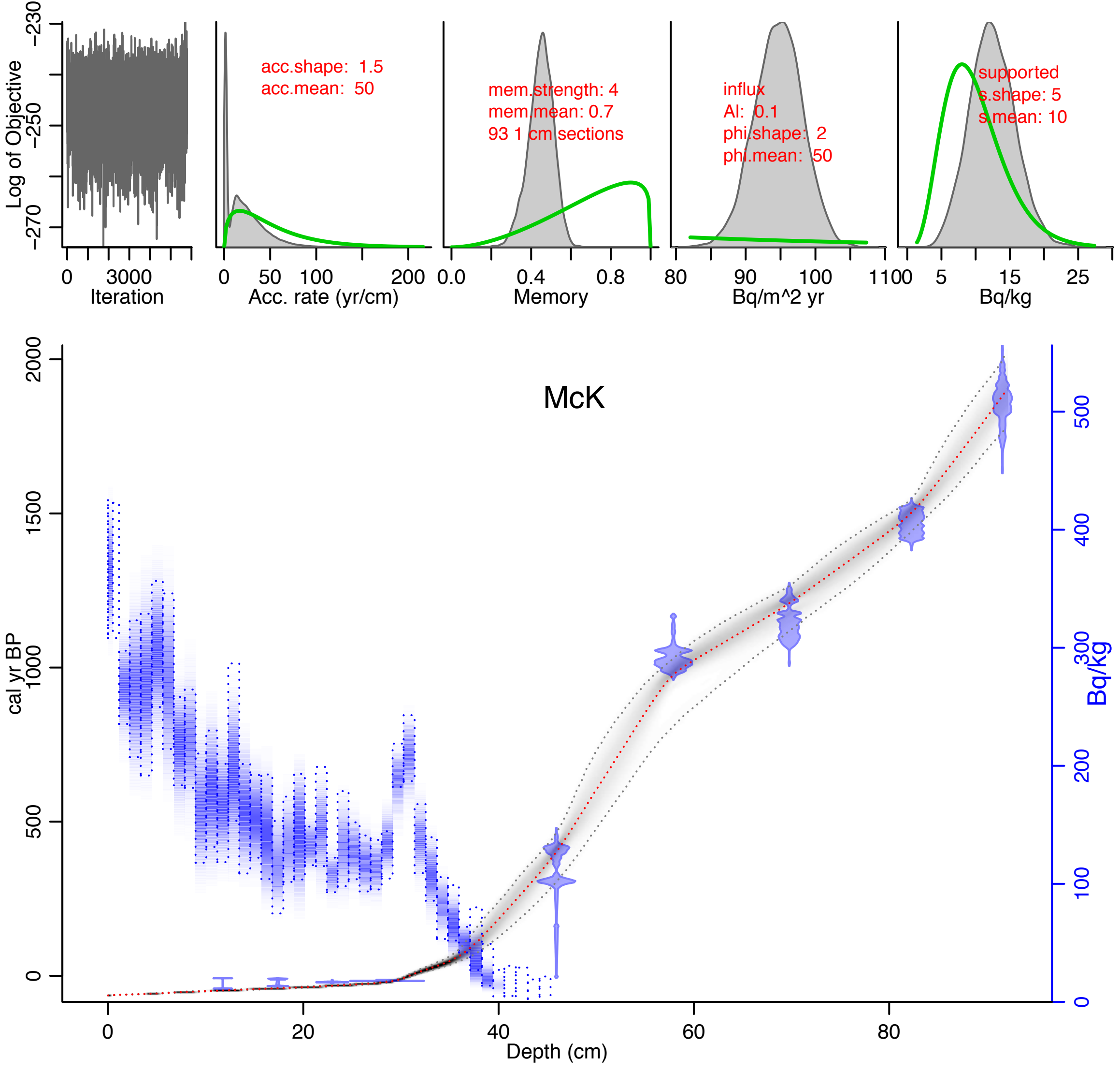

Log of Objective
Iteration
acc.shape: 1.5
acc.mean: 50
Acc. rate (yr/cm)
mem.strength: 4
mem.mean: 0.7
93 1 cm sections
Memory
influx
Al: 0.1
phi.shape: 2
phi.mean: 50
Bq/m^2 yr
supported
s.shape: 5
s.mean: 10
Bq/kg
McK
cal yr BP
Bq/kg
Depth (cm)

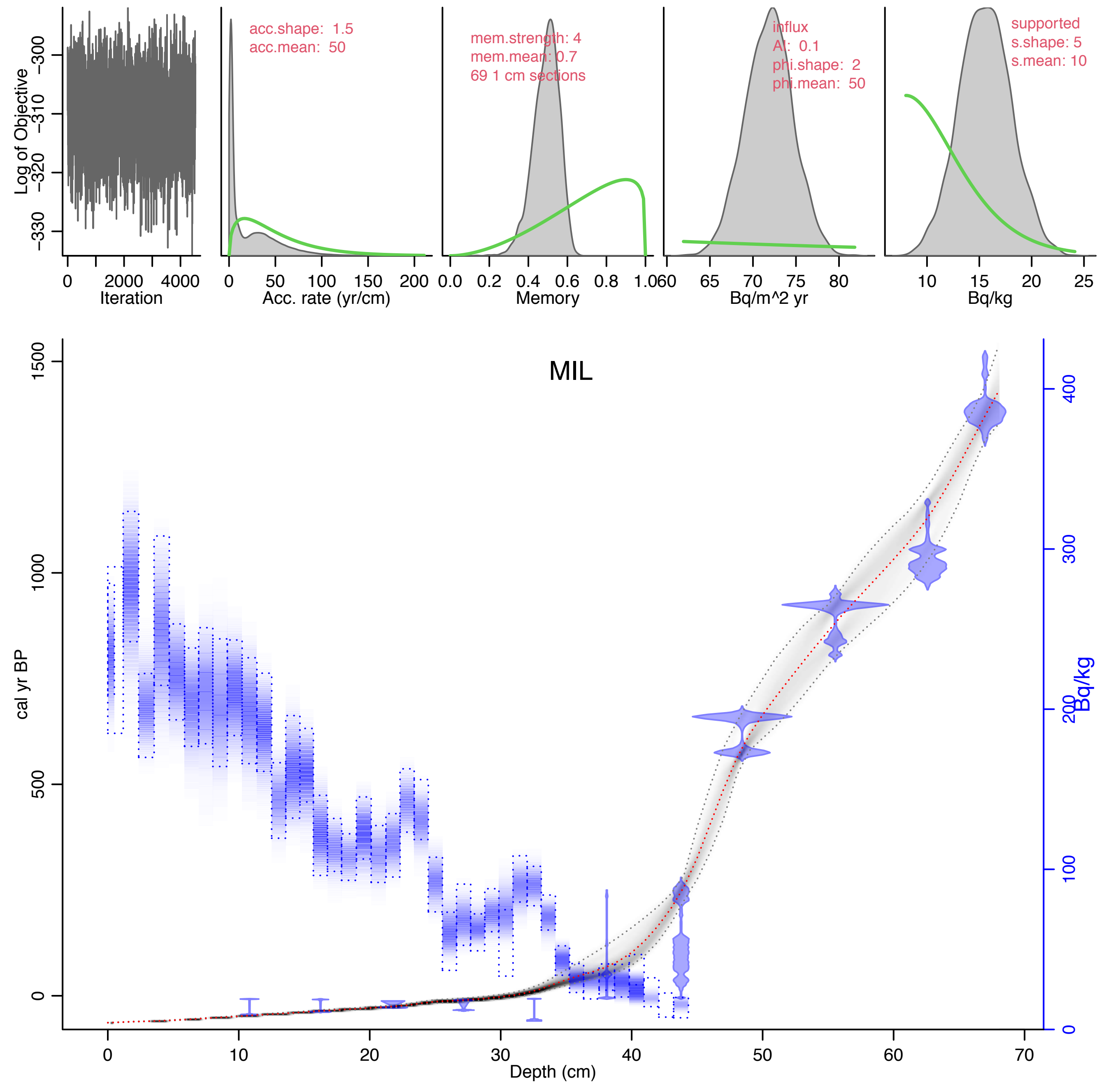

Log of Objective
Iteration
acc.shape: 1.5
acc.mean: 50
Acc. rate (yr/cm)
mem.strength: 4
mem.mean: 0.7
69 1 cm sections
Memory
influx
Al: 0.1
phi.shape: 2
phi.mean: 50
Bq/m^2 yr
supported
s.shape: 5
s.mean: 10
Bq/kg
MIL
cal yr BP
Bq/kg
Depth (cm)

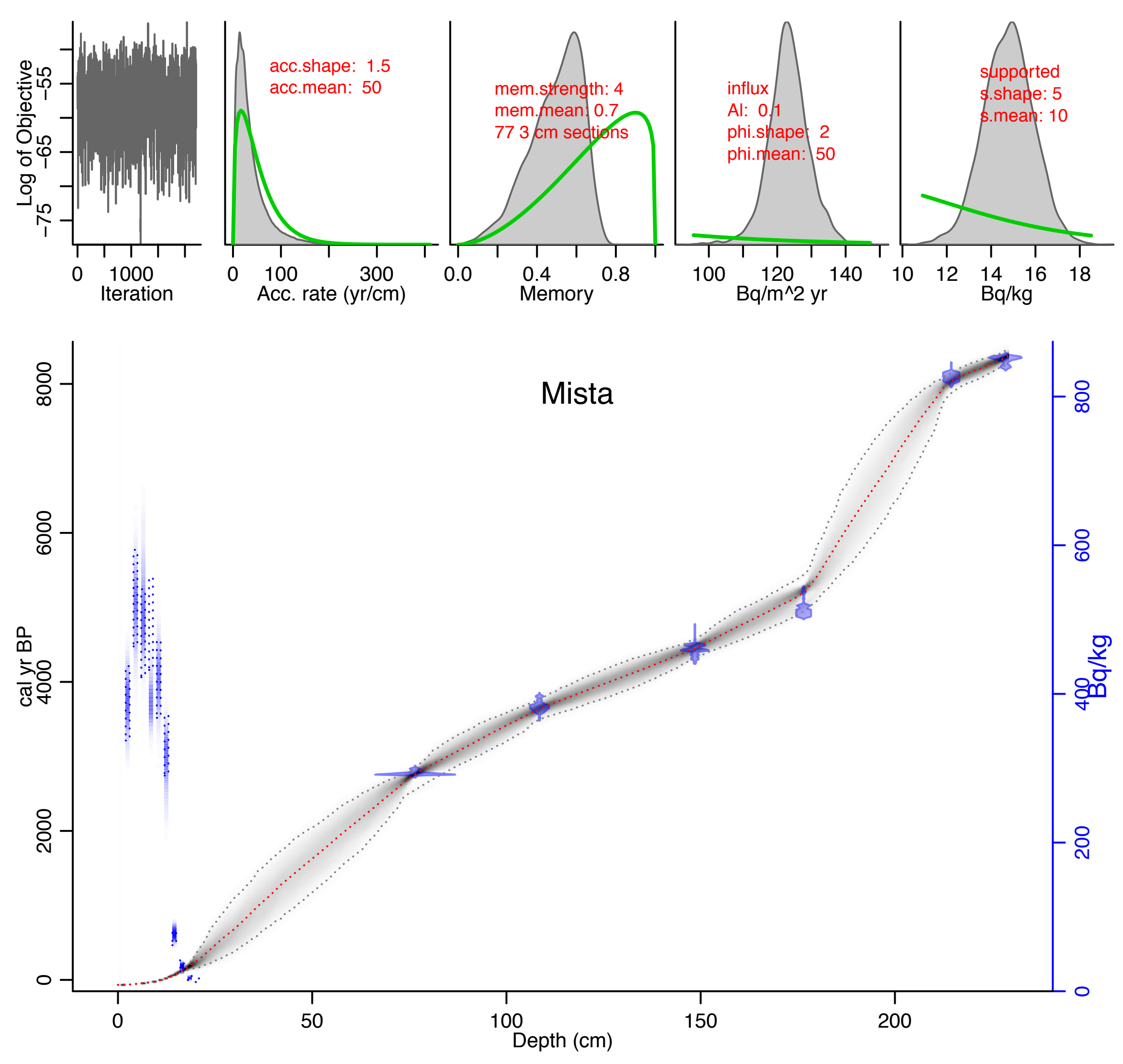

Log of Objective
−55
−65
−75
0
1000
Iteration
acc.shape: 1.5
acc.mean: 50
0
100
300
Acc. rate (yr/cm)
mem.strength: 4
mem.mean: 0.7
77 3 cm sections
0.0
0.4
0.8
Memory
influx
Al: 0.1
phi.shape: 2
phi.mean: 50
100
120
140
Bq/m^2 yr
supported
s.shape: 5
s.mean: 10
10
12
14
16
18
Bq/kg
Mista
cal yr BP
0
2000
4000
6000
8000
Depth (cm)
0
50
100
150
200
Bq/kg
0
200
400
600
800

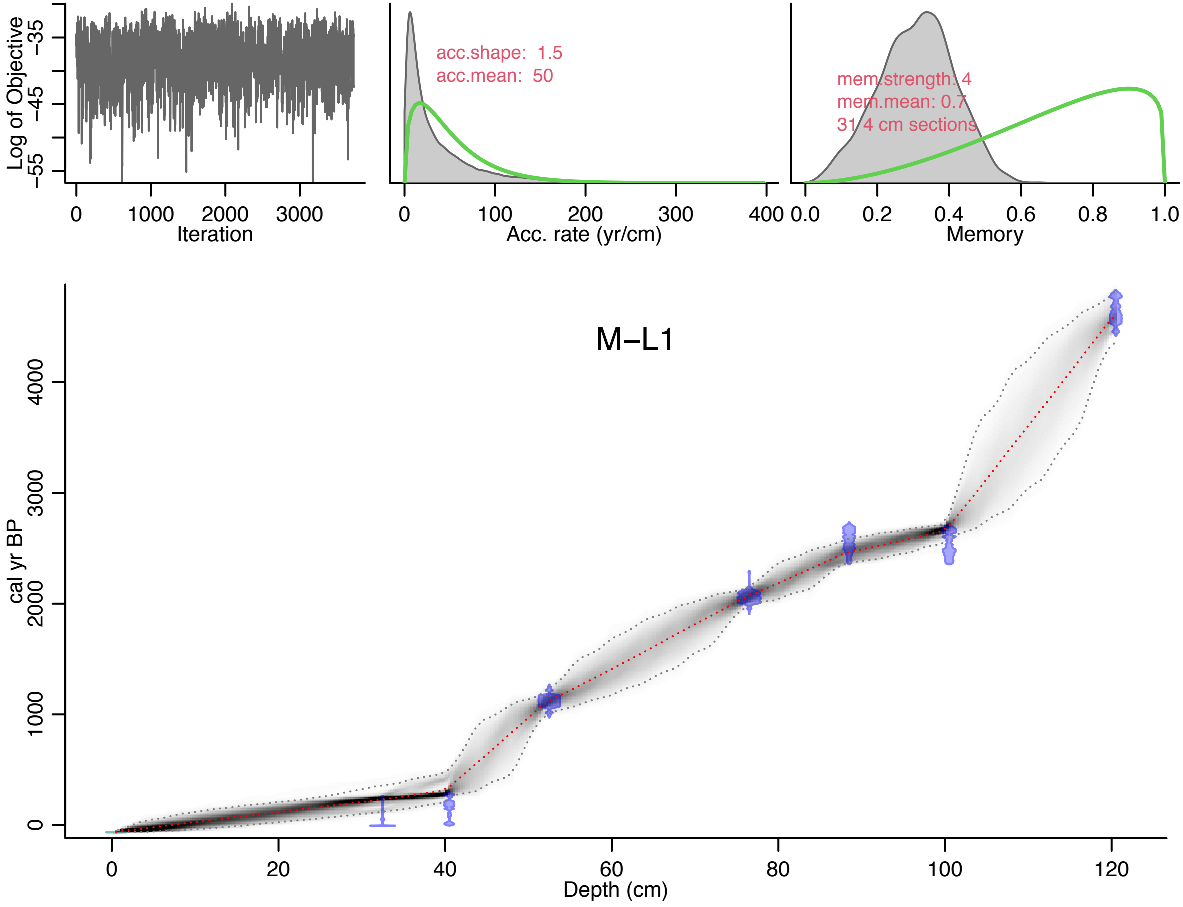

Log of Objective
−35
−45
−55
0
1000
2000
3000
Iteration
acc.shape: 1.5
acc.mean: 50
0
100
200
300
400
Acc. rate (yr/cm)
mem.strength: 4
mem.mean: 0.7
31 4 cm sections
0.0
0.2
0.4
0.6
0.8
1.0
Memory
M–L1
cal yr BP
0
1000
2000
3000
4000
0
20
40
60
80
100
120
Depth (cm)

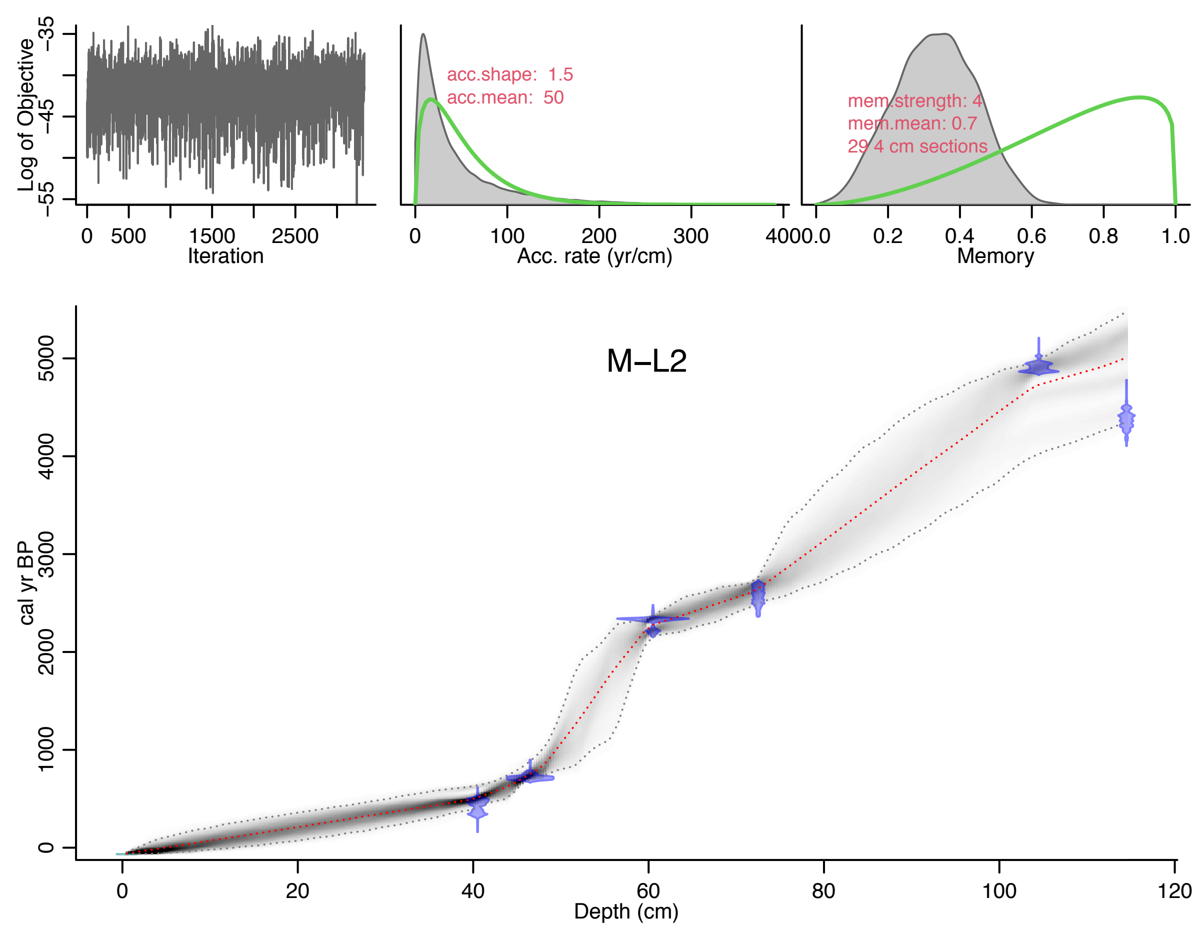

Log of Objective
Iteration
acc.shape: 1.5
acc.mean: 50
Acc. rate (yr/cm)
mem.strength: 4
mem.mean: 0.7
29 4 cm sections
Memory
M–L2
cal yr BP
Depth (cm)

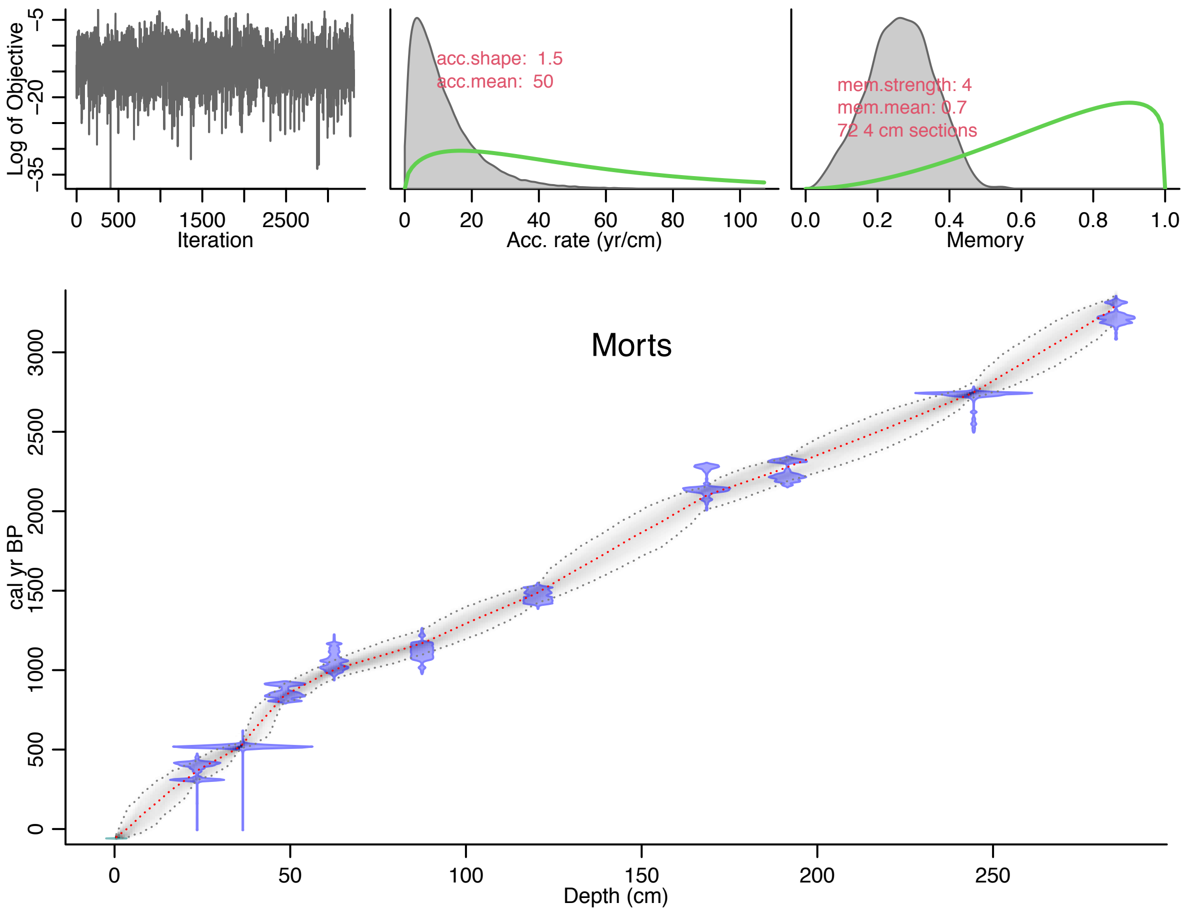

Log of Objective
−5
−20
−35
0
500
1500
2500
Iteration
acc.shape: 1.5
acc.mean: 50
0
20
40
60
80
100
Acc. rate (yr/cm)
mem.strength: 4
mem.mean: 0.7
72 4 cm sections
0.0
0.2
0.4
0.6
0.8
1.0
Memory
Morts
cal yr BP
0
500
1000
1500
2000
2500
3000
0
50
100
150
200
250
Depth (cm)

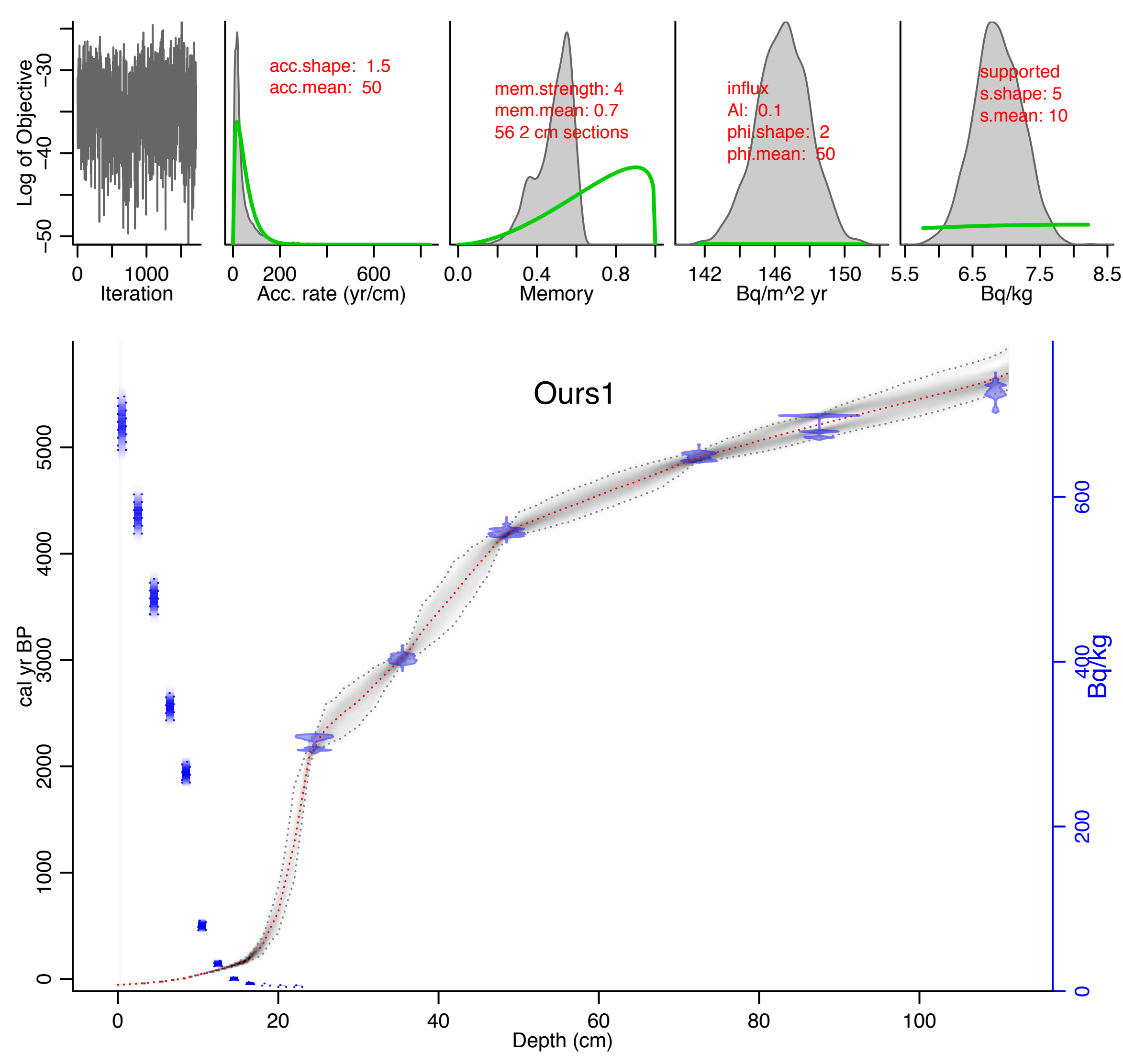

Log of Objective
−30
−40
−50
0
1000
Iteration
acc.shape: 1.5
acc.mean: 50
0
200
600
Acc. rate (yr/cm)
mem.strength: 4
mem.mean: 0.7
56 2 cm sections
0.0
0.4
0.8
Memory
influx
Al: 0.1
phi.shape: 2
phi.mean: 50
142
146
150
Bq/m^2 yr
supported
s.shape: 5
s.mean: 10
5.5
6.5
7.5
8.5
Bq/kg
Ours1
cal yr BP
0
1000
2000
3000
4000
5000
Depth (cm)
0
20
40
60
80
100
Bq/kg
0
200
400
600

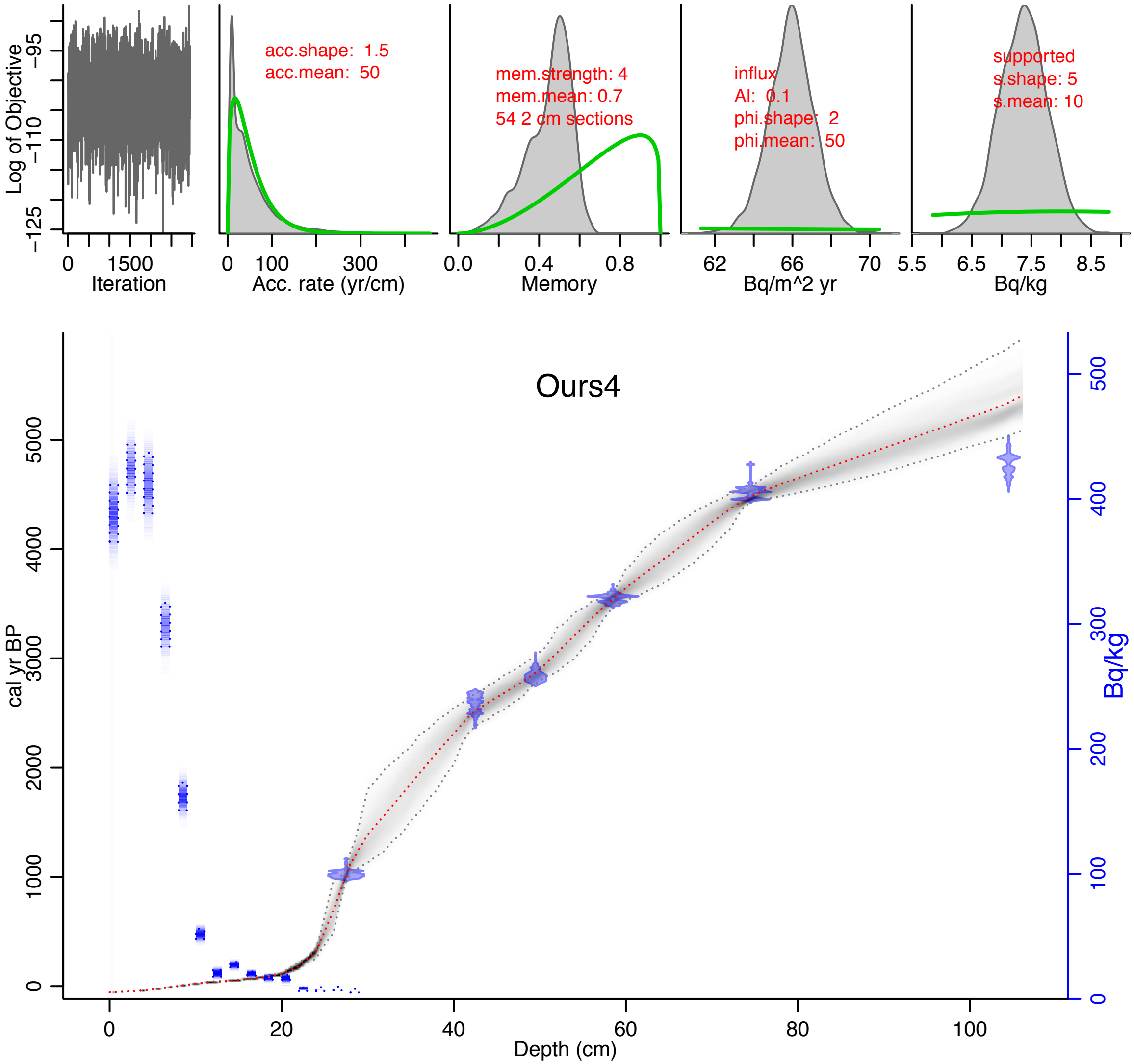

Log of Objective
−95
−110
−125
0
1500
Iteration
acc.shape: 1.5
acc.mean: 50
0
100
300
Acc. rate (yr/cm)
mem.strength: 4
mem.mean: 0.7
54 2 cm sections
0.0
0.4
0.8
Memory
influx
Al: 0.1
phi.shape: 2
phi.mean: 50
62
66
70
Bq/m^2 yr
supported
s.shape: 5
s.mean: 10
5.5
6.5
7.5
8.5
Bq/kg
Ours4
cal yr BP
0
1000
2000
3000
4000
5000
0
20
40
60
80
100
Depth (cm)
Bq/kg
0
100
200
300
400
500

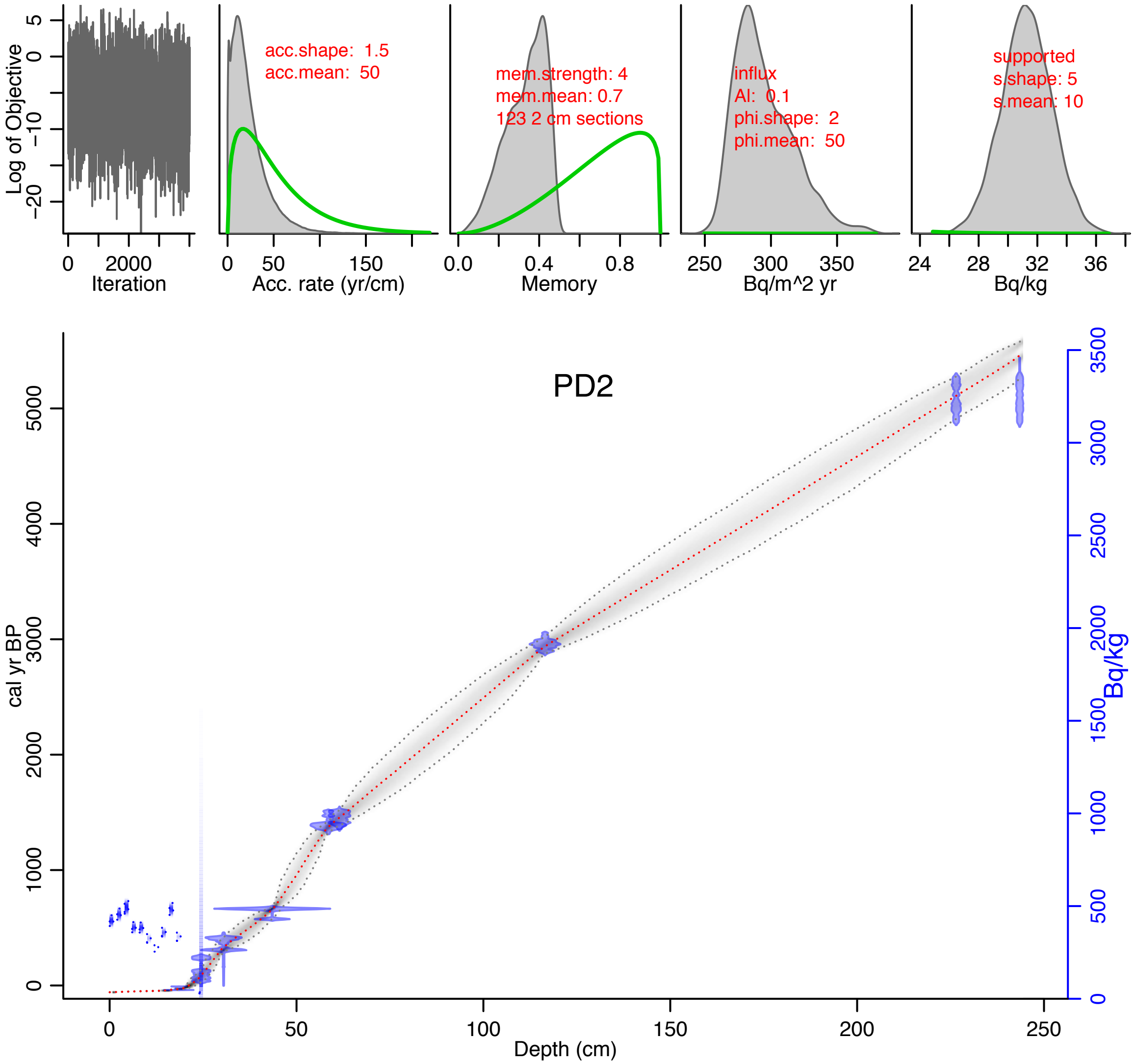
Log of Objective
Iteration
acc.shape: 1.5
acc.mean: 50
Acc. rate (yr/cm)
mem.strength: 4
mem.mean: 0.7
123 2 cm sections
Memory
influx
Al: 0.1
phi.shape: 2
phi.mean: 50
Bq/m^2 yr
supported
s.shape: 5
s.mean: 10
Bq/kg
PD2
cal yr BP
Depth (cm)
Bq/kg

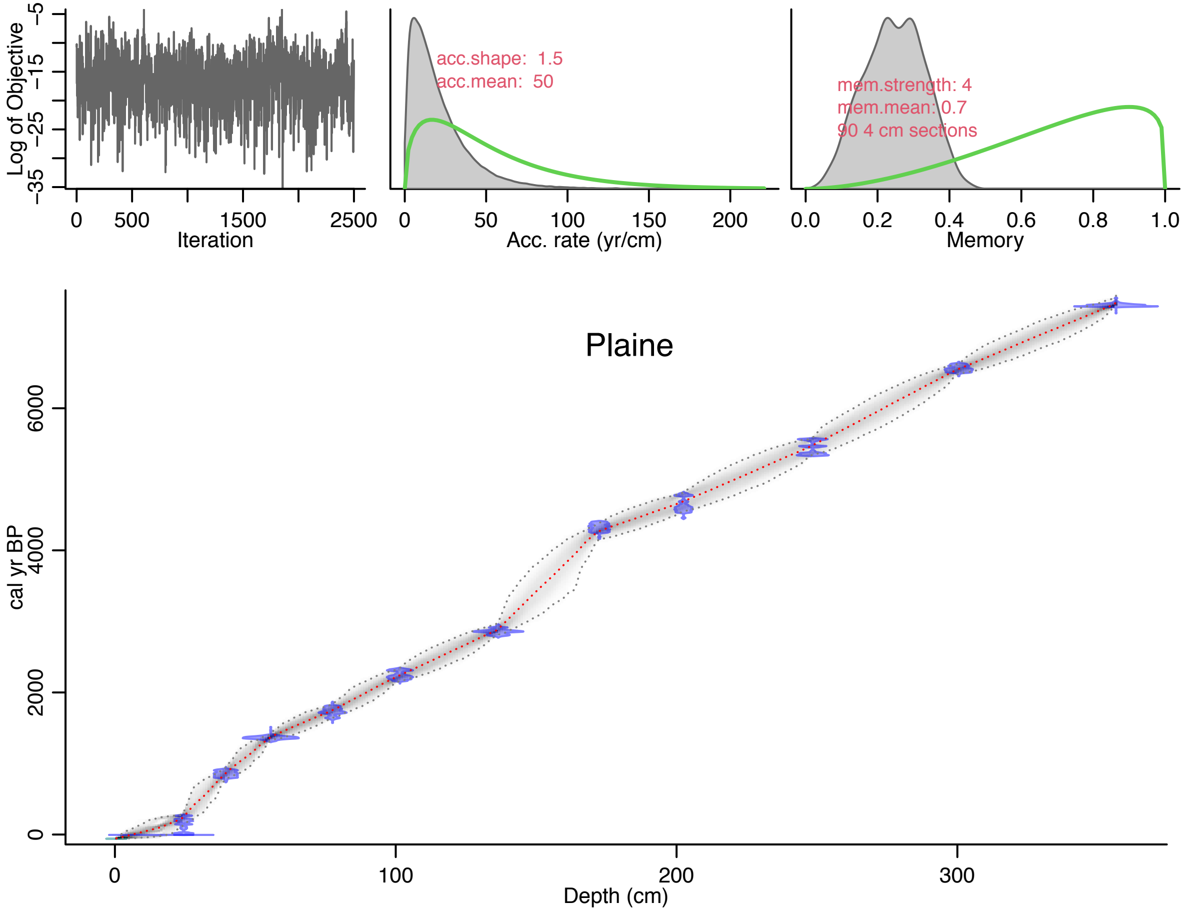

Log of Objective
−5
−15
−25
−35
0
500
1500
2500
Iteration
acc.shape: 1.5
acc.mean: 50
0
50
100
150
200
Acc. rate (yr/cm)
mem.strength: 4
mem.mean: 0.7
90 4 cm sections
0.0
0.2
0.4
0.6
0.8
1.0
Memory
Plaine
cal yr BP
0
2000
4000
6000
0
100
200
300
Depth (cm)

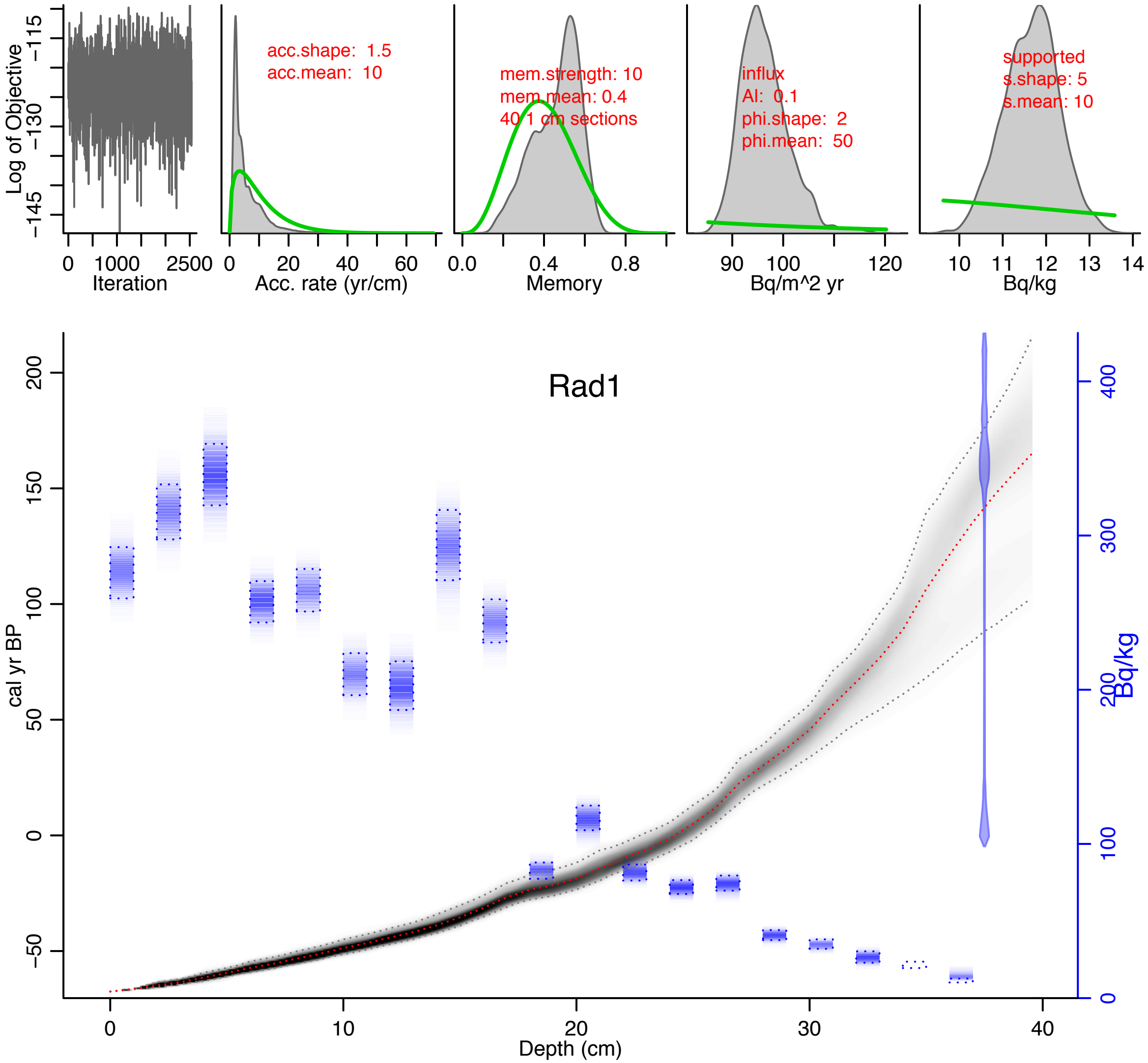

Log of Objective
−115
−130
−145
0
1000
2500
Iteration
acc.shape: 1.5
acc.mean: 10
0
20
40
60
Acc. rate (yr/cm)
mem.strength: 10
mem.mean: 0.4
40 1 cm sections
0.0
0.4
0.8
Memory
influx
Al: 0.1
phi.shape: 2
phi.mean: 50
90
100
120
Bq/m^2 yr
supported
s.shape: 5
s.mean: 10
10
11
12
13
14
Bq/kg
Rad1
cal yr BP
200
150
100
50
0
−50
Bq/kg
400
300
200
100
0
0
10
20
30
40
Depth (cm)

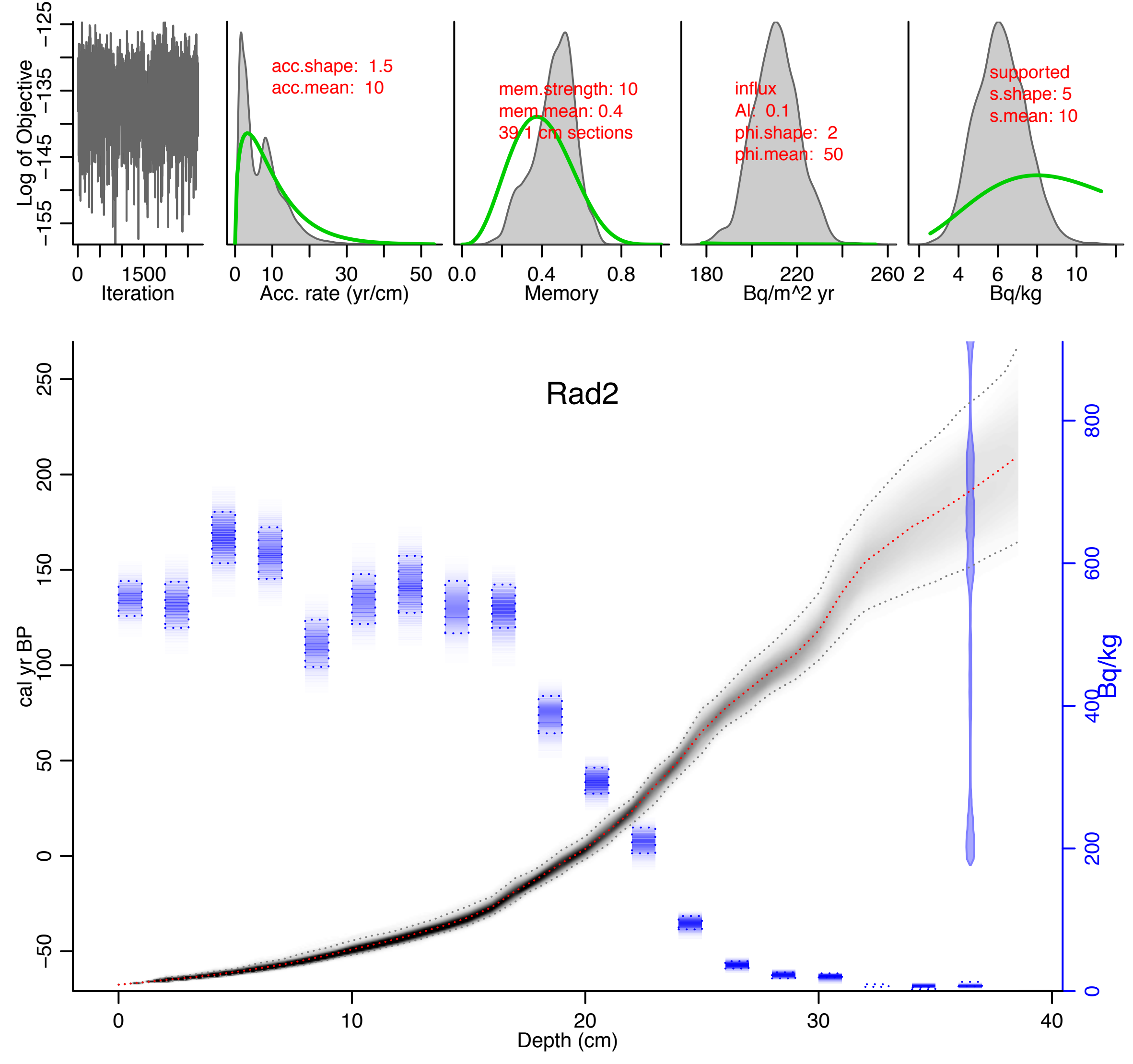

Log of Objective
−125
−135
−145
−155
0
1500
Iteration
acc.shape: 1.5
acc.mean: 10
0
10
30
50
Acc. rate (yr/cm)
mem.strength: 10
mem.mean: 0.4
39 1 cm sections
0.0
0.4
0.8
Memory
influx
Al: 0.1
phi.shape: 2
phi.mean: 50
180
220
260
Bq/m^2 yr
supported
s.shape: 5
s.mean: 10
2
4
6
8
10
Bq/kg
Rad2
cal yr BP
250
200
150
100
50
0
−50
Bq/kg
800
600
400
200
0
0
10
20
30
40
Depth (cm)

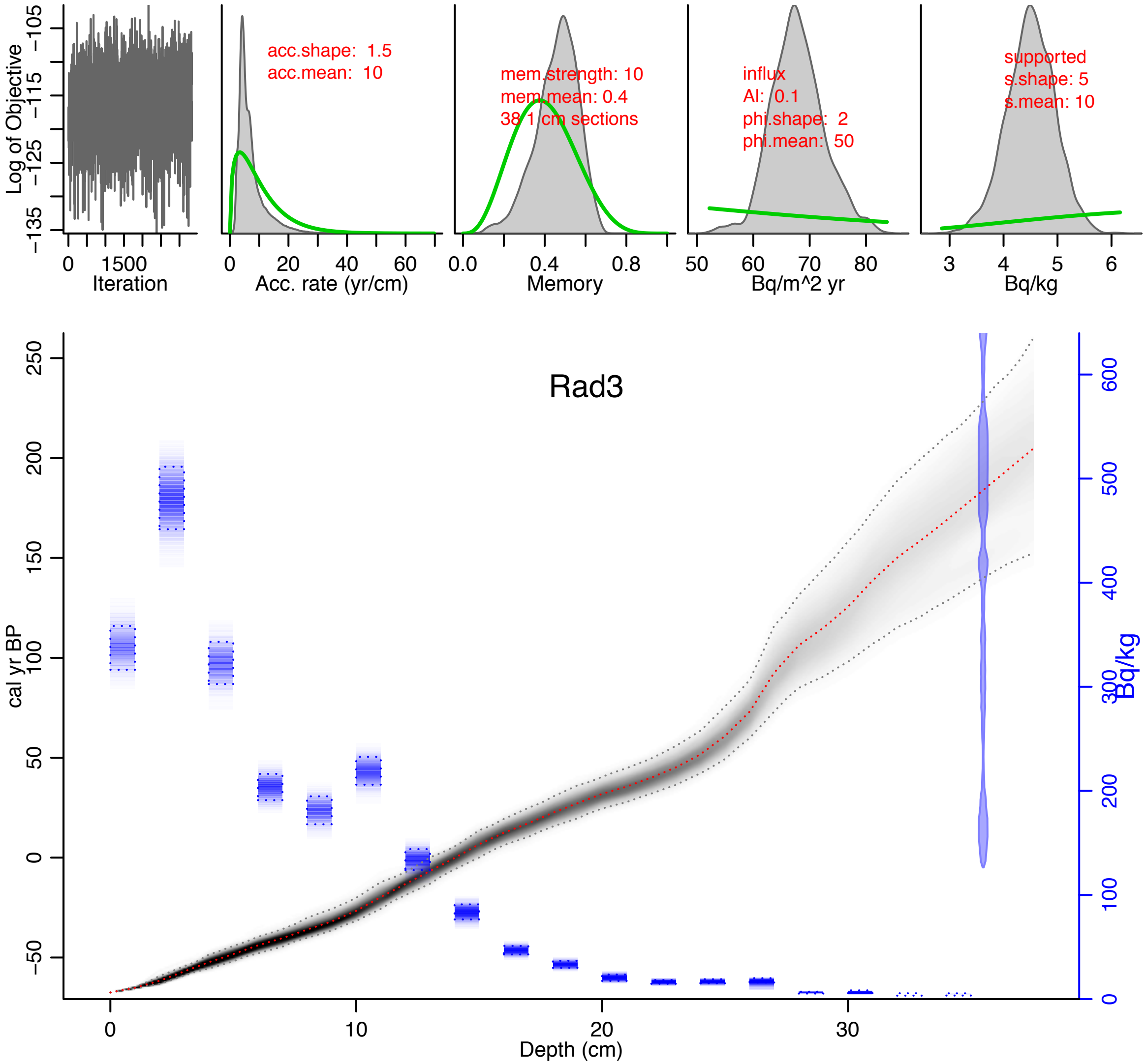

Log of Objective
−105
−115
−125
−135
0
1500
Iteration
acc.shape: 1.5
acc.mean: 10
0
20
40
60
Acc. rate (yr/cm)
mem.strength: 10
mem.mean: 0.4
38 1 cm sections
0.0
0.4
0.8
Memory
influx
Al: 0.1
phi.shape: 2
phi.mean: 50
50
60
70
80
Bq/m^2 yr
supported
s.shape: 5
s.mean: 10
3
4
5
6
Bq/kg
Rad3
cal yr BP
250
200
150
100
50
0
−50
Depth (cm)
0
10
20
30
Bq/kg
600
500
400
300
200
100
0

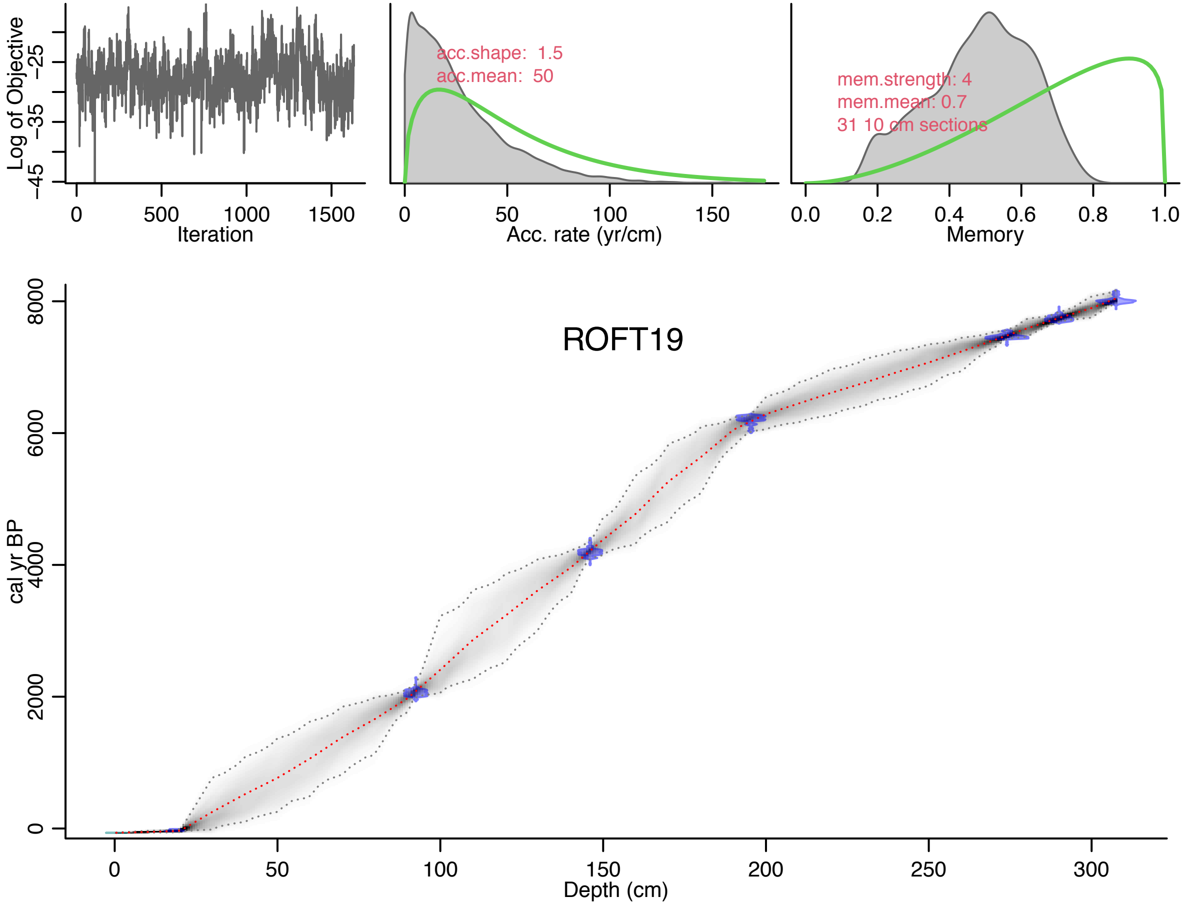

Log of Objective
−45
−35
−25
0
500
1000
1500
Iteration
acc.shape: 1.5
acc.mean: 50
0
50
100
150
Acc. rate (yr/cm)
mem.strength: 4
mem.mean: 0.7
31 10 cm sections
0.0
0.2
0.4
0.6
0.8
1.0
Memory
ROFT19
cal yr BP
0
2000
4000
6000
8000
0
50
100
150
200
250
300
Depth (cm)

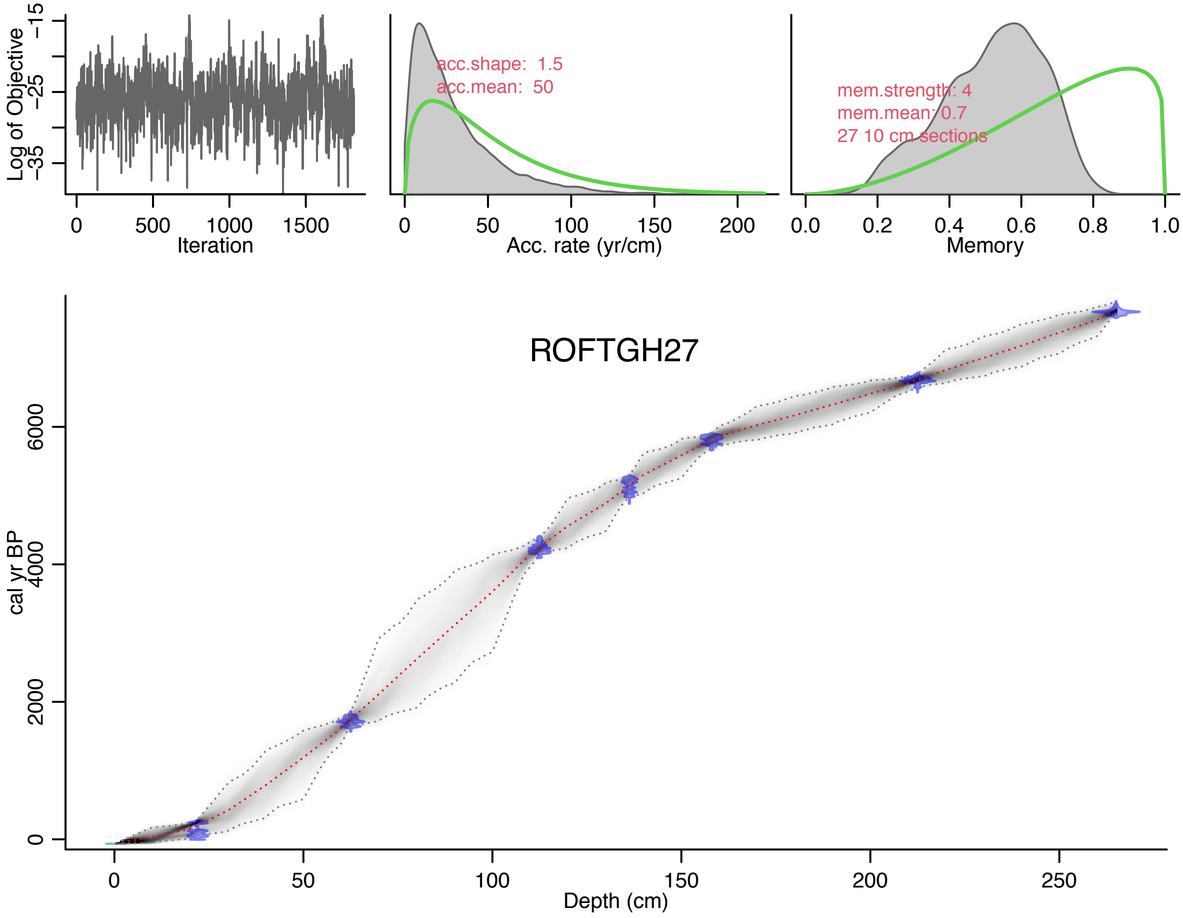

Log of Objective
−15
−25
−35
0
500
1000
1500
Iteration
acc.shape: 1.5
acc.mean: 50
0
50
100
150
200
Acc. rate (yr/cm)
mem.strength: 4
mem.mean: 0.7
27 10 cm sections
0.0
0.2
0.4
0.6
0.8
1.0
Memory
ROFTGH27
cal yr BP
0
2000
4000
6000
0
50
100
150
200
250
Depth (cm)

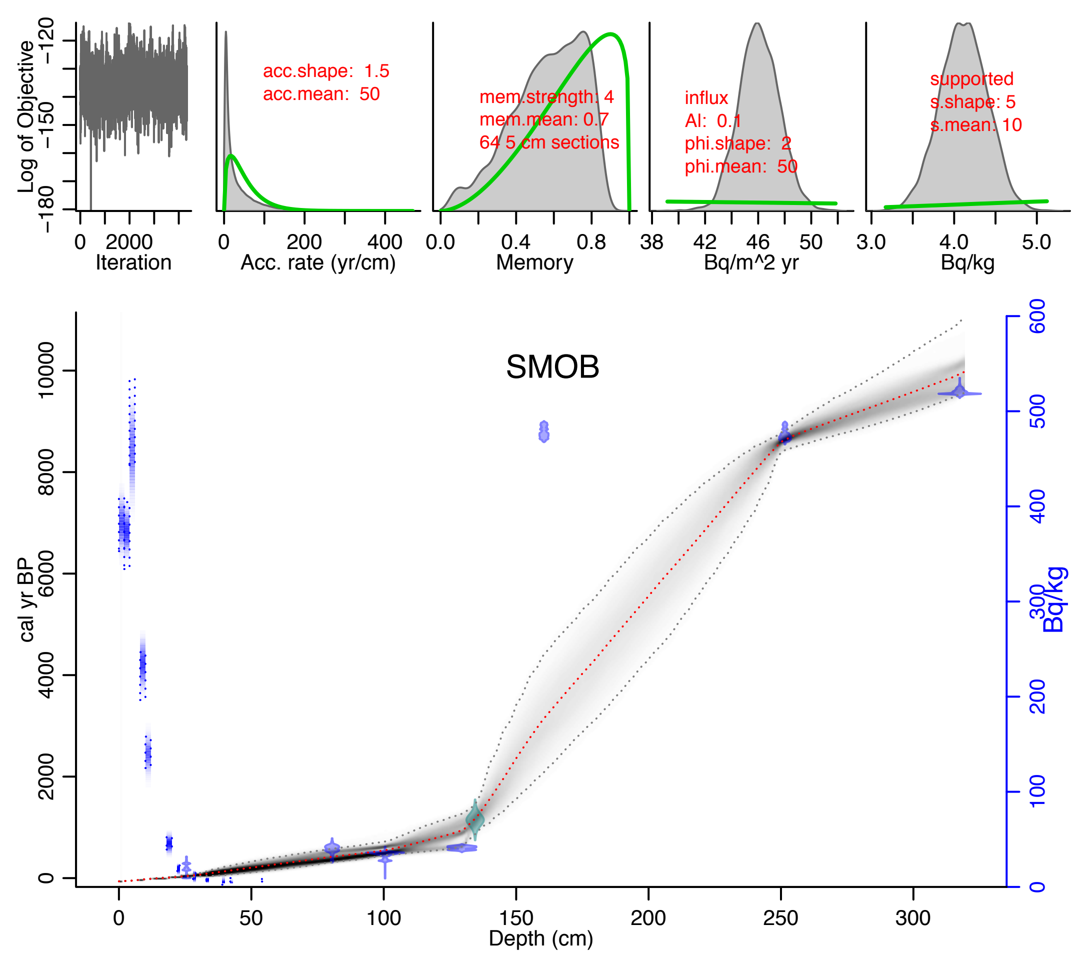

Log of Objective
Iteration
acc.shape: 1.5
acc.mean: 50
Acc. rate (yr/cm)
mem.strength: 4
mem.mean: 0.7
64 5 cm sections
Memory
influx
Al: 0.1
phi.shape: 2
phi.mean: 50
Bq/m^2 yr
supported
s.shape: 5
s.mean: 10
Bq/kg
SMOB
cal yr BP
Bq/kg
Depth (cm)

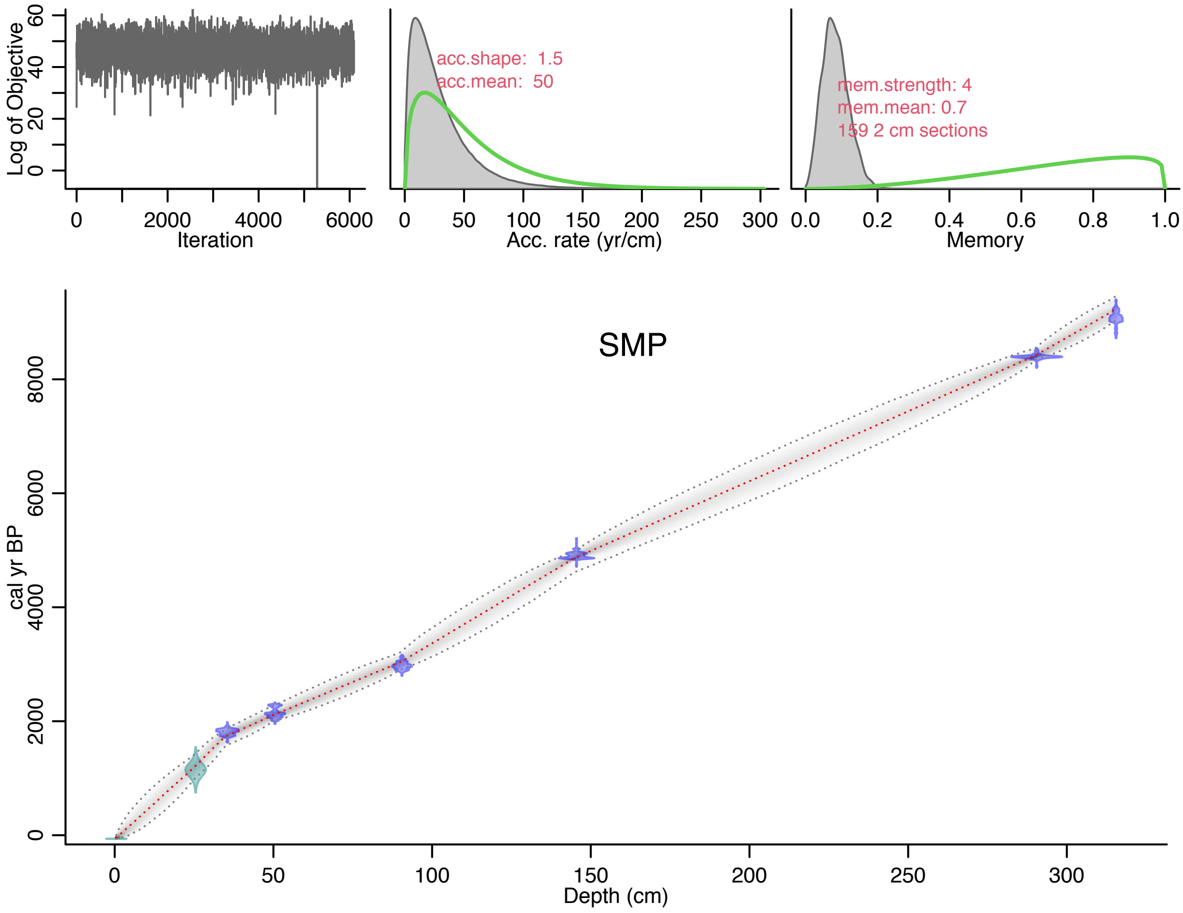
Log of Objective
Iteration
acc.shape: 1.5
acc.mean: 50
Acc. rate (yr/cm)
mem.strength: 4
mem.mean: 0.7
159 2 cm sections
Memory
SMP
cal yr BP
Depth (cm)

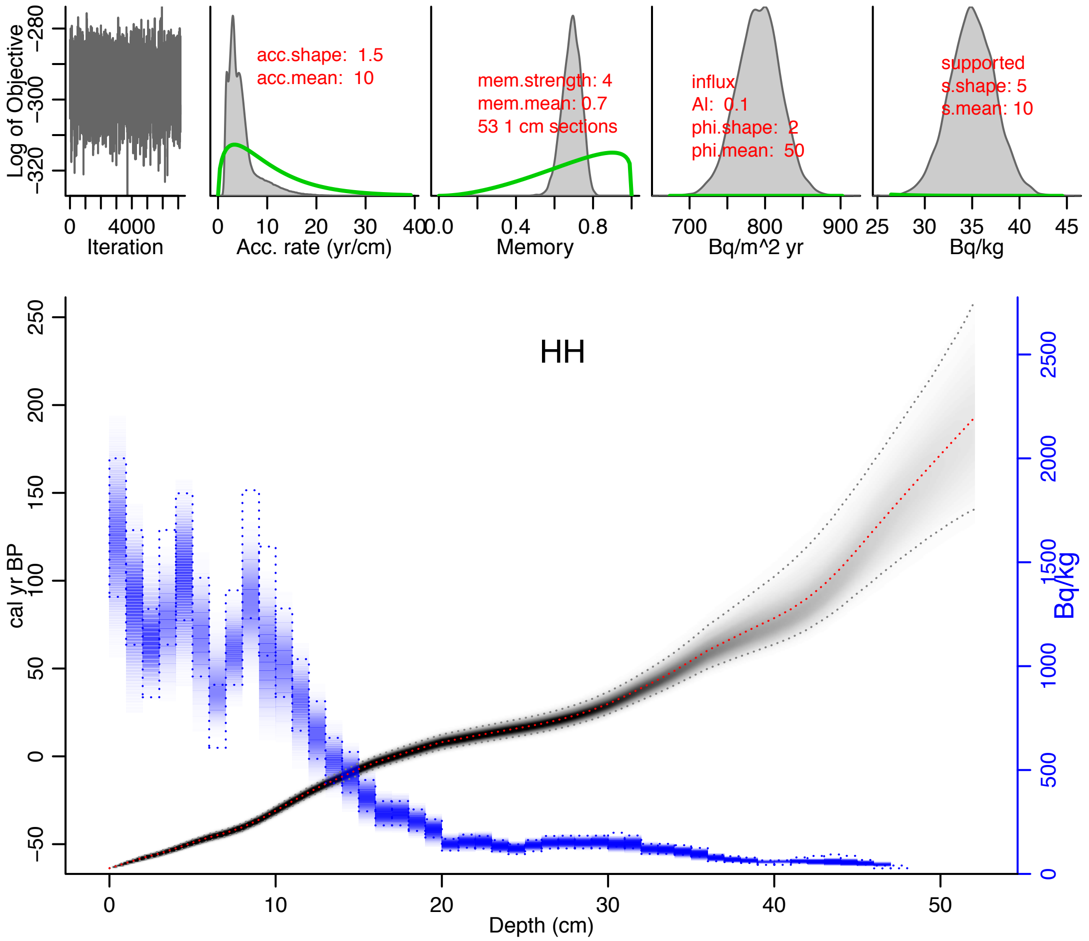

Log of Objective
−280
−300
−320
0
4000
Iteration
acc.shape: 1.5
acc.mean: 10
0
10
20
30
40
Acc. rate (yr/cm)
mem.strength: 4
mem.mean: 0.7
53 1 cm sections
0.0
0.4
0.8
Memory
influx
Al: 0.1
phi.shape: 2
phi.mean: 50
700
800
900
Bq/m^2 yr
supported
s.shape: 5
s.mean: 10
25
30
35
40
45
Bq/kg
HH
cal yr BP
250
200
150
100
50
0
−50
Bq/kg
2500
2000
1500
1000
500
0
0
10
20
30
40
50
Depth (cm)

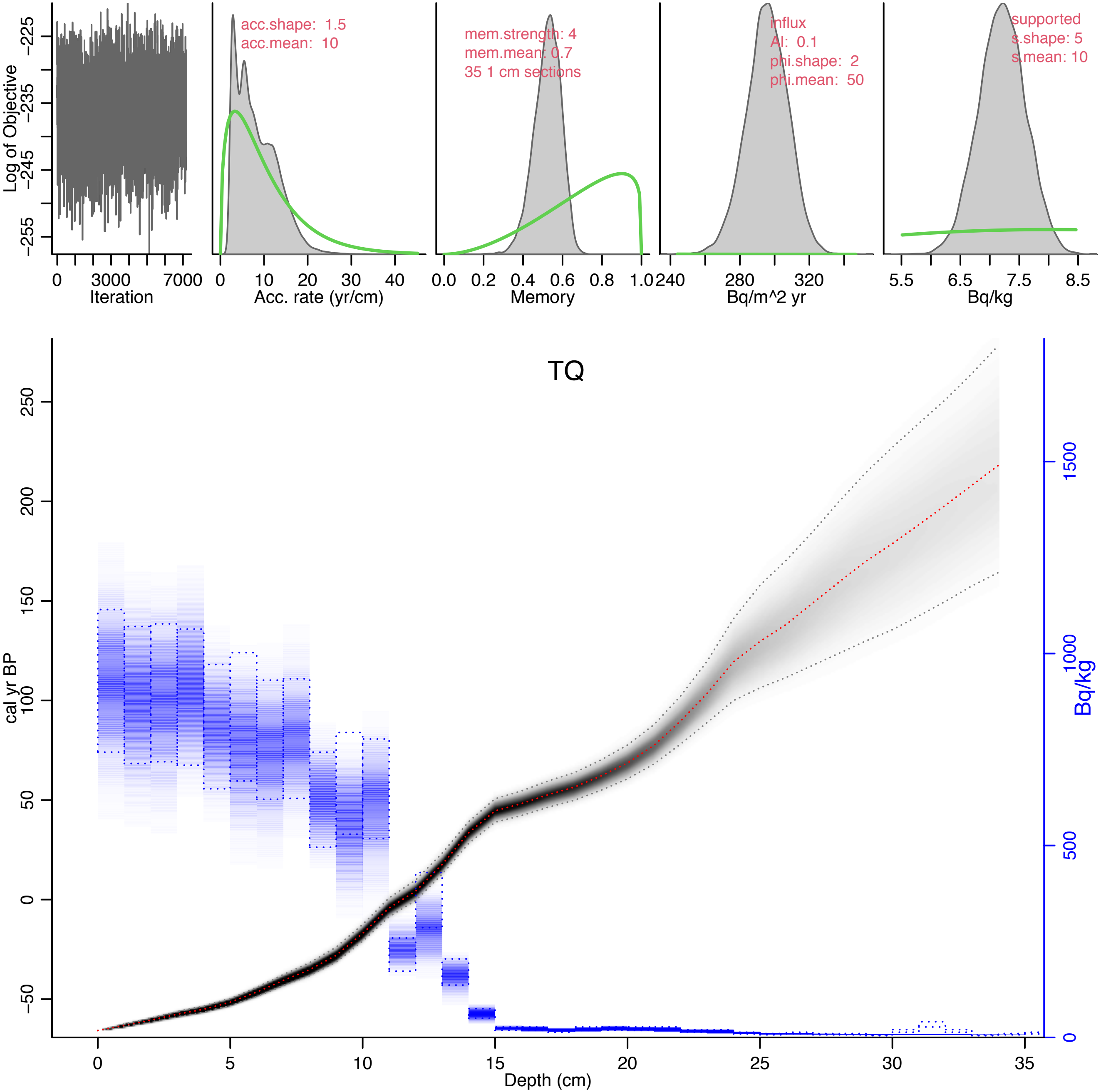

Log of Objective
Iteration
acc.shape: 1.5
acc.mean: 10
Acc. rate (yr/cm)
mem.strength: 4
mem.mean: 0.7
35 1 cm sections
Memory
influx
Al: 0.1
phi.shape: 2
phi.mean: 50
Bq/m^2 yr
supported
s.shape: 5
s.mean: 10
Bq/kg
TQ
cal yr BP
Bq/kg
Depth (cm)

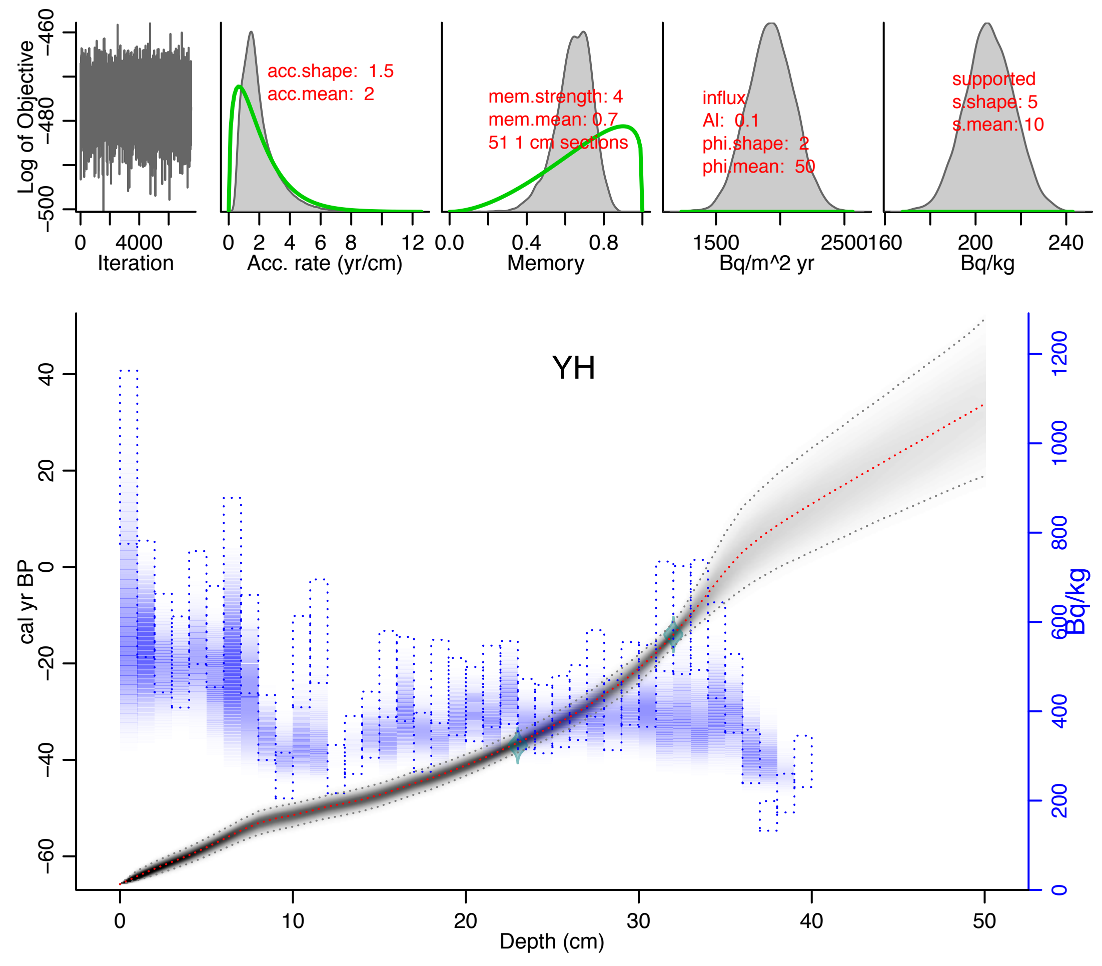

Log of Objective
Iteration
acc.shape: 1.5
acc.mean: 2
Acc. rate (yr/cm)
mem.strength: 4
mem.mean: 0.7
51 1 cm sections
Memory
influx
Al: 0.1
phi.shape: 2
phi.mean: 50
Bq/m^2 yr
supported
s.shape: 5
s.mean: 10
Bq/kg
YH
cal yr BP
Bq/kg
Depth (cm)

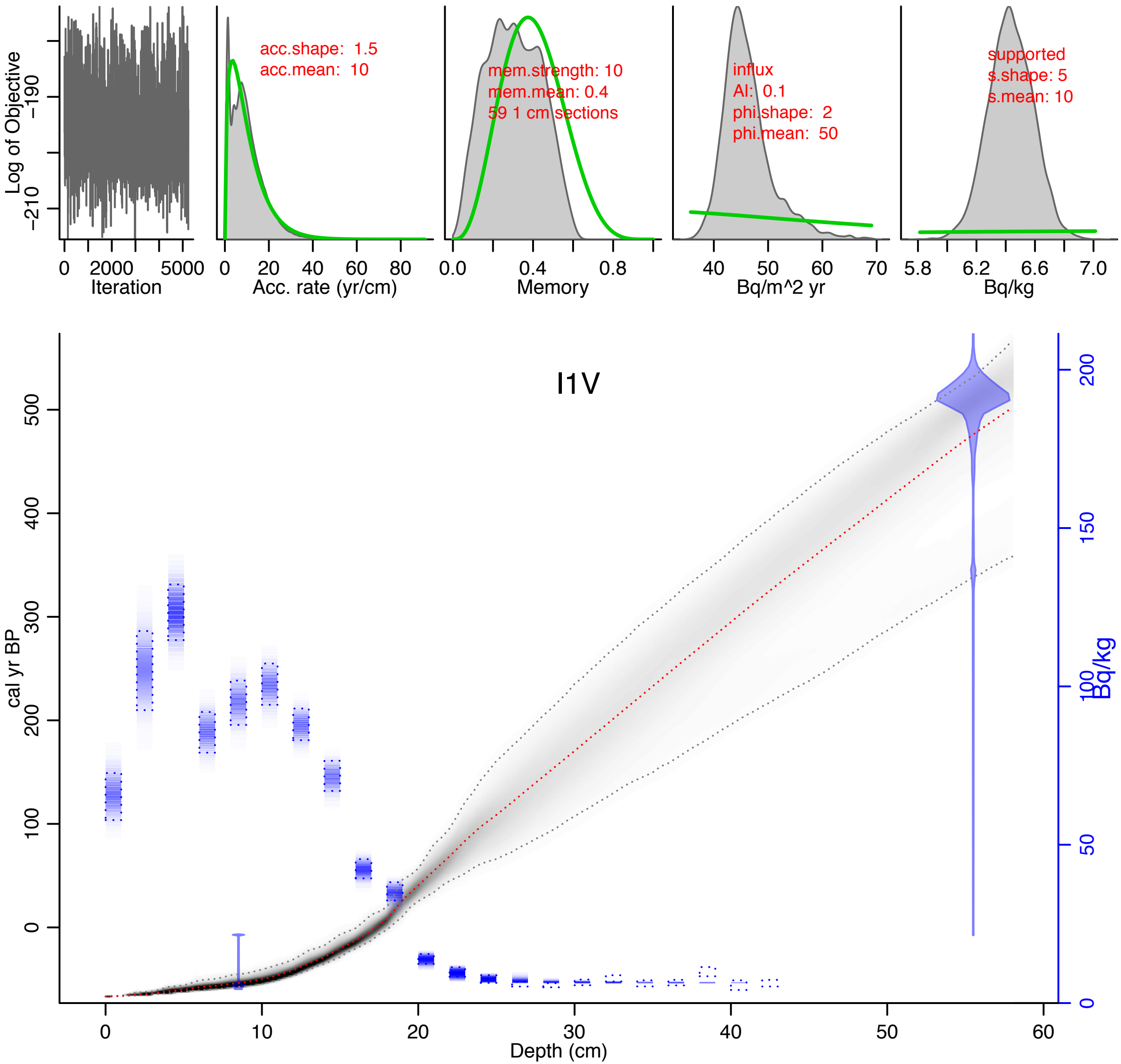

Log of Objective
−190
−210
0 2000 5000
Iteration
acc.shape: 1.5
acc.mean: 10
0 20 40 60 80
Acc. rate (yr/cm)
mem.strength: 10
mem.mean: 0.4
59 1 cm sections
0.0 0.4 0.8
Memory
influx
Al: 0.1
phi.shape: 2
phi.mean: 50
40 50 60 70
Bq/m^2 yr
supported
s.shape: 5
s.mean: 10
5.8 6.2 6.6 7.0
Bq/kg
I1V
cal yr BP
0 100 200 300 400 500
Depth (cm)
0 10 20 30 40 50 60
Bq/kg
0 50 100 150 200

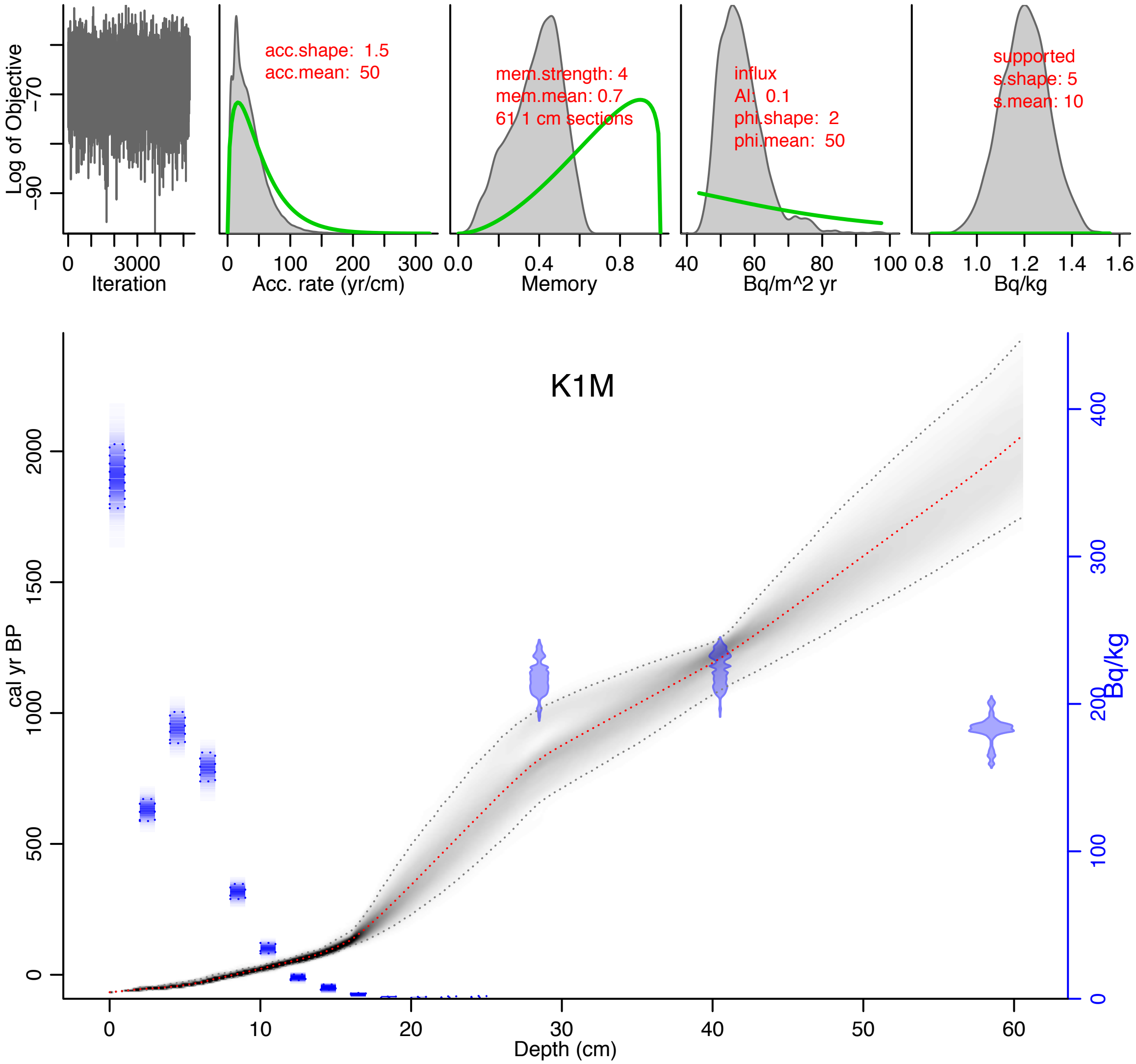

Log of Objective
−70
−90
0
3000
Iteration
acc.shape: 1.5
acc.mean: 50
0
100
200
300
Acc. rate (yr/cm)
mem.strength: 4
mem.mean: 0.7
61 1 cm sections
0.0
0.4
0.8
Memory
influx
Al: 0.1
phi.shape: 2
phi.mean: 50
40
60
80
100
Bq/m^2 yr
supported
s.shape: 5
s.mean: 10
0.8
1.0
1.2
1.4
1.6
Bq/kg
K1M
cal yr BP
0
500
1000
1500
2000
Bq/kg
0
100
200
300
400
0
10
20
30
40
50
60
Depth (cm)

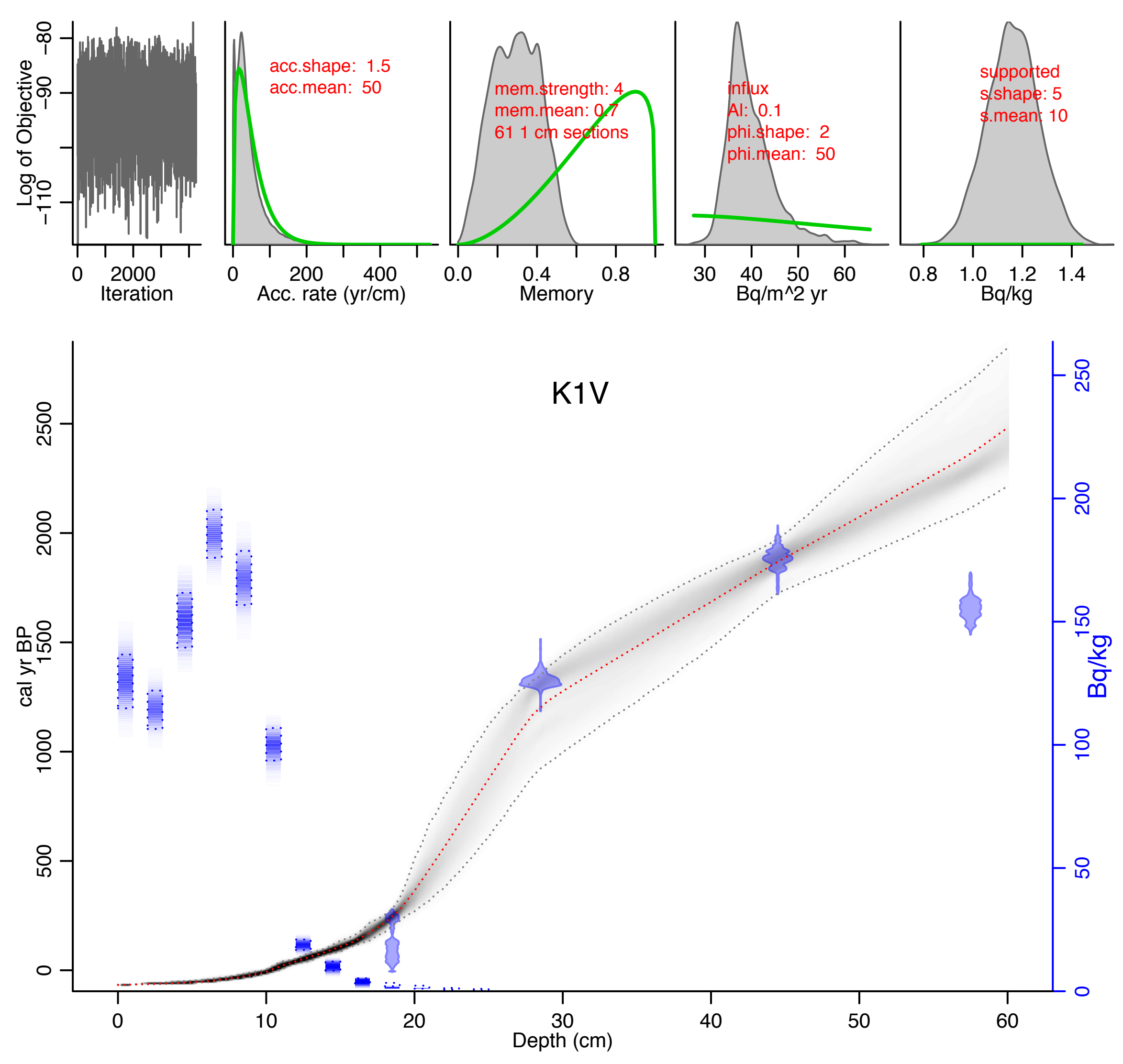

Log of Objective
−80
−90
−110
0
2000
Iteration
acc.shape: 1.5
acc.mean: 50
0
200
400
Acc. rate (yr/cm)
mem.strength: 4
mem.mean: 0.7
61 1 cm sections
0.0
0.4
0.8
Memory
influx
Al: 0.1
phi.shape: 2
phi.mean: 50
30
40
50
60
Bq/m^2 yr
supported
s.shape: 5
s.mean: 10
0.8
1.0
1.2
1.4
Bq/kg
K1V
cal yr BP
0
500
1000
1500
2000
2500
Depth (cm)
0
10
20
30
40
50
60
Bq/kg
0
50
100
150
200
250

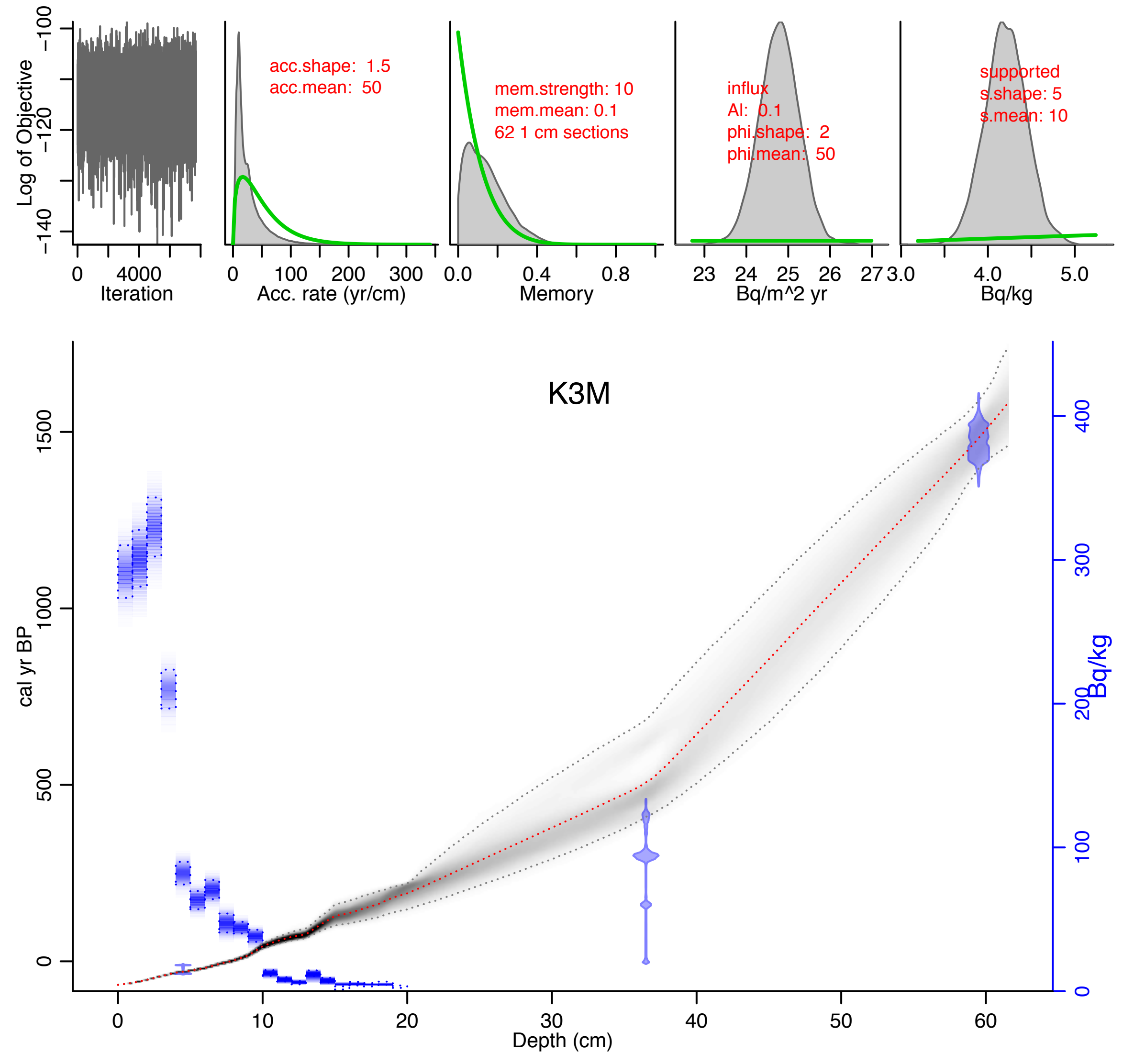

Log of Objective
Iteration
acc.shape: 1.5
acc.mean: 50
Acc. rate (yr/cm)
mem.strength: 10
mem.mean: 0.1
62 1 cm sections
Memory
influx
Al: 0.1
phi.shape: 2
phi.mean: 50
Bq/m^2 yr
supported
s.shape: 5
s.mean: 10
Bq/kg
K3M
cal yr BP
Bq/kg
Depth (cm)

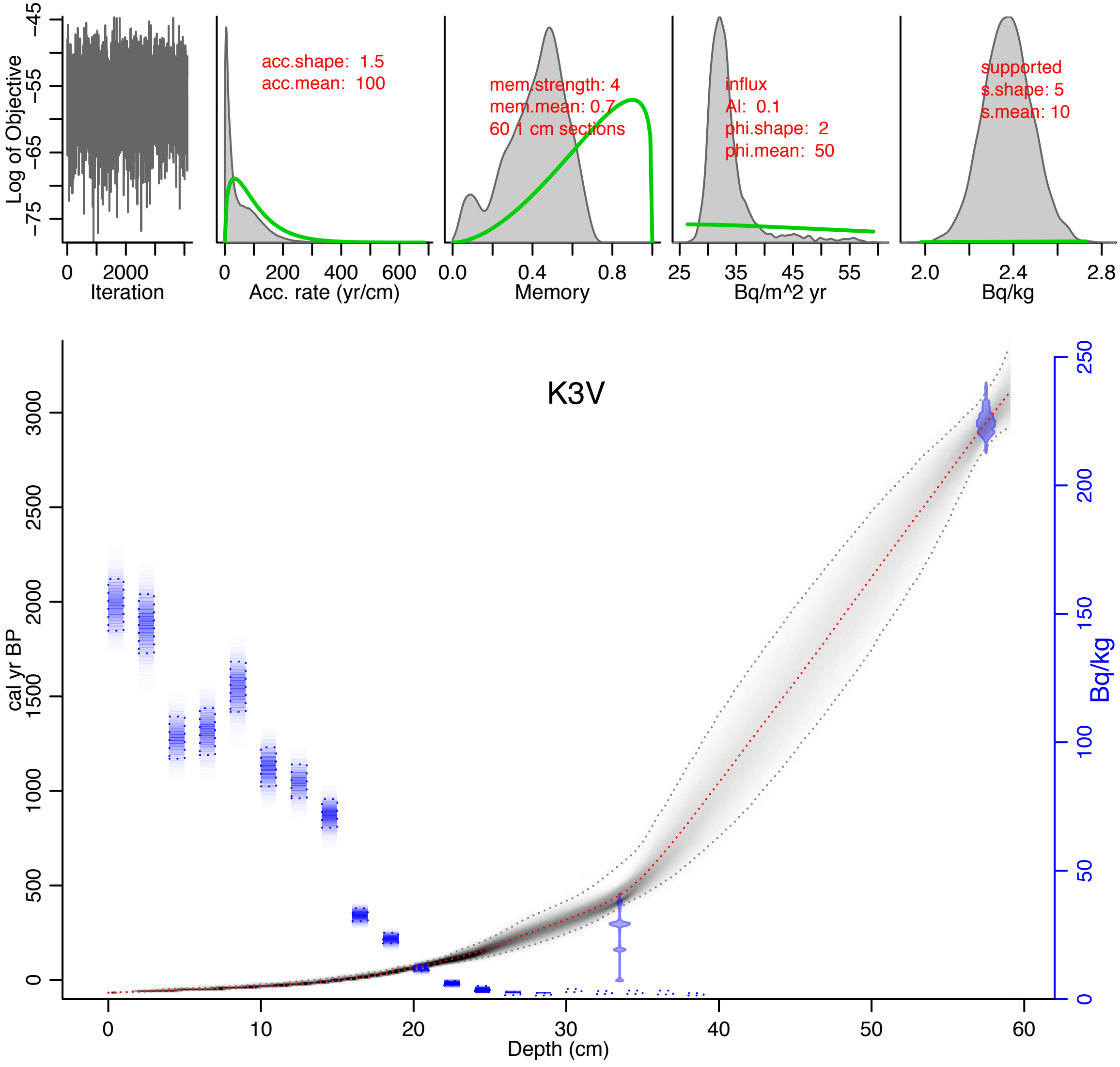
Log of Objective
Iteration
acc.shape: 1.5
acc.mean: 100
Acc. rate (yr/cm)
mem.strength: 4
mem.mean: 0.7
60 1 cm sections
Memory
influx
Al: 0.1
phi.shape: 2
phi.mean: 50
Bq/m^2 yr
supported
s.shape: 5
s.mean: 10
Bq/kg
K3V
cal yr BP
Bq/kg
Depth (cm)

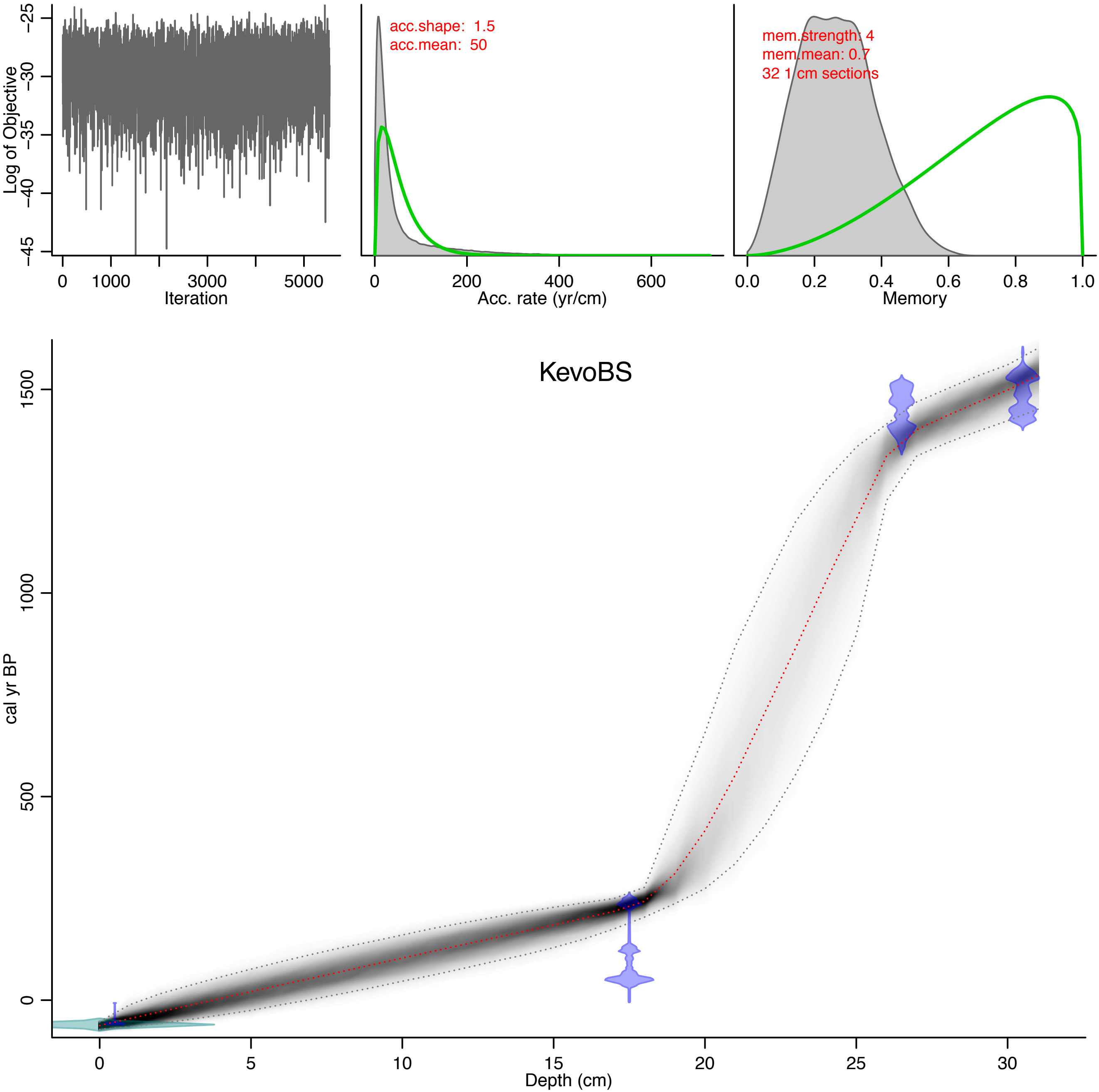

Log of Objective
−25
−30
−35
−40
−45
0
1000
3000
5000
Iteration
acc.shape: 1.5
acc.mean: 50
0
200
400
600
Acc. rate (yr/cm)
mem.strength: 4
mem.mean: 0.7
32 1 cm sections
0.0
0.2
0.4
0.6
0.8
1.0
Memory
KevoBS
cal yr BP
1500
1000
500
0
0
5
10
15
20
25
30
Depth (cm)

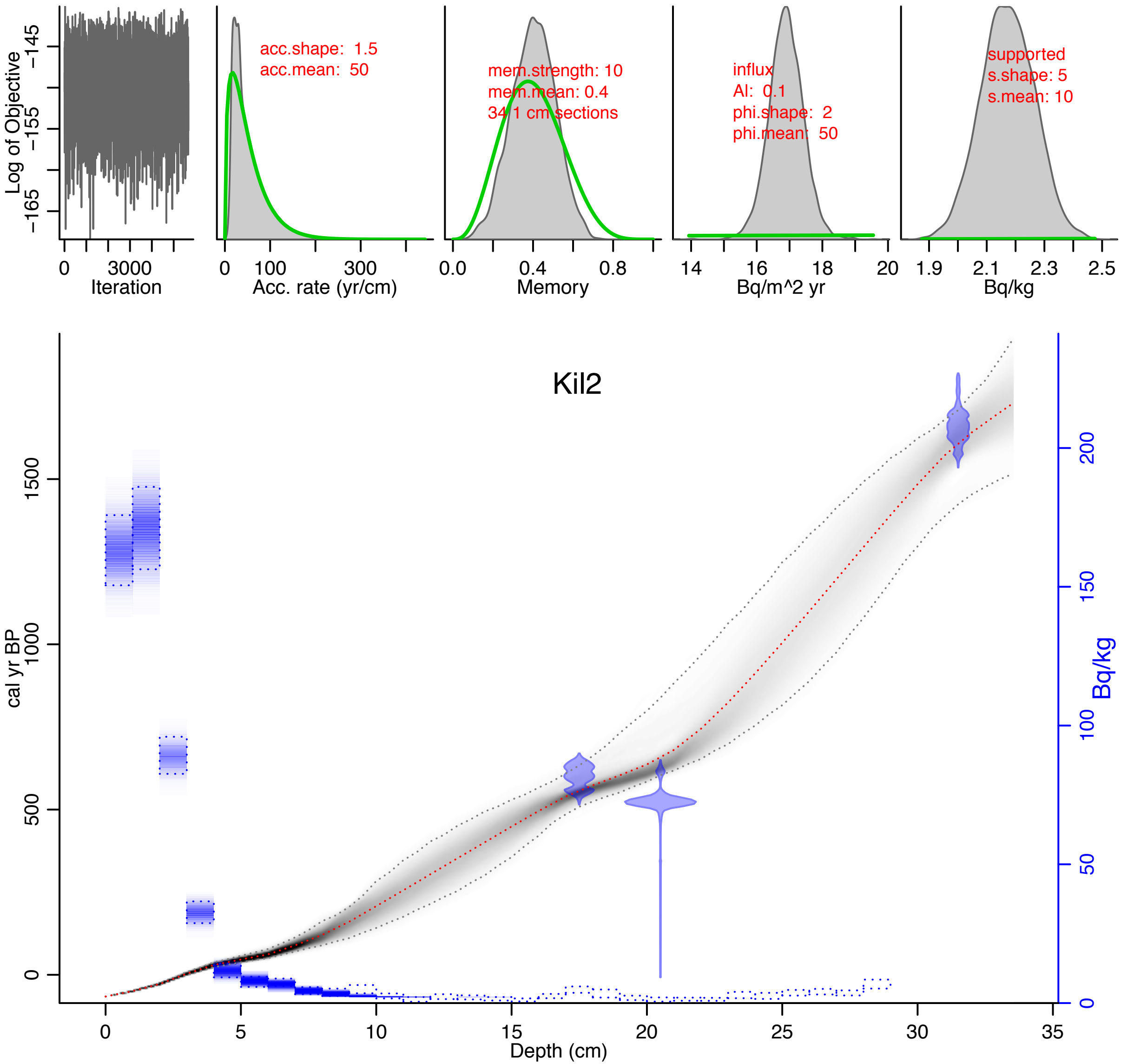

Log of Objective
−145
−155
−165
0
3000
Iteration
acc.shape: 1.5
acc.mean: 50
0
100
300
Acc. rate (yr/cm)
mem.strength: 10
mem.mean: 0.4
34 1 cm sections
0.0
0.4
0.8
Memory
influx
Al: 0.1
phi.shape: 2
phi.mean: 50
14
16
18
20
Bq/m^2 yr
supported
s.shape: 5
s.mean: 10
1.9
2.1
2.3
2.5
Bq/kg
Kil2
cal yr BP
0
500
1000
1500
Bq/kg
0
50
100
150
200
0
5
10
15
20
25
30
35
Depth (cm)

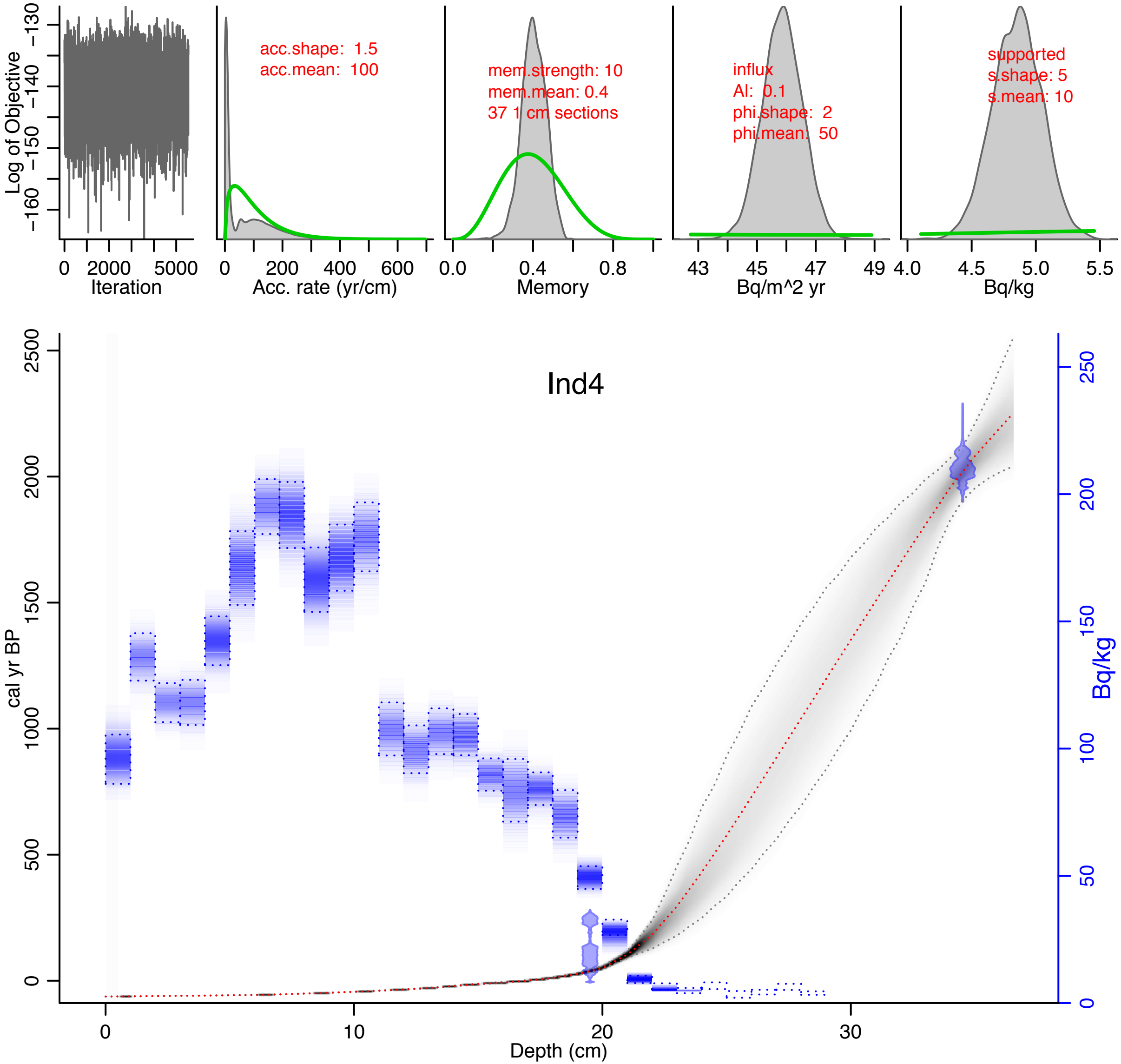
Log of Objective
Iteration
acc.shape: 1.5
acc.mean: 100
Acc. rate (yr/cm)
mem.strength: 10
mem.mean: 0.4
37 1 cm sections
Memory
influx
Al: 0.1
phi.shape: 2
phi.mean: 50
Bq/m^2 yr
supported
s.shape: 5
s.mean: 10
Bq/kg
Ind4
cal yr BP
Bq/kg
Depth (cm)

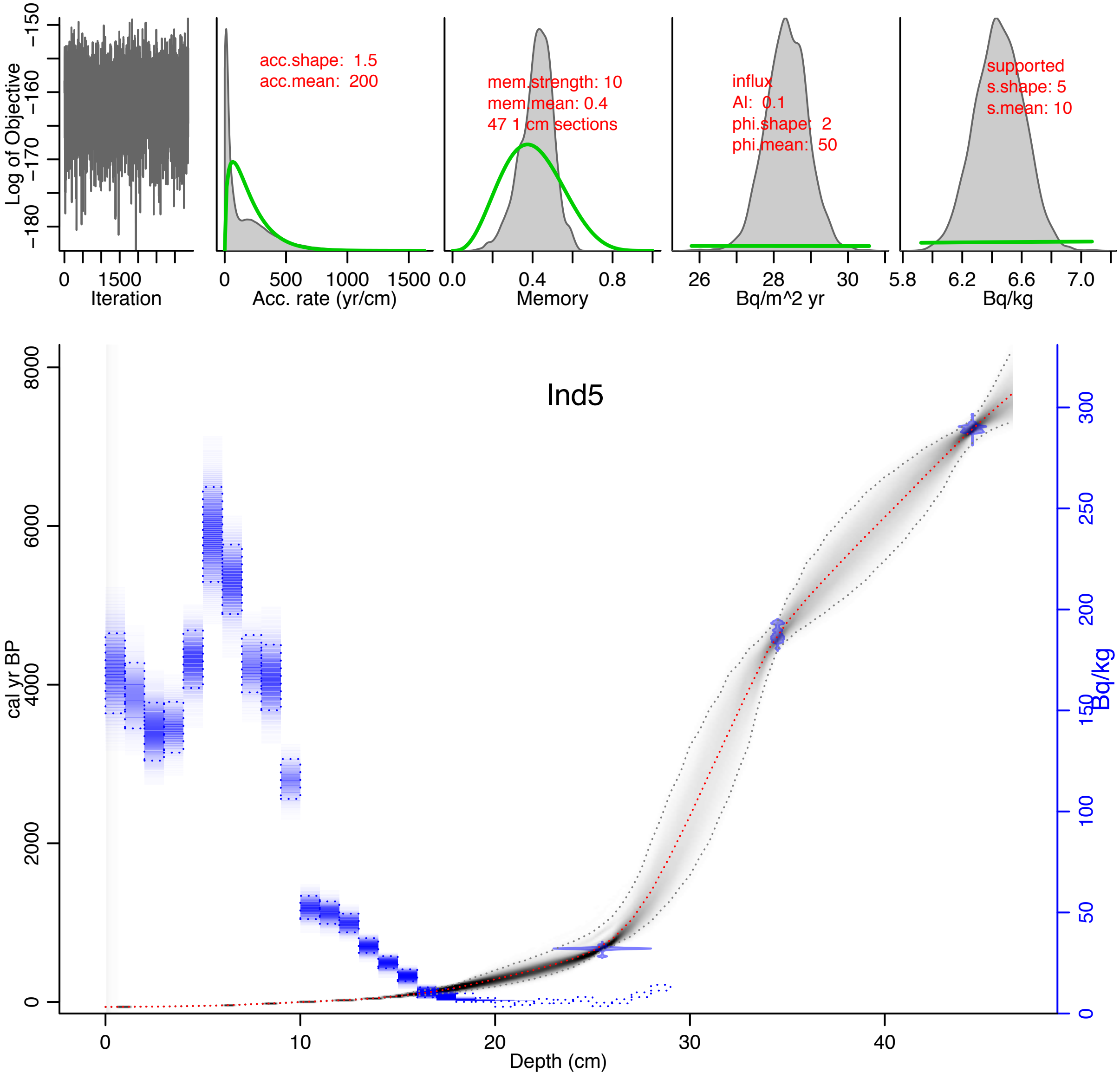

Log of Objective
Iteration
acc.shape: 1.5
acc.mean: 200
Acc. rate (yr/cm)
mem.strength: 10
mem.mean: 0.4
47 1 cm sections
Memory
influx
Al: 0.1
phi.shape: 2
phi.mean: 50
Bq/m^2 yr
supported
s.shape: 5
s.mean: 10
Bq/kg
Ind5
cal yr BP
Bq/kg
Depth (cm)

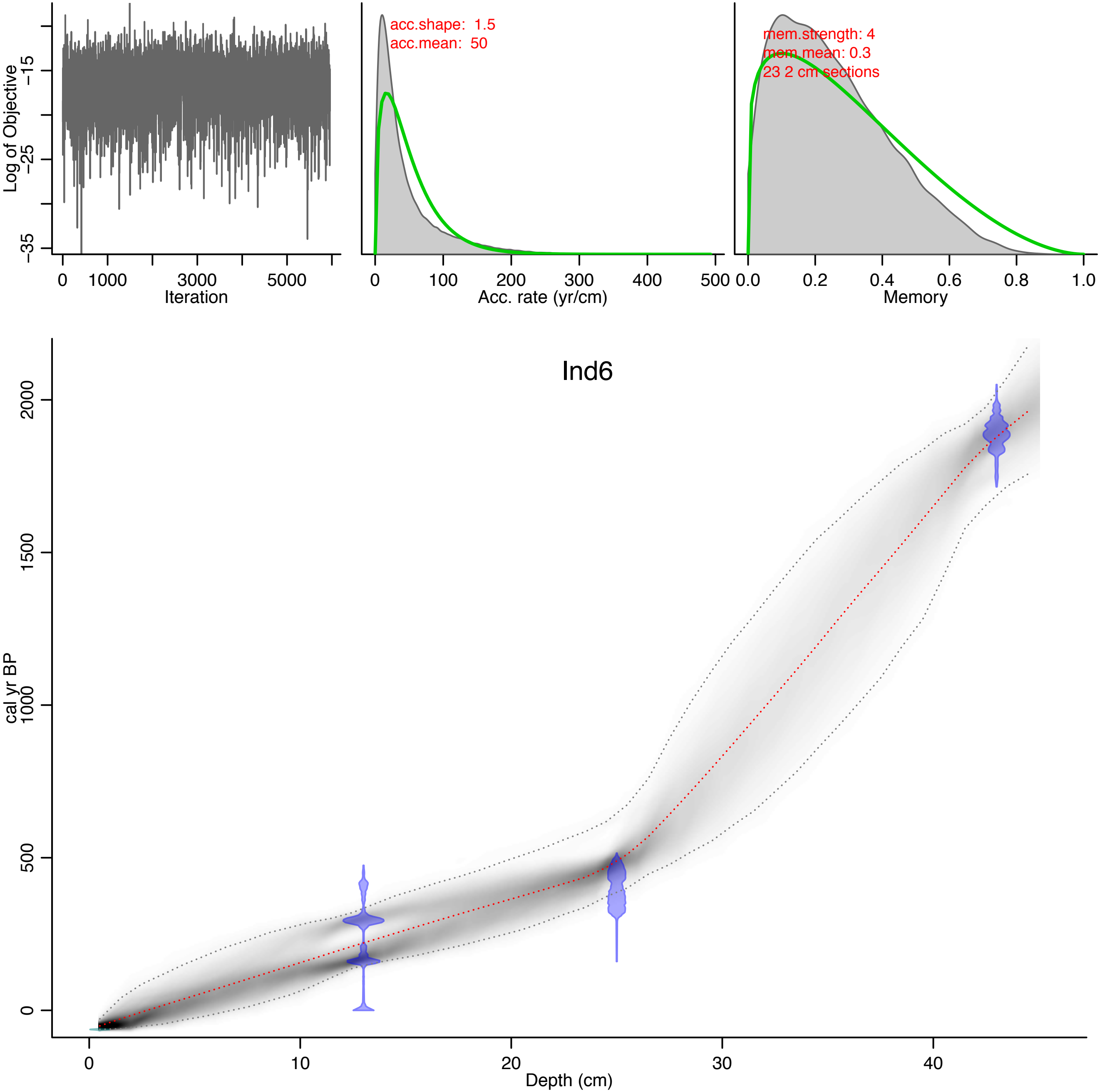

Log of Objective
−15
−25
−35
0
1000
3000
5000
Iteration
acc.shape: 1.5
acc.mean: 50
0
100
200
300
400
500
Acc. rate (yr/cm)
mem.strength: 4
mem.mean: 0.3
23 2 cm sections
0.0
0.2
0.4
0.6
0.8
1.0
Memory
Ind6
cal yr BP
0
500
1000
1500
2000
0
10
20
30
40
Depth (cm)

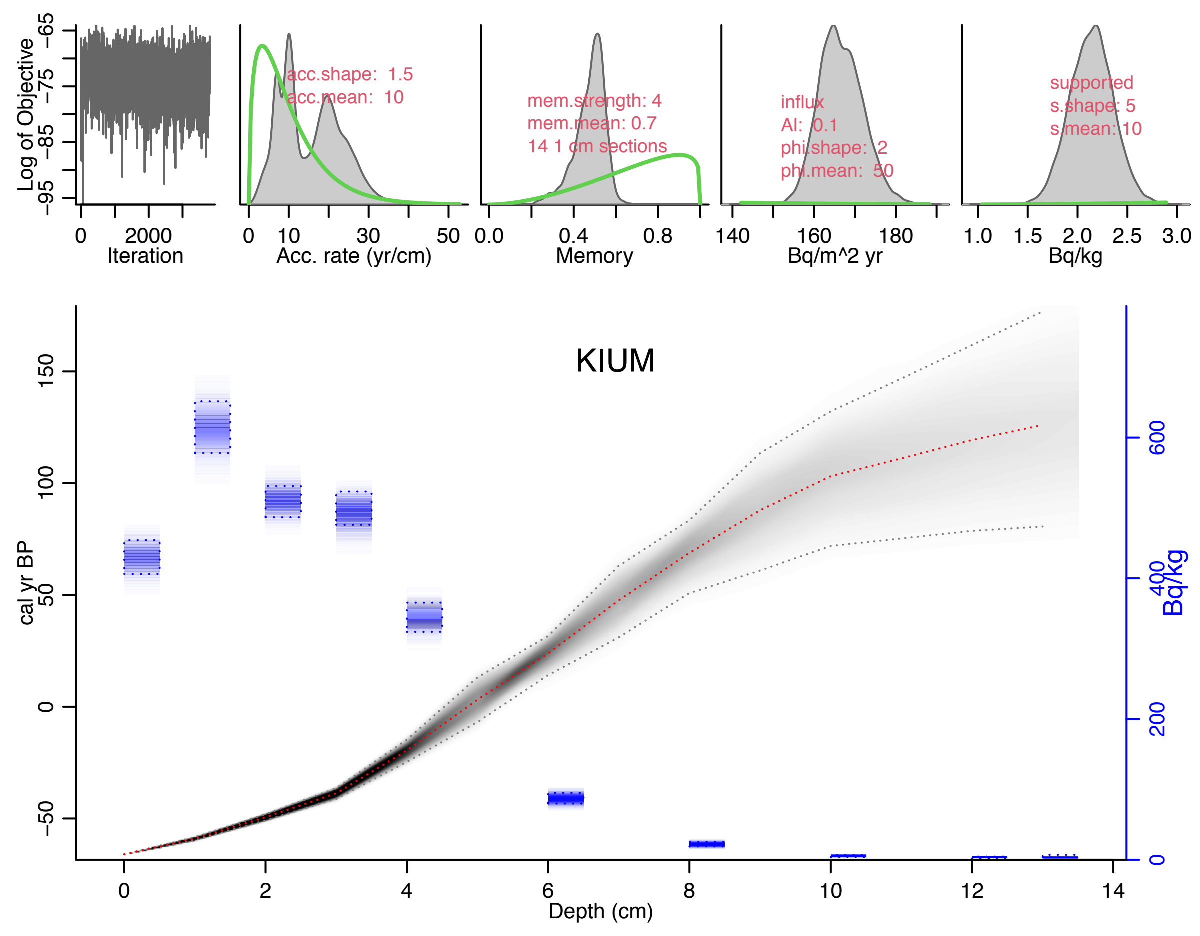

Log of Objective
Iteration
acc.shape: 1.5
acc.mean: 10
Acc. rate (yr/cm)
mem.strength: 4
mem.mean: 0.7
14 1 cm sections
Memory
influx
Al: 0.1
phi.shape: 2
phi.mean: 50
Bq/m^2 yr
supported
s.shape: 5
s.mean: 10
Bq/kg
KIUM
cal yr BP
Depth (cm)
Bq/kg

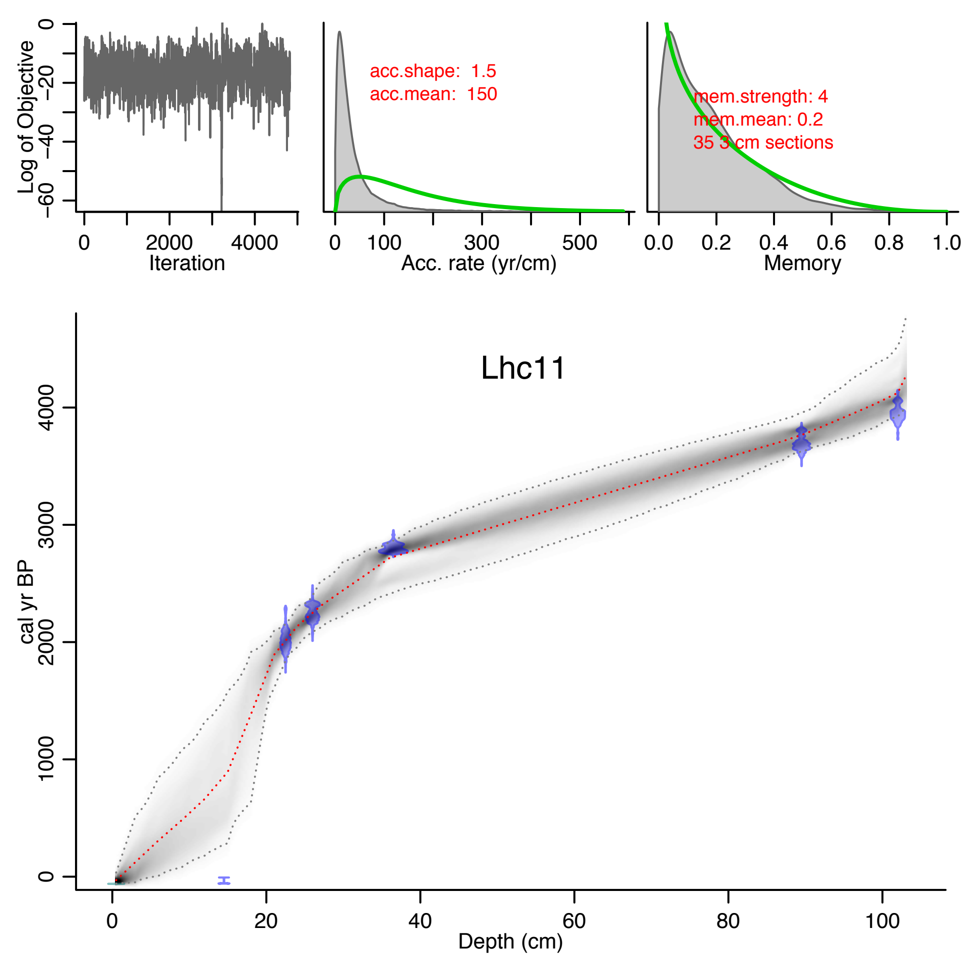

Log of Objective
Iteration
acc.shape: 1.5
acc.mean: 150
Acc. rate (yr/cm)
mem.strength: 4
mem.mean: 0.2
35 3 cm sections
Memory
Lhc11
cal yr BP
Depth (cm)

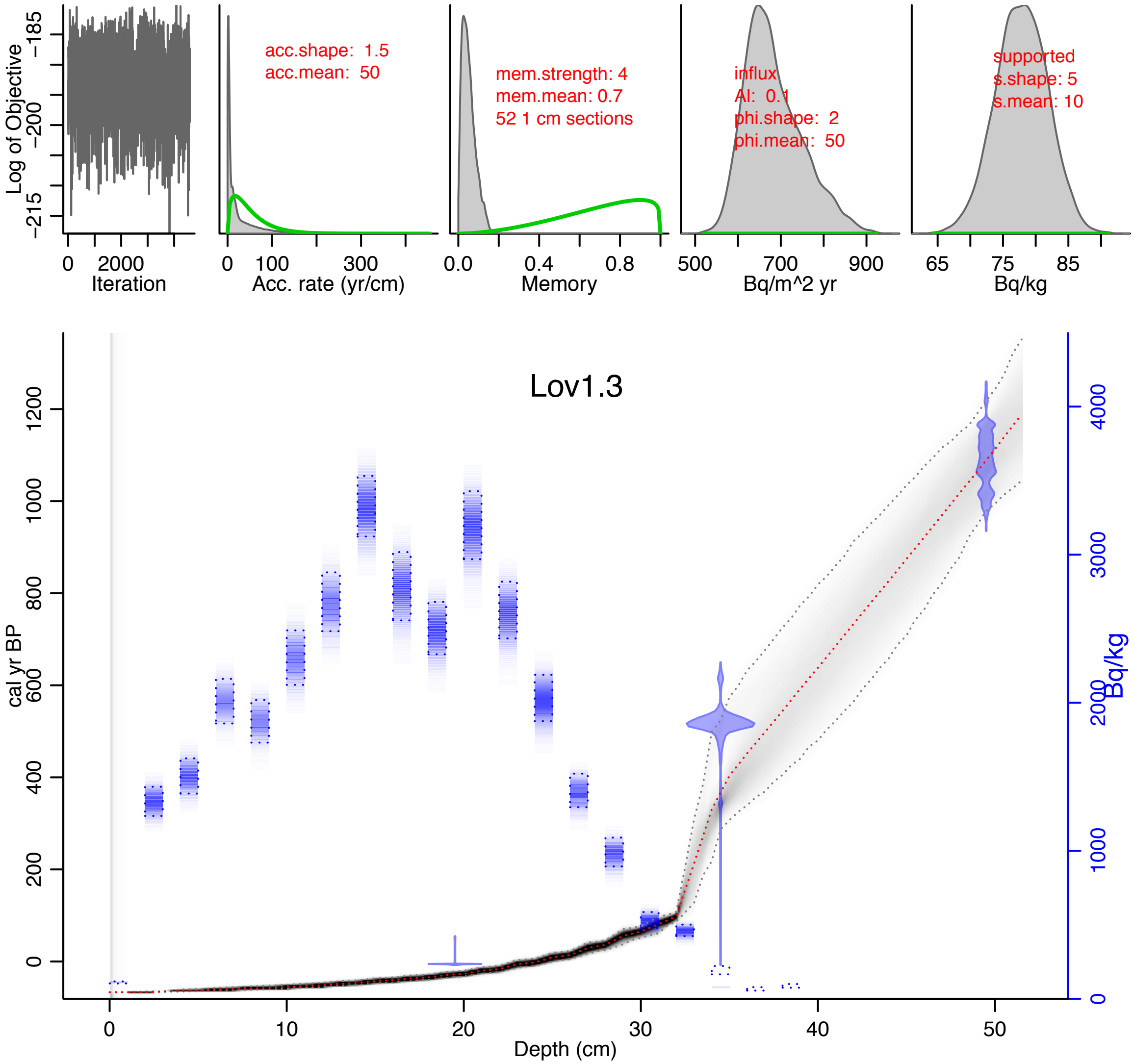

Log of Objective
−185
−200
−215
0
2000
Iteration
acc.shape: 1.5
acc.mean: 50
0
100
300
Acc. rate (yr/cm)
mem.strength: 4
mem.mean: 0.7
52 1 cm sections
0.0
0.4
0.8
Memory
influx
Al: 0.1
phi.shape: 2
phi.mean: 50
500
700
900
Bq/m^2 yr
supported
s.shape: 5
s.mean: 10
65
75
85
Bq/kg
Lov1.3
cal yr BP
0
200
400
600
800
1000
1200
Depth (cm)
0
10
20
30
40
50
Bq/kg
0
1000
2000
3000
4000

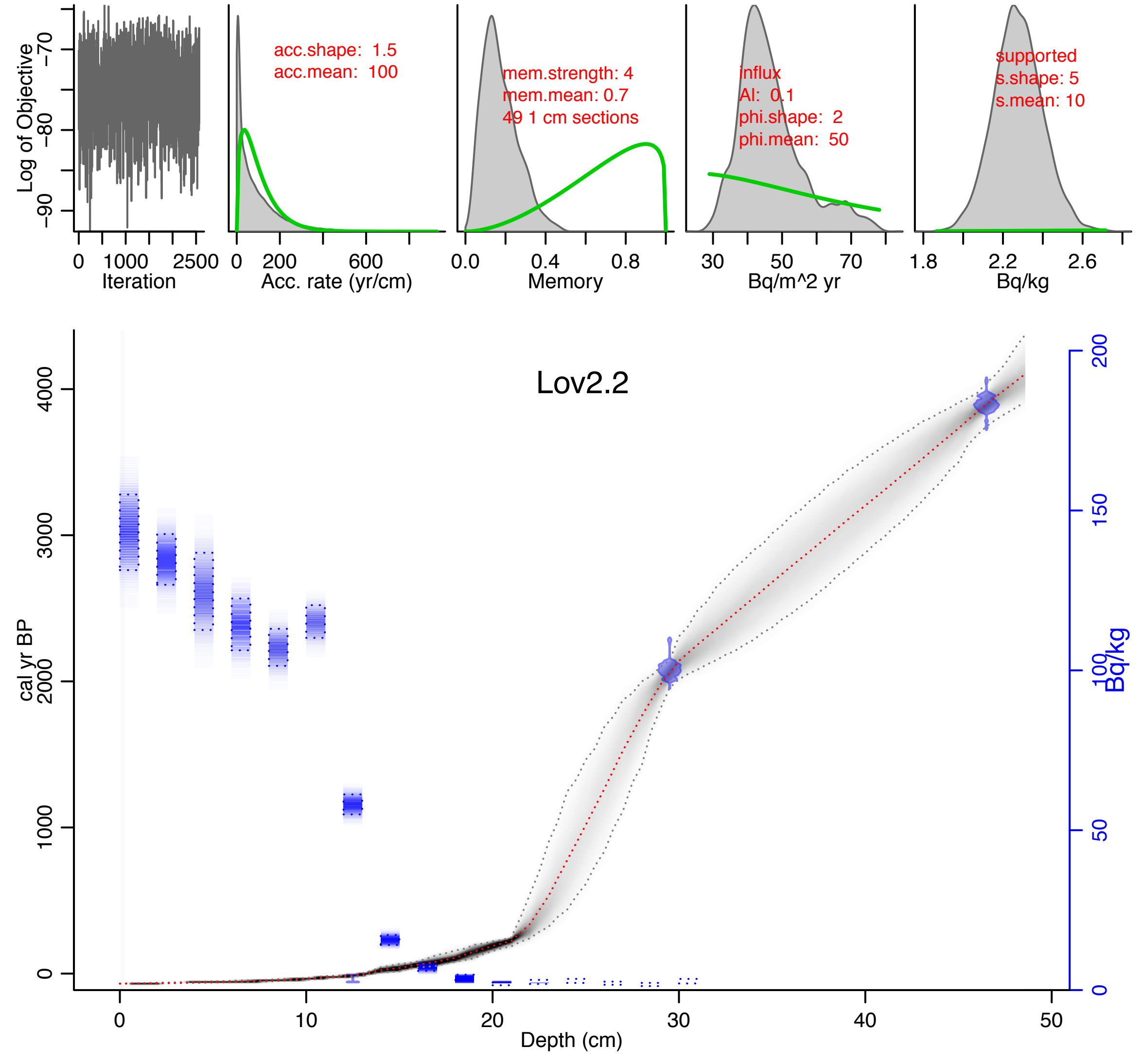

Log of Objective
Iteration
acc.shape: 1.5
acc.mean: 100
Acc. rate (yr/cm)
mem.strength: 4
mem.mean: 0.7
49 1 cm sections
Memory
influx
Al: 0.1
phi.shape: 2
phi.mean: 50
Bq/m^2 yr
supported
s.shape: 5
s.mean: 10
Bq/kg
Lov2.2
cal yr BP
Depth (cm)
Bq/kg

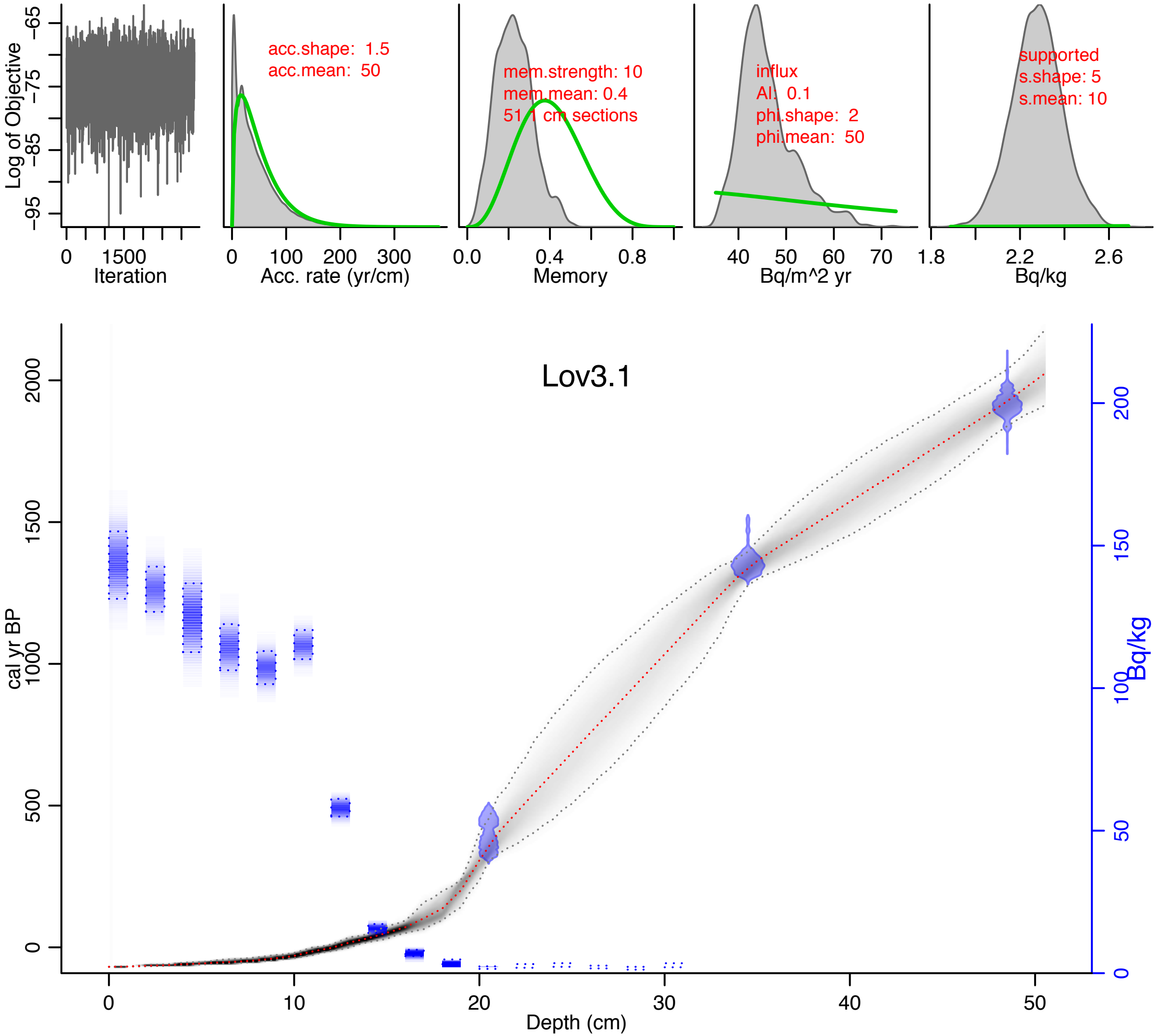
Log of Objective
-65
-75
-85
-95
0
1500
Iteration
acc.shape: 1.5
acc.mean: 50
0
100
200
300
Acc. rate (yr/cm)
mem.strength: 10
mem.mean: 0.4
51 1 cm sections
0.0
0.4
0.8
Memory
influx
Al: 0.1
phi.shape: 2
phi.mean: 50
40
50
60
70
Bq/m^2 yr
supported
s.shape: 5
s.mean: 10
1.8
2.2
2.6
Bq/kg
Lov3.1
cal yr BP
2000
1500
1000
500
0
Bq/kg
200
150
100
50
0
0
10
20
30
40
50
Depth (cm)

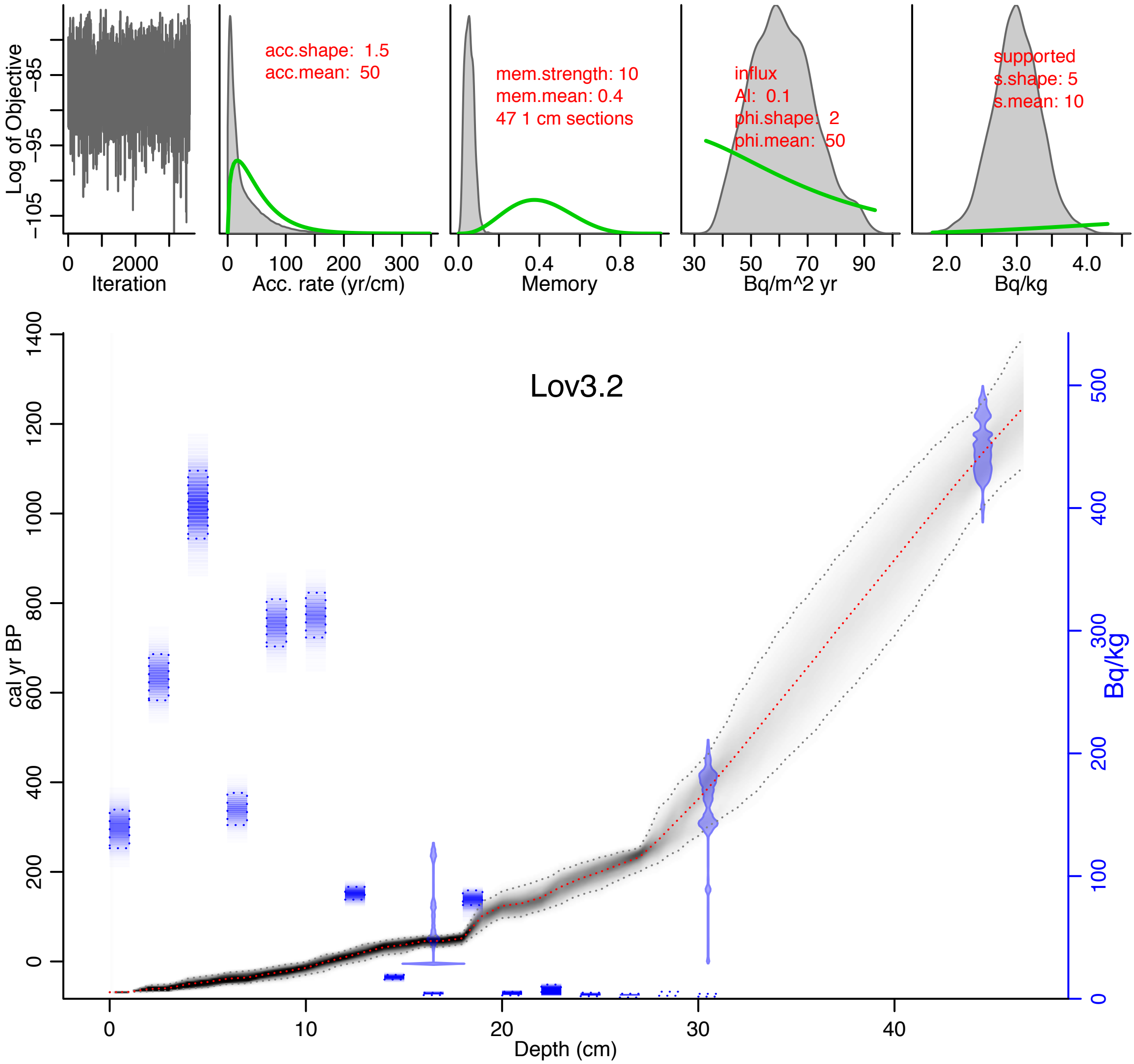

Log of Objective
−85
−95
−105
0
2000
Iteration
acc.shape: 1.5
acc.mean: 50
0
100
200
300
Acc. rate (yr/cm)
mem.strength: 10
mem.mean: 0.4
47 1 cm sections
0.0
0.4
0.8
Memory
influx
Al: 0.1
phi.shape: 2
phi.mean: 50
30
50
70
90
Bq/m^2 yr
supported
s.shape: 5
s.mean: 10
2.0
3.0
4.0
Bq/kg
Lov3.2
cal yr BP
0
200
400
600
800
1000
1200
1400
Bq/kg
0
100
200
300
400
500
0
10
20
30
40
Depth (cm)

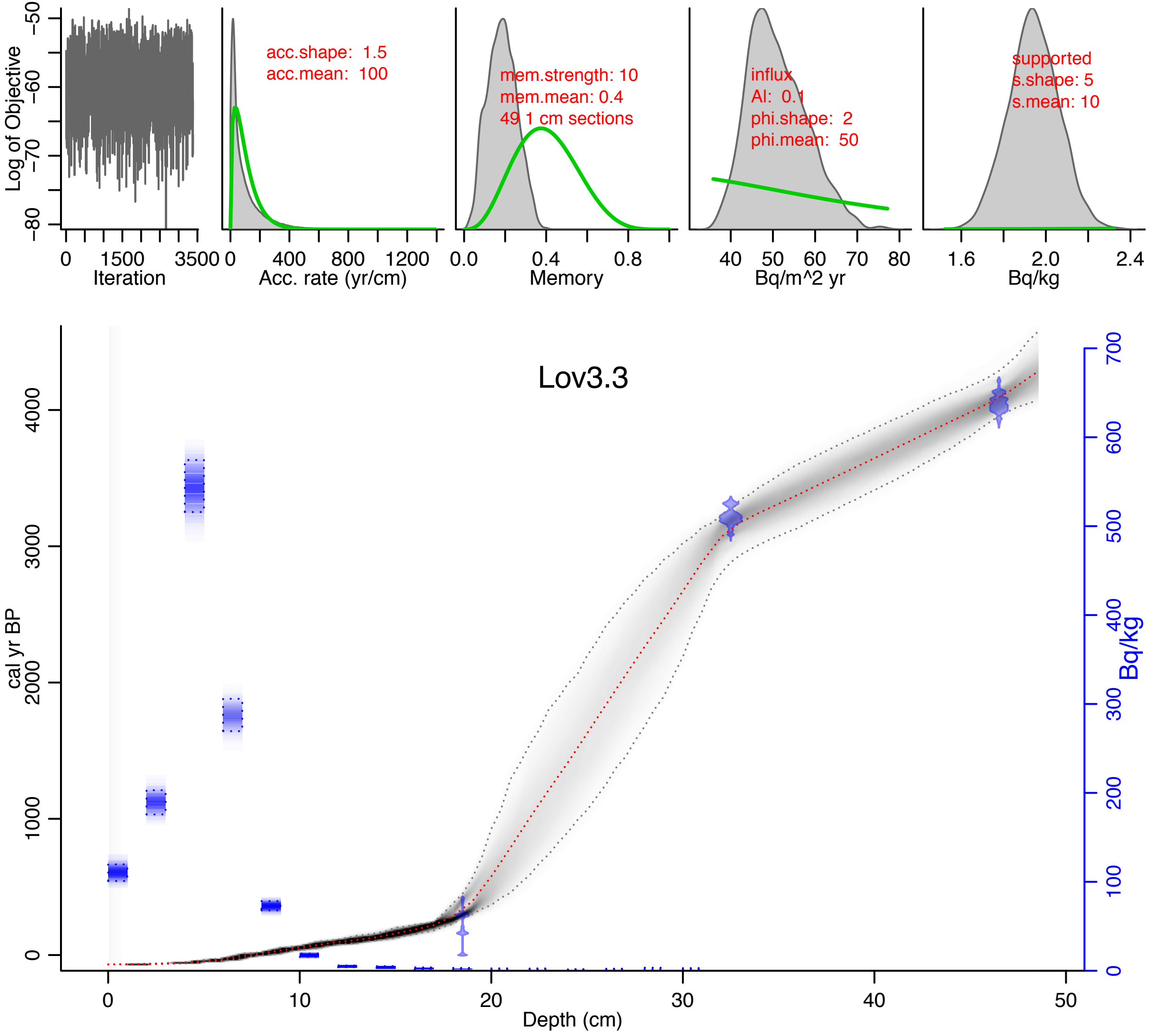
Log of Objective
Iteration
acc.shape: 1.5
acc.mean: 100
Acc. rate (yr/cm)
mem.strength: 10
mem.mean: 0.4
49 1 cm sections
Memory
influx
Al: 0.1
phi.shape: 2
phi.mean: 50
Bq/m^2 yr
supported
s.shape: 5
s.mean: 10
Bq/kg
Lov3.3
cal yr BP
Bq/kg
Depth (cm)

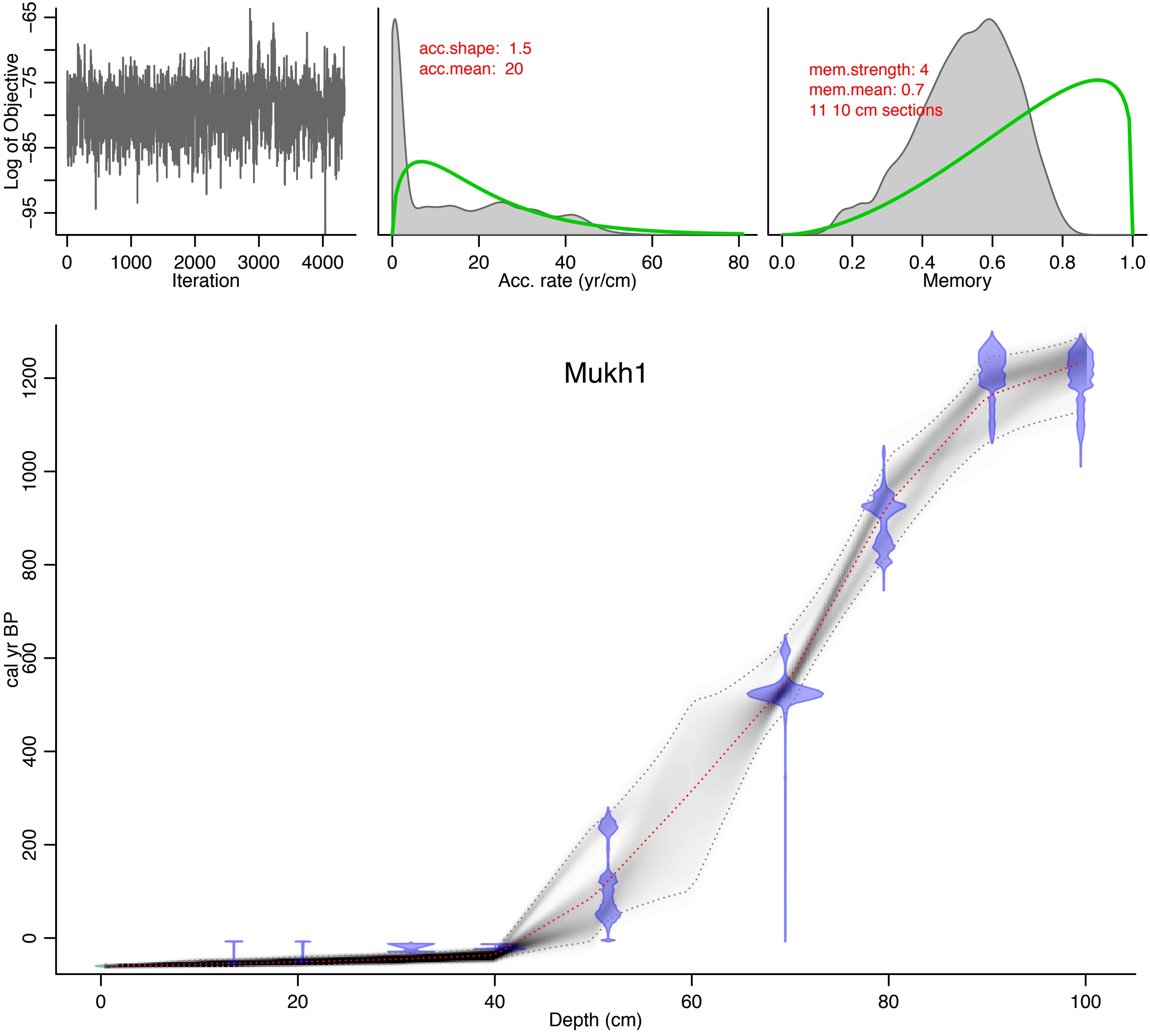

Log of Objective
Iteration
acc.shape: 1.5
acc.mean: 20
Acc. rate (yr/cm)
mem.strength: 4
mem.mean: 0.7
11 10 cm sections
Memory
Mukh1
cal yr BP
Depth (cm)

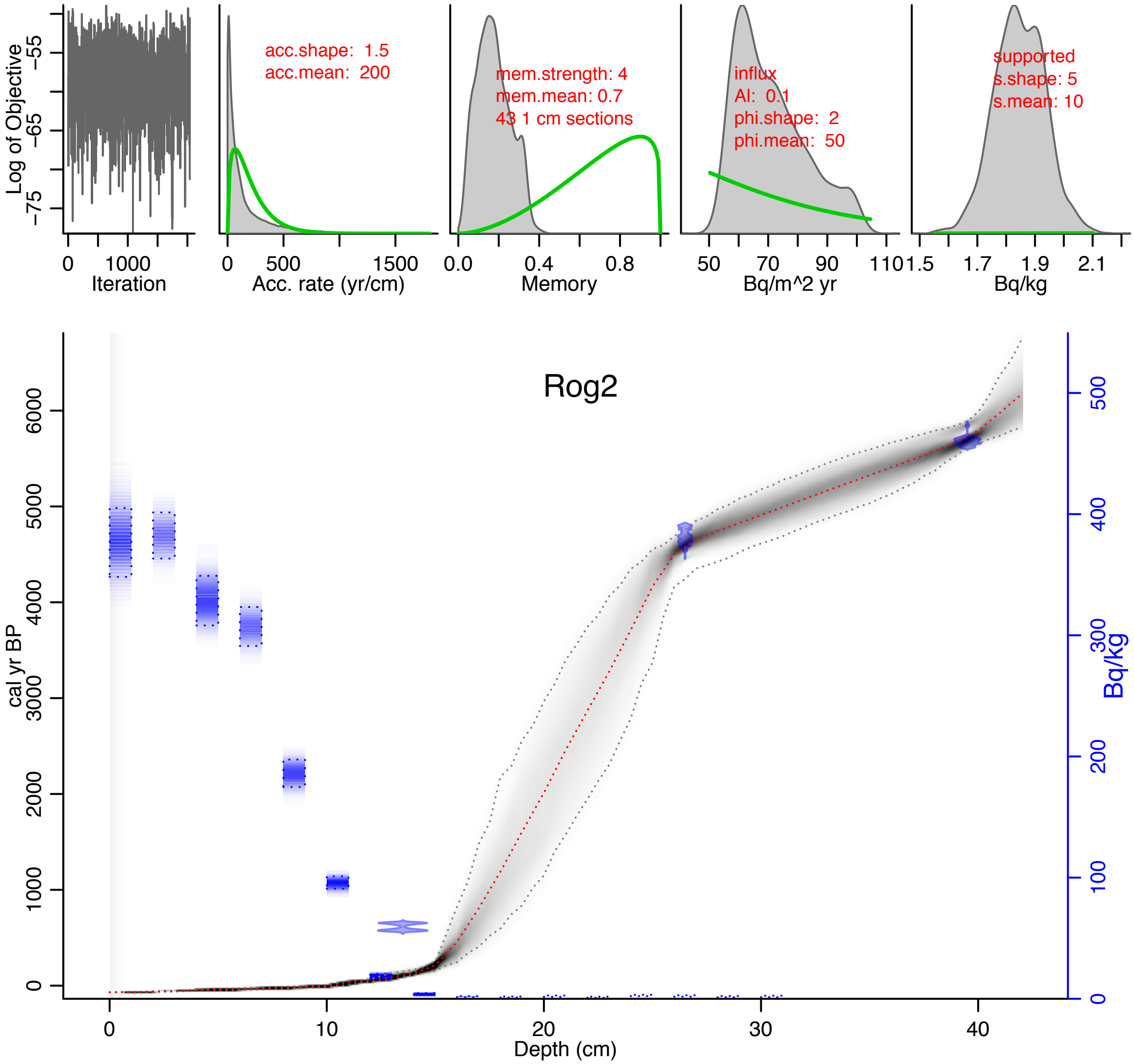
Log of Objective
−55
−65
−75
0
1000
Iteration
acc.shape: 1.5
acc.mean: 200
0
500
1500
Acc. rate (yr/cm)
mem.strength: 4
mem.mean: 0.7
43 1 cm sections
0.0
0.4
0.8
Memory
influx
Al: 0.1
phi.shape: 2
phi.mean: 50
50
70
90
110
Bq/m^2 yr
supported
s.shape: 5
s.mean: 10
1.5
1.7
1.9
2.1
Bq/kg
Rog2
cal yr BP
0
1000
2000
3000
4000
5000
6000
Bq/kg
0
100
200
300
400
500
0
10
20
30
40
Depth (cm)

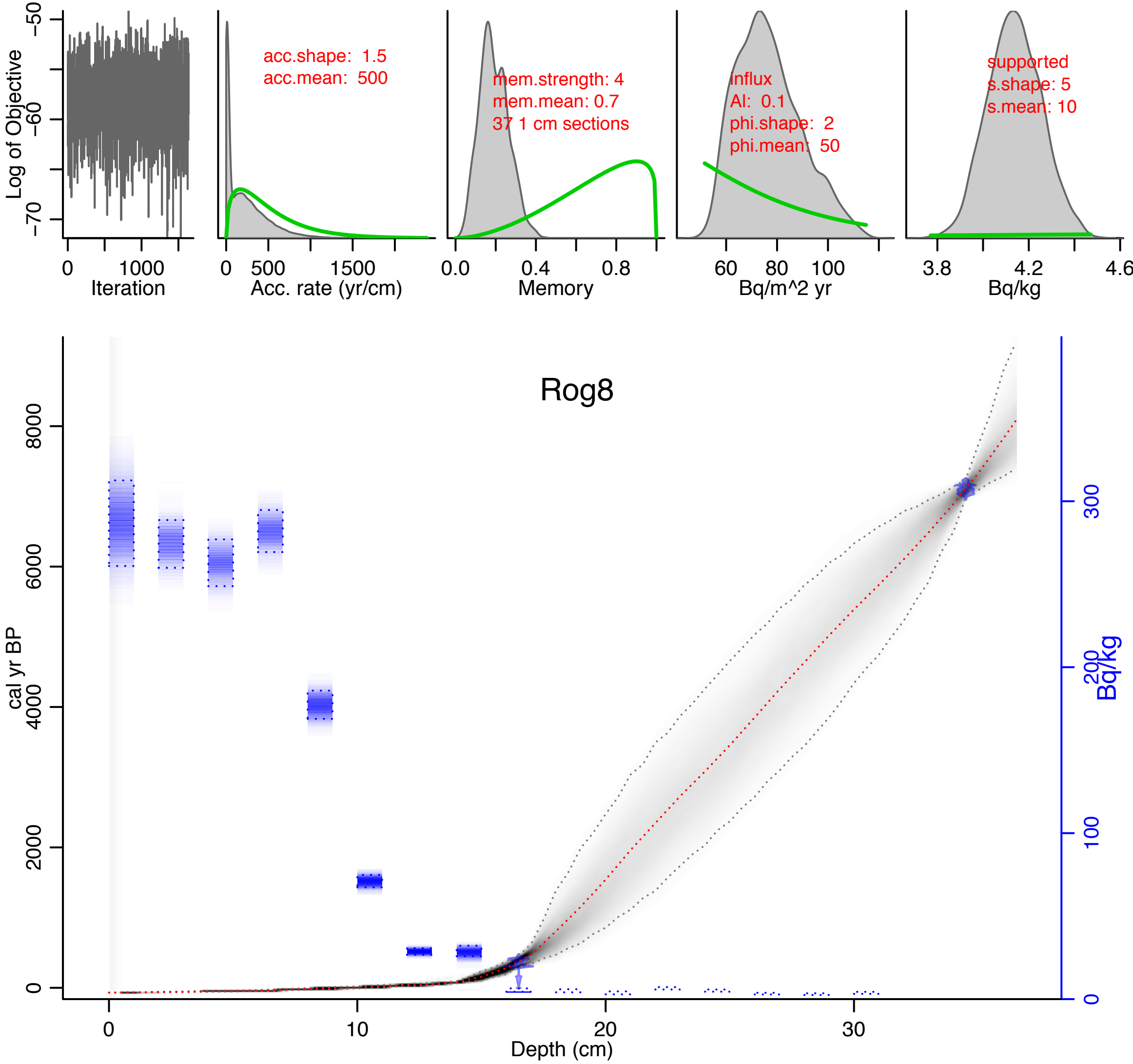

Log of Objective
Iteration
acc.shape: 1.5
acc.mean: 500
Acc. rate (yr/cm)
mem.strength: 4
mem.mean: 0.7
37 1 cm sections
Memory
influx
Al: 0.1
phi.shape: 2
phi.mean: 50
Bq/m^2 yr
supported
s.shape: 5
s.mean: 10
Bq/kg
Rog8
cal yr BP
Bq/kg
Depth (cm)

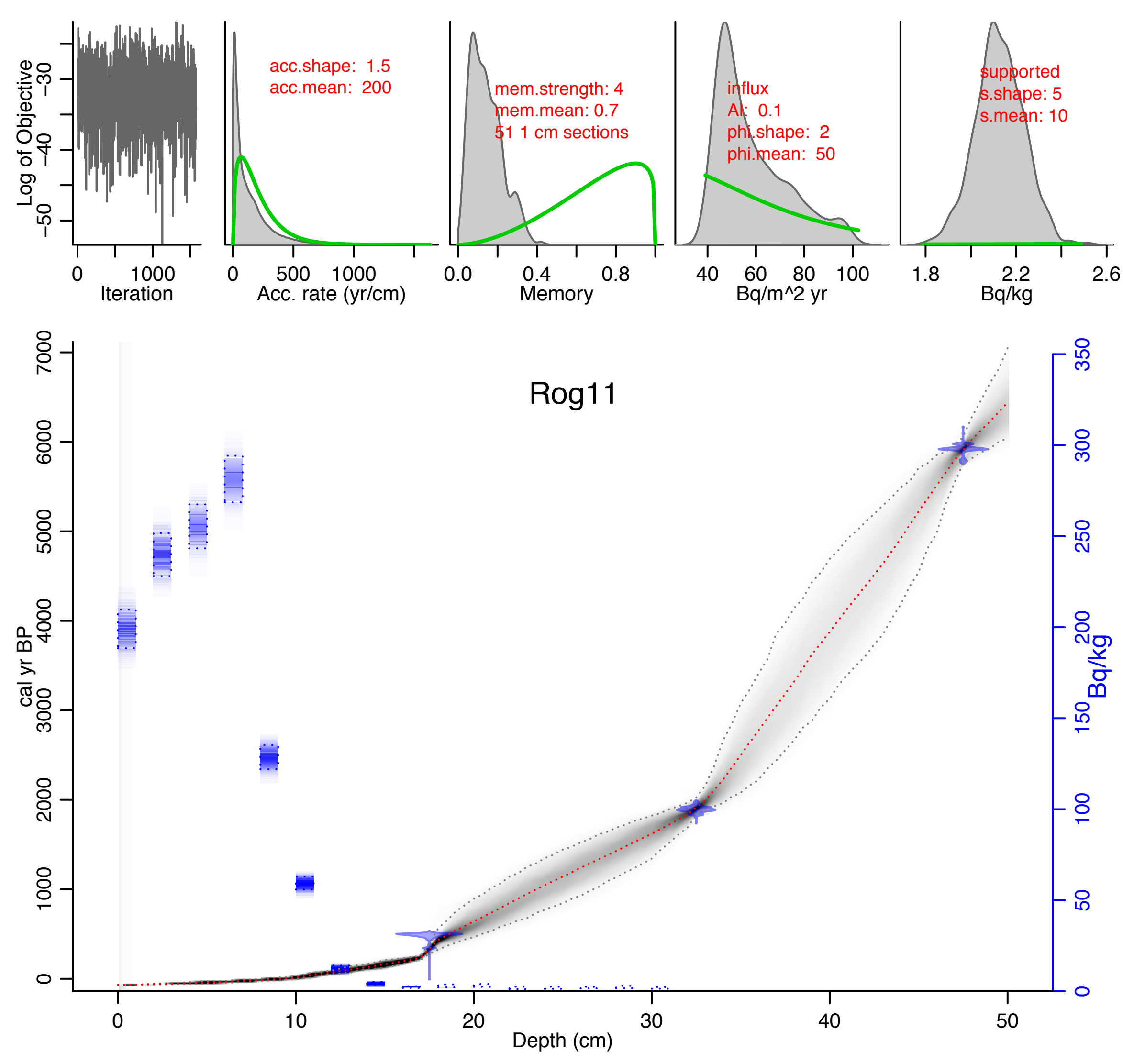

Log of Objective
Iteration
acc.shape: 1.5
acc.mean: 200
Acc. rate (yr/cm)
mem.strength: 4
mem.mean: 0.7
51 1 cm sections
Memory
influx
Al: 0.1
phi.shape: 2
phi.mean: 50
Bq/m^2 yr
supported
s.shape: 5
s.mean: 10
Bq/kg
Rog11
cal yr BP
Bq/kg
Depth (cm)

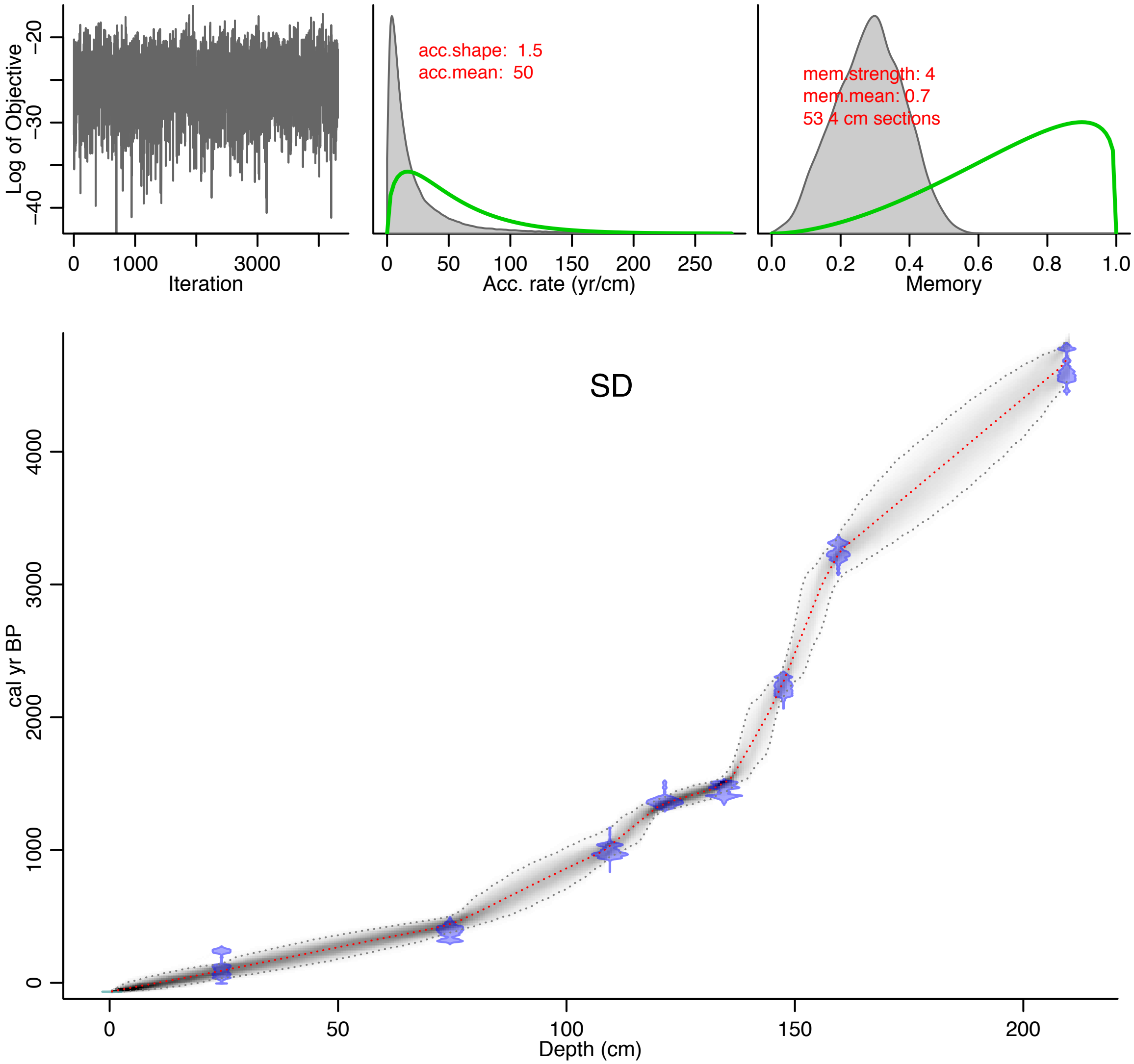

Log of Objective
−20
−30
−40
0
1000
3000
Iteration
acc.shape: 1.5
acc.mean: 50
0
50
100
150
200
250
Acc. rate (yr/cm)
mem.strength: 4
mem.mean: 0.7
53 4 cm sections
0.0
0.2
0.4
0.6
0.8
1.0
Memory
SD
cal yr BP
0
1000
2000
3000
4000
0
50
100
150
200
Depth (cm)

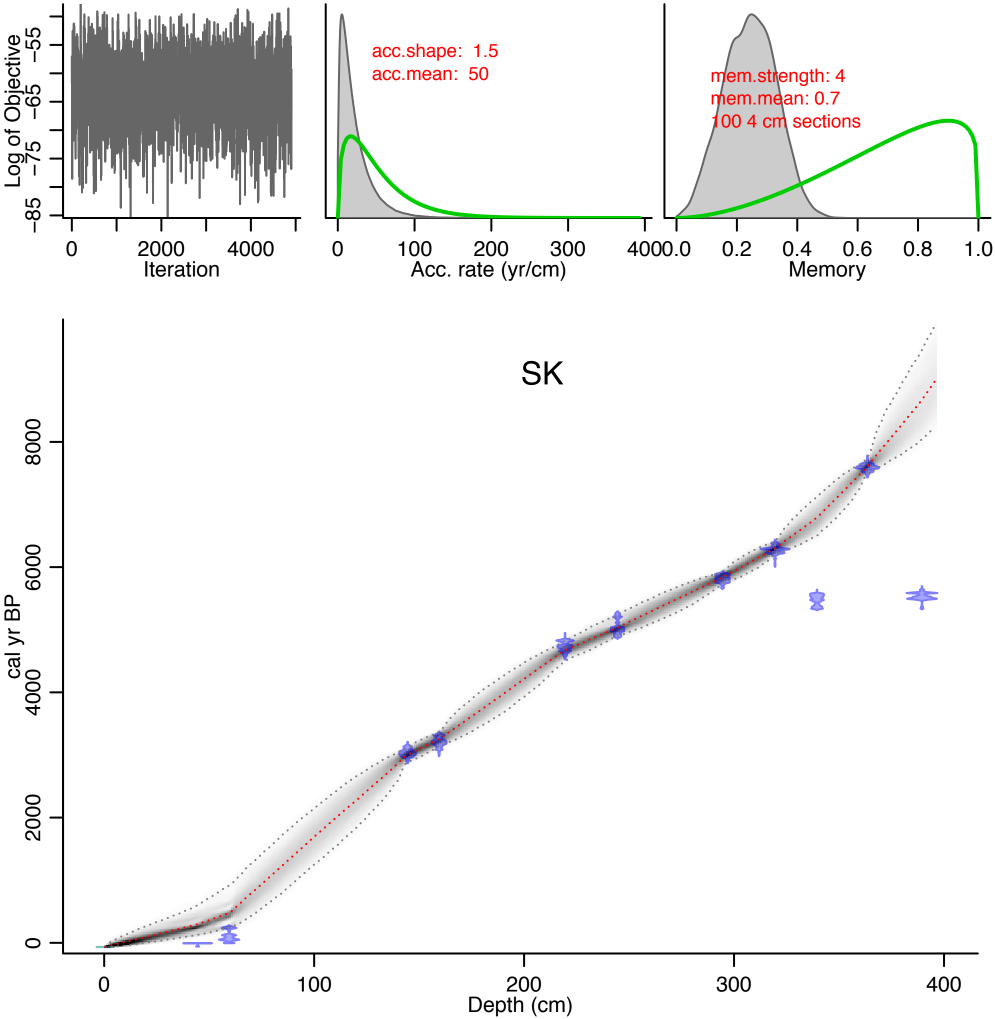
Log of Objective
Iteration
acc.shape: 1.5
acc.mean: 50
Acc. rate (yr/cm)
mem.strength: 4
mem.mean: 0.7
100 4 cm sections
Memory
SK
cal yr BP
Depth (cm)

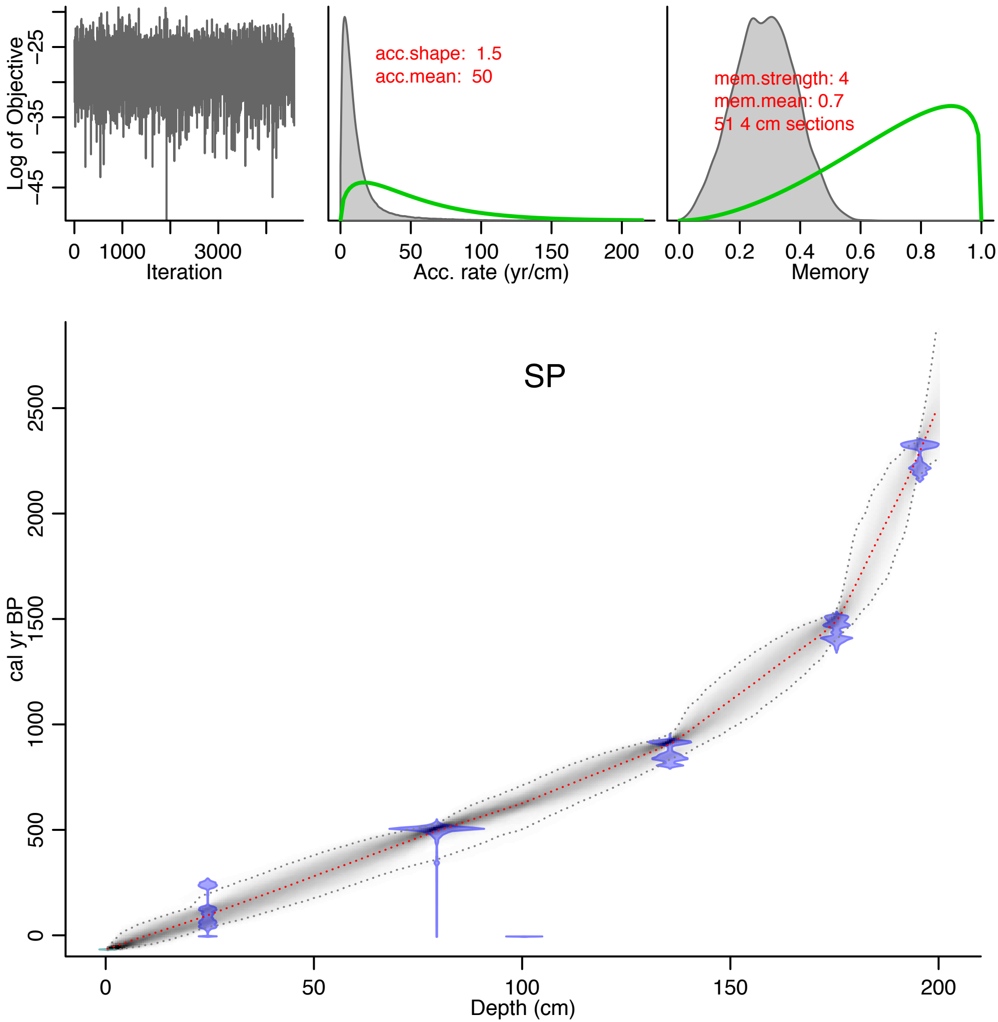

Log of Objective
−25
−35
−45
0 1000 3000
Iteration
acc.shape: 1.5
acc.mean: 50
0 50 100 150 200
Acc. rate (yr/cm)
mem.strength: 4
mem.mean: 0.7
51 4 cm sections
0.0 0.2 0.4 0.6 0.8 1.0
Memory
SP
cal yr BP
0
500
1000
1500
2000
2500
0 50 100 150 200
Depth (cm)

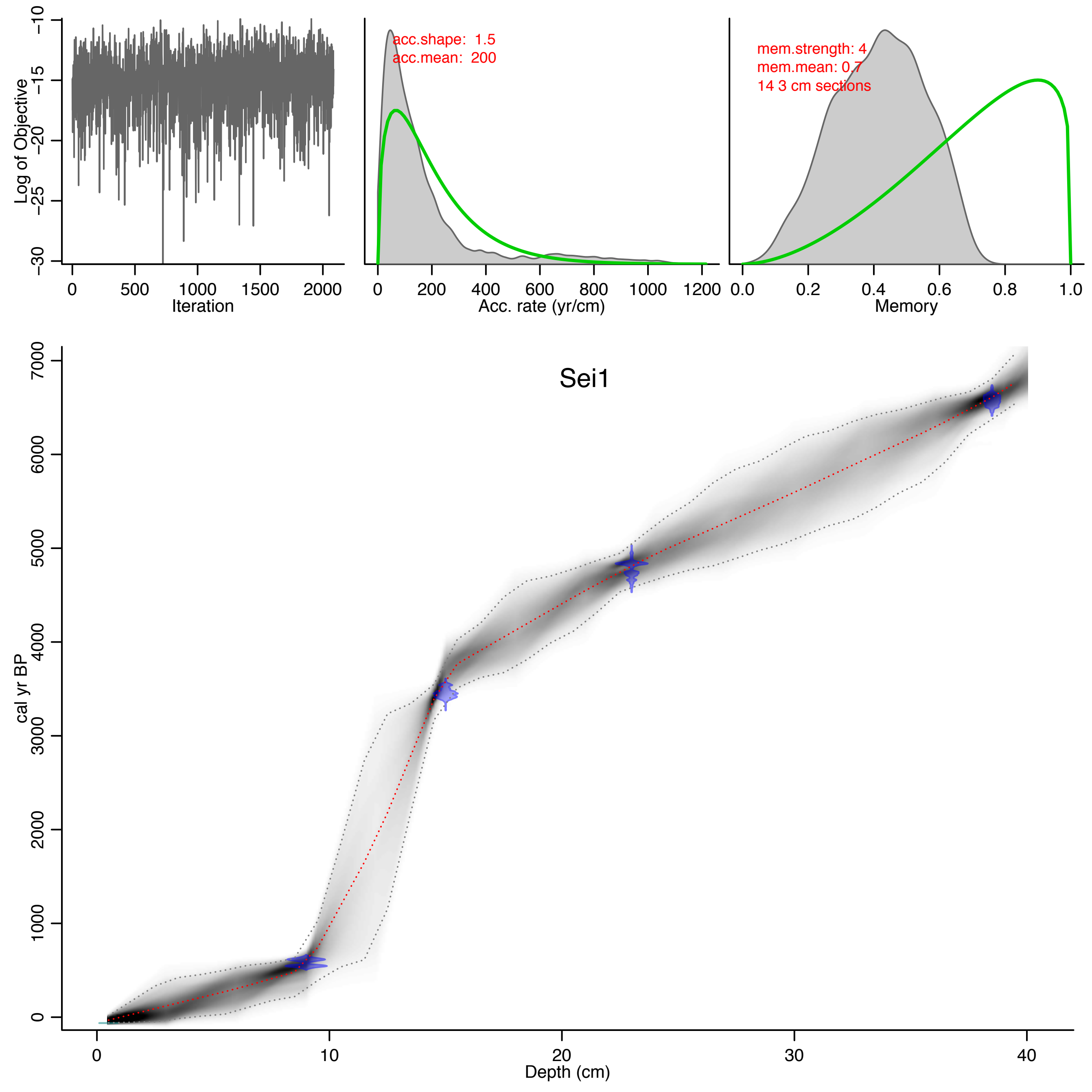

Log of Objective
Iteration
acc.shape: 1.5
acc.mean: 200
Acc. rate (yr/cm)
mem.strength: 4
mem.mean: 0.7
14 3 cm sections
Memory
Sei1
cal yr BP
Depth (cm)

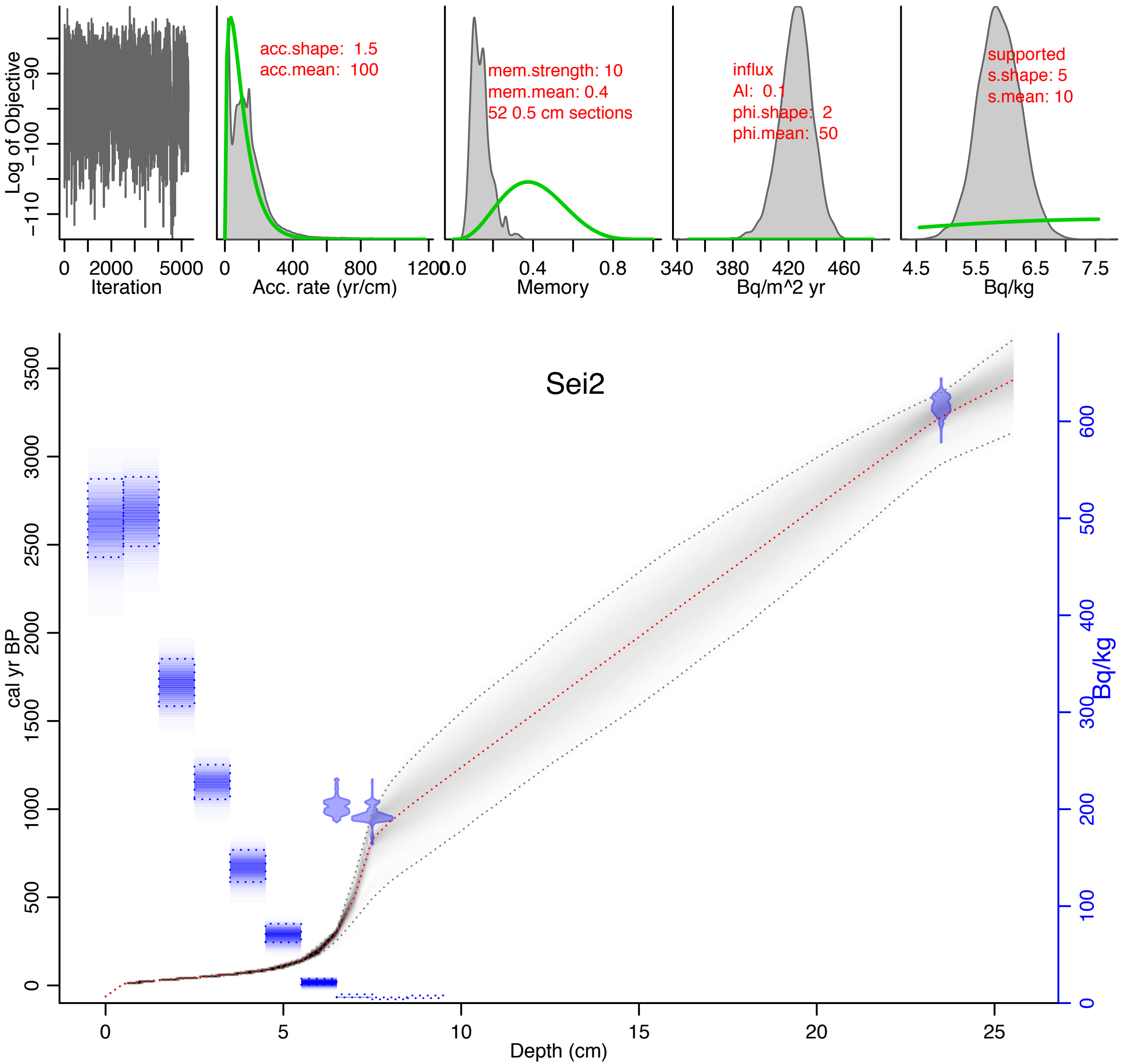

Log of Objective
Iteration
acc.shape: 1.5
acc.mean: 100
Acc. rate (yr/cm)
mem.strength: 10
mem.mean: 0.4
52 0.5 cm sections
Memory
influx
Al: 0.1
phi.shape: 2
phi.mean: 50
Bq/m^2 yr
supported
s.shape: 5
s.mean: 10
Bq/kg
Sei2
cal yr BP
Bq/kg
Depth (cm)

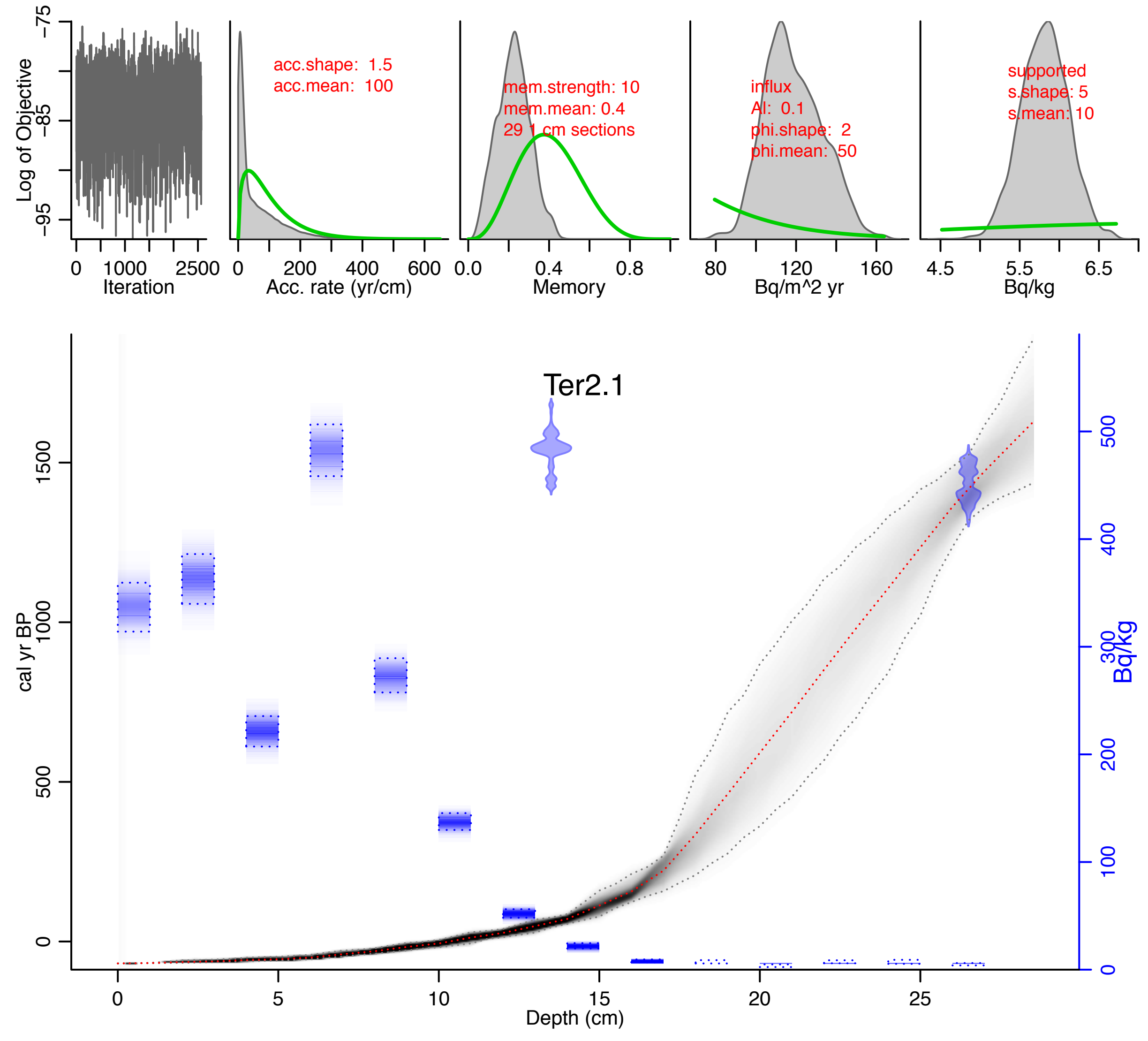

Log of Objective
−75
−85
−95
0 1000 2500
Iteration
acc.shape: 1.5
acc.mean: 100
0 200 400 600
Acc. rate (yr/cm)
mem.strength: 10
mem.mean: 0.4
29 1 cm sections
0.0 0.4 0.8
Memory
influx
Al: 0.1
phi.shape: 2
phi.mean: 50
80 120 160
Bq/m^2 yr
supported
s.shape: 5
s.mean: 10
4.5 5.5 6.5
Bq/kg
Ter2.1
cal yr BP
0
500
1000
1500
Depth (cm)
0 5 10 15 20 25
Bq/kg
0 100 200 300 400 500

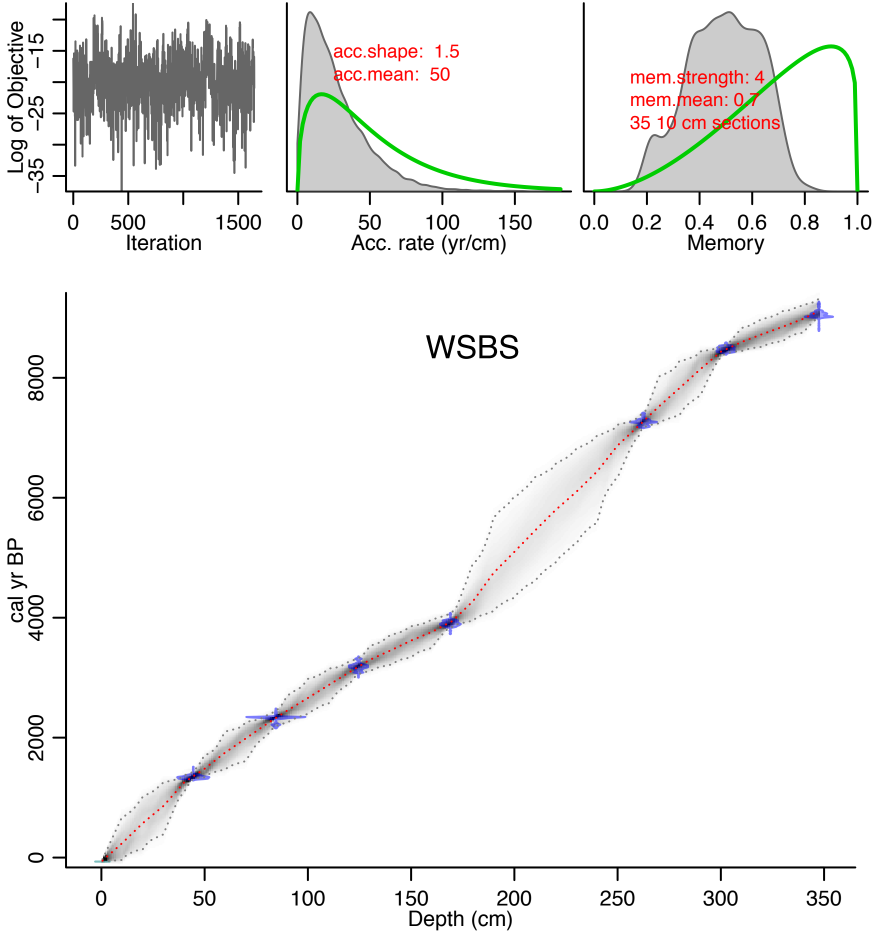

Log of Objective
−15
−25
−35
0
500
1500
Iteration
acc.shape: 1.5
acc.mean: 50
0
50
100
150
Acc. rate (yr/cm)
mem.strength: 4
mem.mean: 0.7
35 10 cm sections
0.0
0.2
0.4
0.6
0.8
1.0
Memory
WSBS
cal yr BP
0
2000
4000
6000
8000
0
50
100
150
200
250
300
350
Depth (cm)

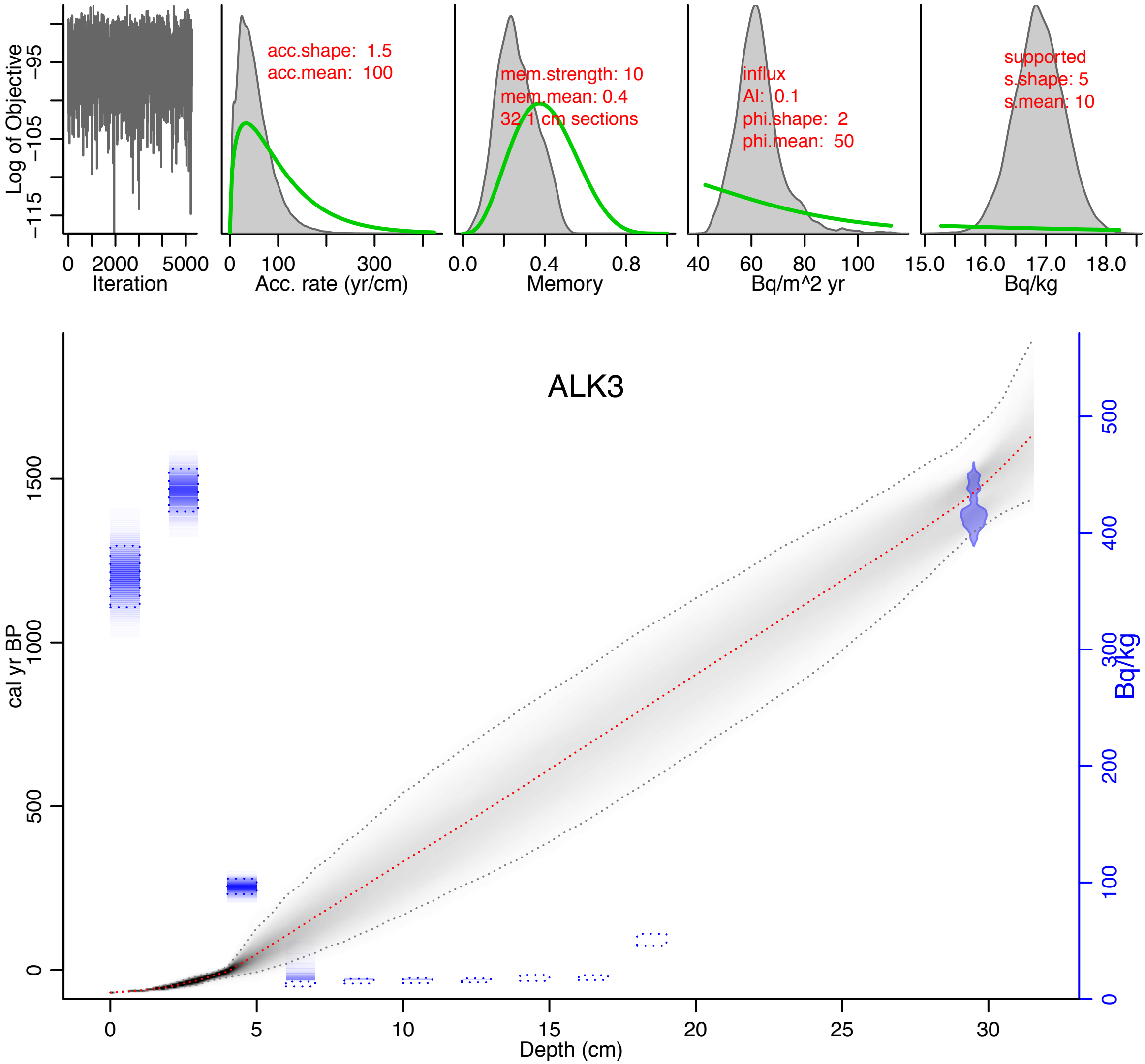

Log of Objective
−95
−105
−115
0
2000
5000
Iteration
acc.shape: 1.5
acc.mean: 100
0
100
300
Acc. rate (yr/cm)
mem.strength: 10
mem.mean: 0.4
32 1 cm sections
0.0
0.4
0.8
Memory
influx
Al: 0.1
phi.shape: 2
phi.mean: 50
40
60
80
100
Bq/m^2 yr
supported
s.shape: 5
s.mean: 10
15.0
16.0
17.0
18.0
Bq/kg
ALK3
cal yr BP
0
500
1000
1500
Depth (cm)
0
5
10
15
20
25
30
Bq/kg
0
100
200
300
400
500

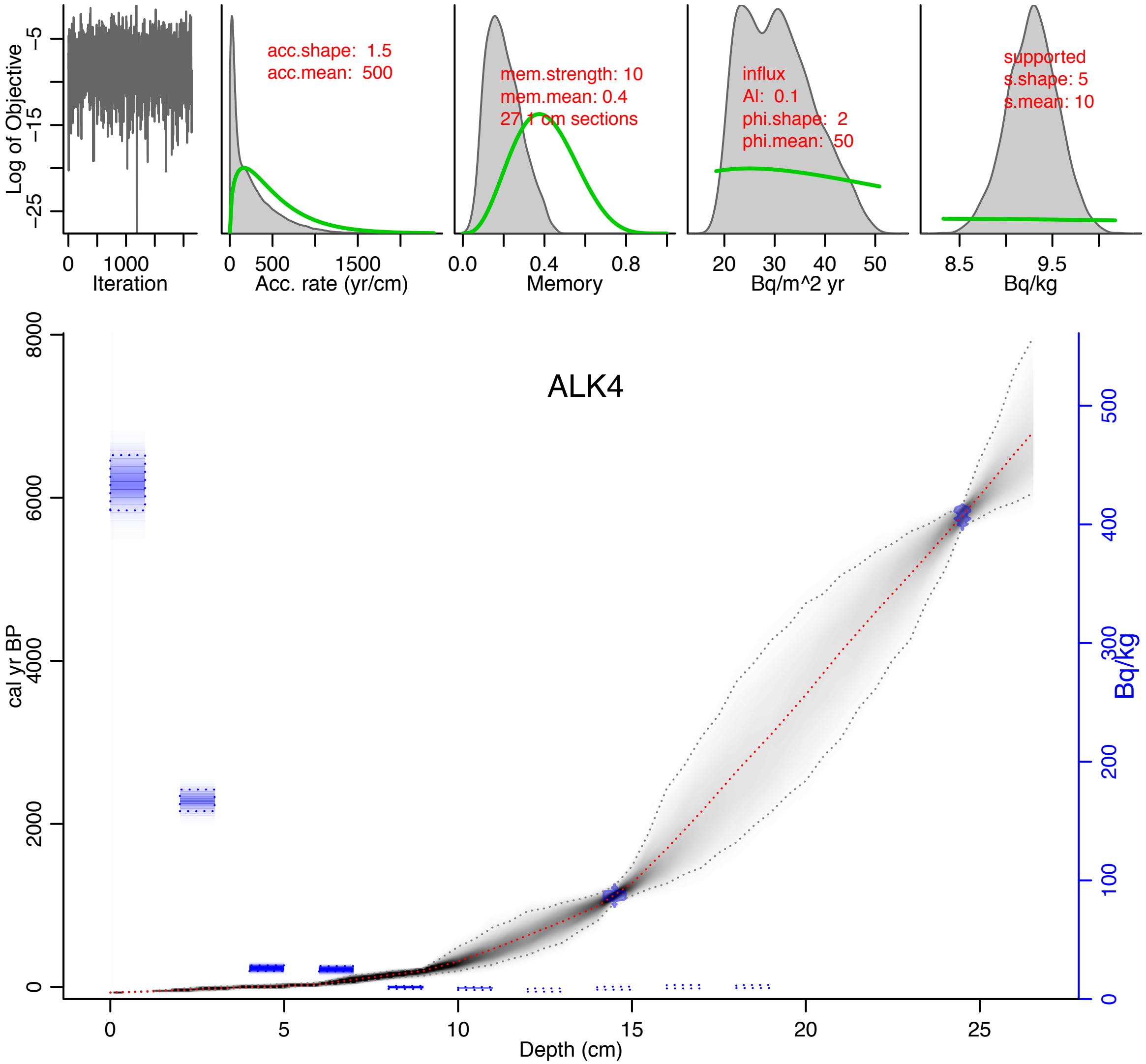

Log of Objective
Iteration
acc.shape: 1.5
acc.mean: 500
Acc. rate (yr/cm)
mem.strength: 10
mem.mean: 0.4
27 1 cm sections
Memory
influx
Al: 0.1
phi.shape: 2
phi.mean: 50
Bq/m^2 yr
supported
s.shape: 5
s.mean: 10
Bq/kg
ALK4
cal yr BP
Bq/kg
Depth (cm)

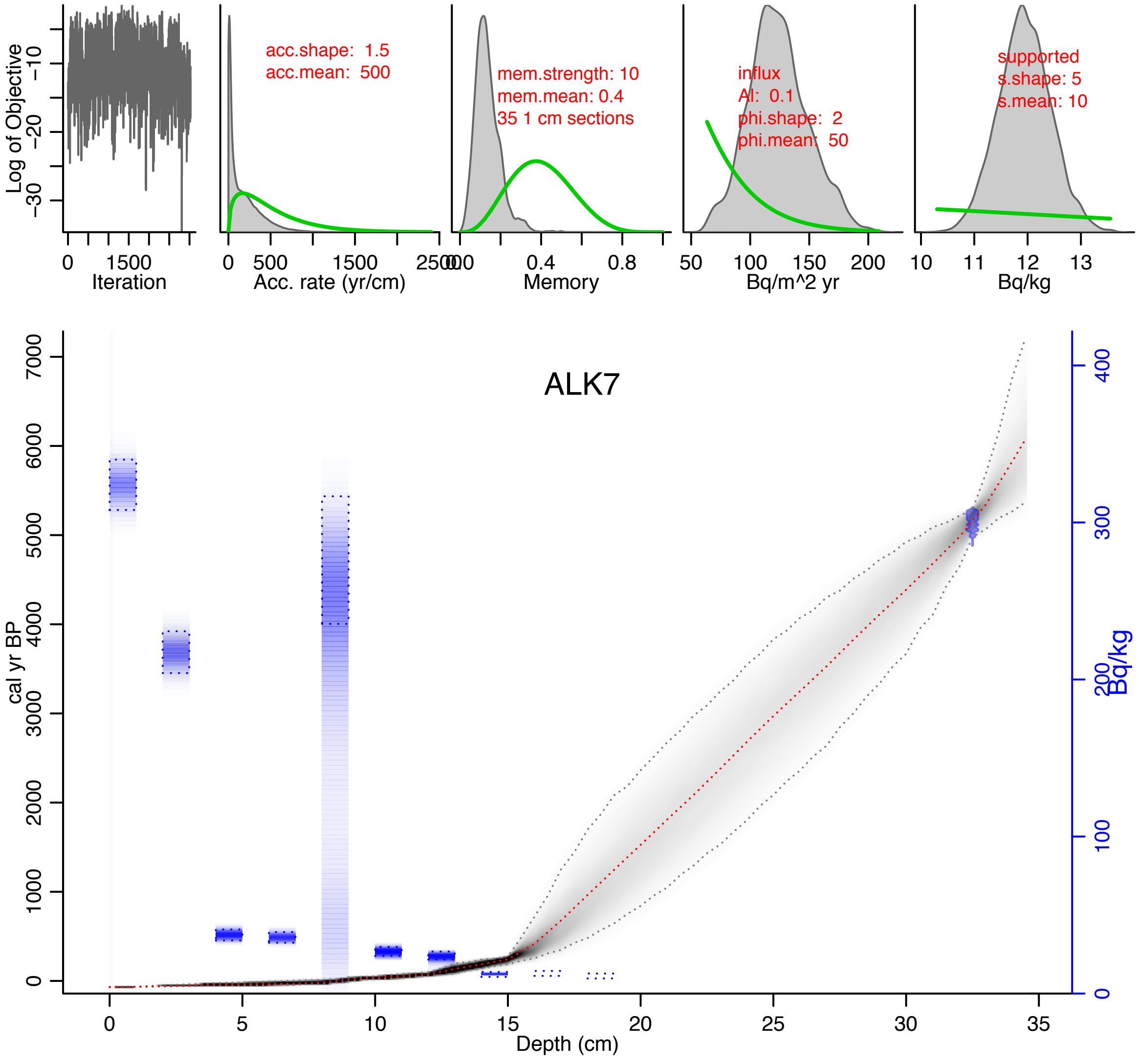
Log of Objective
Iteration
acc.shape: 1.5
acc.mean: 500
Acc. rate (yr/cm)
mem.strength: 10
mem.mean: 0.4
35 1 cm sections
Memory
influx
Al: 0.1
phi.shape: 2
phi.mean: 50
Bq/m^2 yr
supported
s.shape: 5
s.mean: 10
Bq/kg
ALK7
cal yr BP
Bq/kg
Depth (cm)

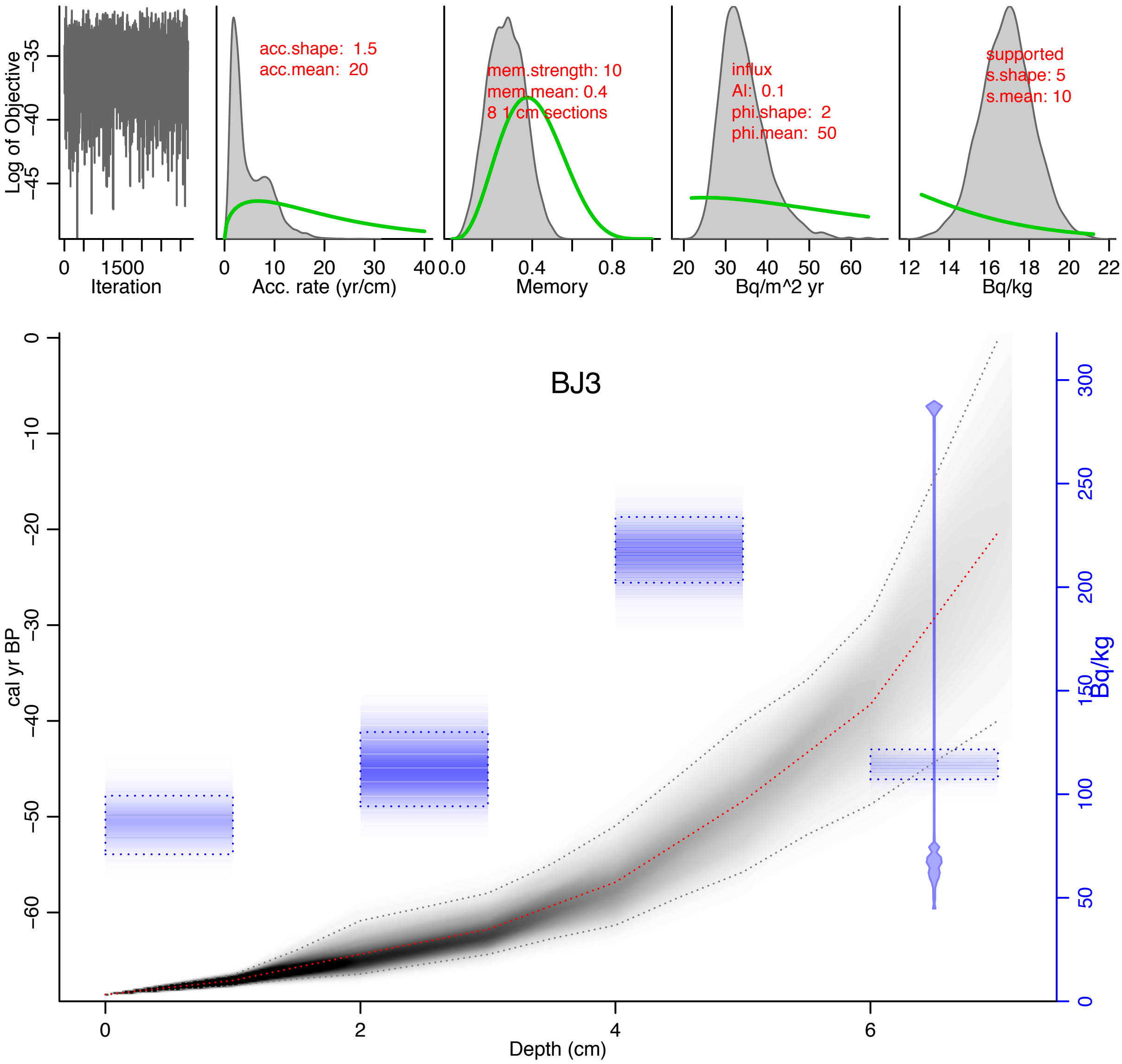

Log of Objective
-35
-40
-45
0
1500
Iteration
acc.shape: 1.5
acc.mean: 20
0
10
20
30
40
Acc. rate (yr/cm)
mem.strength: 10
mem.mean: 0.4
8 1 cm sections
0.0
0.4
0.8
Memory
influx
Al: 0.1
phi.shape: 2
phi.mean: 50
20
30
40
50
60
Bq/m^2 yr
supported
s.shape: 5
s.mean: 10
12
14
16
18
20
22
Bq/kg
BJ3
cal yr BP
0
-10
-20
-30
-40
-50
-60
Depth (cm)
0
2
4
6
Bq/kg
0
50
100
150
200
250
300

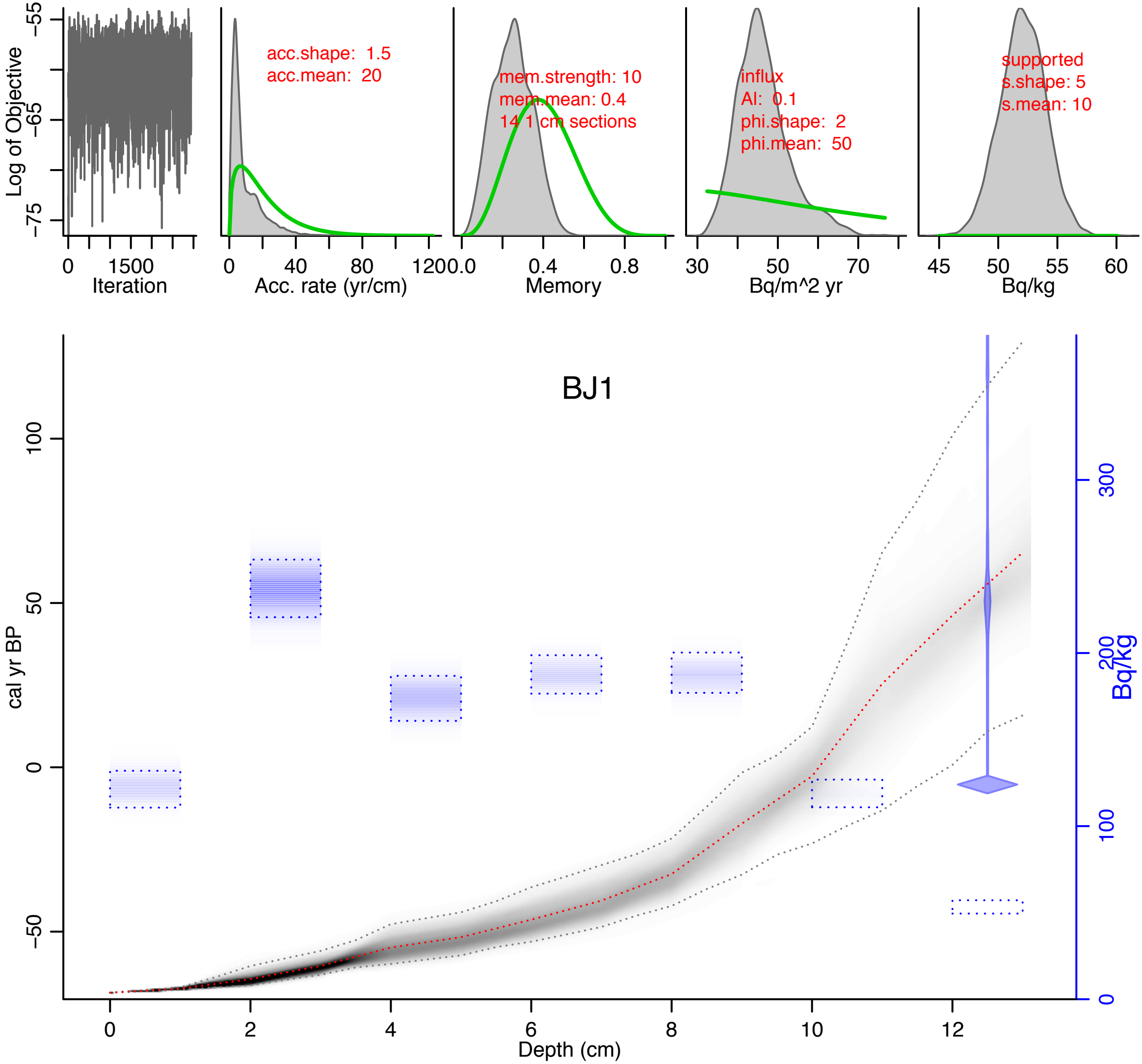
Log of Objective
−55
−65
−75
0
1500
Iteration
acc.shape: 1.5
acc.mean: 20
0
40
80
120
Acc. rate (yr/cm)
mem.strength: 10
mem.mean: 0.4
14 1 cm sections
0.0
0.4
0.8
Memory
influx
Al: 0.1
phi.shape: 2
phi.mean: 50
30
50
70
Bq/m^2 yr
supported
s.shape: 5
s.mean: 10
45
50
55
60
Bq/kg
BJ1
cal yr BP
100
50
0
−50
0
2
4
6
8
10
12
Depth (cm)
Bq/kg
300
200
100
0

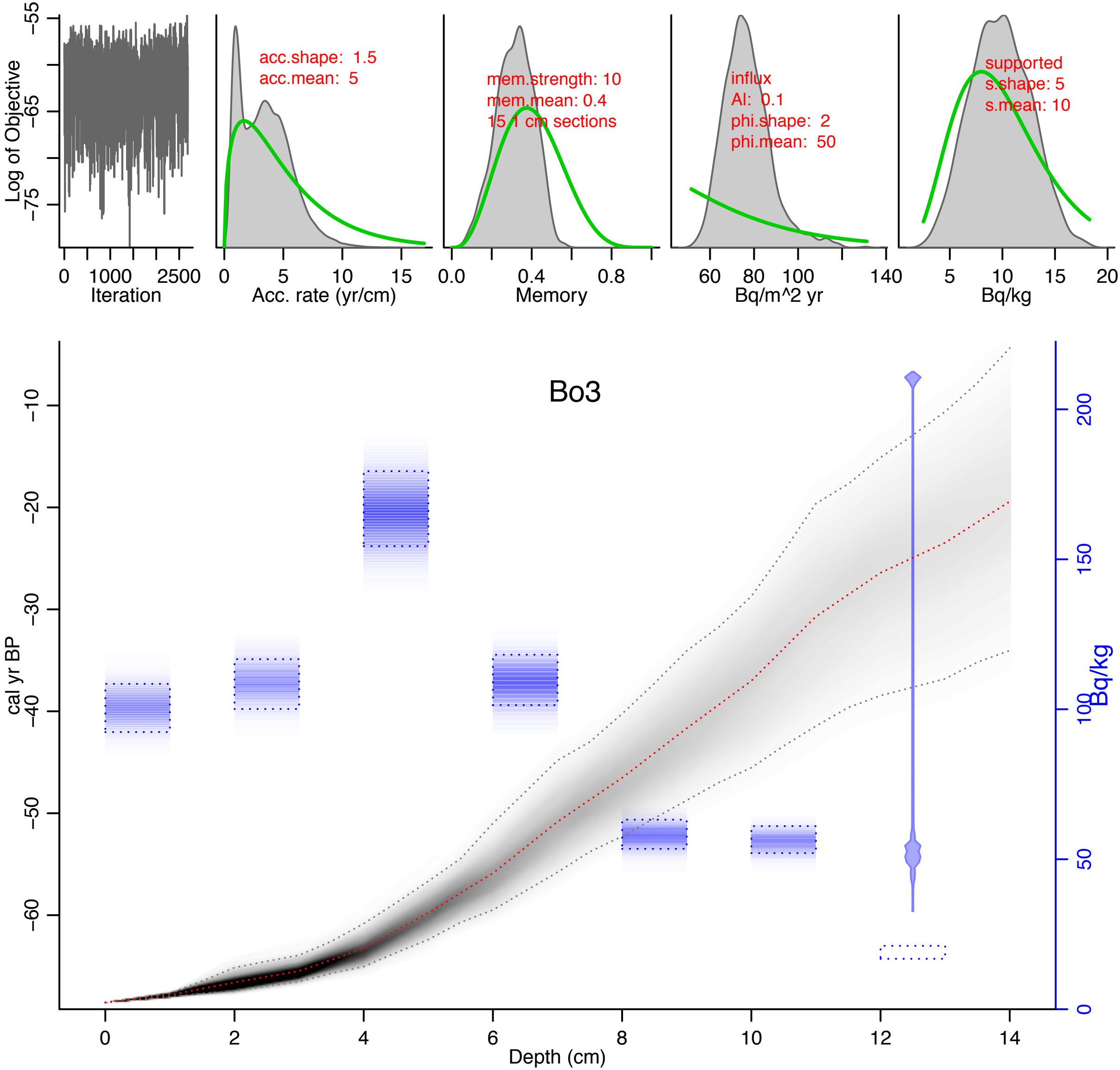

Log of Objective
Iteration
acc.shape: 1.5
acc.mean: 5
Acc. rate (yr/cm)
mem.strength: 10
mem.mean: 0.4
15 1 cm sections
Memory
influx
Al: 0.1
phi.shape: 2
phi.mean: 50
Bq/m^2 yr
supported
s.shape: 5
s.mean: 10
Bq/kg
Bo3
cal yr BP
Bq/kg
Depth (cm)

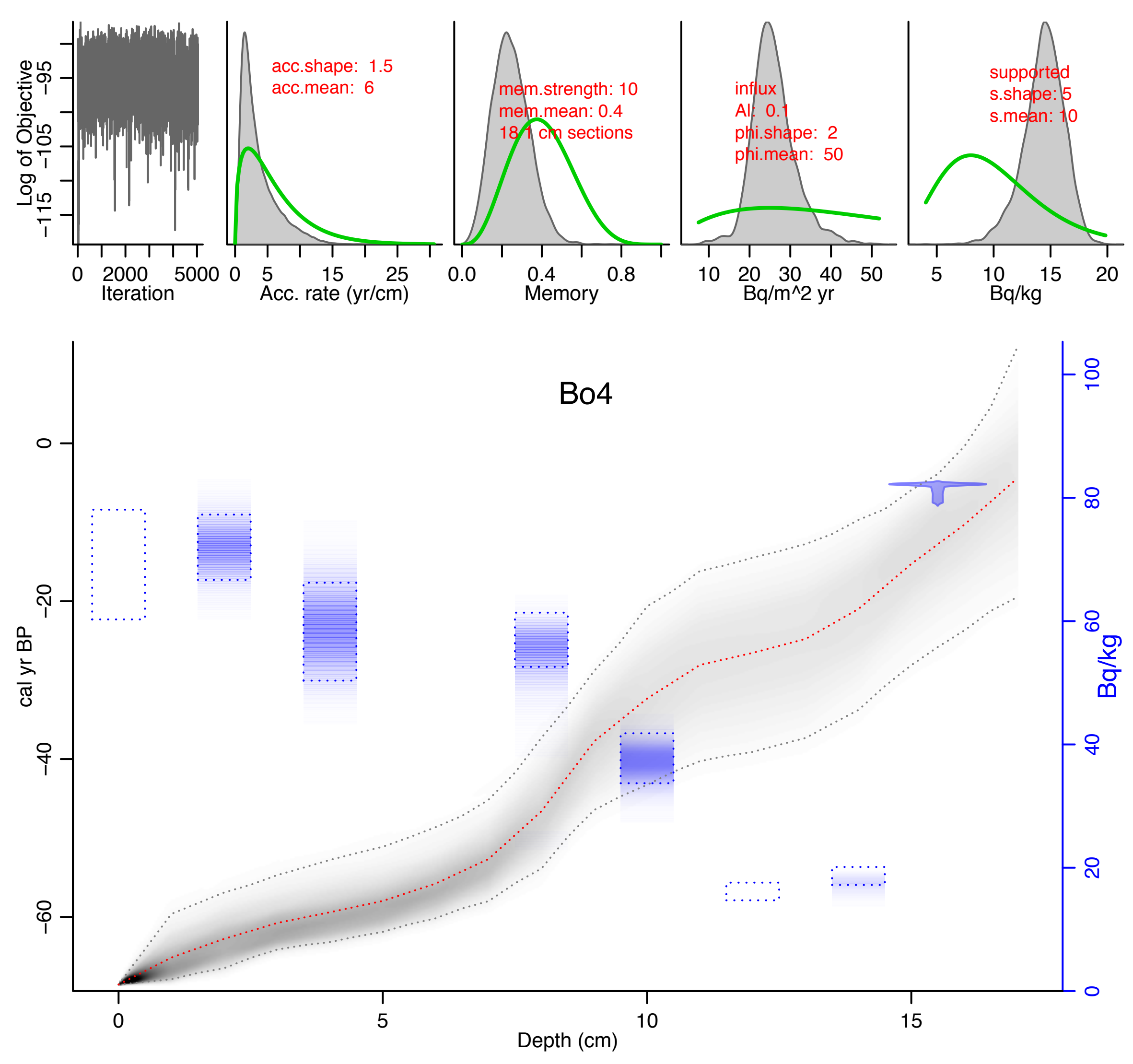

Log of Objective
Iteration
acc.shape: 1.5
acc.mean: 6
Acc. rate (yr/cm)
mem.strength: 10
mem.mean: 0.4
18 1 cm sections
Memory
influx
Al: 0.1
phi.shape: 2
phi.mean: 50
Bq/m^2 yr
supported
s.shape: 5
s.mean: 10
Bq/kg
Bo4
cal yr BP
Depth (cm)
Bq/kg

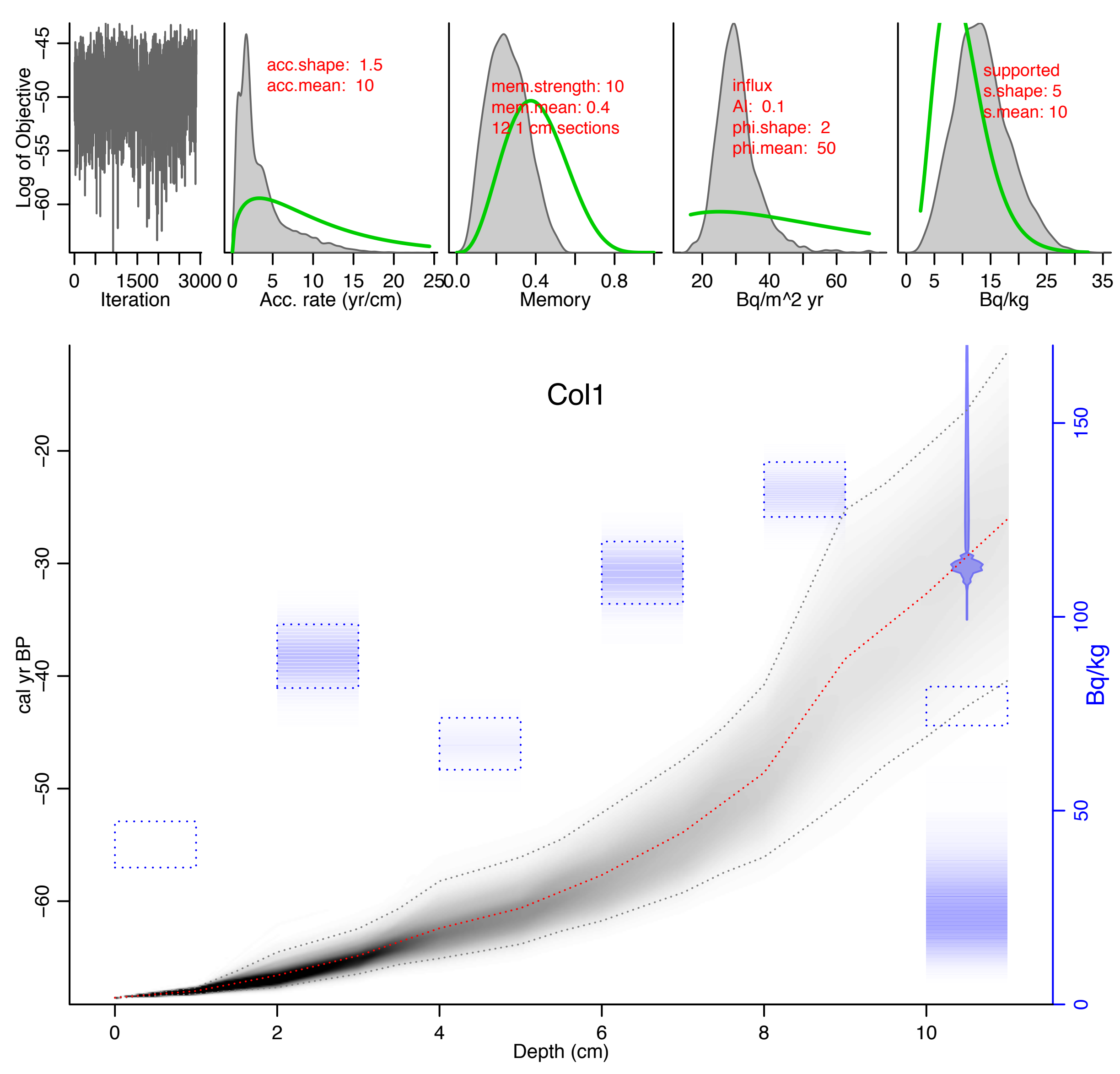

Log of Objective
Iteration
acc.shape: 1.5
acc.mean: 10
Acc. rate (yr/cm)
mem.strength: 10
mem.mean: 0.4
12 1 cm sections
Memory
influx
Al: 0.1
phi.shape: 2
phi.mean: 50
Bq/m^2 yr
supported
s.shape: 5
s.mean: 10
Bq/kg
Col1
cal yr BP
Depth (cm)
Bq/kg

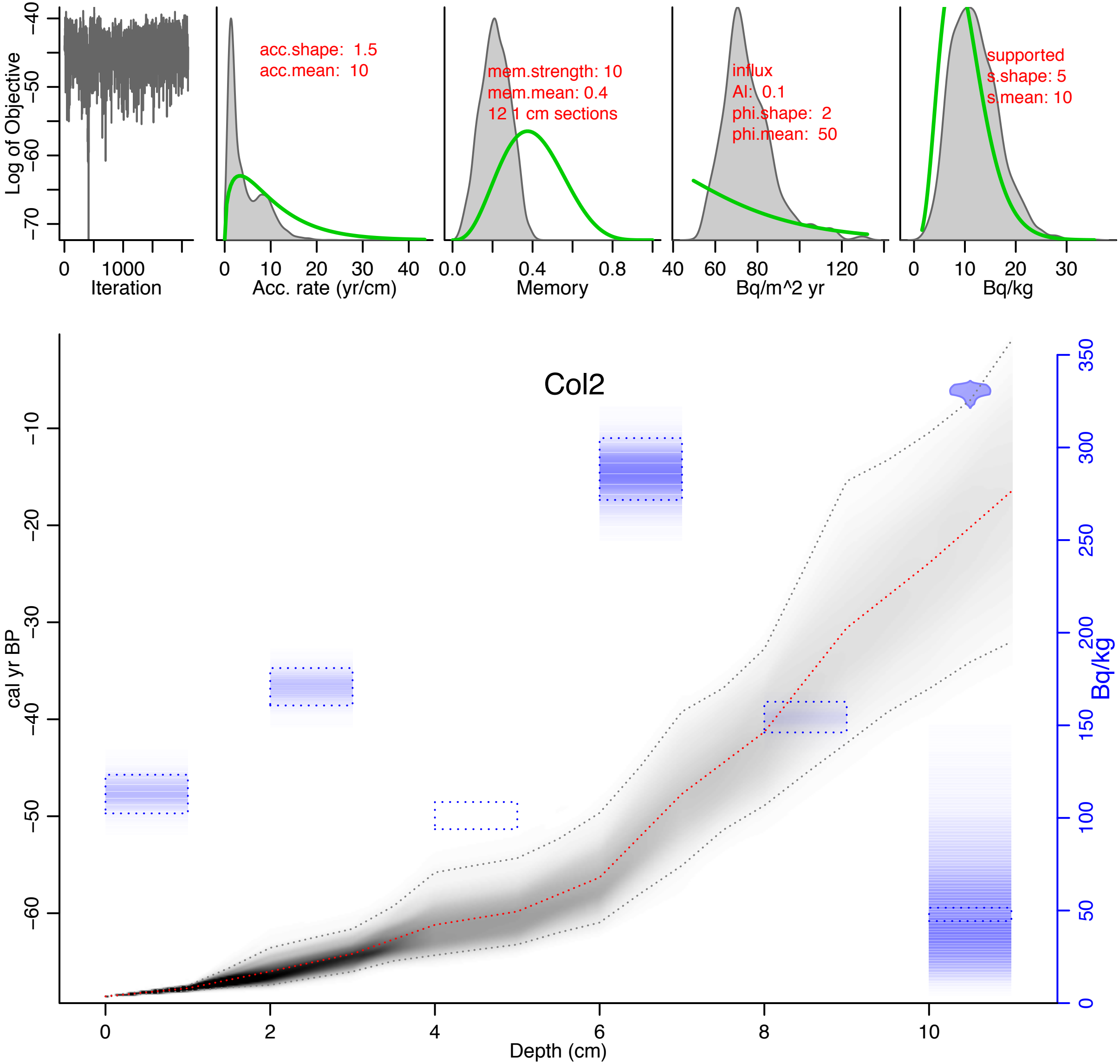

Log of Objective
Iteration
acc.shape: 1.5
acc.mean: 10
Acc. rate (yr/cm)
mem.strength: 10
mem.mean: 0.4
12 1 cm sections
Memory
influx
Al: 0.1
phi.shape: 2
phi.mean: 50
Bq/m^2 yr
supported
s.shape: 5
s.mean: 10
Bq/kg
Col2
cal yr BP
Bq/kg
Depth (cm)

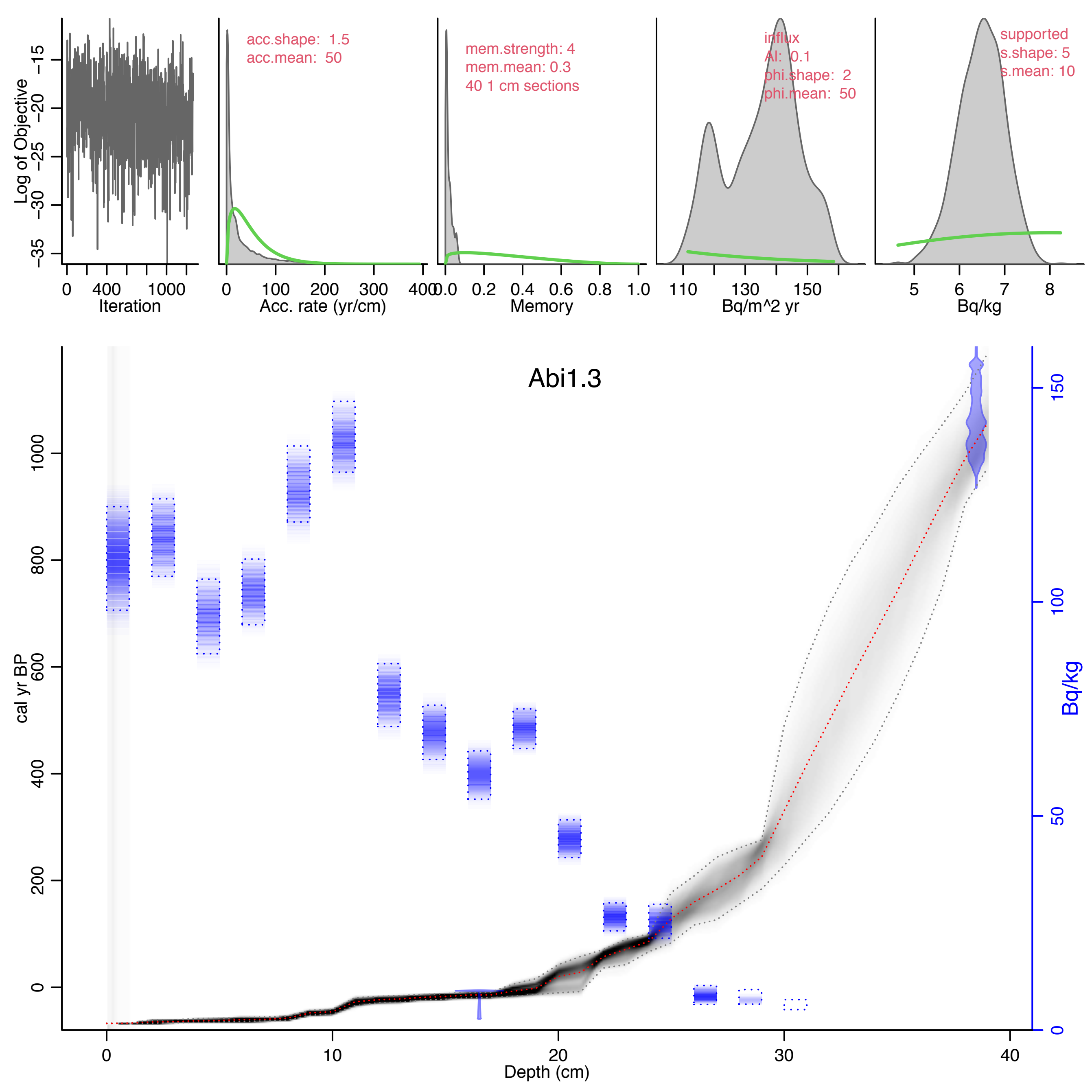

Log of Objective
Iteration
acc.shape: 1.5
acc.mean: 50
Acc. rate (yr/cm)
mem.strength: 4
mem.mean: 0.3
40 1 cm sections
Memory
influx
Al: 0.1
phi.shape: 2
phi.mean: 50
Bq/m^2 yr
supported
s.shape: 5
s.mean: 10
Bq/kg
Abi1.3
cal yr BP
Depth (cm)
Bq/kg

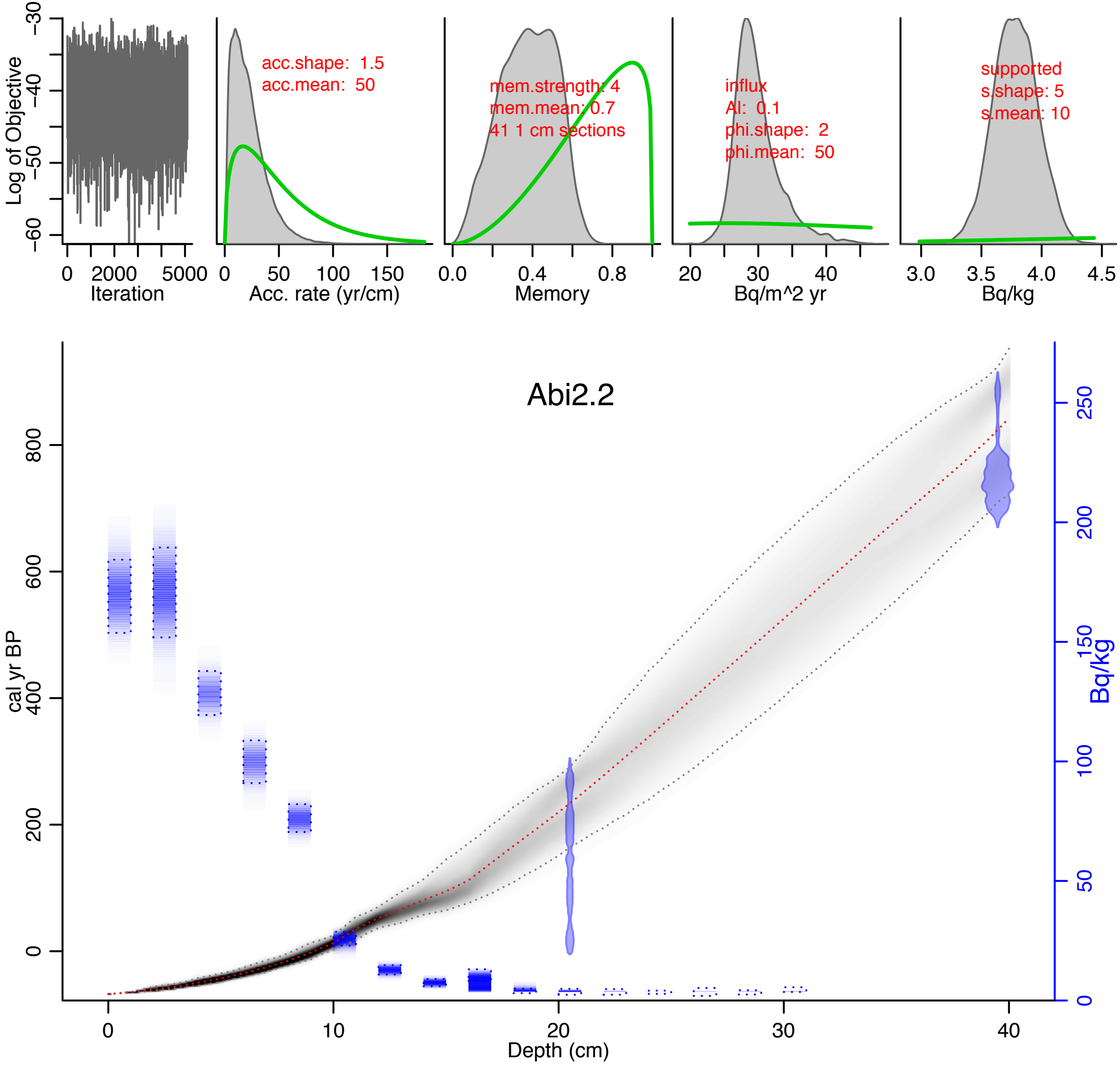
Log of Objective
Iteration
acc.shape: 1.5
acc.mean: 50
Acc. rate (yr/cm)
mem.strength: 4
mem.mean: 0.7
41 1 cm sections
Memory
influx
Al: 0.1
phi.shape: 2
phi.mean: 50
Bq/m^2 yr
supported
s.shape: 5
s.mean: 10
Bq/kg
Abi2.2
cal yr BP
Bq/kg
Depth (cm)

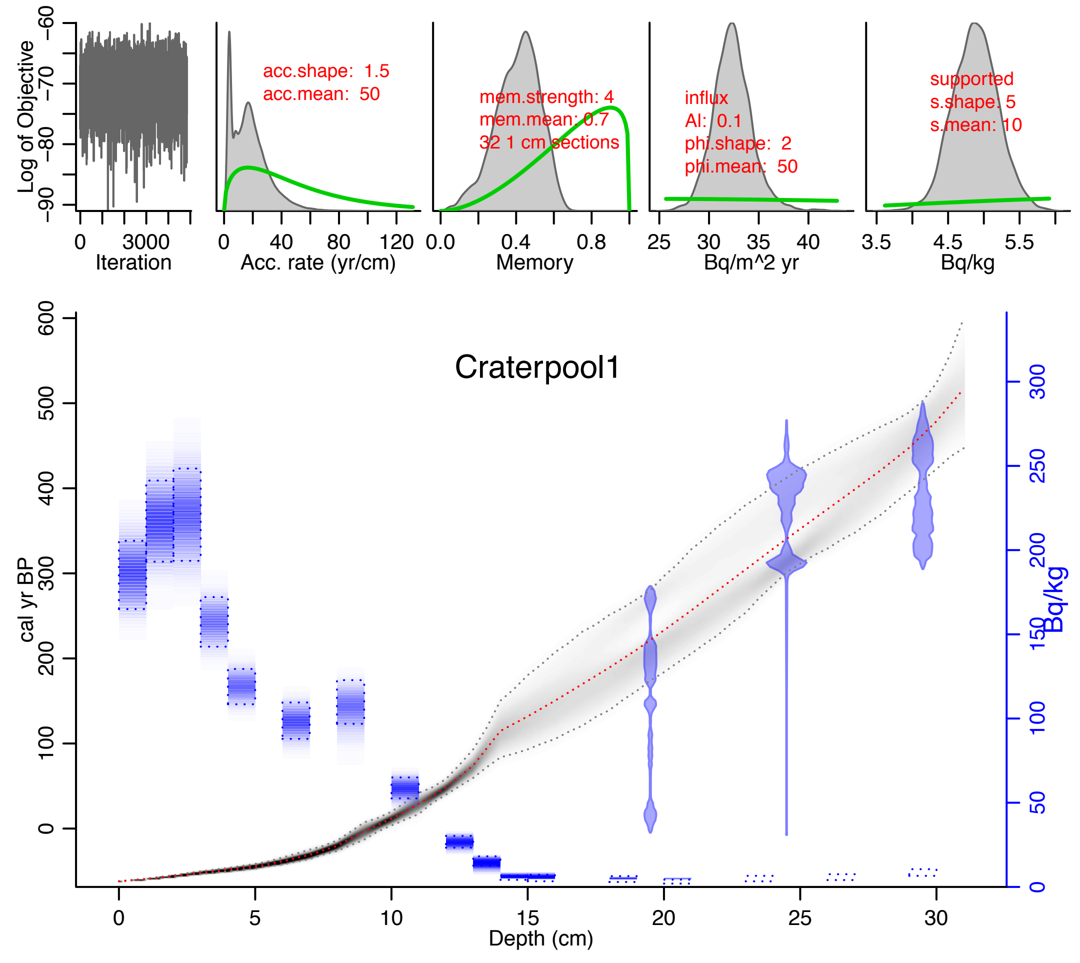
Log of Objective
Iteration
acc.shape: 1.5
acc.mean: 50
Acc. rate (yr/cm)
mem.strength: 4
mem.mean: 0.7
32 1 cm sections
Memory
influx
Al: 0.1
phi.shape: 2
phi.mean: 50
Bq/m^2 yr
supported
s.shape: 5
s.mean: 10
Bq/kg
Craterpool1
cal yr BP
Depth (cm)
Bq/kg

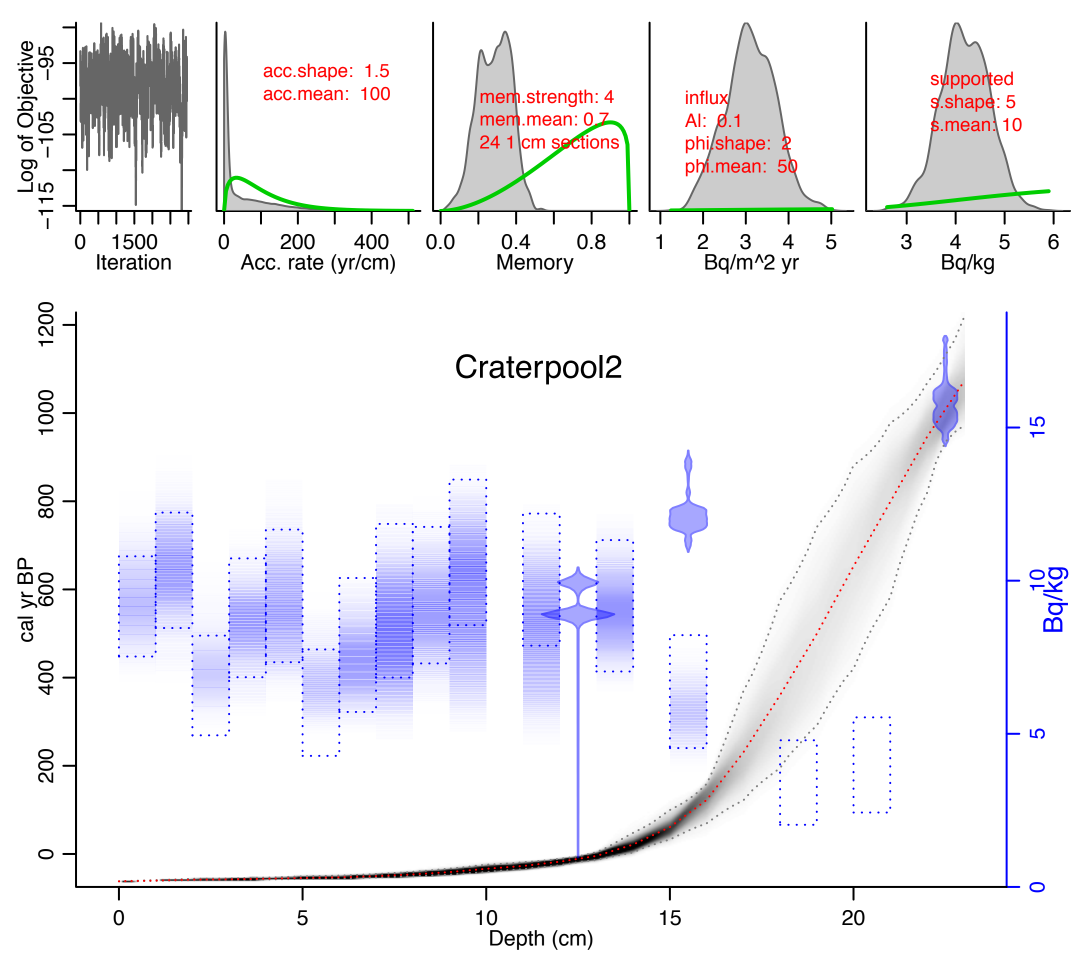

Log of Objective
−95
−105
−115
0
1500
Iteration
acc.shape: 1.5
acc.mean: 100
0
200
400
Acc. rate (yr/cm)
mem.strength: 4
mem.mean: 0.7
24 1 cm sections
0.0
0.4
0.8
Memory
influx
Al: 0.1
phi.shape: 2
phi.mean: 50
1
2
3
4
5
Bq/m^2 yr
supported
s.shape: 5
s.mean: 10
3
4
5
6
Bq/kg
Craterpool2
cal yr BP
0
200
400
600
800
1000
1200
Bq/kg
0
5
10
15
Depth (cm)
0
5
10
15
20

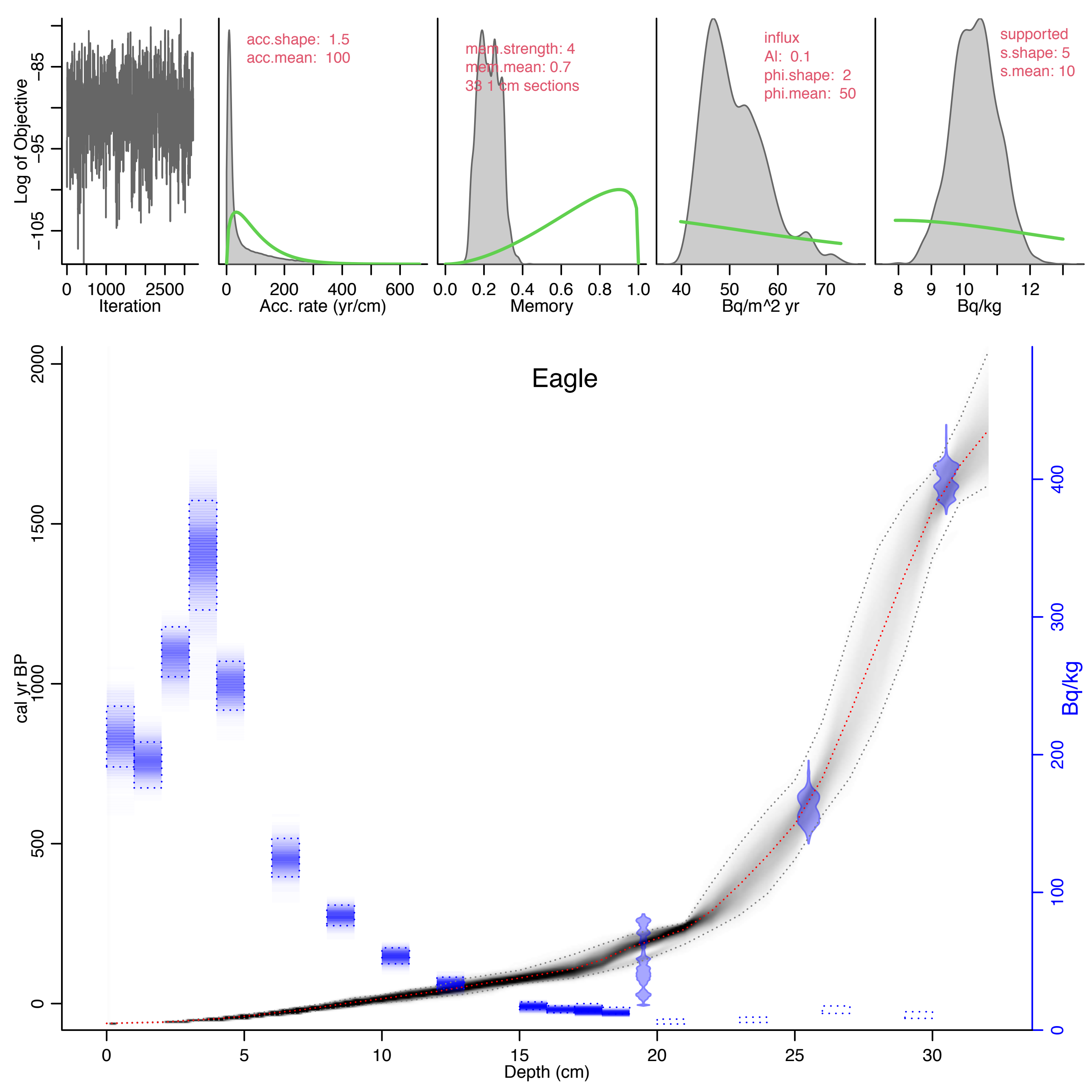

Log of Objective
−85
−95
−105
0 1000 2500
Iteration
acc.shape: 1.5
acc.mean: 100
0 200 400 600
Acc. rate (yr/cm)
mem.strength: 4
mem.mean: 0.7
33 1 cm sections
0.0 0.2 0.4 0.6 0.8 1.0
Memory
influx
Al: 0.1
phi.shape: 2
phi.mean: 50
40 50 60 70
Bq/m^2 yr
supported
s.shape: 5
s.mean: 10
8 9 10 12
Bq/kg
Eagle
cal yr BP
0 500 1000 1500 2000
Bq/kg
0 100 200 300 400
0 5 10 15 20 25 30
Depth (cm)

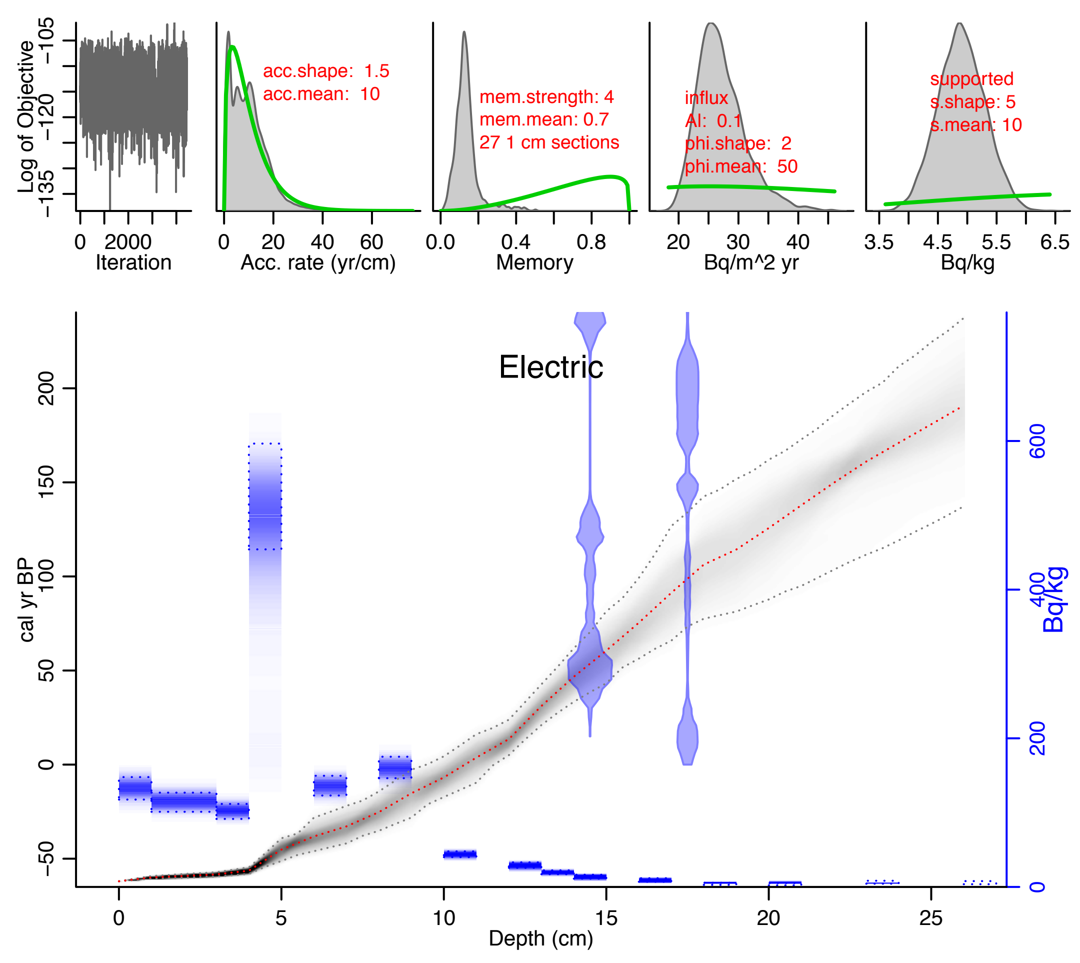

Log of Objective
Iteration
Acc. rate (yr/cm)
acc.shape: 1.5
acc.mean: 10
Memory
mem.strength: 4
mem.mean: 0.7
27 1 cm sections
Bq/m^2 yr
influx
Al: 0.1
phi.shape: 2
phi.mean: 50
Bq/kg
supported
s.shape: 5
s.mean: 10
Electric
cal yr BP
Depth (cm)
Bq/kg

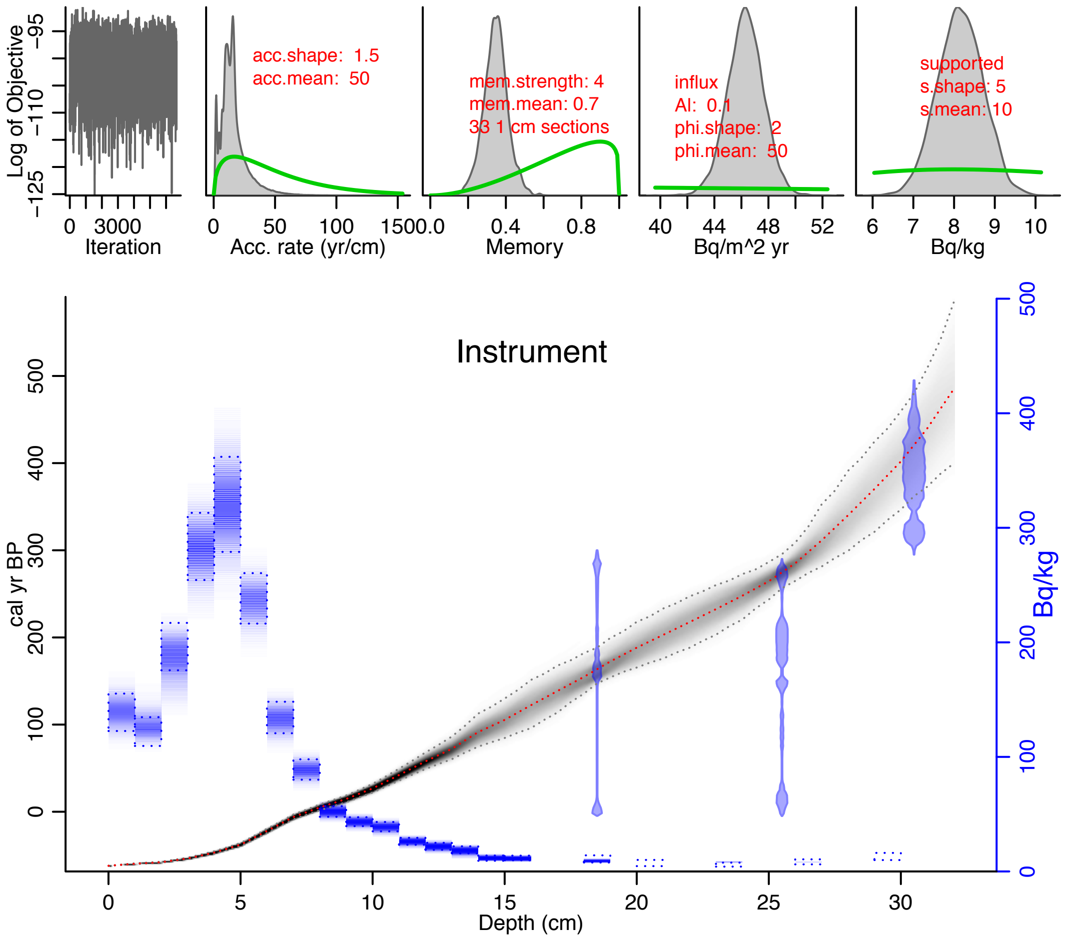

Log of Objective
Iteration
acc.shape: 1.5
acc.mean: 50
Acc. rate (yr/cm)
mem.strength: 4
mem.mean: 0.7
33 1 cm sections
Memory
influx
Al: 0.1
phi.shape: 2
phi.mean: 50
Bq/m^2 yr
supported
s.shape: 5
s.mean: 10
Bq/kg
Instrument
cal yr BP
Bq/kg
Depth (cm)

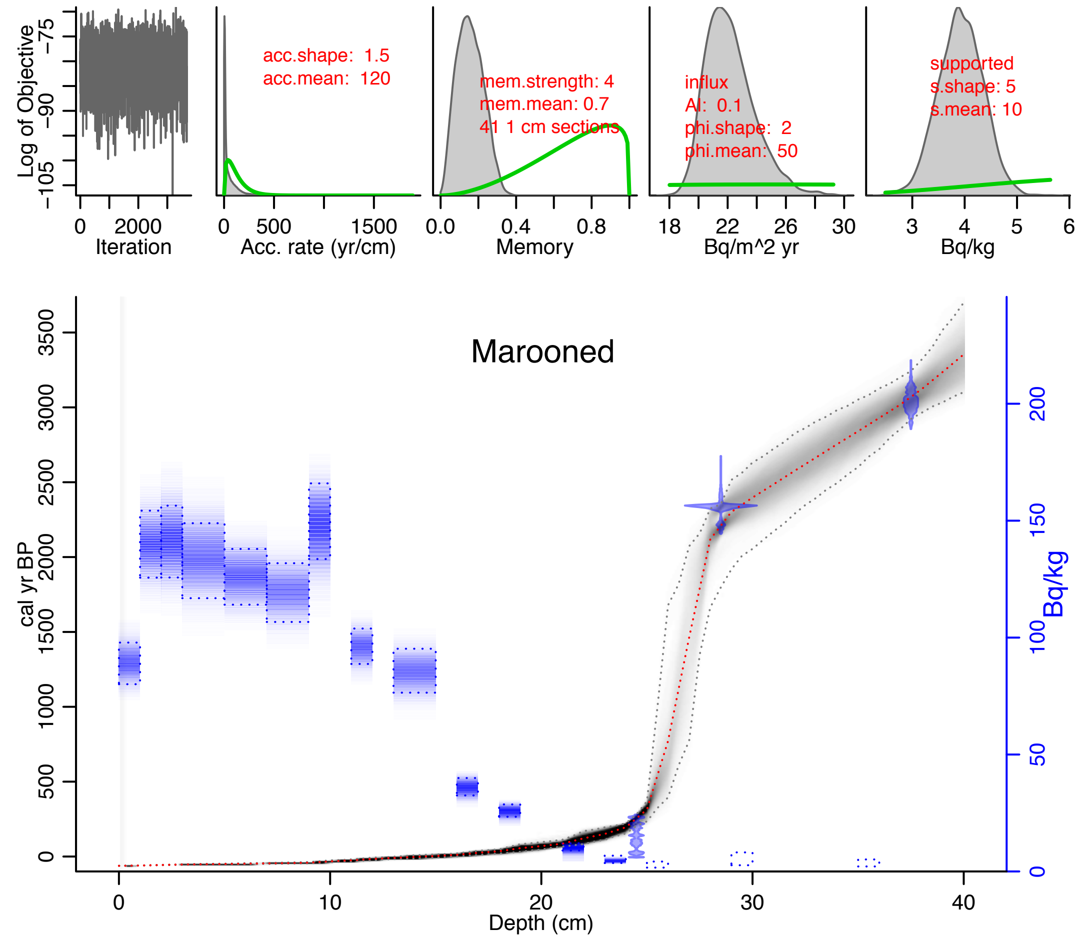

Log of Objective
Iteration
acc.shape: 1.5
acc.mean: 120
Acc. rate (yr/cm)
mem.strength: 4
mem.mean: 0.7
41 1 cm sections
Memory
influx
Al: 0.1
phi.shape: 2
phi.mean: 50
Bq/m^2 yr
supported
s.shape: 5
s.mean: 10
Bq/kg
Marooned
cal yr BP
Bq/kg
Depth (cm)

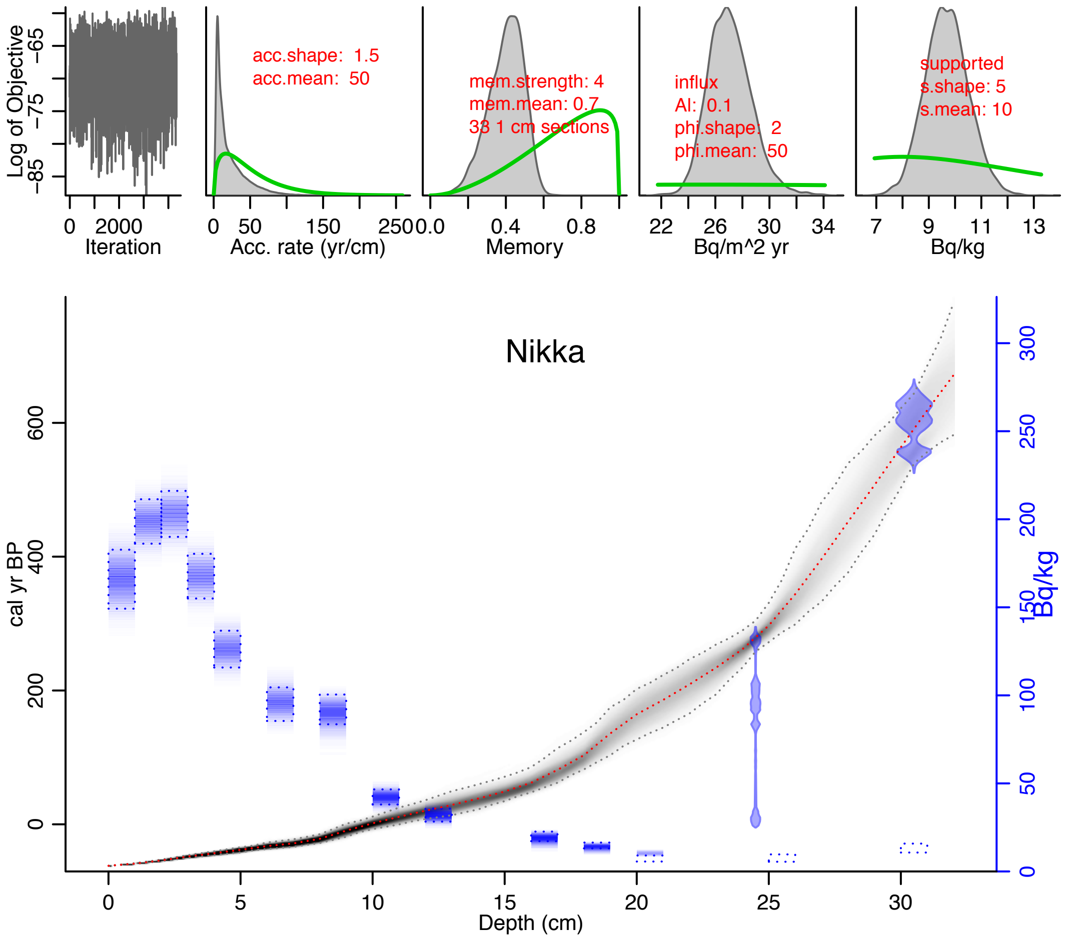

Log of Objective
−65
−75
−85
0
2000
Iteration
acc.shape: 1.5
acc.mean: 50
0
50
150
250
Acc. rate (yr/cm)
mem.strength: 4
mem.mean: 0.7
33 1 cm sections
0.0
0.4
0.8
Memory
influx
Al: 0.1
phi.shape: 2
phi.mean: 50
22
26
30
34
Bq/m^2 yr
supported
s.shape: 5
s.mean: 10
7
9
11
13
Bq/kg
Nikka
cal yr BP
0
200
400
600
Bq/kg
0
50
100
150
200
250
300
0
5
10
15
20
25
30
Depth (cm)

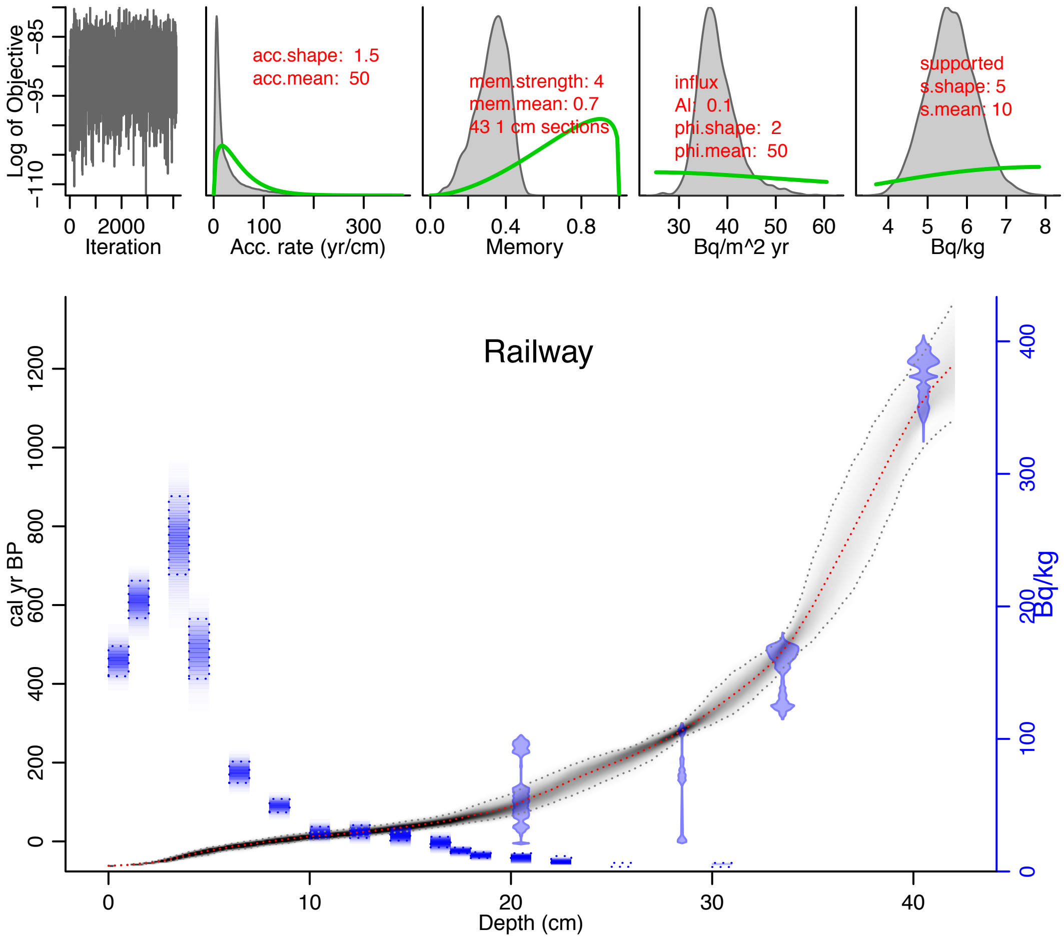

Log of Objective
Iteration
acc.shape: 1.5
acc.mean: 50
Acc. rate (yr/cm)
mem.strength: 4
mem.mean: 0.7
43 1 cm sections
Memory
influx
Al: 0.1
phi.shape: 2
phi.mean: 50
Bq/m^2 yr
supported
s.shape: 5
s.mean: 10
Bq/kg
Railway
cal yr BP
Bq/kg
Depth (cm)

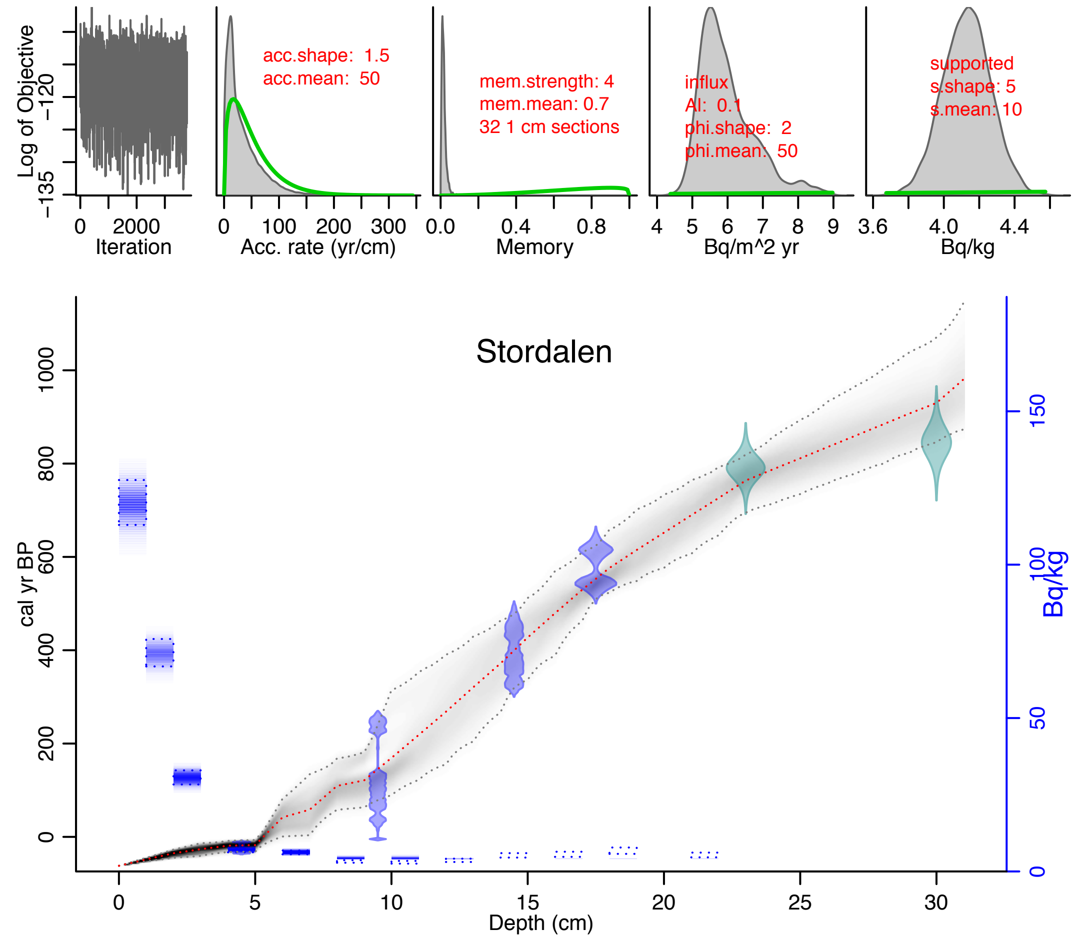
Log of Objective
−135
−120
0
2000
Iteration
0
100
200
300
Acc. rate (yr/cm)
acc.shape: 1.5
acc.mean: 50
0.0
0.4
0.8
Memory
mem.strength: 4
mem.mean: 0.7
32 1 cm sections
4
5
6
7
8
9
Bq/m^2 yr
influx
Al: 0.1
phi.shape: 2
phi.mean: 50
3.6
4.0
4.4
Bq/kg
supported
s.shape: 5
s.mean: 10
Stordalen
cal yr BP
0
200
400
600
800
1000
Depth (cm)
0
5
10
15
20
25
30
Bq/kg
0
50
100
150

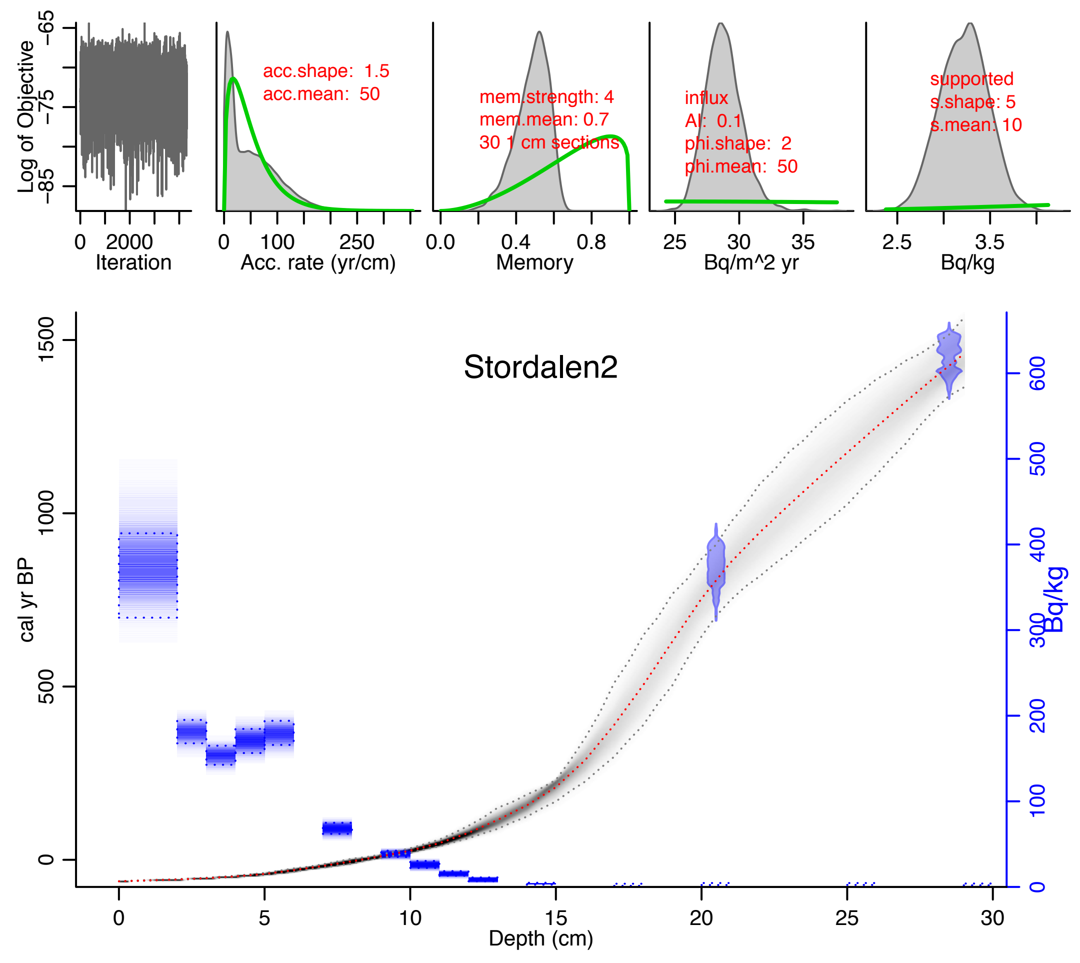
Log of Objective
−65
−75
−85
0
2000
Iteration
acc.shape: 1.5
acc.mean: 50
0
100
250
Acc. rate (yr/cm)
mem.strength: 4
mem.mean: 0.7
30 1 cm sections
0.0
0.4
0.8
Memory
influx
Al: 0.1
phi.shape: 2
phi.mean: 50
25
30
35
Bq/m^2 yr
supported
s.shape: 5
s.mean: 10
2.5
3.5
Bq/kg
Stordalen2
cal yr BP
1500
1000
500
0
Depth (cm)
0
5
10
15
20
25
30
Bq/kg
600
500
400
300
200
100
0

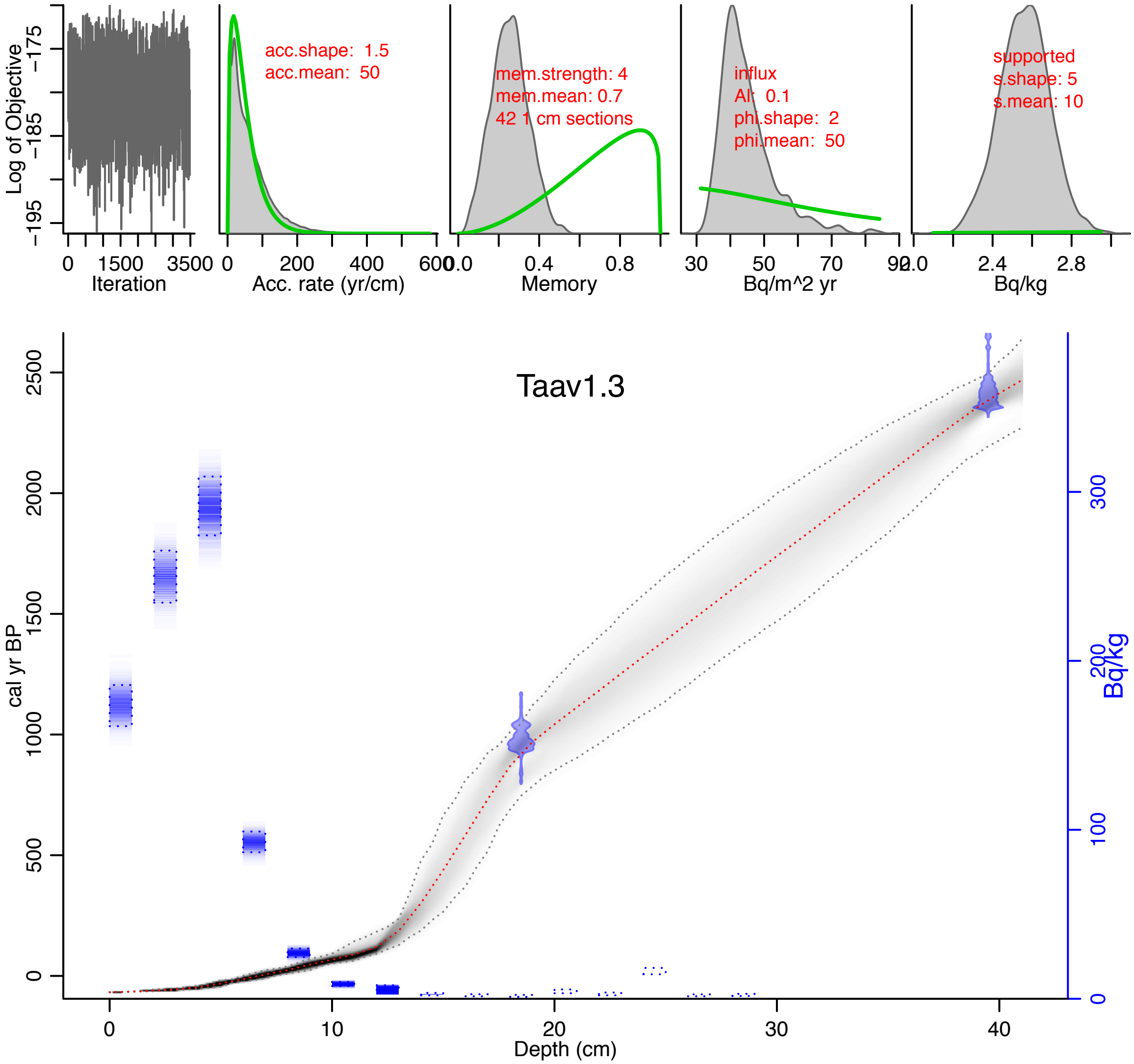
Log of Objective
−175
−185
−195
0
1500
3500
Iteration
acc.shape: 1.5
acc.mean: 50
0
200
400
600
Acc. rate (yr/cm)
mem.strength: 4
mem.mean: 0.7
42 1 cm sections
0.0
0.4
0.8
Memory
influx
Al: 0.1
phi.shape: 2
phi.mean: 50
30
50
70
90
Bq/m^2 yr
supported
s.shape: 5
s.mean: 10
2.0
2.4
2.8
Bq/kg
Taav1.3
cal yr BP
2500
2000
1500
1000
500
0
Bq/kg
300
200
100
0
Depth (cm)
0
10
20
30
40

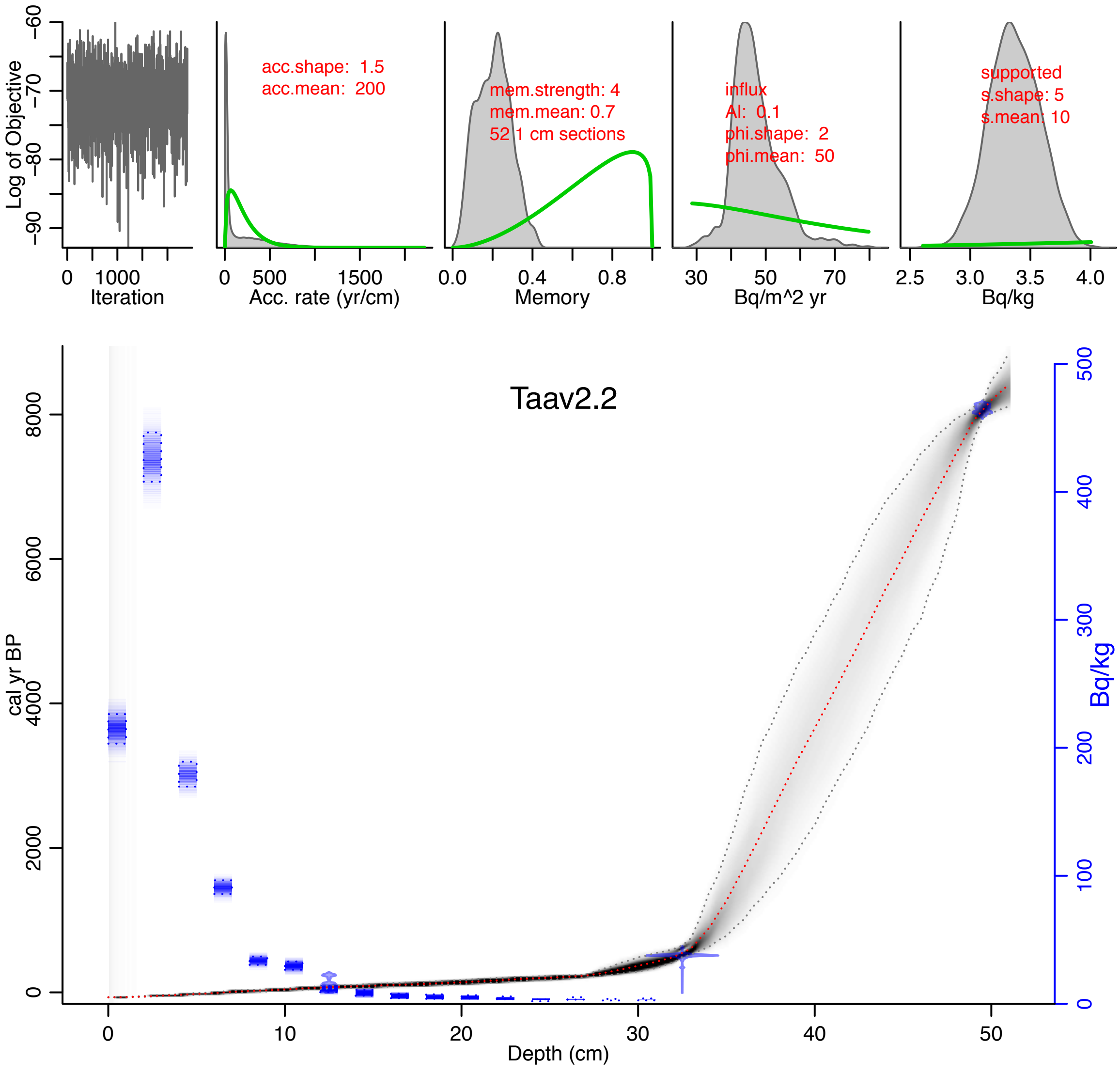
Log of Objective
Iteration
acc.shape: 1.5
acc.mean: 200
Acc. rate (yr/cm)
mem.strength: 4
mem.mean: 0.7
52 1 cm sections
Memory
influx
Al: 0.1
phi.shape: 2
phi.mean: 50
Bq/m^2 yr
supported
s.shape: 5
s.mean: 10
Bq/kg
Taav2.2
cal yr BP
Bq/kg
Depth (cm)

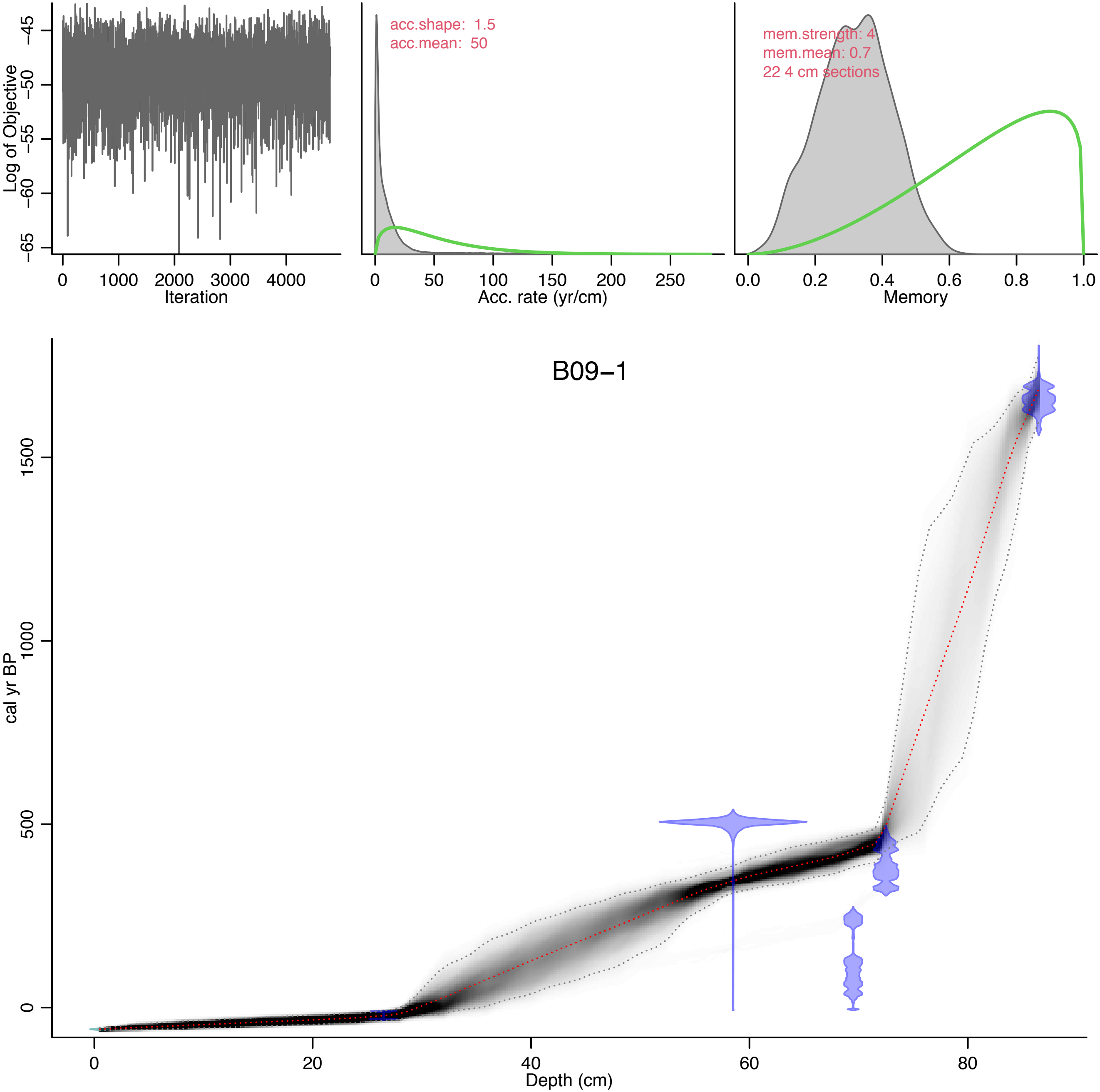

Log of Objective
−45
−50
−55
−60
−65
0
1000
2000
3000
4000
Iteration
acc.shape: 1.5
acc.mean: 50
0
50
100
150
200
250
Acc. rate (yr/cm)
mem.strength: 4
mem.mean: 0.7
22 4 cm sections
0.0
0.2
0.4
0.6
0.8
1.0
Memory
B09−1
cal yr BP
0
500
1000
1500
0
20
40
60
80
Depth (cm)

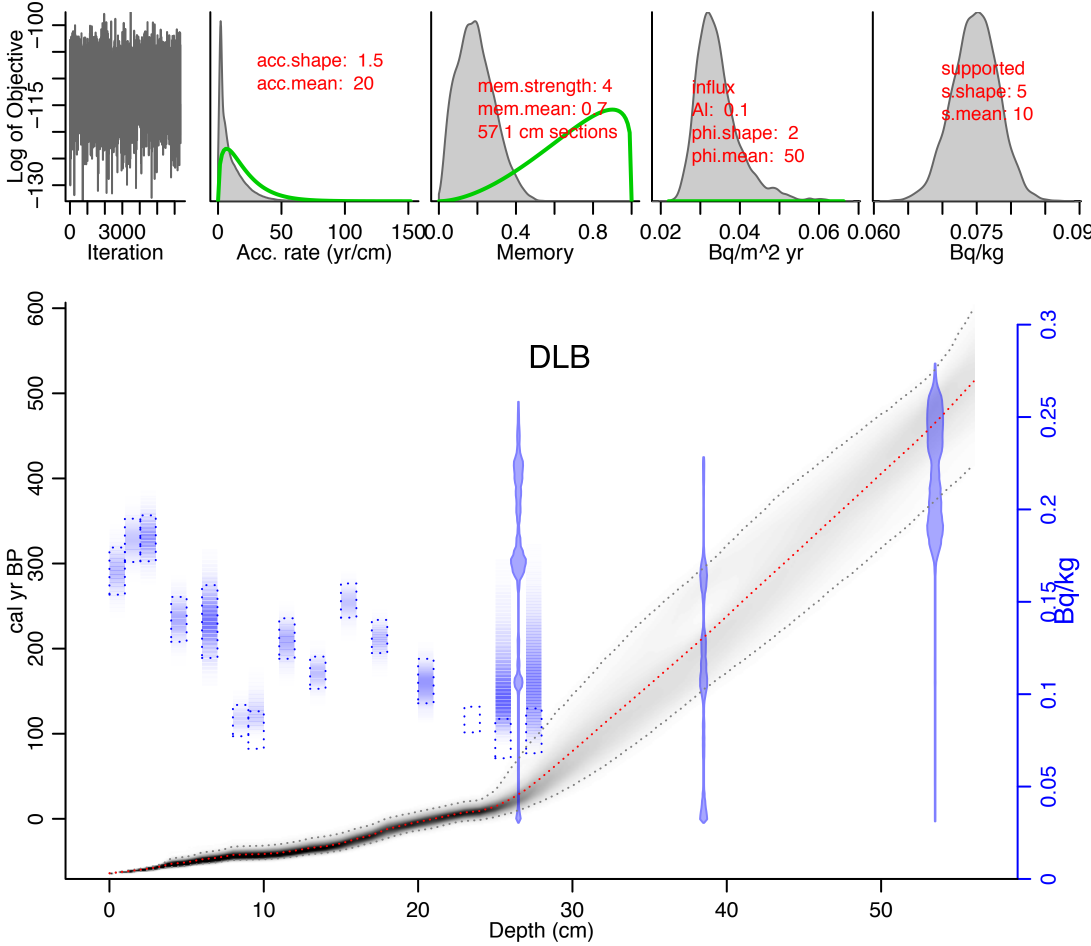

Log of Objective
Iteration
acc.shape: 1.5
acc.mean: 20
Acc. rate (yr/cm)
mem.strength: 4
mem.mean: 0.7
57 1 cm sections
Memory
influx
Al: 0.1
phi.shape: 2
phi.mean: 50
Bq/m^2 yr
supported
s.shape: 5
s.mean: 10
Bq/kg
DLB
cal yr BP
Depth (cm)
Bq/kg

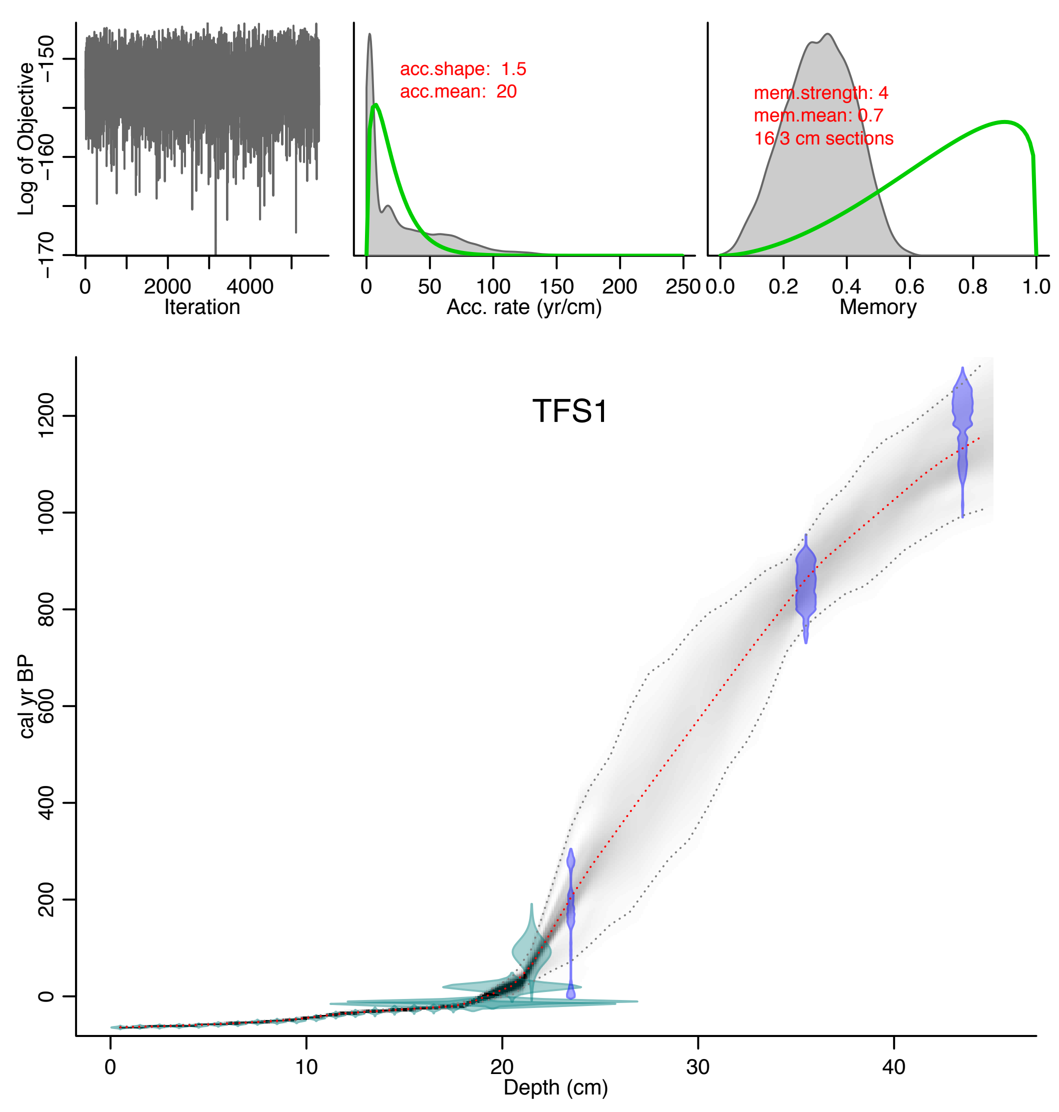

Log of Objective
−150
−160
−170
0
2000
4000
Iteration
acc.shape: 1.5
acc.mean: 20
0
50
100
150
200
250
Acc. rate (yr/cm)
mem.strength: 4
mem.mean: 0.7
16 3 cm sections
0.0
0.2
0.4
0.6
0.8
1.0
Memory
TFS1
cal yr BP
1200
1000
800
600
400
200
0
0
10
20
30
40
Depth (cm)

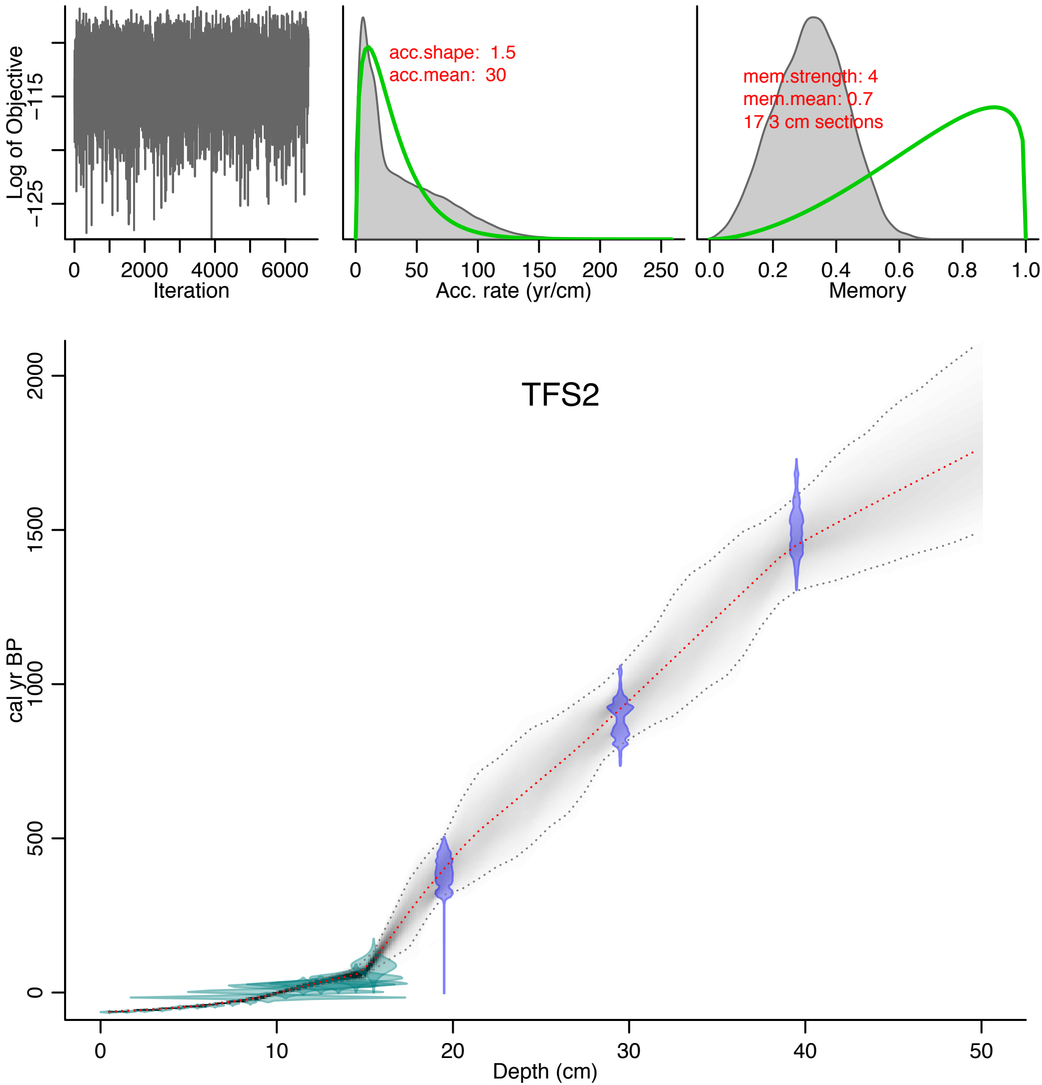

Log of Objective
−115
−125
0
2000
4000
6000
Iteration
acc.shape: 1.5
acc.mean: 30
0
50
100
150
200
250
Acc. rate (yr/cm)
mem.strength: 4
mem.mean: 0.7
17 3 cm sections
0.0
0.2
0.4
0.6
0.8
1.0
Memory
TFS2
cal yr BP
2000
1500
1000
500
0
0
10
20
30
40
50
Depth (cm)

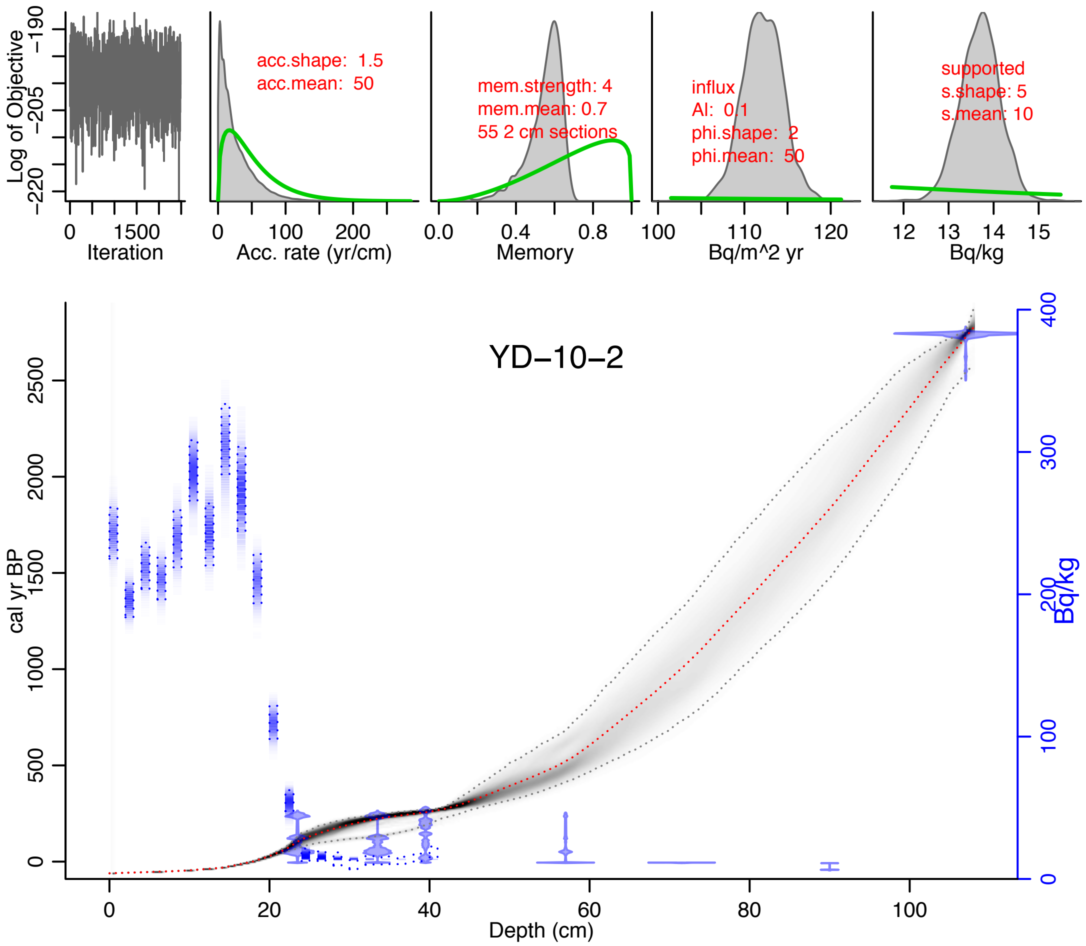
Log of Objective
−190
−205
−220
0
1500
Iteration
acc.shape: 1.5
acc.mean: 50
0
100
200
Acc. rate (yr/cm)
mem.strength: 4
mem.mean: 0.7
55 2 cm sections
0.0
0.4
0.8
Memory
influx
Al: 0.1
phi.shape: 2
phi.mean: 50
100
110
120
Bq/m^2 yr
supported
s.shape: 5
s.mean: 10
12
13
14
15
Bq/kg
YD−10−2
cal yr BP
0
500
1000
1500
2000
2500
Depth (cm)
0
20
40
60
80
100
Bq/kg
0
100
200
300
400

Supplementary Figure 3

Comparison of water-table reconstructions of each record using different transfer functions. The selection of transfer functions for each record was based on the geographic locations of the study sites: European and Holarctic transfer functions for European sites; North American and Holarctic transfer functions for North American sites; Asian transfer function for Asian sites; European, Holarctic, and Asian transfer functions for Asian sites located in western Siberian Russian that may also influenced by European Russian climate. Red line indicates the Holarctic transfer function, black line indicates the regional transfer function (either European, North American or Asian transfer function), blue line indicates reconstruction based on local transfer function when the Holarctic and regional ones are not applicable, unless otherwise specified in the figure.

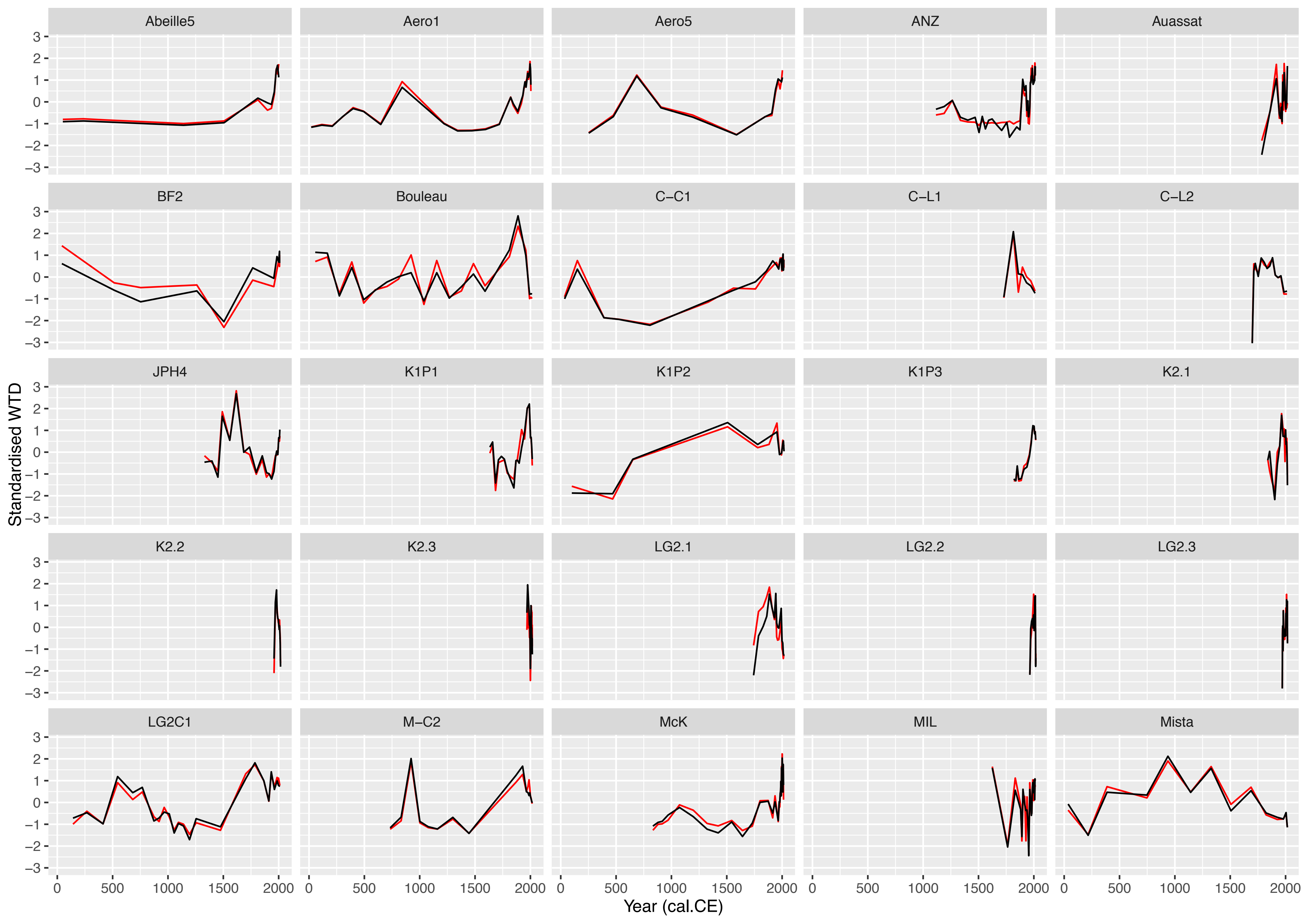
Abeille5
Aero1
Aero5
ANZ
Auassat
BF2
Bouleau
C–C1
C–L1
C–L2
JPH4
K1P1
K1P2
K1P3
K2.1
K2.2
K2.3
LG2.1
LG2.2
LG2.3
LG2C1
M–C2
McK
MIL
Mista
Standardised WTD
Year (cal.CE)
3
2
1
0
−1
−2
−3
0
500
1000
1500
2000

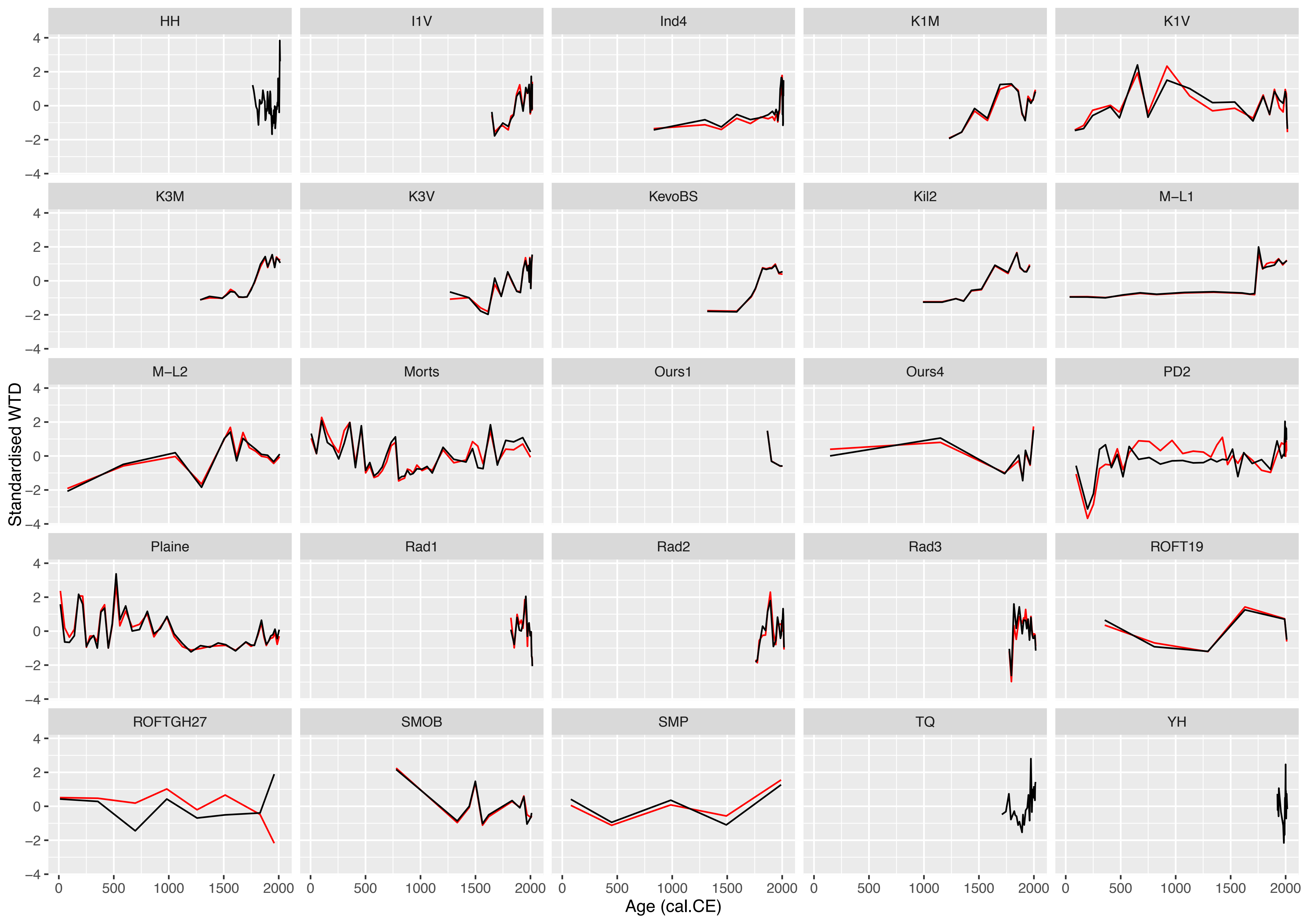
HH
I1V
Ind4
K1M
K1V
K3M
K3V
KevoBS
Kil2
M−L1
M−L2
Morts
Ours1
Ours4
PD2
Plaine
Rad1
Rad2
Rad3
ROFT19
ROFTGH27
SMOB
SMP
TQ
YH
Standardised WTD
Age (cal.CE)
4
2
0
−2
−4
0
500
1000
1500
2000

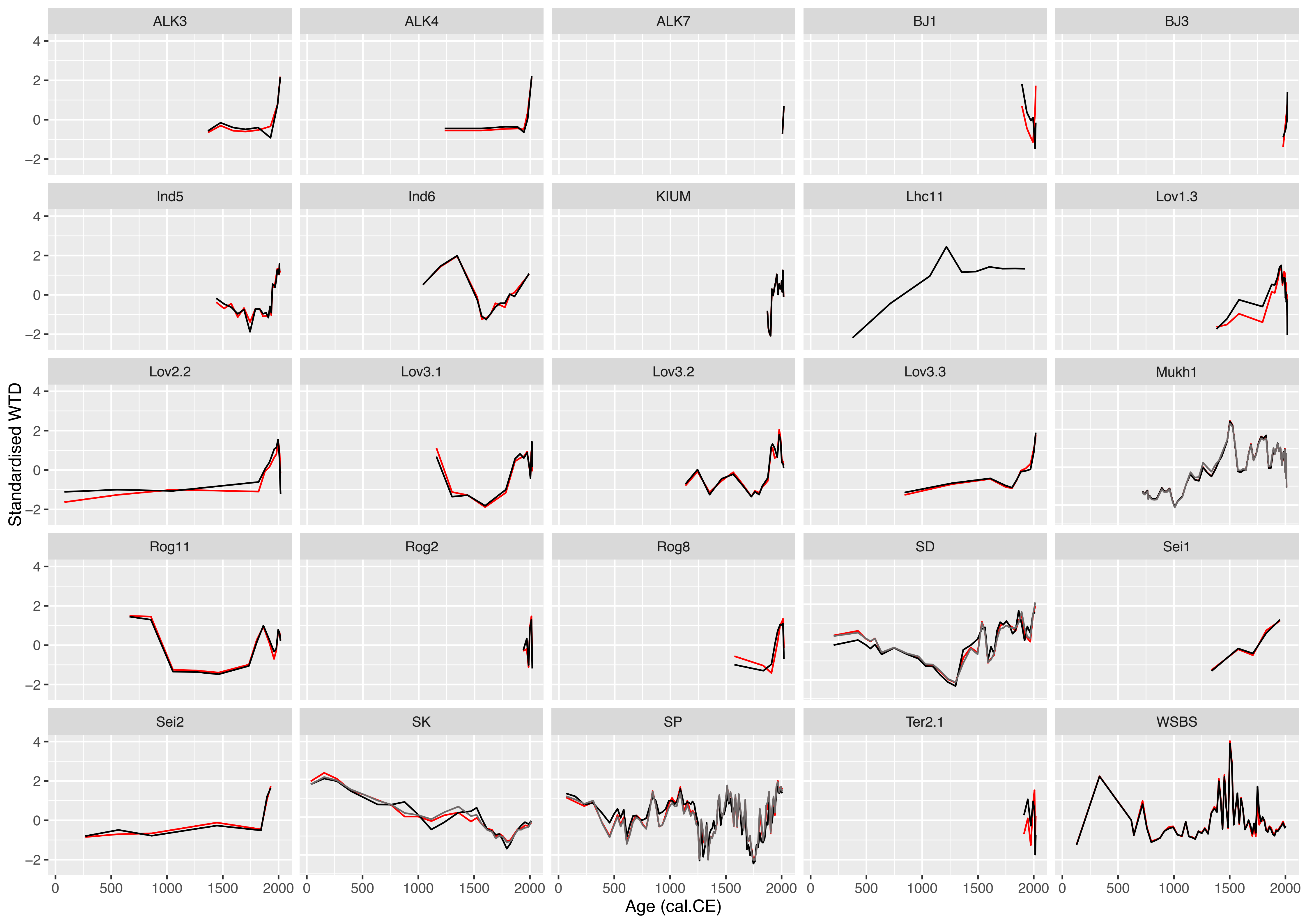

ALK3
ALK4
ALK7
BJ1
BJ3
Ind5
Ind6
KIUM
Lhc11
Lov1.3
Lov2.2
Lov3.1
Lov3.2
Lov3.3
Mukh1
Rog11
Rog2
Rog8
SD
Sei1
Sei2
SK
SP
Ter2.1
WSBS
Standardised WTD
Age (cal.CE)
4
2
0
−2
0
500
1000
1500
2000

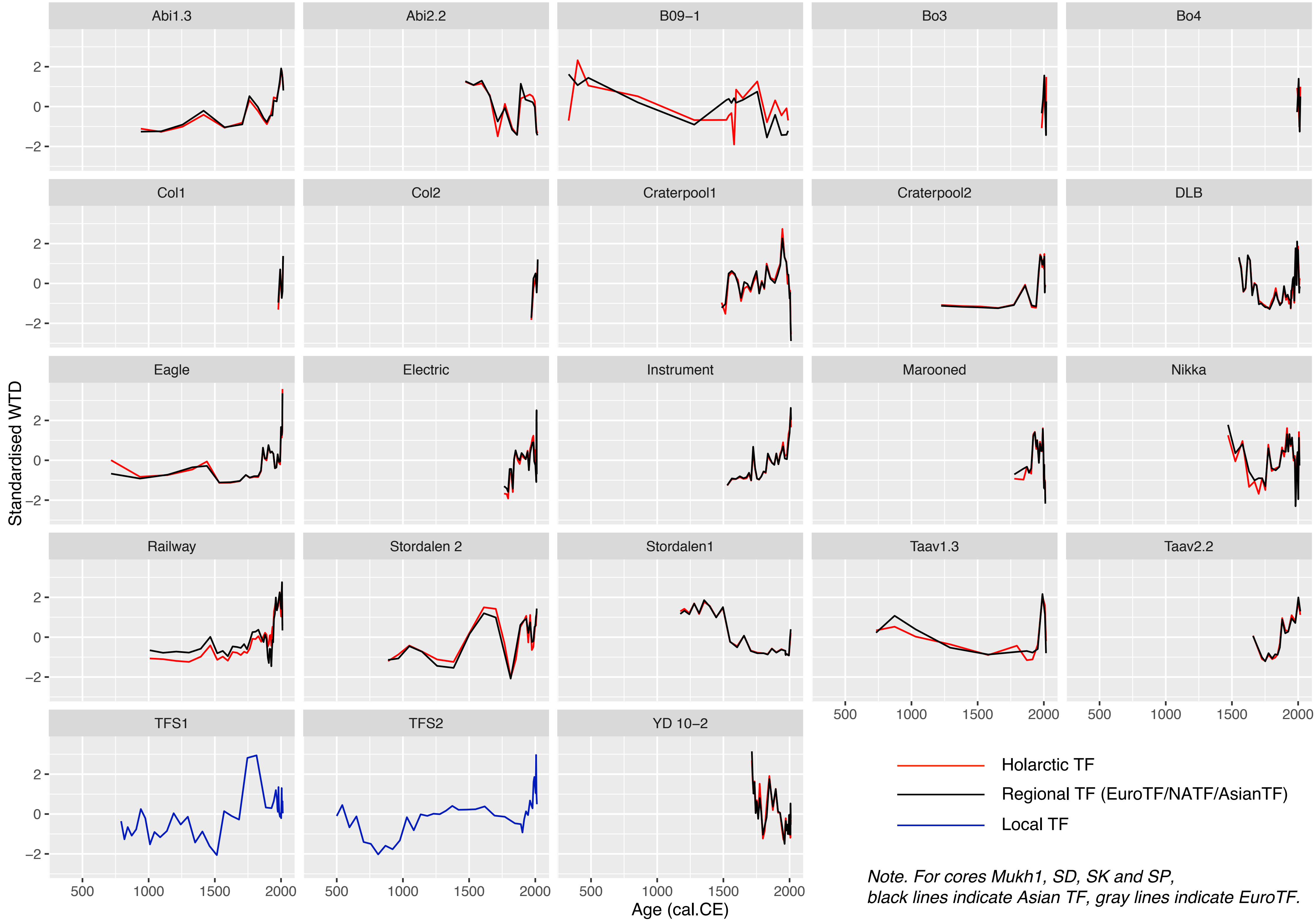

Note. For cores Mukh1, SD, SK and SP, black lines indicate Asian TF, gray lines indicate EuroTF.

Supplementary Figure 4

Water-table reconstructions of 98 records using the best transfer function (selected based on Supplementary Figure 3) on the datasets with and without weak silicic idiosomic test (*Corythion*, *Euglypha*, and *Trinema* spp.). Black line indicates reconstruction with weak silicic idiosomic test included, blue line indicates reconstruction without weak silicic idiosomic test included.

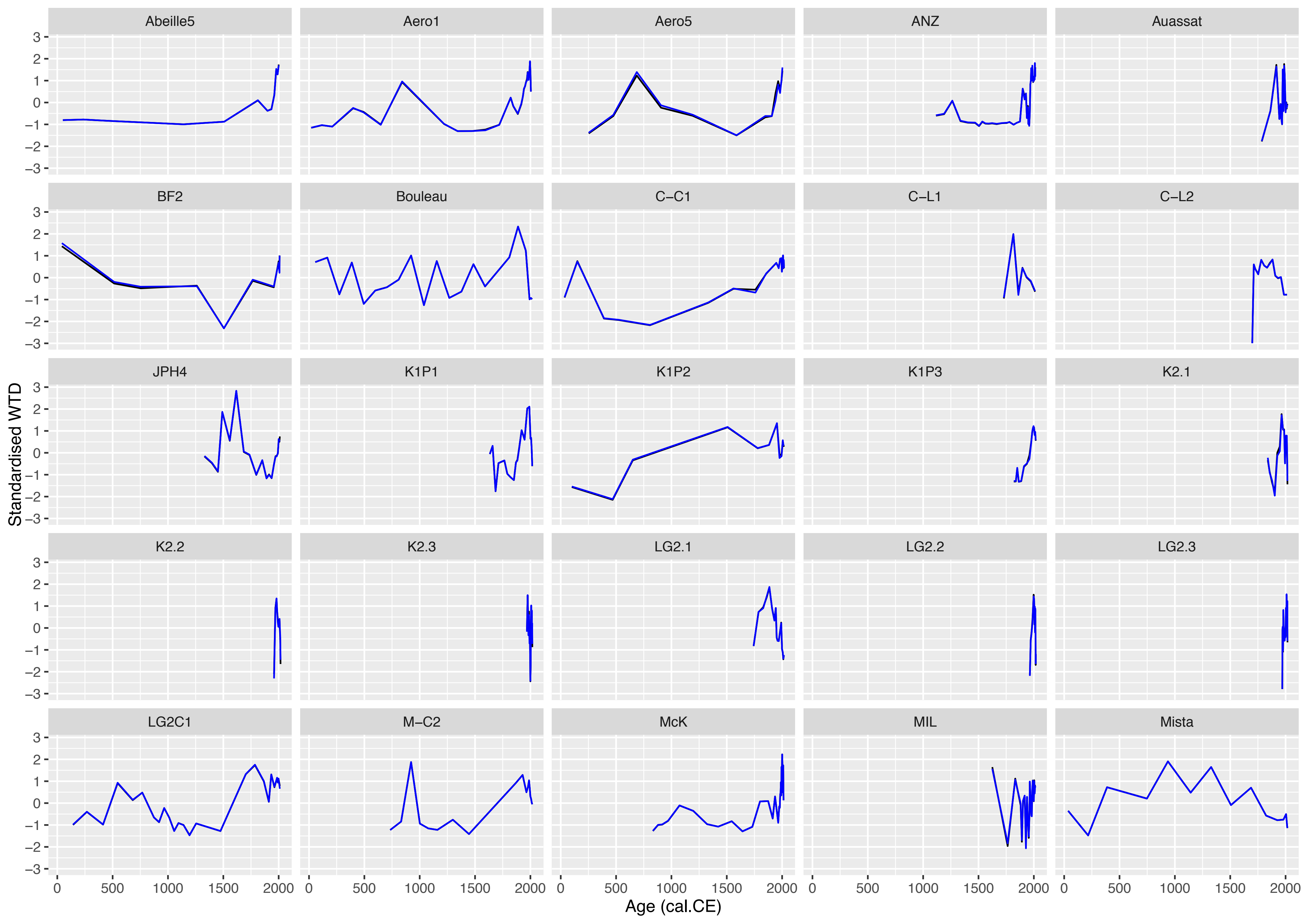

Abeille5
Aero1
Aero5
ANZ
Auassat
BF2
Bouleau
C–C1
C–L1
C–L2
JPH4
K1P1
K1P2
K1P3
K2.1
K2.2
K2.3
LG2.1
LG2.2
LG2.3
LG2C1
M–C2
McK
MIL
Mista
Standardised WTD
Age (cal.CE)

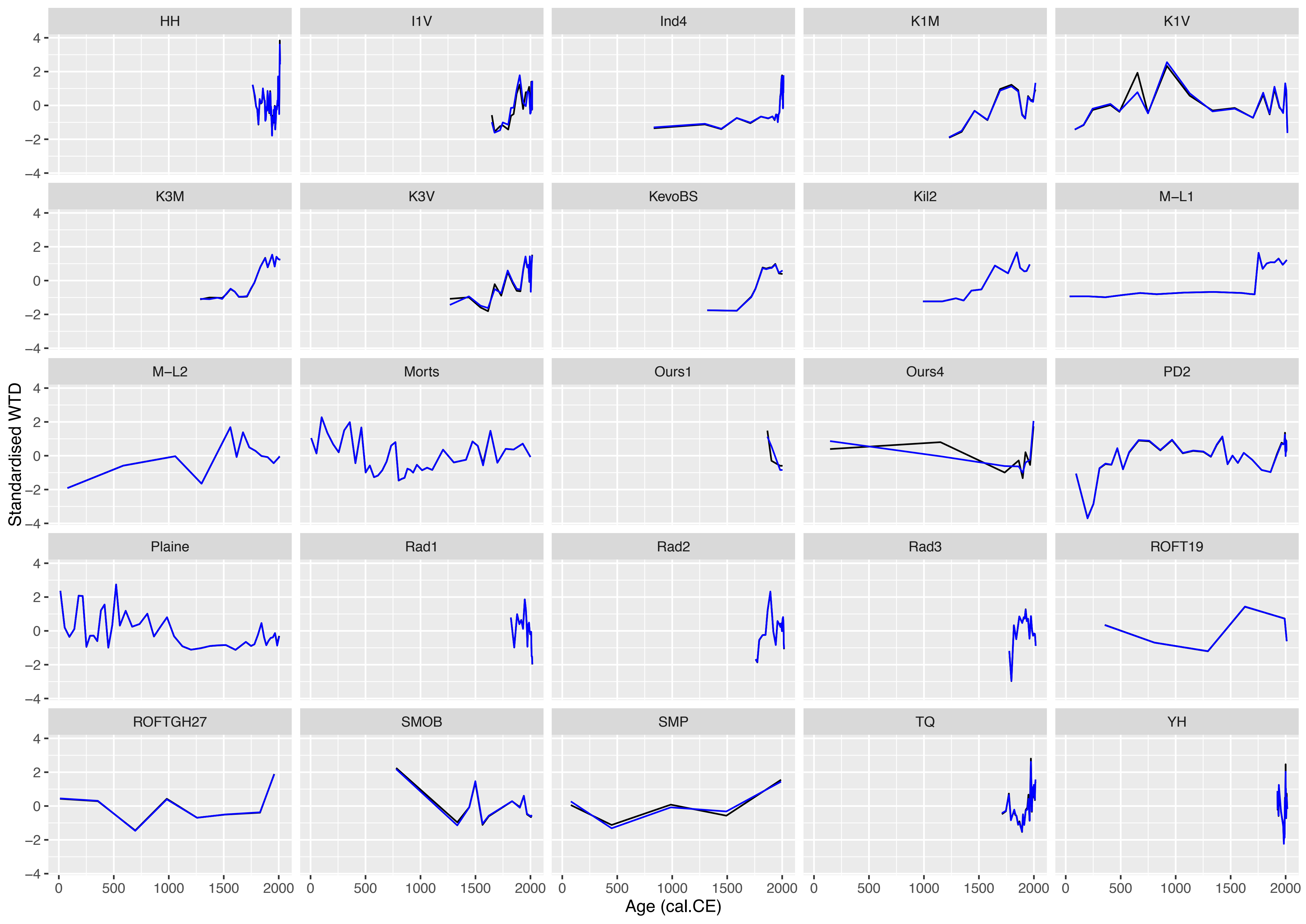
HH
I1V
Ind4
K1M
K1V
K3M
K3V
KevoBS
Kil2
M−L1
M−L2
Morts
Ours1
Ours4
PD2
Plaine
Rad1
Rad2
Rad3
ROFT19
ROFTGH27
SMOB
SMP
TQ
YH
Standardised WTD
Age (cal.CE)
4
2
0
−2
−4
0
500
1000
1500
2000

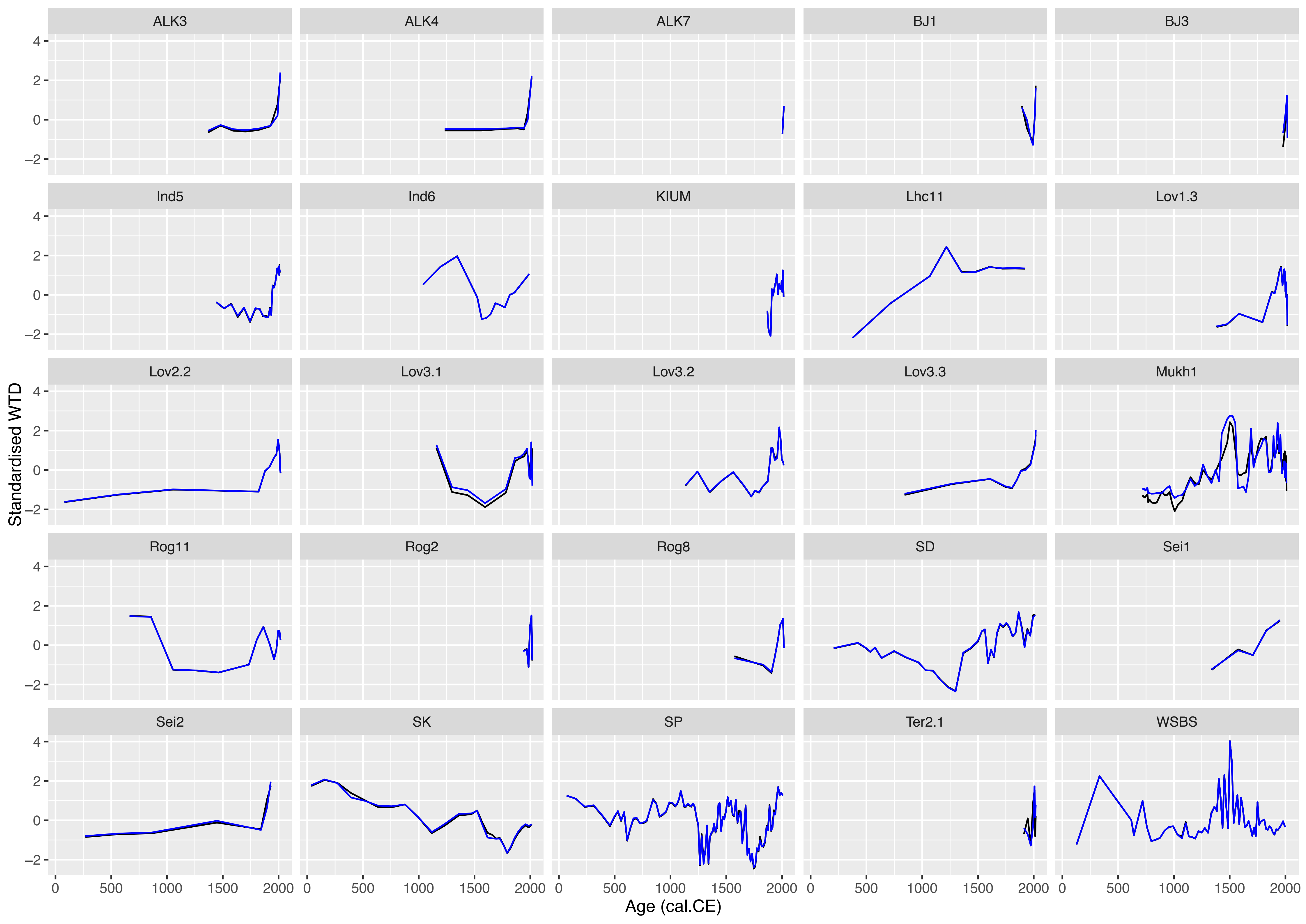

ALK3
ALK4
ALK7
BJ1
BJ3
Ind5
Ind6
KIUM
Lhc11
Lov1.3
Lov2.2
Lov3.1
Lov3.2
Lov3.3
Mukh1
Rog11
Rog2
Rog8
SD
Sei1
Sei2
SK
SP
Ter2.1
WSBS
Standardised WTD
Age (cal.CE)
4
2
0
−2
0
500
1000
1500
2000

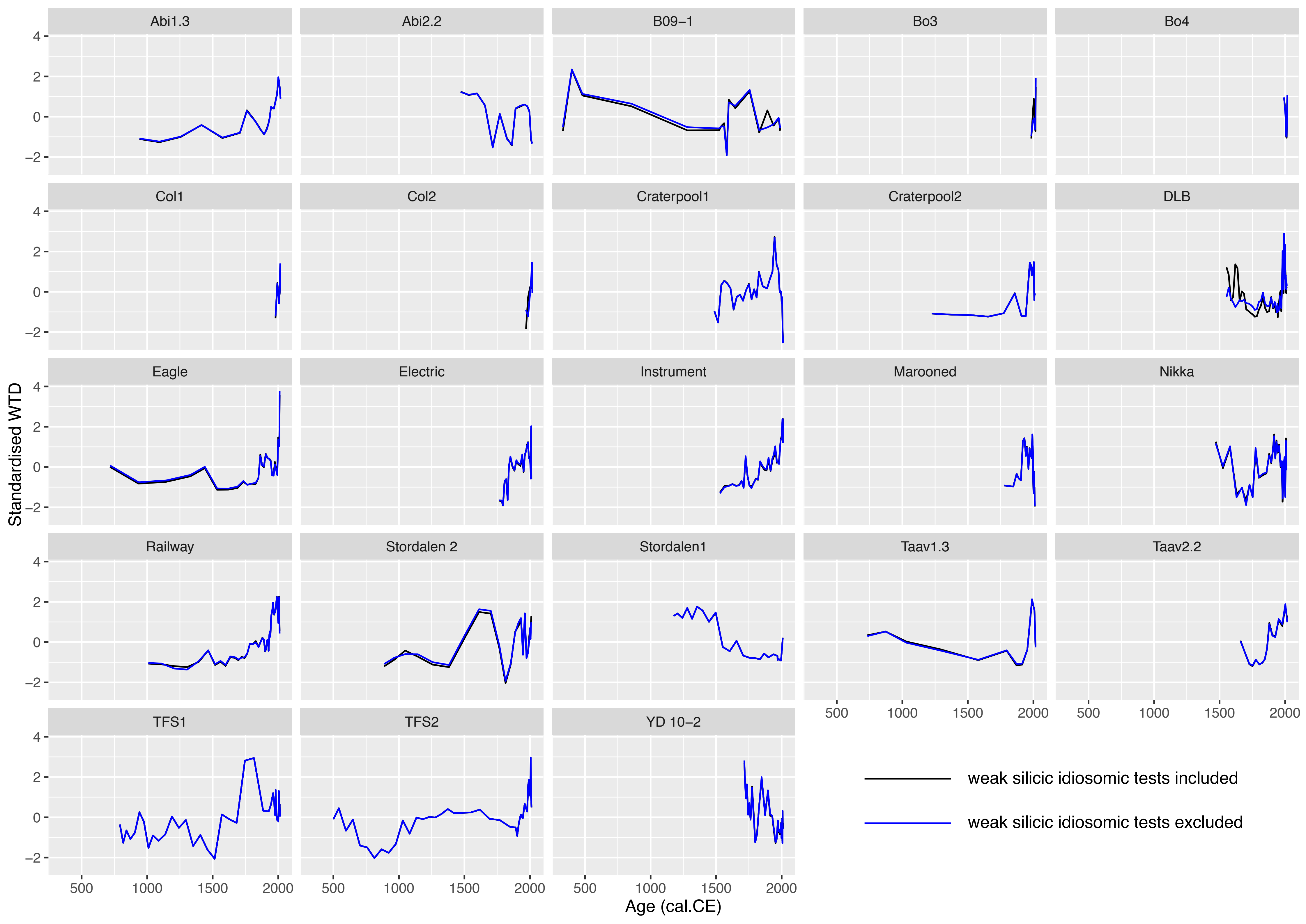
Abi1.3
Abi2.2
B09−1
Bo3
Bo4
Col1
Col2
Craterpool1
Craterpool2
DLB
Eagle
Electric
Instrument
Marooned
Nikka
Railway
Stordalen 2
Stordalen1
Taav1.3
Taav2.2
TFS1
TFS2
YD 10−2
Standardised WTD
Age (cal.CE)
4
2
0
−2
500
1000
1500
2000
weak silicic idiosomic tests included
weak silicic idiosomic tests excluded
