# Supplementary material for: Recent climate change has driven divergent hydrological shifts in high-latitude peatlands
Source: Nat Commun. 2022 Aug 24;13:4959. doi: 10.1038/s41467-022-32711-4 (PMC9402595; doi:10.1038/s41467-022-32711-4)
Supplement: Supplementary file 2 — Description of Additional Supplementary Files [file 41467_2022_32711_MOESM2_ESM.pdf]

Description of Additional Supplementary Files

File Name: Supplementary Data 1
Description: Study site and testate amoebae taxon harmonisation information.
